# Desymmetrization of Prochiral Cyclobutanones via Nitrogen Insertion: A Concise Route to Chiral γ‐Lactams

**DOI:** 10.1002/anie.202100642

**Published:** 2021-03-10

**Authors:** Jan Sietmann, Mike Ong, Christian Mück‐Lichtenfeld, Constantin G. Daniliuc, Johannes M. Wahl

**Affiliations:** ^1^ Organisch-Chemisches Institut Westfälische Wilhelms-Universität Corrensstrasse 36 48149 Münster Germany; ^2^ Department Chemie Johannes Gutenberg-Universität Duesbergweg 10–14 55128 Mainz Germany

**Keywords:** asymmetric synthesis, cyclobutanone, desymmetrization, γ-lactams

## Abstract

Asymmetric access to γ‐lactams is achieved via a cyclobutanone ring expansion using widely available (1S,2R)‐1‐amino‐2‐indanol for chiral induction. Mechanistic analysis of the key N,O‐ketal rearrangement reveals a Curtin–Hammett scenario, which enables a downstream stereoinduction (up to 88:12 dr) and is corroborated by spectroscopic, crystallographic, and computational studies. In combination with an easy deprotection protocol, this operationally simple sequence allows the synthesis of a range of optically pure γ‐lactams, including those bearing all‐carbon quaternary stereocenters. In addition, the formal synthesis of drug molecules baclofen, brivaracetam, and pregabalin further demonstrates the synthetic utility and highlights the general applicability of the presented method.

## Graphical abstract

(1*S*,2*R*)‐2‐Amino‐1‐indanol was identified as a chiral nitrogen source in an oxidative rearrangement of prochiral cyclobutanones. Building on strain release as the driving force, this downstream stereoinduction enables a variety of γ‐lactams to be synthesized including those bearing quaternary stereocenters. In addition, drug molecules pregabalin, baclofen, and brivaracetam are accessible, which underlines the broad applicability of this method.

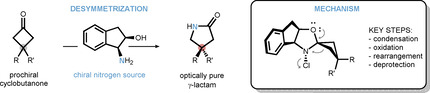

## Introduction

**Scheme 1:**
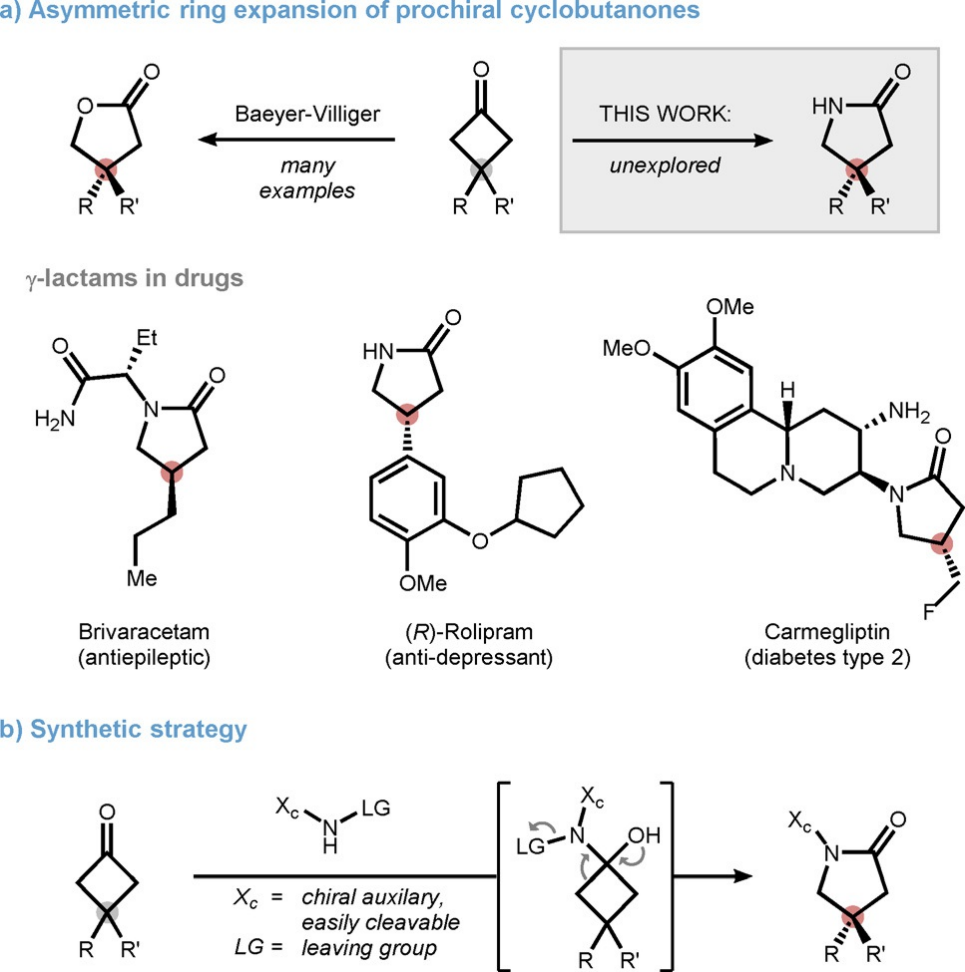
Access to chiral γ‐lactams via nitrogen‐based ring expansion of cyclobutanones.

In light of the importance of cyclic networks in nature, ring expansions rank among the most attractive transformations in chemistry. In many cases, these ring expansions represent the preferred route to medium rings that are kinetically difficult to access by ring closure.^[1]^ Additionally, five‐ to seven‐membered rings can be synthesized from smaller rings harnessing the inherent ring strain as a driving force for reaction progress. As such, rearrangements of prochiral cyclobutanones are particularly intriguing, as they allow for distal stereoinduction through a desymmetrization strategy.^[2]^ An archetypal example is the Baeyer–Villiger oxidation, which has found numerous applications for the synthesis of chiral γ‐lactones under remarkably mild conditions (Scheme 1 a, top).[^3^, ^4^] The related asymmetric nitrogen‐based ring expansion, which leads to chiral γ‐lactams, represents an underexplored obstacle, despite the significance of this motif in drug discovery.[^5^, ^6^] To date, many approved and investigational drugs such as brivaracetam,^[7]^ rolipram,^[8]^ or carmegliptin^[9]^ contain β‐substituted γ‐lactams providing further incentive for the development of such ring‐expansions (Scheme 1 a, bottom). The lack of general methods in this area plausibly arises from the highly acidic reaction media required for Beckmann and Schmidt rearrangements, which poses a considerable challenge when performing these reactions asymmetrically.^[10]^

To address this problem, we envisaged a synthetic strategy based on a bi‐functionalized amine bearing a suitable leaving group and a chiral auxiliary X_c_ (Scheme 1 b).^[11]^ In contrast to the classical Beckmann reaction, we hypothesized that due to the inherent ring strain, rearrangement can occur from a hemiaminal intermediate (and prior to oxime formation) when using an adequately potent leaving group.^[12]^ Migration of one of the two prochiral C−C bonds can then be directed by the chiral auxiliary enabling, after separation of the diastereomers and deprotection, a concise route to enantiopure lactams.[^13^, ^14^] In this Research Article, our results on identifying a suitable amine motif are presented, along with detailed mechanistic analysis, deprotection protocols, and synthetic applications.

## Results and Discussion

We commenced our study by exploring different leaving groups at the nitrogen atom to better understand their ability to trigger the postulated rearrangement (cf. Scheme 1 b). Hydroxylamine derivatives bearing an acetate or benzoate group were generally not potent enough and no rearrangement was observed (see SI for further information). Based on Fujioka's studies concerning halonium‐initiated rearrangements of cyclobutanone aminals and ketals,^[15]^ we started exploring chiral aminoalcohols that can be activated in situ by an appropriate halogen source. Thus, we identified aminoindanol **2** and trichloroisocyanuric acid (TCICA) as a formidable combination to convert prochiral 3‐phenylcyclobutanone **1 a** to the corresponding γ‐lactam **3 a** (Table 1).^[16]^

**Table 1:** Reaction optimization using (1*S*,2*R*)‐1‐amino‐2‐indanol for asymmetric induction. 
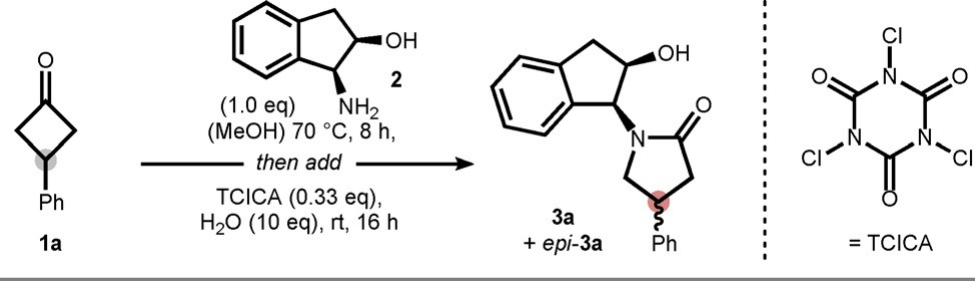

Entry	Change from standard conditions^[a]^	Yield [%]^[b]^ **3 a**+*epi‐* **3 a**	dr^[b]^	Yield [%]^[b,c]^ of major **3 a**
1	–	96	80:20	77 (69)
2	9.0 mmol scale	n.d.	80:20	(68)
3	THF instead of MeOH	24	85:15	20
4	CH_2_Cl_2_ instead of MeOH	68	87:13	59
5	DMF instead of MeOH	50	77:23	39
6	−30 °C instead of rt	48	87:13	42
7	No water added	77	81:19	62
8	NBS instead of TCICA	55	78:22	43
9	NCS instead of TCICA	98	80:20	78

[a] Reactions were run on a 1.0 mmol scale in 5.0 mL of solvent (0.2 m). [b] Determined by ^1^H NMR using mesitylene as an internal standard. [c] Yield of isolated major isomer **3 a** in parentheses. THF=tetrahydrofuran, DMF=dimethylformamide, n.d.=not determined.

Under the optimized conditions (see SI for further information), high yield was achieved on small as well as gram‐scale (Table 1, entries 1 and 2). Considering the distance of the stereocenters, the diastereomeric ratio (dr) of 80:20 was quite pleasing, especially since both isomers were easily separable by column chromatography. Changing the solvent as well as decreasing the temperature led to incomplete conversions and/or the formation of side‐products (Table 1, entries 3–6). While some solvents (e.g. dichloromethane, THF) outperformed methanol in terms of selectivity, the overall yield of the major isomer was considerably lower, which was also observed when no water was added along with TCICA (Table 1, entry 7). Finally, the effect of the halogen source was studied indicating a less fruitful outcome for reactions with brominating agents such as *N*‐bromosuccinimide (NBS; Table 1, entry 8). On the other hand, *N*‐chlorosuccinimide (NCS) and TCICA performed equally well (Table 1, entry 9). However, TCICA is considerably cheaper and bears three active chlorine atoms, which were all transferred during the reaction making it an atom efficient oxidizing agent and thus our preferred choice.^[17]^

**Scheme 2:**
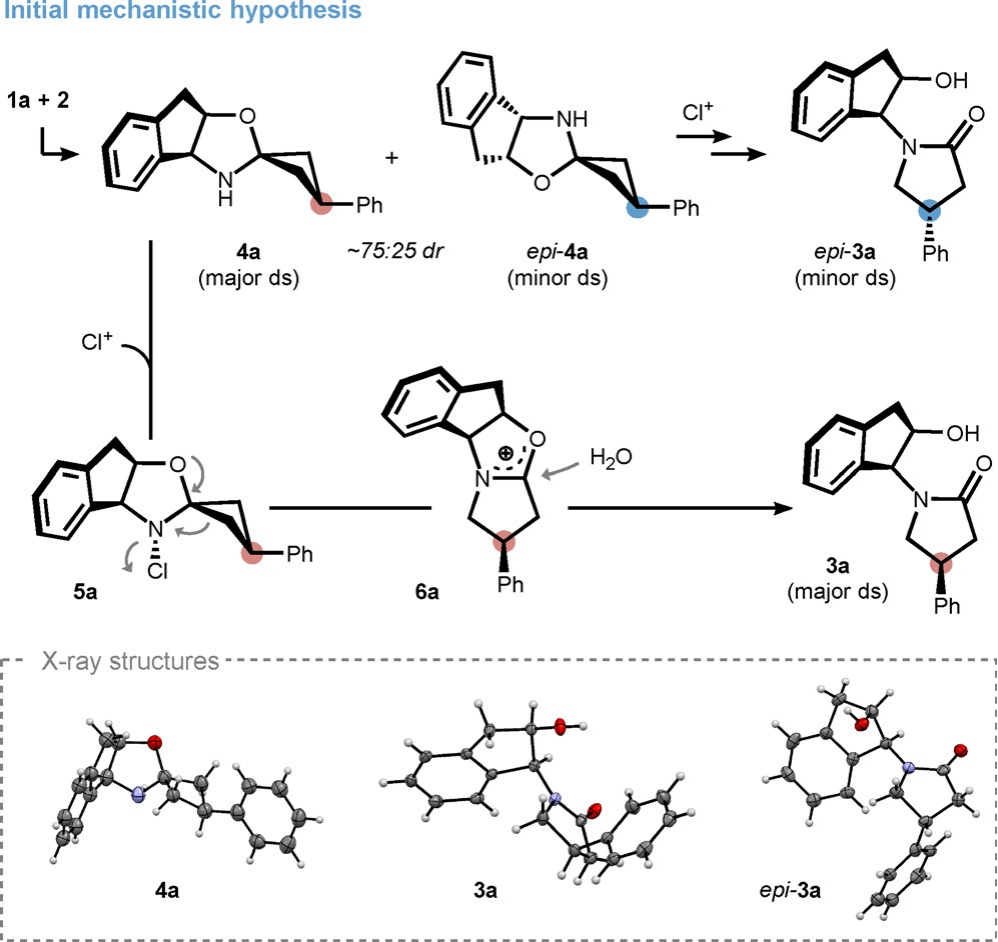
Initial hypothesis via a stereospecific reaction trajectory. ds=diastereomer. CCDC 2051973 (mixture of **4a**/*epi* **‐4a**, only structure **4a** shown), 2051970 (**3a**), and 2051971 (*epi*‐**3a**).

To better understand the reaction outcome, we continued elucidating the details of the stereochemical induction process by spectroscopic and computational means. Pre‐mixing of cyclobutanone **1 a** and indanol **2** was essential and lower yields for premature addition of TCICA were observed indicating a condensation prior to rearrangement. Indeed, a mixture of N,O‐ketals **4 a** and *epi*‐**4 a** was formed in an approximately 75:25 dr when equimolar amounts of the respective starting materials were gently heated (Scheme 2). Assignment of the relative configuration of the major diastereomer **4 a** was possible by NOE correlations revealing an *anti*‐relationship between the NH and phenyl groups. Further structural information was collected by X‐ray diffraction, which indicated a puckered cyclobutane placing the phenyl group in a pseudo equatorial position (see SI for further information). In our initial mechanistic hypothesis, we expected a stereospecific reaction sequence commencing with the chlorination of ketal **4 a** from its sterically accessible side to furnish chloroamine **5 a**. Due to stereoelectronic considerations (σ_C−C_→σ^*^_N−Cl_), the subsequent rearrangement was believed to occur via migration of the anti C−C bond to provide oxocarbenium **6 a** as indicated by the gray arrows. Based on a previous study on hydrolysis of related cations,^[18]^ we concluded that lactam **3 a** must thus be formed as the major diastereomer, which was unambiguously confirmed by X‐ray analysis.

**Scheme 3:**
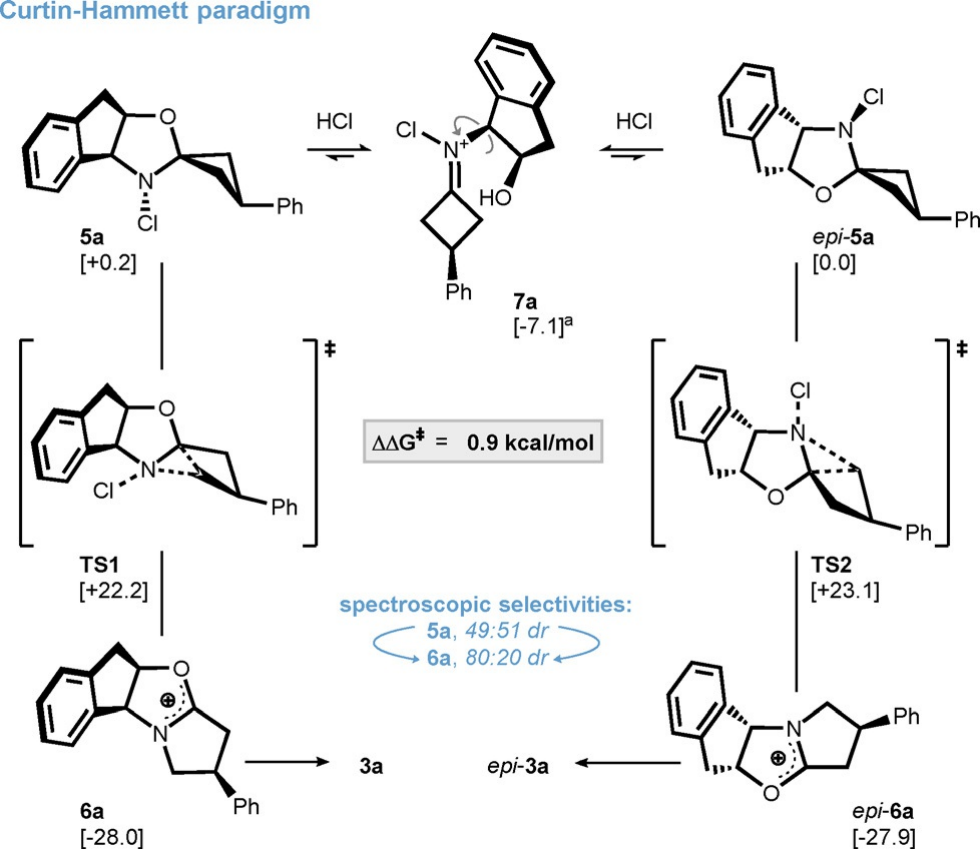
Explanation for a downstream induction based on the Curtin–Hammett principle with calculated relative energies in brackets (Δ*G*_298_(THF) in kcal mol^−1^). Energies were obtained by using the following method: PW6B95‐D3//TPSS‐D3/def2‐TZVP+COSMO‐RS. [a] Epimer (*epi*‐**7 a**) is not shown for clarity.

Interestingly, we were able to monitor the formation of intermediates **5 a**/*epi*‐**5 a** and **6 a**/*epi*‐**6 a** by NMR spectroscopy in *d_4_*‐methanol. Assignment of the corresponding carbenium ions is based on similarities to literature precedent^[19]^ as well as computed ^13^C NMR shifts (see SI for further information), which correlate well with our spectroscopic data. In contrast to the proposed reaction sequence in Scheme 2, the spectroscopically observed selectivity was different for every pair of intermediates indicating a more complex mechanism. An induction of selectivity from 49:51 dr for chloroamines **5 a**/*epi* **‐5 a** to 80:20 dr for oxocarbenium **6 a**/*epi*‐**6 a** was particularly intriguing (Scheme 3). This enhancement can be rationalized under the premise of the Curtin–Hammett principle,^[20]^ which was evaluated by calculating the transition state energies using the def2‐TZVP basis set. While inversion at the nitrogen stereocenter prior to rearrangement led to energetically less favorable trajectories (not shown, see SI for further information), it was found that interconversion of chloroamines **5 a** and *epi*‐**5 a** can occur via an iminium species such as **7 a**. This process depends on protonation of the oxygen atom prior to ring opening and is presumably initiated by HCl that is formed over the course of the reaction. The 49:51 dr between chloroamines **5 a** and *epi*‐**5 a** can thus be explained by their relative stabilities, which differ by only 0.2 kcal mol^−1^. Furthermore, the calculated energetic difference between the stereodetermining transition states **TS1** and **TS2** was found to be 0.9 kcal mol^−1^, which translates well to the downstream selectivity of 80:20 dr for the oxocarbenium and lactam species.

**Scheme 4:**
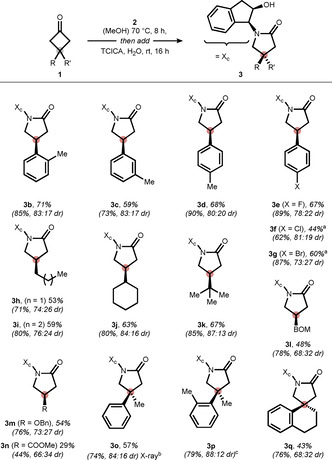
Nitrogen‐based ring expansion of cyclobutanones to access chiral γ‐lactams. Yield of the isolated major diastereomer. Combined yield and diastereoselectivity (in parentheses) was established by NMR analysis of the crude reaction mixture using mesitylene as an internal standard. [a] 70 °C instead of rt. [b] CCDC 2051972 (**3o**). [c] Diastereomers were not separable. BOM=benzyloxymethyl.

Based on the aforementioned downstream stereoinduction, we became interested in exploring the scope of the reaction (Scheme 4). First, we evaluated the steric influence of *ortho*, *meta*, and *para*‐methyl groups at the phenyl ring. As for the parent compound, the diastereomers were all separable and diasteromerically pure γ‐lactams **3 b**, **3 c**, and **3 d** were accessible. Next, we examined halogens that are interesting as bioisosteres (fluorine) and as a handle for cross‐coupling reactions (chlorine and bromine). Although for the *para*‐chloro and *para*‐bromo cyclobutanone higher temperature was required to ensure full conversion, we were pleased to isolate lactams **3 e**, **3 f**, and **3 g** in good yields. Besides aryl, alkyl substitution was well tolerated revealing a bump in selectivity (up to 87:13 dr) when going from primary (**3 h** and **3 i**) to secondary (**3 j**) and tertiary scaffolds (**3 k**). In addition, functional groups such as ether (**3 l** and **3 m**) and ester (**3 n**) were compatible with the oxidative reaction medium, albeit with lower selectivity. Finally, lactams **3 o**, **3 p**, and spirolactam **3 q** containing quaternary all‐carbon stereocenters were constructed. Substrate **3 o** further underlines the downstream stereoinduction as its N,O‐ketal was formed in 59:41 dr improving to 84:16 dr for the γ‐lactam (absolute configuration was established through X‐ray analysis, see SI for further information). The good compatibility with fully substituted stereocenters not only highlights the complementary nature of the presented approach (previous procedures are focused on the synthesis of β‐monosubstituted γ‐lactams)^[21]^ but also elegantly tackles this challenge in asymmetric synthesis.^[22]^

**Scheme 5:**
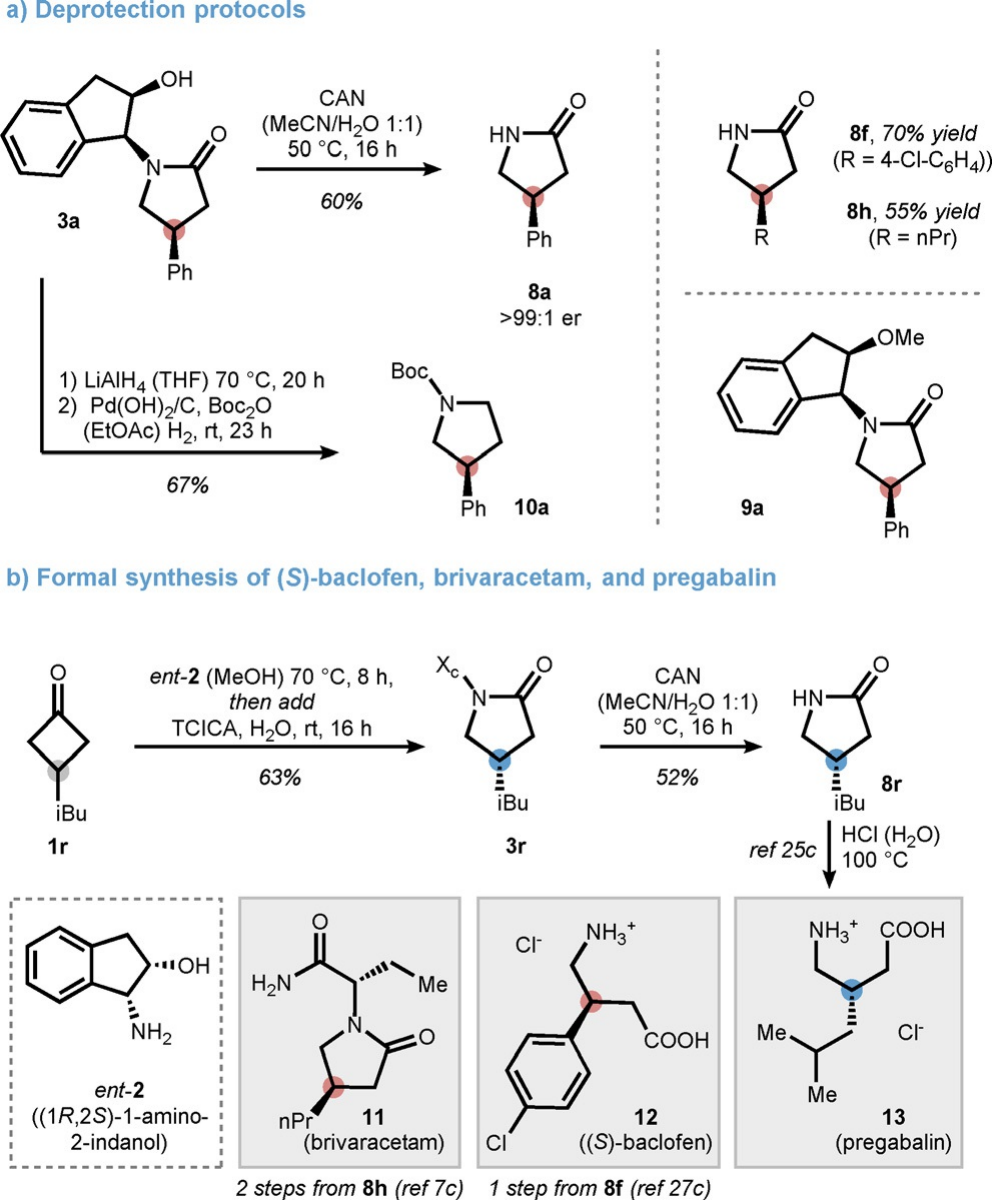
Deprotection protocols and application of the method.

To ensure a synthetically useful reaction, establishing a practical protocol for the removal of the indanyl unit was inevitable (Scheme 5, top). Although it is known that benzyl‐protected lactams can be liberated under Birch conditions, we evaluated more practical possibilities first.^[23]^ While hydrogenolysis, even under harsh conditions, was not successful, we were surprised to find cerium ammonium nitrate (CAN) as a viable mediator to provide **8 a** in 60 % yield and >99 % enantiomeric excess (see SI for further information). Interestingly, the methylated counterpart **9 a** did not undergo deprotection (<10 % yield) emphasizing the crucial role of the hydroxy group for successful cleavage. Similarly, lactams **8 f** and **8 h**, key intermediates for the synthesis of drug molecules (see below), were obtained in 70 % and 55 % yield, respectively. A complementary deprotection was also found in a sequence of reduction and protecting‐group swap yielding chiral pyrrolidine **10 a** in 67 % yield over the two steps.^[24]^

Finally, pregabalin (Lyrica^®^) was formally synthesized from cyclobutanone **1 r** (Scheme 5, bottom).^[25]^ To match the absolute configuration of pregabalin, we used (1*R*,2*S*)‐1‐amino‐2‐indanol (*ent*‐**2**) for asymmetric induction, which is also widely available (81.70 €/25 g for **2** and 128.40 €/25 g for *ent*‐**2**).^[26]^ After successful rearrangement (via **3 r**) and deprotection, lactam **8 r** was isolated and can be hydrolyzed by HCl to release the active drug component. Furthermore, baclofen (Lioresal^®^), which is used to treat muscle spasticity, can be accessed in its enantiopure form (*S*)‐baclofen (**12**).^[27]^ This is worth mentioning because baclofen is given as the racemate even though it is known that the two isomers have different activity.^[27a]^ In analogy, brivaracetam (**11**, Briviact^®^),^[7]^ which was released in 2016 to treat partial‐onset seizures, was formally accessed from key intermediate **8 h**.

## Conclusion

Overall, this study enables the asymmetric nitrogen‐based ring expansions of prochiral cyclobutanones to a variety of γ‐lactams. While the cyclobutanone's strain energy was crucial for this unusual rearrangement to occur, the key for selectivity was found in the widely available 2‐amino‐1‐indanol. An induction downstream the reaction trajectory was identified, which led to good selectivity even when quaternary all‐carbon stereocenters were forged. Moreover, this method does not require potentially toxic transition metals making it ideally suited for the synthesis of drug molecules. Along this line, baclofen, brivaracetam, and pregabalin were formally synthesized to highlight the expected impact on future applications in γ‐lactam syntheses.

## Conflict of interest

The authors declare no conflict of interest.

## Supporting information

As a service to our authors and readers, this journal provides supporting information supplied by the authors. Such materials are peer reviewed and may be re‐organized for online delivery, but are not copy‐edited or typeset. Technical support issues arising from supporting information (other than missing files) should be addressed to the authors.

Supplementary
