# Supplementary material for: Desymmetrization of Prochiral Cyclobutanones via Nitrogen Insertion: A Concise Route to Chiral γ‐Lactams
Source: Angew Chem Int Ed Engl. 2021 Mar 10;60(17):9719–23. doi: 10.1002/anie.202100642 (PMC8252468; doi:10.1002/anie.202100642)
Supplement: Supplementary file 1 — Supplementary [file ANIE-60-9719-s001.pdf]

Angewandte Chemie
Eine Zeitschrift der Gesellschaft Deutscher Chemiker

Supporting Information

# Desymmetrization of Prochiral Cyclobutanones via Nitrogen Insertion: A Concise Route to Chiral γ-Lactams

*Jan Sietmann+, Mike Ong+, Christian Mück-Lichtenfeld, Constantin G. Daniliuc, and Johannes M. Wiest**

anie_202100642_sm_miscellaneous_information.pdf

**Supporting Information**

## Contents

1. General ........................................................................................................ 1
2. Synthesis of starting materials ................................................................ 3
3. Optimization of the asymmetric ring expansion..................................... 12
4. Mechanistic Anaylsis................................................................................ 13
5. Computational Studies ............................................................................. 20
6. Substrate scope ........................................................................................ 27
7. Deprotection of hydroxyindanyl γ-lactams.............................................. 42
8. Crystallographic data ................................................................................ 47
9. References................................................................................................. 50
10. NMR spectra, HPLC traces, and cartesian coordinates.................................. 52

## 1. General

***Analytical methods:*** $^{1}H$ NMR and $^{13}C$ NMR spectra were recorded by the analytical department of the Organisch-Chemisches Institut at the Westfälische Wilhelms-Universität using an Avance II 400 (Bruker), DD2 500 (Agilent), and DD2 600 (Agilent) spectrometer at 26 °C (unless otherwise noted). Chemical shifts are reported in ppm with the solvent resonance as the internal standard ($^{1}H$ NMR $CHCl_3$: $\delta$ = 7.26 ppm, $C_6HD_5$: $\delta$ = 7.16 ppm, $(CHD_2)(CD_3)SO$ = 2.50 ppm; $^{13}C$ NMR $CDCl_3$: $\delta$ = 77.16 ppm, $C_6D_6$ $\delta$ = 128.06 ppm, $(CD_3)_2SO$ = 39.5 ppm, ). The data is reported as follows: chemical shift, multiplicity (s = singlet, d = doublet, t = triplet, q = quartet, p = pentet, br = broad, m = multiplet or combinations of these), coupling constants (Hz) and integration. Apparent multiplicity, which occurs as a result of accidental equality of coupling constants to magnetically non-equivalent protons, is marked as *app.* Infrared (IR) spectra were obtained on a Perkin-Elmer 100 FT-IR spectrometer and are reported in wavenumbers ($cm^{-1}$). Bands are characterized as broad (br), strong (s), medium (m), and weak (w). Melting points were measured on a Büchi B-545 melting-point apparatus. High Resolution Mass Spectrometry (HRMS) was performed by the analytical department of the Organisch-Chemisches Institut at the Westfälische Wilhelms-Universität using a Bruker Daltonics MicroTof or on a Thermo-Fisher Scientific Orbitrap LTQ XL signals are reported as mass to charge ratio *m/z*. GC-MS data was acquired using an Agilent 7890A Gas Chromatograph and an Agilent 5975 or 5975 VL MSD Inert Mass Selective Detector (EI) and is reported as *m/z* (relative intensity). Optical rotations were measured on a JASCO P2000 polarimeter at 589 nm wavelength (sodium D-line) using a standard 10 cm cell (1 mL). Specific rotations, $[\alpha]_D^{20}$, are reported in degree mL/(g·dm) at the specific temperature. Concentrations (*c*) are given in grams per 100 mL of the specific solvent.

***Reaction set-up:*** Unless otherwise noted, all reactions have been carried out with distilled and degassed solvents under an atmosphere of dry Ar in oven- (125 °C) and flame-dried glassware with standard *Schlenk* techniques. All work-up and purification procedures were carried out with pre-distilled technical grade solvents. Standard column chromatography techniques using 40-63 µm silica gel (VWR chemicals) were used for purification and glass silica gel plates 60 F254 (Merck) for thin layer chromatography (TLC).

***X-Ray diffraction:*** Data sets for compounds **[3a]**, **[*epi*-3a]**, **[3o]** and **[4a]** were collected with a Bruker D8 Venture PHOTON III diffractometer. Programs used: data collection: APEX3 V2016.1-0[1] (Bruker AXS Inc., **2016**); cell refinement: SAINT V8.37A (Bruker AXS Inc., **2015**); data reduction: SAINT V8.37A (Bruker AXS Inc., **2015**); absorption correction, SADABS V2014/7 (Bruker AXS Inc., **2014**); structure solution *SHELXT-2015*[2] (Sheldrick, G. M. *Acta Cryst.,* **2015**, *A71*, 3-8); structure refinement *SHELXL-2015*[3] (Sheldrick, G. M. *Acta Cryst.,* **2015**, *C71* (1), 3-8) and graphics, *XP*[4] (Version 5.1, Bruker AXS Inc., Madison, Wisconsin, USA, **1998**). *R*-values are given for observed reflections, and $wR^2$ values are given for all reflections.

*Exceptions and special features*: For compound **[4a]** the four membered ring and the corresponding phenyl substituent were found disordered over two positions in the asymmetric unit. Several restraints (SADI, SAME, ISOR and SIMU) were used in order to improve refinement stability.

***DFT Calculations:*** All geometry optimizations and energy evaluations were performed with the TURBOMOLE 7.4.1[5] program. The structures were optimized without any geometry constraints using the TPSS meta-GGA functional[6] and an atom-pairwise dispersion correction (D3).[7] A flexible triple zeta basis set (def2-TZVP)[8] was used in all calculations. For the calculation of free energy contributions of translation, rotations

and harmonic vibrations ($G^{RRHO}_{298}$), a rotor approximation was applied for vibrational modes with wave numbers below 100 $cm^{-1}$.[9] Single point calculations were performed with the hybrid functional PW6B95(-D3).[10] Free energies of solvation ($G^{solv}_{298}$ ) were obtained with the COSMO-RS model[11] for 298 K using THF as solvent.Theoretical NMR chemical shifts were determined with the B3LYP hybrid functional[12] and the def2-TZVP basis set using Turbomole.

## 2. Synthesis of starting materials

### General procedure A for the preparation of olefins via Wittig-reaction

The olefins were prepared following a literature protocol by Chemler and Liwosz.[13] In an oven dried Schlenk flask methyl-triphenylphosphoniumbromid (3.0 equiv.) was suspended in THF (0.2 M) at 0°C. Potassium *tert*-butoxide (3.0 equiv.) was added slowly to the suspension. The mixture was stirred for 30 min at 0°C. The corresponding ketone (1.0 equiv.) was added and the reaction mixture was allowed to warm to room temperature within 16 h. Water (100 mL) was added afterwards and the mixture was extracted with $Et_2O$ (6 × 50 mL). The combined organic layers were dried over $MgSO_4$, filtered and concentrated under reduced pressure. The desired product was obtained by flash column chromatography with the conditions given in the corresponding entry.

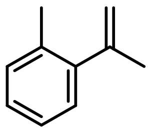

**1-Methyl-2-(prop-1-en-2-yl)benzene [S1]:**
Following the general procedure **A** using 2-methylacetophenone (2.62 mL, 2.69 g, 20 mmol, 1.0 equiv.) the target product **[S1]** (2.02 g, 15 mmol, 75%) was obtained by flash column chromatography ($SiO_2$, pentane, stain with $KMnO_4$) as a colorless oil. Spectroscopic data was in agreement with that previously reported.[13]
**$^1$H NMR (400 MHz, $CDCl_3$)**: δ = 7.21 − 7.09 (m, 4H), 5.21 − 5.17 (m, 1H), 4.86 − 4.83 (m, 1H), 2.32 (s, 3H), 2.07 − 2.02 (m, 3H). **$^{13}$C NMR (100 MHz, $CDCl_3$)**: δ = 146.0, 144.0, 134.6, 130.2, 128.0, 126.9, 125.7, 114.8, 24.5, 19.9.

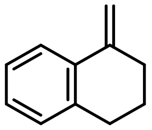

**1-Methylene-1,2,3,4-tetrahydronaphthalene [S2]:**
Following the general procedure **A** using 1-tetralone (5.32 mL, 5.85 g, 40 mmol, 1.0 equiv.) the target product **[S2]** (4.99 g, 35 mmol, 88%) was obtained by flash column chromatography ($SiO_2$, pentane, stain with $KMnO_4$) as a colorless oil. Spectroscopic data was in agreement with that previously reported.[14]
**$^1$H NMR (400 MHz, $C_6D_6$)**: δ = 7.67 − 7.58 (m, 1H), 7.09 − 6.98 (m, 2H), 6.95 − 6.89 (m, 1H), 5.48 (*app* d, $J$ ≈ 1.3 Hz, 1H), 4.90 (*app* q, $J$ ≈ 1.5 Hz, 1H), 2.55 (t, $J$ = 6.3 Hz, 2H), 2.39 – 2.31 (m, 2H), 1.67 − 1.56 (m, 2H). **$^{13}$C NMR (100 MHz, $C_6D_6$)**: δ =143.8, 137.5, 135.1, 129.5, 127.9, 126.3, 124.7, 108.0, 33.6, 30.7, 24.1.

### General procedure B and C for the synthesis of cyclobutanones

### Method B: [2+2] cycloaddition of ketene iminium salt

The prochiral cyclobutanones were prepared following a modified literature protocol by Haufe *et al.*[15] A Schlenk tube was charged with dimethylacetamide (1.2 equiv.) in 1,2-

dichloroethane (0.5 M). The reaction solution was kept at room temperature with a water bath. $Tf_2O$ (2.0 equiv.) was added dropwise and the reaction mixture was stirred at room temperature for 10 min. A solution of the corresponding alkene (1.0 equiv.) and 2,6-lutidine (2.0 equiv.) in 1,2-dichloroethane (2.0 M) was added dropwise to the reaction mixture, which was stirred at 90 °C for 8 h. The reaction was allowed to cool to room temperature and water (20 mL) was added. The reaction mixture was stirred at 90 °C for 16 h. The mixture was allowed to cool to room temperature and the layers were separated. The aqueous layer was extracted with $CH_2Cl_2$ (4 × 50 mL). The combined organic layers were dried over $MgSO_4$, filtered and the solvent was removed under reduced pressure. The product was separated by flash column chromatography with the conditions given in the corresponding entry.

**Method C: [2+2] cycloaddition of dichloroketene**

The prochiral cyclobutanones were prepared following a modified literature protocol by Kočovský *et al.*[16] A Schlenk flask was charged with Zn dust (6.0 equiv.) and freshly distilled $Et_2O$ (0.14 M). The corresponding alkene (1.0 equiv.) was added to the suspension. The suspension was kept at room temperature with a water bath while a solution of trichloroacetyl chloride (2.5 equiv.) and $POCl_3$ (1.1 equiv.) in freshly distilled $Et_2O$ (0.5 M) was added. The water bath was removed and the suspension was stirred at 40 °C for 8 h. After complete conversion, the suspension was allowed to cool down to room temperature and filtered through a pad of Celite® and washed with $CH_2Cl_2$. The solvents were removed under reduced pressure and $CH_2Cl_2$ (100 mL) was added. The organic layer was washed with water (3 × 100 mL) and with an aqueous saturated $NaHCO_3$ solution (3 × 100 mL). The organic layer was dried over $MgSO_4$, filtered and the solvent was removed under reduced pressure. The crude reaction mixture was used without further purification.

To a round flask the crude reaction mixture of the first reaction step and glacial acetic acid (40 mL) were added. The solution was kept at 20 °C with a water bath and Zn dust (4.0 equiv.) was slowly added. The suspension was heated to 80 °C and stirred for 16 h. The mixture was allowed to cool down to room temperature, filtered through a pad of Celite® and washed with $CH_2Cl_2$. The solvent was removed under reduced pressure. The residue was redissolved in $CH_2Cl_2$ (50 mL) and the organic layer was washed with an aqueous saturated $NaHCO_3$ solution (3 × 50 mL) and water (3 × 50 mL). The organic layer was dried over $MgSO_4$, filtered and the solvent was removed under reduced pressure. The product was obtained by flash column chromatography with the conditions given in the corresponding entry.

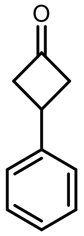


**3-Phenylcyclobutan-1-one [1a]:**
Following the general procedure **B** using styrene (5.73 mL, 5.21 g, 50 mmol, 1.0 equiv.). The desired product **[1a]** (5.29 g, 36 mmol, 72%) was obtained by gradient flash column chromatography ($SiO_2$, pentane : $Et_2O$ (19:1 to 9:1), stained with $KMnO_4$) as a colorless oil. Spectroscopic data was in agreement with that previously reported.[17]
**$^1$H NMR (400 MHz, $CDCl_3$)**: $\delta$ = 7.40 − 7.34 (m, 2H), 7.34 − 7.24 (m, 3H), 3.74 − 3.63 (m, 1H), 3.57 − 3.45 (m, 2H), 3.32 − 3.21 (m, 2H). **$^{13}$C NMR (101 MHz, $CDCl_3$)**: $\delta$ = 206.9, 143.7, 128.8, 126.7, 126.6, 54.8, 28.5.

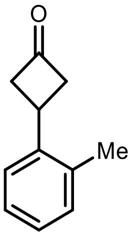


**3-(2'-Methylphenyl)cyclobutan-1-one [1b]:**
Following the general procedure **B** using 2-methylstyrene (2.18 mL, 2.00 g, 17 mmol, 1.0 equiv.) the desired product **[1b]** (1.87 g, 11.7 mmol, 71%) was obtained by flash column chromatography ($SiO_2$, pentane : EtOAc (20:1), stained with $KMnO_4$) as a clear oil. Spectroscopic data was in agreement with that previously reported.[18]
**$^1$H NMR (400 MHz, $CDCl_3$)**: $\delta$ = 7.25 – 7.08 (m, 4H), 3.69 (*app.* p, $J \approx$ 8.3 Hz, 1H), 3.44 – 3.27 (m, 2H), 3.26 – 3.09 (m, 2H), 2.25 (s, 3H), **$^{13}$C NMR (101 MHz, $CDCl_3$)**: $\delta$ = 206.8, 140.8, 136.5, 130.6, 126.9, 126.4, 124.6, 53.1, 26.2, 20.1.

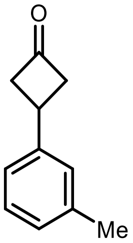


**3-(3'-Methylphenyl)cyclobutan-1-one [1c]:**
Following the general procedure **B** using 3-methylstyrene (2.22 mL, 2.00 g, 17 mmol, 1.0 equiv.) the desired product **[1c]** (1.93 g, 12 mmol, 71%) was obtained by flash column chromatography ($SiO_2$, pentane : EtOAc (20:1), stained with $KMnO_4$) as a clear oil. Spectroscopic data was in agreement with that previously reported.[18]
**$^1$H NMR (400 MHz, $CDCl_3$)**: $\delta$ = 7.44 – 7.35 (m, 1H), 7.30 – 7.18 (m, 3H), 3.86 – 3.73 (m, 1H), 3.68 – 3.57 (m, 2H), 3.44 – 3.34 (m, 2H), 2.51 (s, 3H), **$^{13}$C NMR (101 MHz, $CDCl_3$)**: $\delta$ = 207.1, 143.7, 138.5, 128.7, 127.5, 127.4, 123.6, 54.8, 28.5, 21.6.

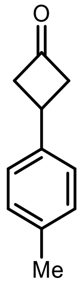


**3-(4'-Methylphenyl)cyclobutan-1-one [1d]:**
Following the general procedure **B** using 4-methylstyrene (2.73 mL, 2.50 g, 21 mmol, 1.0 equiv.) the desired product **[1d]** (2.03 g, 13 mmol, 62%) was obtained by flash column chromatography ($SiO_2$, pentane : EtOAc (20:1), stained with $KMnO_4$) as a clear oil. Spectroscopic data was in agreement with that previously reported.[17]
**$^1$H NMR (400 MHz, $CDCl_3$)**: $\delta$ = 7.24 – 7.15 (m, 4H), 3.73 – 3.59 (m, 1H), 3.54 – 3.41 (m, 2H), 3.30 – 3.15 (m, 2H), 2.36 (s, 3H), **$^{13}$C NMR (101 MHz, $CDCl_3$)**: $\delta$ = 207.2, 140.7, 136.4, 129.5, 126.5, 54.9, 28.2, 21.1.

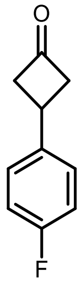


**3-(4'-Fluorophenyl)cyclobutan-1-one [1e]:**
Following the general procedure **B** using 4-fluorostyrene (1.17 mL, 1.20 g, 9.8 mmol, 1.0 equiv.) the desired product **[1e]** (1.20 g, 7.3 mmol, 75%) was obtained by flash column chromatography ($SiO_2$, pentane : EtOAc (20:1), stained with $KMnO_4$) as a clear oil. Spectroscopic data was in agreement with that previously reported.[16]
**$^1$H NMR (400 MHz, $CDCl_3$)**: $\delta$ = 7.34 – 7.23 (m, 2H), 7.15 – 6.98 (m, 2H), 3.84 – 3.61 (m, 1H), 3.60 – 3.45 (m, 2H), 3.33 – 3.06 (m, 2H).**$^{13}$C NMR (101 MHz, $CDCl_3$)**: $\delta$ = 206.4, 161.7 (d, $J$ = 245.1 Hz), 139.4 (d, $J$ = 3.2 Hz), 128.1 (d, $J$ = 8.0 Hz), 115.6 (d, $J$ = 21.4 Hz), 55.0, 28.0.

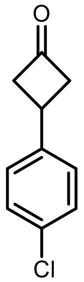


**3-(4'-Chlorophenyl)cyclobutan-1-one [1f]:**

Following the general procedure **B** using 4-chlorostyrene (2.30 mL, 2.50 g, 18 mmol, 1.0 equiv.) the desired product **[1f]** (1.87 g, 10 mmol, 56%) was obtained by flash column chromatography ($SiO_2$, pentane : EtOAc (20:1), stained with $KMnO_4$) as a clear oil. Spectroscopic data was in agreement with that previously reported.[19]
**$^1$H NMR (400 MHz, $CDCl_3$)**: $\delta$ = 7.35 – 7.28 (m, 2H), 7.26 – 7.20 (m, 2H), 3.72 – 3.59 (m, 1H), 3.56 – 3.44 (m, 2H), 3.26 – 3.14 (m, 2H), **$^{13}$C NMR (101 MHz, $CDCl_3$)**: $\delta$ = 206.1, 142.1, 132.6, 128.9, 128.0, 54.9, 28.1.

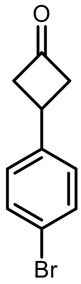


**3-(4'-Bromophenyl)cyclobutan-1-one [1g]:**
Following the general procedure **B** using 4-bromostyrene (1.43 mL, 2.00 g, 11 mmol, 1.0 equiv.) the desired product **[1g]** (1.33 g, 5.9 mmol, 54%) was obtained by flash column chromatography ($SiO_2$, pentane : EtOAc (20:1), stained with $KMnO_4$) as a brown solid. Spectroscopic data was in agreement with that previously reported.[17]
**$^1$H NMR (400 MHz, $CDCl_3$)**: $\delta$ = 7.54 – 7.32 (m, 2H), 7.21 – 7.15 (m, 2H), 3.71 – 3.59 (m, 1H), 3.55 – 3.41 (m, 2H), 3.31 – 3.12 (m, 2H), **$^{13}$C NMR (101 MHz, $CDCl_3$)**: $\delta$ = 206.0, 142.7, 131.9, 128.4, 120.6, 54.8, 28.2.

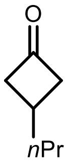


**3-Propylcyclobutan-1-one [1h]:**
Following the procedure of K. L. Erickson[20], in an oven-dried Schlenk tube a suspension of Zn dust (1.31 g, 20 mmol, 2.0 equiv.) and 1-pentene (0.45 mL, 0.29 g, 1.0 mmol, 1.0 equiv.) in freshly distilled $Et_2O$ (25 mL) was placed in an ultrasonic water bath maintained at 10-15 °C. While sonicating the reaction mixture, a solution of trichloroacetyl chloride (2.73 g, 15 mmol, 1.5 equiv.) in 10 mL of freshly distilled $Et_2O$ was added dropwise over 50 min and the sonication was continued for further 30 min. The reaction mixture was filtered through cotton using $Et_2O$ as eluent. $H_2O$ (50 ml) was added, the layers were separated and the aqueous layer was extracted with $Et_2O$ (3 x 50 mL). The combined organic layers were washed with $H_2O$ (100 mL), an aqueous saturated $NaHCO_3$ solution (100 mL) and an aqueous saturated NaCl solution (100 mL). The organic layer was dried over $MgSO_4$, filtered and the solvent was removed under reduced pressure. The crude reaction mixture was used without further purification.
For dehalogenation the second part of general procedure **C** was performed. The desired product **[1h]** (717 g, 6.4 mmol, 64%) was obtained as a clear oil without chromatographic purification.

**IR (neat)**: $\tilde{\nu}$ = 2959 (m), 2928 (m), 2874 (w), 1713 (m), 1465 (w), 1380 (w), 1202 (m), 1168 (w), 1110 (w), 1020 (w), **$^1$H NMR (500 MHz, $CDCl_3$)**: $\delta$ = 3.18 – 3.08 (m, 2H), 2.72 – 2.58 (m, 2H), 2.42 – 2.31 (m, 1H), 1.59 – 1.52 (m, 2H), 1.40 – 1.30 (m, 2H), 0.94 (t, $J$ = 7.3 Hz, 3H), **$^{13}$C NMR (126 MHz, $CDCl_3$)**: $\delta$ = 208.9, 52.7, 38.6, 23.8, 21.5, 14.0, **HRMS (EI)**: Calculated for $C_6H_{12}O$ $[M]^+$: 112.0883, Found: 112.0883.

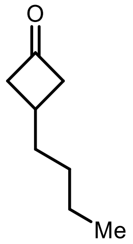


**3-Butylcyclobutanone [1i]:**
Following the general procedure **C** using 1-hexene (1.24 mL, 843 mg, 10 mmol, 1.0 equiv.) the desired product **[1i]** (471 mg, 3.7 mmol, 37%) was obtained by flash column chromatography ($SiO_2$, pentane/$Et_2O$ 19:1, stained with $KMnO_4$) as a colorless oil. Spectroscopic data was in agreement with that previously reported.[21]
**$^1$H NMR (400 MHz, $CDCl_3$)**: $\delta$ = 3.22 − 3.07 (m, 2H), 2.72 − 2.60 (m, 2H), 2.42 − 2.28 (m, 1H), 1.58 (*app.* q, $J \approx$ 7.6 Hz, 2H), 1.41 − 1.24 (m, 4H), 0.92 (t, $J$ = 6.9 Hz, 3H). **$^{13}$C NMR (125 MHz, $CDCl_3$)**: $\delta$ = 209.0, 52.7, 36.2, 30.6, 24.0, 22.6, 14.2.

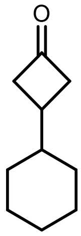


**3-Cyclohexylcyclobutanone [1j]:**
Following the general procedure **C** with minor alterations[a] using vinylcyclohexane (1.37 mL, 1.10 g, 10 mmol, 1.0 equiv.) the desired product **[1j]** (600 mg, 3.9 mmol, 39%) was obtained by flash column chromatography ($SiO_2$, pentane : $Et_2O$ (19:1), stained with $KMnO_4$) as a colorless oil. Spectroscopic data was in agreement with that previously reported.[16]
**$^1$H NMR (400 MHz, $CDCl_3$)**: $\delta$ = 3.09 − 2.98 (m, 2H), 2.80 − 2.69 (m, 2H), 2.13 − 1.98 (m, 1H), 1.82 − 1.71 (m, 4H), 1.71 − 1.63 (m, 1H), 1.34 − 1.10 (m, 4H), 1.00 − 0.86 (m, 2H). **$^{13}$C NMR (125 MHz, $CDCl_3$)**: $\delta$ = 208.6, 50.9, 43.9, 31.0, 30.1, 26.3, 26.2.

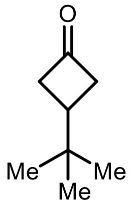


[a] Reduction was performed with Zn dust (5.23 g, 80 mmol, 8.0 equiv.).

**3-(*tert*-Butyl)cyclobutanone [1k]:**
Following the general procedure **C** with minor alterations[b] using 3,3-dimethylbutene (2.57 mL, 1.68 g, 20 mmol, 1.0 equiv.) the desired product **[1k]** (284 mg, 2.3 mmol, 12%) was obtained by flash column chromatography ($SiO_2$, pentane : $Et_2O$ (19:1), stained with $KMnO_4$) as a colorless oil. Spectroscopic data was in agreement with that previously reported.[22]
**$^1$H NMR (400 MHz, $CDCl_3$)**: $\delta$ = 2.96 − 2.79 (m, 4H), 2.36 − 2.24 (m, 1H), 0.94 (s, 9H).
**$^{13}$C NMR (101 MHz, $CDCl_3$)**: $\delta$ = 208.4, 47.9, 34.9, 31.6, 26.6.

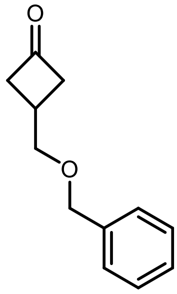


**3-Benzyloxymethyl-cyclobutanone [1l]:**
Following the general procedure **C** with minor alterations[a] using allyloxymethylbenzene (1.48 g, 10 mmol, 1.0 equiv.) the desired product **[1l]** (390 mg, 2.1 mmol, 21%) was obtained by flash column chromatography ($SiO_2$, pentane : $Et_2O$ (19:1), stained with $KMnO_4$) as a colorless oil. Spectroscopic data was in agreement with that previously reported.[23]
**$^1$H NMR (400 MHz, $CDCl_3$)**: $\delta$ = 7.40 − 7.27 (m, 5H), 4.56 (s, 2H), 3.60 (d, *J* = 6.4 Hz, 2H), 3.21 − 3.05 (m, 2H), 2.95 − 2.81 (m, 2H), 2.78 − 2.62 (m, 1H). **$^{13}$C NMR (100 MHz, $CDCl_3$)**: $\delta$ = 207.7, 138.2, 128.6, 127.9, 127.8, 73.4, 73.1, 50.2, 23.8.

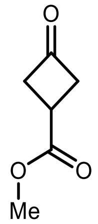


**Methyl 3-oxocyclobutane-1-carboxylate [1n]:**
3-Oxocyclobutane-1-carboxylic acid (456 mg, 4.0 mmol, 1.0 equiv.) and the solid was dissolved in MeOH (20 mL). $H_2SO_4$ (conc., 0.11 mL) was added dropwise and the reaction mixture was heated to 75 °C for 2 h. The organic solvent was removed under reduced pressure and water (50 mL) was added. The mixture was extracted with $CH_2Cl_2$ (3 × 50 mL). The organic layer was washed with an aqueous solution of $NaHCO_3$ (10%, 2 × 50 mL) and with water (2 × 50 mL). The organic layer was dried over $MgSO_4$, filtered and concentrated under reduced pressure. The desired product **[1n]** (399 mg, 3.1 mmol, 78%) was obtained by flash column chromatography ($SiO_2$, pentane : $Et_2O$ (7:3), stained with $KMnO_4$) as a colorless oil. Spectroscopic data was in agreement with that previously reported.[24]
**$^1$H NMR (400 MHz, $CDCl_3$)**: $\delta$ = 3.77 (s, 3H), 3.48 − 3.37 (m, 2H), 3.34 − 3.19 (m, 3H).
**$^{13}$C NMR (101 MHz, $CDCl_3$)**: $\delta$ = 203.8, 174.6, 52.5, 51.8, 27.4.

---

[b] [2+2] Cycloaddition was performed with Zn dust (5.23 g, 80 mmol, 4.0 equiv.).
[a] Reduction was performed with Zn dust (5.23 g, 80 mmol, 8.0 equiv.).

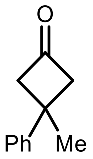


**3-Methyl-3-phenylcyclobutan-1-one [1o]:**
Following the general procedure **C** using isopropenylbenzene (1.23 mL, 1.12 g, 10 mmol, 1.0 equiv.) the desired product **[1o]** (1.15 g, 7.2 mmol, 72%) was obtained by flash column chromatography ($SiO_2$, pentane : EtOAc (15:1), stained with $KMnO_4$) as a clear oil. Spectroscopic data was in agreement with that previously reported.[25]
**$^1$H NMR (400 MHz, $CDCl_3$)**: $\delta$ = 7.43 – 7.28 (m, 4H), 7.31 – 7.22 (m, 1H), 3.53 – 3.42 (m, 2H), 3.18 – 3.07 (m, 2H), 1.61 (s, 3H). **$^{13}$C NMR (101 MHz, $CDCl_3$)**: $\delta$ = 206.8, 148.4, 128.7, 126.4, 125.8, 59.4, 34.1, 31.2.

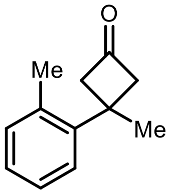


**3-Methyl-3-(*o*-tolyl)cyclobutanone [1p]:**
Following the general procedure **C** with minor alterations[c] using 1-methyl-2-(prop-1-en-2-yl)benzene **[S1]** (1.32 g, 10 mmol, 1.0 equiv.) the target product **[1p]** (281 mg, 1.6 mmol, 16%) was obtained by gradient flash column chromatography ($SiO_2$, pentane : $Et_2O$ (19:1), stain with $KMnO_4$) as a colorless oil. Spectroscopic data was in agreement with that previously reported.[26]
**$^1$H NMR (400 MHz, $CDCl_3$)**: δ =7.24 − 7.14 (m, 4H), 3.59 − 3.44 (m, 2H), 3.18 − 3.05 (m, 2H), 2.34 (s, 3H), 1.56 (s, 3H). **$^{13}$C NMR (100 MHz, $CDCl_3$)**: δ = 207.2, 145.6, 135.3, 131.8, 127.0, 126.7, 126.4, 59.3, 34.8, 28.7, 20.5.

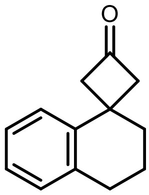


**3',4'-Dihydro-2'H-spiro[cyclobutane-1,1'-naphthalen]-3-one [1q]:**
Following the general procedure **C** with minor alterations[d] using 1-methylene-1,2,3,4-tetrahydronaphthalene **[S2]** (2.15 g, 15 mmol, 1.0 equiv.) the desired product **[1q]** (992 mg, 5.3 mmol, 35%) was obtained by gradient flash column chromatography ($SiO_2$, pentane : $Et_2O$ (19:1 to 9:1), stained with $KMnO_4$) as a yellow oil.
**IR (neat)**: $\tilde{\nu}$ = 2924 (br), 1783 (s), 1492 (w), 1452 (w), 1378 (w), 1112 (m), 759 (m), 728 (w). **$^1$H NMR (500 MHz, $CDCl_3$)**: $\delta$ = 7.41 − 7.36 (m, 1H), 7.24 − 7.19 (m, 1H), 7.15 (td, $J$ = 7.4, 1.3 Hz, 1H), 7.12 − 7.08 (m, 1H), 3.43 − 3.35 (m, 2H), 3.11 − 3.03 (m, 2H),

[c] [2+2] Cycloaddition was performed at room temperature.
[d] [2+2] Cycloaddition was performed with Zn dust (3.92 g, 60 mmol, 4.0 equiv.), trichloroacetyl chloride (2.79 mL, 4.55 g, 25 mmol, 1.7 equiv.) at room temperature.

2.87 (t, $J$ = 6.4 Hz, 2H), 2.07 − 2.03 (m, 2H), 1.90 − 1.83 (m, 2H). **$^{13}$C NMR (126 MHz, $CDCl_3$)**: $\delta$ = 208.4, 142.1, 137.0, 129.4, 126.8, 126.4, 125.2, 62.2, 37.6, 32.3, 30.2, 21.1. **HRMS (EI)**: Calculated for $C_{13}H_{14}O$ $[M]^+$: 186.1039, Found: 186.1038.

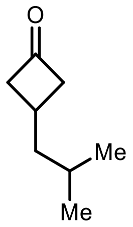


**3-Isobutylcyclobutanone [1r]:**

Following the general procedure **C** with minor alterations[b] using 4-methyl-1-pentene (2.53 mL, 1.68 g, 20 mmol, 1.0 equiv.) the desired product **[1r]** (591 mg, 4.7 mmol, 24%) was obtained by flash column chromatography ($SiO_2$, pentane : $Et_2O$ (19:1), stained with $KMnO_4$) as a colorless oil.

**IR (neat)**: $\tilde{\nu}$ = 2956 (br), 1782 (s), 1469 (m), 1386 (m), 1285 (w), 1197 (m), 1095 (m), 984 (w), 856 (w).**$^1$H NMR (400 MHz, $CDCl_3$)**: $\delta$ = 3.19 − 3.04 (m, 2H), 2.71 − 2.63 (m, 2H), 2.53 – 2.35 (m, 1H), 1.68 − 1.50 (m, 1H), 1.48 (t, $J$ = 7.2 Hz, 2H), 0.92 (d, $J$ = 6.6 Hz, 6H).**$^{13}$C NMR (101 MHz, $CDCl_3$)**: $\delta$ = 208.9, 53.0, 45.8, 27.5, 22.7, 22.2. **HRMS (EI)**: Calculated for $C_8H_{14}O$ $[M]^+$: 126.1039, Found: 126.1040.

[b] [2+2] Cycloaddition was performed with Zn dust (5.23 g, 80 mmol, 4.0 equiv.).

## 3. Optimization of the asymmetric ring expansion

### Identifying of an appropriate amine source

*Unsuccessfull/less successful substrates:*

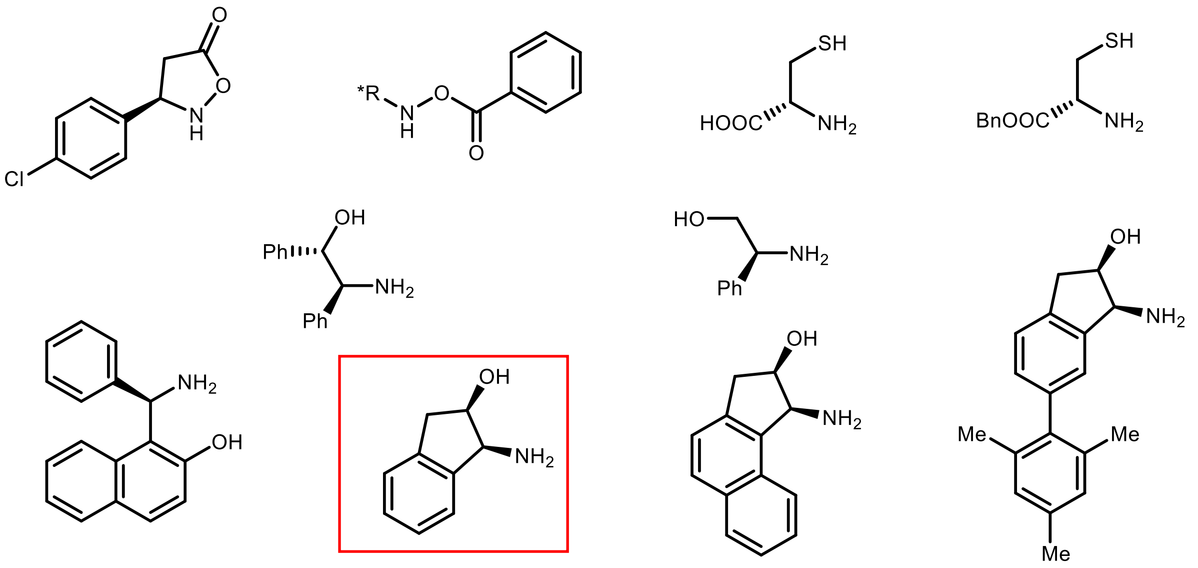


Starting point for optimization

### Optimization Tables

*Table 1: Reaction optimization for the asymmetric ring expansion to γ-lactam* ***[3a]*** *using (1S,2R)-1-amino-2-indanol. Yield was determined by $^{1}H$ NMR using mesitylene as an internal standard.*

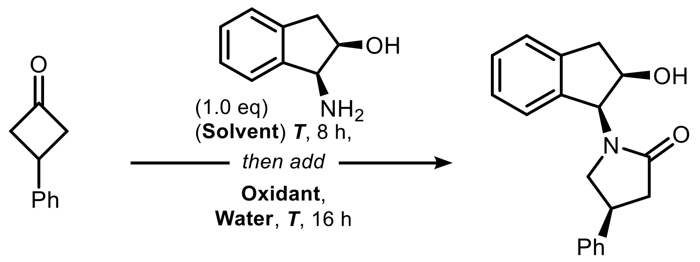


| Oxidant (equiv.) | Water | Solvent | *T* | Further changes | Yield | dr | | Yield major |
|---|---|---|---|---|---|---|---|---|
| TCICA (0.33) | 10 mmol | MeOH | 70 °C, rt | - | 96% | 80 | 20 | 77% |
| TCICA (0.33) | 10 mmol | $CH_2Cl_2$ | 70 °C, rt | - | 68% | 87 | 13 | 59% |
| TCICA (0.33) | 10 mmol | THF | 70 °C, rt | - | 24% | 85 | 15 | 20% |
| TCICA (0.33) | 10 mmol | DMF | 70 °C, rt | - | 50% | 77 | 23 | 39% |
| TCICA (0.33) | 10 mmol | MeOH | 70 °C, 70 °C | - | 73% | 80 | 20 | 58% |
| TCICA (0.33) | 10 mmol | MeOH | 70 °C, -30 °C | - | 48% | 87 | 13 | 42% |
| TCICA (0.33) | - | MeOH | 70 °C, rt | - | 77% | 81 | 19 | 62% |
| TCICA (0.33) | 5 mL | MeOH | 70 °C, rt | - | 84% | 78 | 22 | 66% |
| TCICA (0.33) | - | $CH_2Cl_2$ | 70 °C, rt | | 29% | 88 | 12 | 26% |
| TCICA (0.33) | 5 mL | $CH_2Cl_2$ | 70 °C, rt | | 91% | 73 | 27 | 66% |
| TCICA (0.33) | 10 mmol | MeOH | 70 °C, rt | 1) 1 h | 74% | 80 | 20 | 59% |
| NCS (1.0) | 10 mmol | MeOH | 70 °C, rt | - | 98% | 80 | 20 | 78% |
| NBS (1.0) | 10 mmol | MeOH | 70 °C, rt | - | 55% | 78 | 22 | 43% |

## 4. Mechanistic Anaylsis

### Rate and mechanism of *N,O*-ketal formation

*Premature addition of an oxidizing agent (NCS or TCICA) led to sluggish reaction and significantly lower yields highlighting the important role of the N,O-ketal. The rate of its formation was studied at different temperatures and with acid additives both leading to an acceleration, but acid additives (acetic acid (AcOH) or p-toluenesulfonic acid (TsOH)) led to lower overall yields.*

*The following curves were observed by in situ NMR monitoring:*

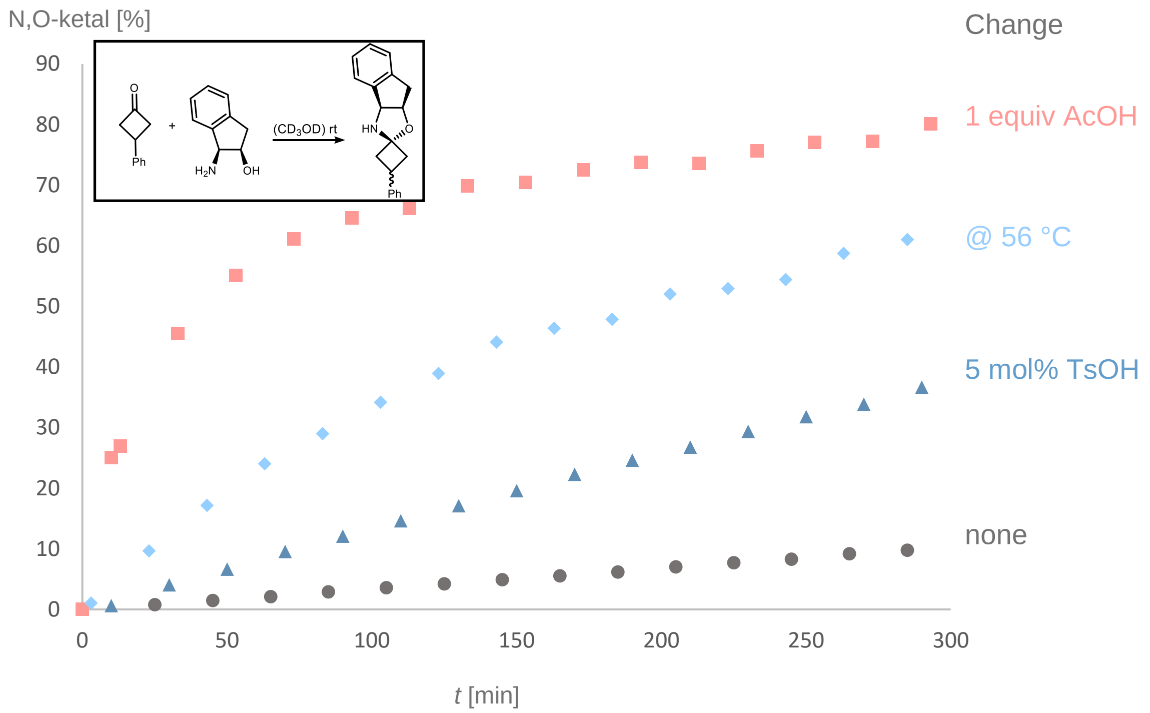


*Figure 1: Rate of N,O-Ketal formation against time in CD₃OD under standard conditions (dot), with addition of TsOH (triangle), at 56 °C (hash), with addition of AcOH (square).*

*The following overall yields were obtained after rearrangement:*

a) With TsOH the reaction mixture was kept at rt for 16 h before TCICA (0.33 equiv) was added, *75% yield*
b) With acetic acid the reaction mixture was kept at rt for 16 h before TCICA (0.33 equiv), *70% yield*

*Interesting was that the alpha protons of the cyclobutanone exchange for D and we obtained the tetradeuterated product* ***[S3]*** *(vide infra) in significant amounts after rearrangement. This indicates the transient formation of an enol/enamine during the N,O-ketal formation.*

*When acetic acid was added from the beginning, the incorporation of deuterium can be increased from an average of 2.48 deuterium (without acetic acid) to 3.66 deuterium*

*(with acetic acid). This highlights the ability of acid additives to catalyze the forward and reverse reaction.*

**[S3]**

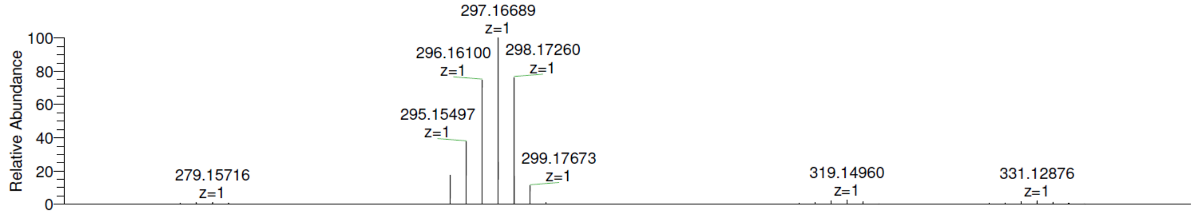


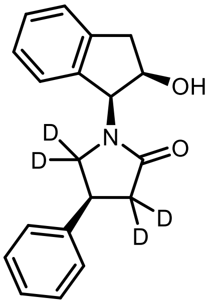


| | |
|---|---|
| Formula: | C19 H20 N O2 |
| Mass (monoisotopic): | 294.15 |
| Difference Value: | 0.000004 |
| Error Sum: | 0.002 |
| Error (%): | 0.036 |

Deuterium: 0-fold (%): 21.36 6.85
Deuterium: 1-fold (%): 42.36 13.59
Deuterium: 2-fold (%): 80.70 25.89
Deuterium: 3-fold (%): 100.00 32.08
Deuterium: 4-fold (%): 67.31 21.59
Label Atom Sum: 2.48 (12.40%)

**[S3]**, *from reaction with 1 equiv of acetic acid*

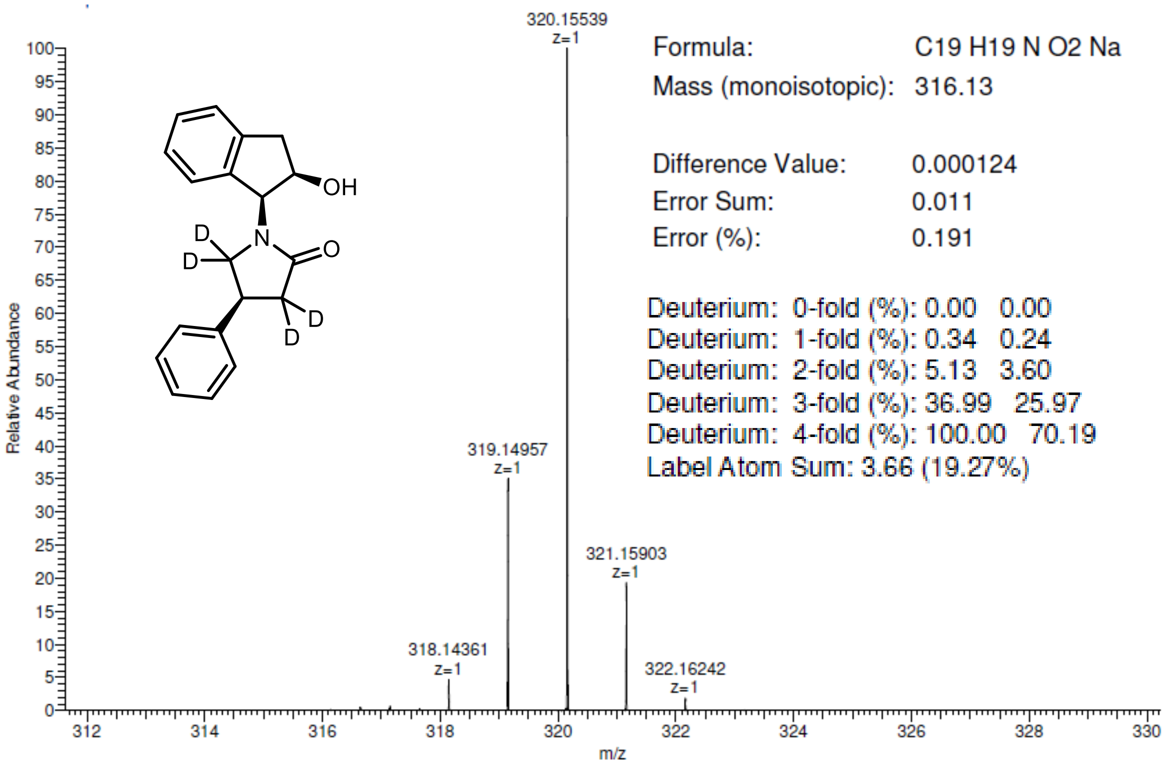

## Synthesis, isolation, and characterization of *N,O*-ketal

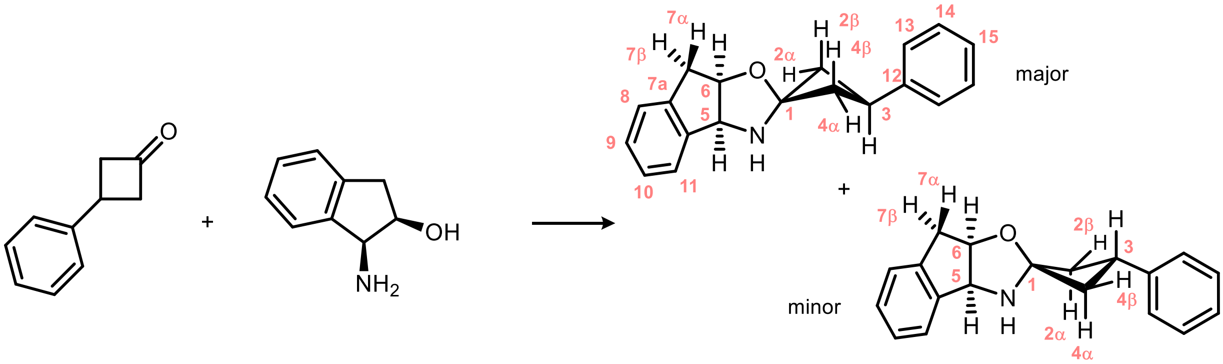


**(1*r*,3*a*'*S*,8*a*'*R*)-3-phenyl-3',3*a*',8',8*a*'-tetrahydrospiro[cyclobutane-1,2'-indeno[1,2-d]oxazole] [4a, *epi*-4a]:**

3-Phenylcyclobutan-1-one (1.46 g, 10 mmol, 1.0 equiv) and (1*S*,2*R*)-1-amino-2,3-dihydro-1*H*-inden-2-ol (1.49 g, 10 mmol, 1.0 equiv) were dissolved in toluene (25.0 mL). The reaction mixture was stirred at 50 °C for 16 h under an argon atmosphere. Most of the toluene was evaporated under reduced pressure and the crude material was suspended in heptane (~100 mL) and heated to reflux for 1 h. The hot solution was filtered and cooled to -20 °C. After 16 h, the solvent was decanted and the residue was dried under vacuum to give the *N,O*-ketal **[4a, *epi*-4a]** (2.20 g, 7.9 mmol, 79 % yield, 74:26 dr) as a white solid as a mixture of diastereomers.

**Major diastereomer [4a]: $^{1}$H NMR (600 MHz, DMSO-d6)**: $\delta$ = 7.43-7.38 (m, 1H, H-11), 7.26-7.09 (m, 8H, H-8, H-9, H-10, H-12, H-13, H-14), 4.89 (d, $^{3}J$ = 5.7 Hz, 1H, H-5), 4.61 (*app.* t, $^{3}J \approx$ 5.9 Hz, 1H, H-6), 3.71 (br s, 1H, NH), 3.11 (dd, $^{2}J$ = 17.3 Hz, $^{3}J$ = 6.1 Hz, 1H, H-7β), 3.03 (*app.* p, $^{3}J \approx$ 9.2 Hz, 1H, H-3), 2.97 (d, $^{2}J$ = 17.3 Hz, 1H, H-7α), 2.54 (ddd, $^{2}J$ = 11.5 Hz, $^{3}J$ = 8.2 Hz, $^{4}J$ = 5.3 Hz, 1H, H-2α), 2.35 (dd, $^{2}J$ = 11.5 Hz, $^{3}J$ = 9.8 Hz, 1H, H-2β), 1.70 (dd, $^{2}J$ = 11.0 Hz, $^{3}J$ = 9.5 Hz, 1H, H-4β), 1.68-1.62 (m, 1H, H-4α). **$^{13}$C NMR (150 MHz, DMSO-d6)**: $\delta$ = 144.8 (s, C-12), 142.9 (s, C-11a), 141.3 (s, C-7a), 128.1 (d, C-14), 127.7 (d, C-9), 126.6 (d, C-13), 126.3 (d, C-11), 125.7 (d, C-15*), 125.6 (d, C-10*), 124.8 (d, C-8), 93.4 (s, C-1), 79.8 (d, C-6), 67.5 (d, C-5), 45.1 (t, C-4), 42.0 (t, C-2), 39.0 (t, C-7), 30.2 (d, C-3).

**Minor diastereomer [*epi*-4a]**: **$^{1}$H NMR (600 MHz, DMSO-d6)**: $\delta$ = 7.35-7.32 (m, 1H, H-11), 7.27-7.09 (m, 8H, H-8, H-9, H-10, H-12, H-13, H-14), 4.81 (d, $^{2}J$ = 5.6 Hz, 1H, H-5), 4.63 (*app.* t, $^{3}J \approx$ 5.9 Hz, 1H, H-6), 3.71 (br s, 1H, NH), 3.25 (*app.* p, $^{3}J \approx$ 8.7 Hz, 1H, H-3), 3.13 (dd, $^{2}J$ = 17.2 Hz, $^{3}J$ = 5.8 Hz, 1H, 7β), 3.02 (d, $^{2}J$ = 17.2 Hz, 1H, 7α), 2.67 (dddd, $^{2}J$ = 12.3 Hz, $^{3}J$ = 9.4 Hz, $^{4}J$ = 4.0, 0.9 Hz, 1H, H-2β), 2.31 (*app.* ddt, $^{2}J$ = 12.3 Hz, $^{3}J$ = 8.0 Hz, $^{4}J \approx$ 1.3 Hz, 1H, H-2α), 1.89 (dddd, $^{2}J$ = 11.8 Hz, $^{3}J$ = 9.2 Hz, $^{4}J$ = 4.0, 0.9 Hz, 1H, H-4β), 1.68-1.62 (m, 1H, H-4α). **$^{13}$C NMR (150 MHz, DMSO-d6)**: $\delta$ = 145.4 (s, C-12), 143.4 (s, C-11a), 141.3 (s, C-7a), 128.1 (d, C-14), 127.6 (d, C-9), 126.5 (d, C-11*), 126.4 (d, C-13), 125.6 (d, C-10*), 125.5 (d, C-15*), 124.7 (d, C-8), 95.1 (s, C-1), 79.7 (d, C-6), 67.1 (d, C-5), 45.1 (t, C-4), 41.4 (t, C-2), 38.9 (t, C-7), 30.8 (d, C-3). [* = Assignment is interconvertible]. **HRMS (ESI)**: Calculated for $C_{19}H_{20}NO$ $[M+H]^{+}$: 278.1539, Found: 278.1536. Relative stereochemistry was established by 2D-NMR experiments on the diastereomeric mixture. Further structural information was extracted from X-ray diffraction.

**Characterization of downstream intermediates in $CD_3OD$:**

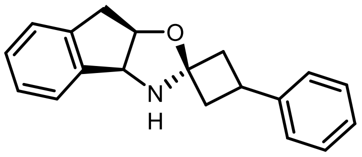

**(3a'S,8a'R)-3-phenyl-3',3a',8',8a'-tetrahydrospiro[cyclobutane-1,2'-indeno[1,2-*d*]oxazole] [4a, *epi*-4a]:**

*Freshly prepared N,O-ketal was dissolved in $CD_3OD$. During 24 h, no exchange of alpha protons, no hydrolysis and no change in diastereoselectivity was observed.*

**Major diastereomer [4a]**: **$^1$H NMR (600 MHz, $CD_3OD$)**: $\delta$ = 7.49-7.45 (m, 1H), 7.26-7.04 (m, 8H), 4.95 (d, $J$ = 5.8 Hz, 1H), 4.74 (*app.* td, $J \approx$ 6.0, 1.3 Hz, 1H), 3.21-3.12 (m, 2H), 3.07 (d, $J$ = 16.8 Hz, 1H), 2.60 (ddd, $J$ = 11.9, 8.3, 5.1 Hz, 1H), 2.46 (dd, $J$ = 11.9, 9.7 Hz, 1H), 1.92-1.76 (m, 2H). **$^{13}$C NMR (150 MHz, $CD_3OD$)**: $\delta$ = 146.0, 142.8, 142.7, 129.4, 129.3, 128.1, 127.4, 127.0, 126.8, 126.2, 94.9, 81.8, 67.0, 45.4, 43.0, 40.1, 31.6.

**Minor diastereomer [ *epi*-4a]**: **$^1$H NMR (600 MHz, $CD_3OD$)**: $\delta$ = 7.43-7.38 (m, 1H), 7.26-7.07 (m, 8H), 4.85 (d, $J$ = 6.1 Hz, 1H), 4.78 (*app.* td, $J \approx$ 5.8, 1.2 Hz, 1H), 3.37 (*app.* p, $J \approx$ 9.0 Hz, 1H), 3.25-3.10 (m, 2H), 2.69 (ddd, $J$ = 12.3, 9.0, 4.7 Hz, 1H), 2.42 (dd, $J$ = 12.3, 9.2 Hz, 1H), 1.98-1.88 (m, 2H). **$^{13}$C NMR (150 MHz, $CD_3OD$)**: $\delta$ = 146.2, 143.3, 142.8, 129.4, 129.3, 128.0, 127.4, 126.9, 126.7, 126.2, 96.5, 81.8, 68.5, 45.3, 42.3, 40.1, 33.2.

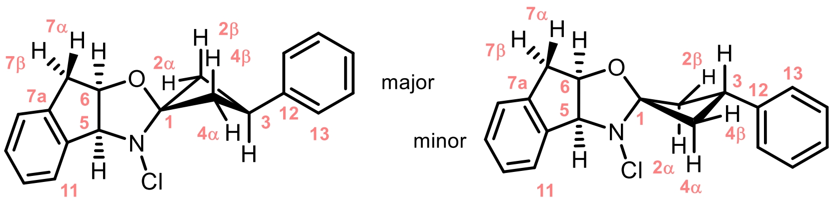


**(3a'S,8a'R)-3'-chloro-3-phenyl-3',3a',8',8a'-tetrahydrospiro[cyclobutane-1,2'-indeno[1,2-*d*]oxazole] [5a, *epi*-5a]:**

*N,O-ketal was dissolved in $C_6D_6$. NCS was added and the reaction mixture stirred at room temperature for 5 minutes. The reaction mixture was filtered through a small plug of silica gel and the crude solution directly subjected to NMR spectroscopic analysis. In benzene the chloroamine diastereomers were stable for ~24 h and could be fully characterized by 2D methods (59:41 dr). However, when the solvent was switched to $CD_3OD$, the chloride was stable for ~10 minutes and rearrangement started right away. Both spectra are attached.*

**Major diastereomer [5a]**: **$^1$H NMR (600 MHz, $C_6D_6$)**: $\delta$ = 7.44-7.39 (m, 1H, H-11), 7.06-6.82 (m, 8H, H-8, H-9, H-10, H-12. H-13, H-14), 5.13 (d, $^3J$ = 5.5 Hz, 1H, H-5), 4.91 (*app.* t, $^3J \approx$ 5.8 Hz, 1H, H-6), 3.16 (ddd, $^2J$ = 13.1 Hz, $^3J$ = 8.8 Hz, $^4J$ = 4.5 Hz, 1H, H-2β), 3.01 (*app.* p, $^3J \approx$ 8.1 Hz, 1H, H-3), 2.98 (d, $^2J$ = 17.6 Hz, 1H, H-7α), 2.71-2.66 (m, 2H, H-2α, H-7β), 1.80-1.72 (m, 2H, H-4). **$^{13}$C NMR (150 MHz, $C_6D_6$)**: $\delta$ = 144.6 (s, C-12), 141.8 (s, C-7a), 140.5 (s, C-11a), 129.2 (d, C-9), 128.6 (d, C-14), 127.4 (d, C-10), 126.9 (d, C-13), 126.6 (d, C-11), 126.3 (d, C-15), 125.4 (d, C-8), 102.6 (s, C-1), 83.6 (d, C-5), 79.7 (d, C-6), 46.5 (t, C-4), 41.2 (t, C-2), 39.7 (t, C-7), 29.9 (d, C-3).

**Minor diastereomer [*epi*-5a]**: **$^1$H NMR (600 MHz, $C_6D_6$)**: $\delta$ = 7.34 (d, $^2J$ = 7.6 Hz, 1H), 7.06-6.82 (m, H-8, H-9, H-10, H-12. H-13, H-14), 5.02 (d, $^3J$ = 5.3 Hz, 1H), 4.96 (*app.* t, $^3J \approx$ 5.5 Hz, 1H), 3.20 (*app.* p, $^3J \approx$ 9.4 Hz, 1H, H-3), 3.04 (d, $^2J$ = 17.5 Hz, 1H, H-7α), 2.86-2.80 (m, 2H, H-2), 2.77-2.70 (m, 1H, H-7β), 1.83 (dd, $^2J$ = 12.3 Hz, $^3J$ = 9.1

Hz, 1H, H-4α), 1.60-1.57 (m, 1H, H-4β). **$^{13}$C NMR (150 MHz, $C_6D_6$)**: *δ* = 144.5 (s, C-12), 142.2 (s, C-7a), 140.2 (s, C-11a), 129.2 (s, C-9), 128.6 (d, C-14), 127.3 (d, C-10), 126.8 (d, C-13), 126.7 (d, C-11), 126.3 (d, C-15), 125.2 (d, C-8), 103.4 (s, C-1), 82.7 (d, C-5), 80.3 (d, C-6), 46.6 (t, C-4), 40.8 (t, C-2), 39.7 (t, C-7), 31.5 (d, C-3).
**Major diastereomer [5a]**: **$^{1}$H NMR (600 MHz, $CD_3OD$)**: *δ* = 7.60-7.05 (m, 9H), 5.43 (d, *J* = 5.5 Hz, 1H), 5.15 (*app.* t, *J* ≈ 5.7 Hz, 1H), 3.30-2.98 (m, 4H), 2.52-2.41 (m, 1H), 1.64-1.58 (m, 2H). **$^{13}$C NMR (150 MHz, $CD_3OD$)**: *δ* = 145.5, 143.0, 141.2, 130.3, 129.4, 128.3, 127.4, 127.3, 127.2, 126.4, 103.2, 84. 5, 80.9, 46.6, 41.7, 40.3, 30.5.
**Minor diastereomer [*epi*-5a]**: **$^{1}$H NMR (600 MHz, $CD_3OD$)**: *δ* = 7.60-7.05 (m, 9H), 5.33 (d, *J* = 5.3 Hz, 1H), 5.19 (*app.* td, *J* ≈ 5.4, 1.1 Hz, 1H), 3.23-3.12 (m, 3H), 2.71-2.59 (m, 2H), 1.65-1.58 (m, 1H), 1.54 (ddd, *J* = 12.7, 8.9, 4.4 Hz, 1H). **$^{13}$C NMR (150 MHz, $CD_3OD$)**: 145.5, 143.3, 140.9, 130.3, 129.4, 128.1, 127.4, 127.4, 127.3, 126.2, 104.0, 83.6, 81.5, 46.8, 41.3, 40.4, 31.9.

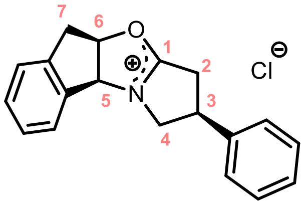


**(2*S*,4a*R*,9b*S*)-2-phenyl-1,2,3,4a,5,9b-hexahydroindeno[1,2-*d*]pyrrolo[2,1-b]oxazol-10-ium chloride [6a]:**
*Due to the low concentration, only the major isomer was characterized. dr ~ 77:23*
**Major diastereomer [6a]**: **$^{1}$H NMR (600 MHz, $CD_3OD$)**: *δ* = 7.58 (dd, *J* = 7.4, 0.7 Hz, 1H, H-11), 7.48-7.20 (m, 8H, H-8, H-9, H-10, H-13, H-14, H-15), 6.71 (*app.* td, *J* ≈ 7.7, 1.8 Hz, 1H, H-6), 5.98 (d, *J* = 7.9 Hz, 1H, H-5), 4.53 (ddd, *J* = 10.9, 8.8, 1.7 Hz, 1H, H-4), 4.48 (*app.* p, *J* ≈ 8.5 Hz, 1H), 3.80 (dd, *J* = 10.9, 7.6 Hz, 1H, H-4), 3.79 (dd, *J* = 18.9, 7.5 Hz, 1H, H-7), 3.67 (dd, *J* = 18.9, 1.8 Hz, 1H, H-7), 3.42 (ddt, *J* = 19.0, 9.3, 1.7 Hz, 1H, H-2), 3.15 (dd, *J* = 19.0, 8.5 Hz, 1H, H-2). **$^{13}$C NMR (150 MHz, $CD_3OD$)**: *δ* = 184.1 (s, C-1), 141.6 (s, Ar-C), 140.0-120.0 (not assignable, 5C, Ar-C), 140.3 (s), 135.5 (d, Ar-C), 128.1 (d, Ar-C), 126.2 (d, Ar-C), 101.8 (d, C-6), 68.8 (d, C-5), 53.5 (t, C-4), 45.5 (d, C-3), 39.7 (t, C-7), 32.8 (t, C-2).

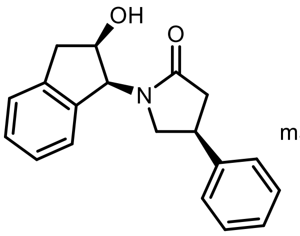

major

minor
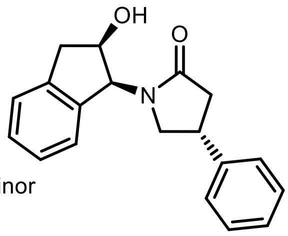


**1-((1*S*,2*R*)-2-hydroxy-2,3-dihydro-1*H*-inden-1-yl)-4-phenylpyrrolidin-2-one [3a, *epi*-3a ]**
*For the synthesis, see chapter 6. Here are the NMR details in d4-MeOH:*
**Major diastereomer [3a]**: **$^{1}$H NMR (600 MHz, $CD_3OD$)**: *δ* = 7.32-7.23 (m, 8H), 7.22-7.17 (m, 1H), 5.56 (d, *J* = 6.5 Hz, 1H), 4.72 (*app.* td, *J* ≈ 6.7, 5.4 Hz, 1H), 3.57 (*app.* t, *J* ≈ 8.7 Hz, 1H), 3.54-3.48 (m, 1H), 3.44 (dd, *J* = 9.1, 7.8 Hz, 1H), 3.20 (dd, *J* = 16.3, 6.9 Hz, 1H), 2.91-2.80 (m, 2H), 2.67 (dd, *J* = 16.6, 9.6 Hz, 1H). **$^{13}$C NMR (150 MHz, $CD_3OD$)**: *δ* = 177.2, 143.3, 142.5, 139.2, 129.8, 129.7, 128.3, 128.1, 127.9, 126.4, 126.2, 73.9, 60.2, 54.0, 41.1, 40.4, 39.6.

**Minor diastereomer [*epi*-3a]**: **$^{1}$H NMR (600 MHz, $CD_3OD$)**: $\delta$ = 7.32-7.12 (m, 8H), 7.10-7.05 (m, 1H), 5.54 (d, $J$ = 6.5 Hz, 1H), 4.70 (*app.* td, $J \approx$ 6.8, 5.3 Hz, 1H), 3.83 (dd, $J$ = 10.0, 7.9 Hz, 1H), 3.60 (*app.* p, $J \approx$ 7.7 Hz, 1H), 3.21 (dd, $J$ = 16.4, 7.0 Hz, 1H), 3.11 (dd, $J$ = 10.0, 5.9 Hz, 1H), 2.95-2.88 (m, 2H), 2.61 (dd, $J$ = 16.8, 6.9 Hz, 1H). **$^{13}$C NMR (150 MHz, $CD_3OD$)**: $\delta$ = 177.4, 144.2, 142.4, 138.9, 129.8, 129.7, 128.1, 127.9, 127.6, 126.4, 126.4, 73.8, 60.1, 54.5, 41.0, 39.9, 38.4.

*Monitoring the reaction by $^{1}$H NMR in $CD_3OD$:*

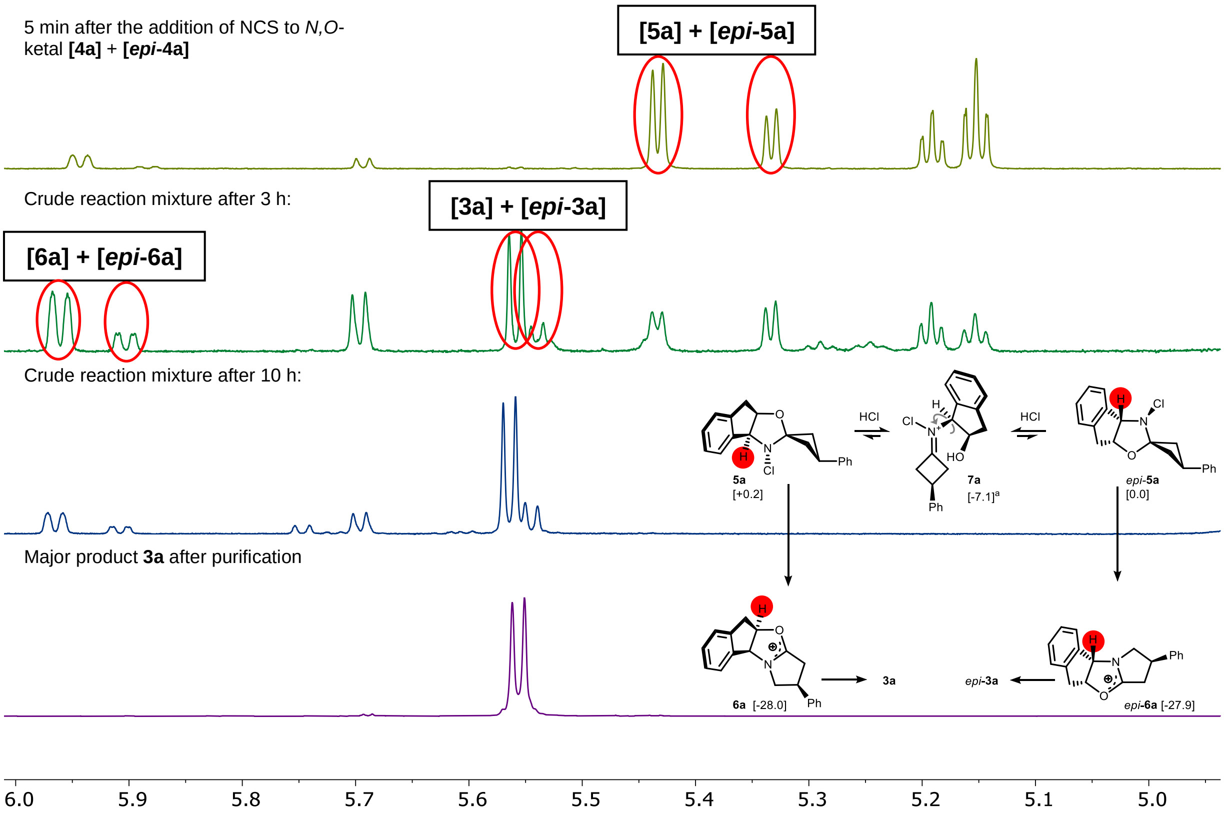


*When the solvent was $CH_2Cl_2$, one of the side products is chlorolactam **[S4]** (vide infra), which does not accumulate in methanol as indicated by the crude reaction spectra above. **[S4]** does also not undergo $S_N2$ substitution to furnish the product, which was checked by subjecting **[S4]** to the standard reaction conditions (1.0 equiv trichloroisocyanuric acid, 1.0 equiv HCl, 10 equiv $H_2O$ in methanol).*

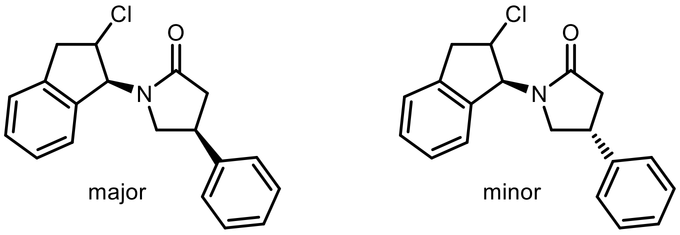


**1-((1*S*)-2-chloro-2,3-dihydro-1*H*-inden-1-yl)-4-phenylpyrrolidin-2-one [S4, *epi*-S4]**

**Major diastereomer [S4]**: **$^{1}$H NMR (600 MHz, $CD_3OD$)**: $\delta$ = 7.35-7.13 (m, 9H), 5.74 (d, $J$ = 7.7 Hz, 1H), 4.57 (*app.* q, $J \approx$ 7.8 Hz, 1H), 3.68-3.61 (m, 1H), 3.54-3.44 (m, 2H), 3.34-3.27 (m, 1H), 3.13 (dd, $J$ = 15.8, 8.1 Hz, 1H), 2.96 (dd, $J$ = 16.9, 8.8 Hz, 1H), 2.67

(dd, $J$ = 16.9, 8.1 Hz, 1H). **$^{13}$C NMR (150 MHz, $CD_3OD$)**: $\delta$ = 177.4, 143.6, 141.1, 138.6, 130.0, 129.9, 128.7, 128.1, 127.8, 126.0, 124.5, 66.0, 59.6, 51.5, 41.5, 40.2, 38.8.
**Minor diastereomer [*epi*-S4]**: **$^{1}$H NMR (600 MHz, $CD_3OD$)**: $\delta$ = 7.35-7.13 (m, 9H), 5.72 (d, $J$ = 7.3 Hz, 1H), 4.63 (*app.* q, $J \approx$ 7.4 Hz, 1H), 3.73-3.60 (m, 2H), 3.56-3.50 (m, 1H), 3.16-3.10 (m, 1H), 3.07 (dd, $J$ = 9.4, 6.0 Hz, 1H), 2.94 (dd, $J$ = 15.5, 6.8 Hz, 1H), 2.71 (dd, $J$ = 17.0, 7.4 Hz, 1H). **$^{13}$C NMR (150 MHz, $CD_3OD$)**: $\delta$ = 177.4, 143.6, 141.2, 138.4, 129.9, 129.8, 128.5, 128.1, 127.8, 126.0, 124.8, 66.3, 60.0, 51.9, 41.7, 39.7, 38.4. *Relative configuration at chloride stereocenter could not be proved.*

## 5. Computational Studies

In our model calculation, we have considered $MeOH_2^+$ as the Bronsted acid that protonates **[5a]** to form the iminium **[7a].** The released chloride ($Cl^-$) was treated as an isolated ion. Since the solvation energy of both ions is calculated with an implicit solvation model (COSMO-RS), we have to assume that relative energies including reactions with these two ions contain a residual error, but are qualitatively correct.

*We considered two Curtin-Hammett scenarios that can explain the observed product resolution from 5a/epi-5a to 6a/epi/6a:*

***Scenario 1:***

*Inversion of N-Cl stereocenter of epi-5a (via TS(epi-5a,Cl-Inv)) to its higher-energy conformer epi-5a-Cl-Inv, which preferably rearranges (via TS5a-6a, syn-N-Cl)*

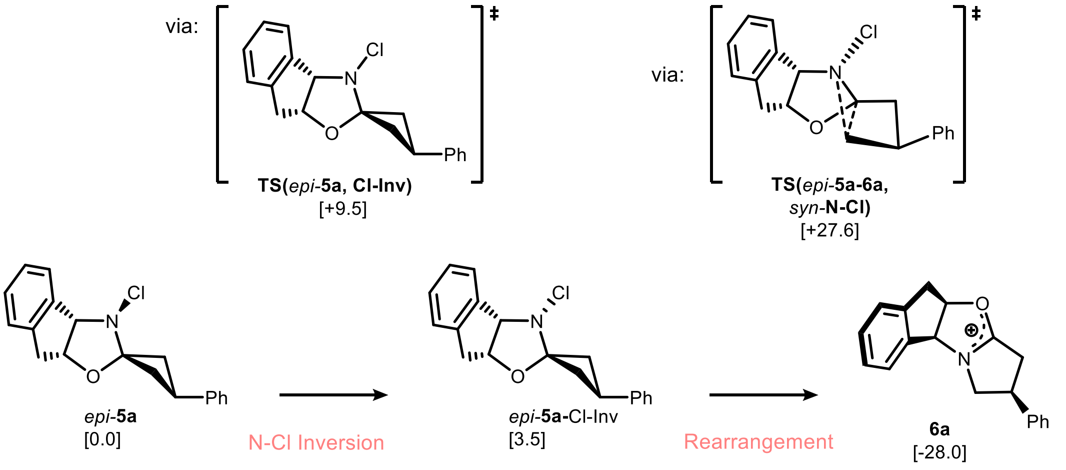


*(shown for only one isomer)*

***Scenario 2:***

*Interconversion of epi-5a and 5a. It was found that HCl was required in this process followed by rearrangement via preferred TS(5a-6a)*

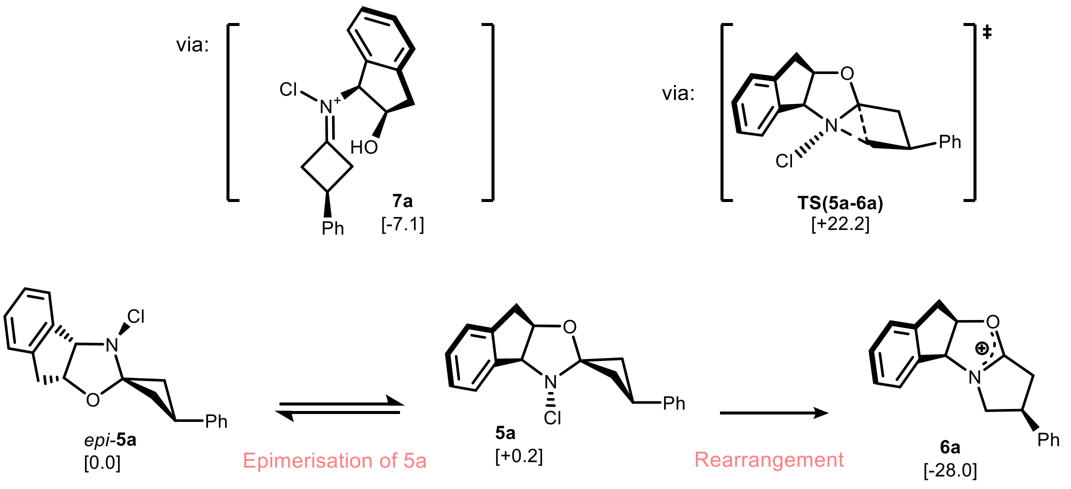

Based on the calculated TS energies and relative stabilities, scenario 2 appears much more likely and was discussed in the paper. The complete picture and respective relative energies are given below:

**Scheme S1:**

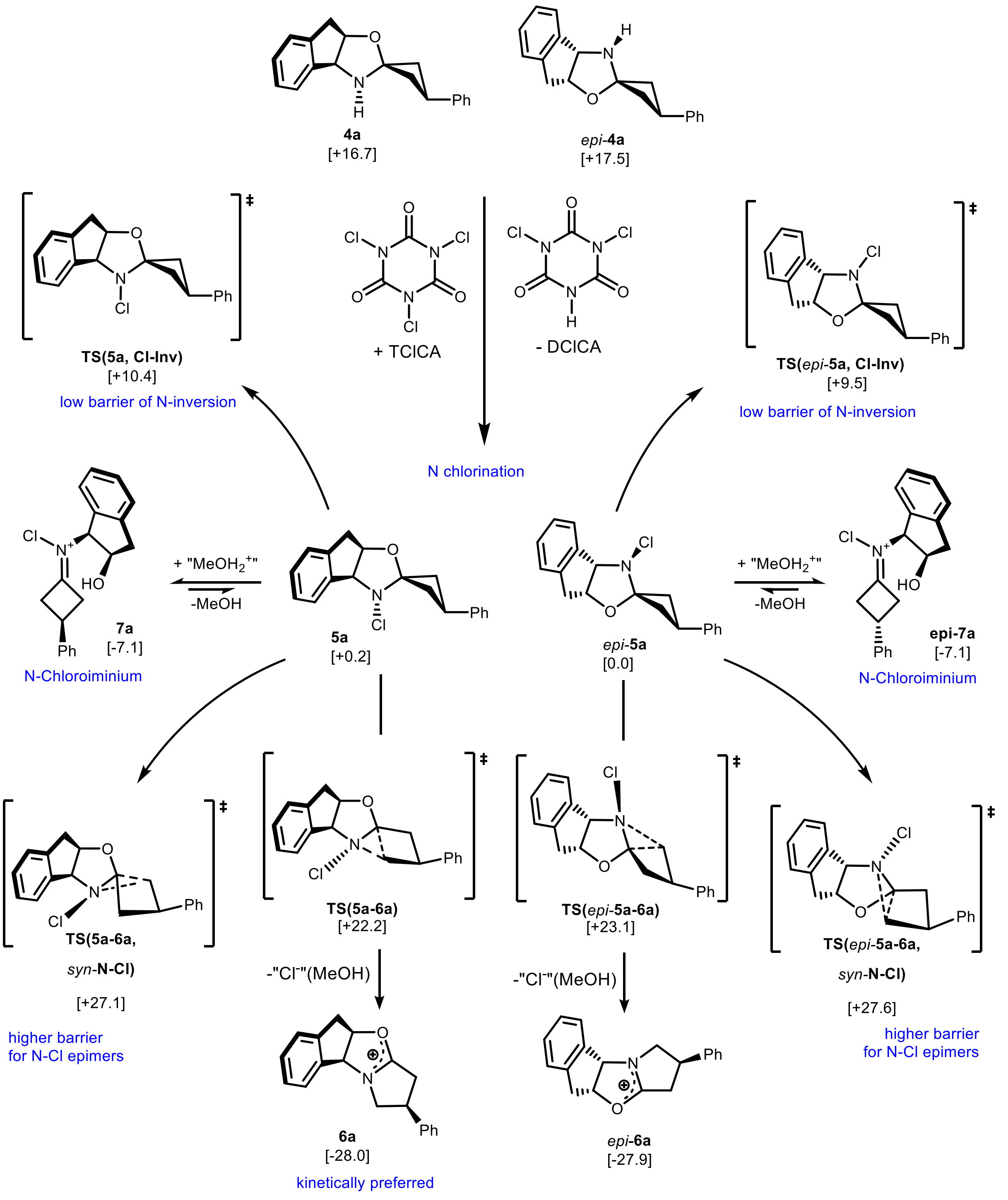

**Table S1.** Relative energies of molecular species in the reaction of 3-phenylcyclobutanone **[1a]**, calculated with DFT[a]. The relative free energy of the intermediates *with respect to isolated* **[*epi*-5a]**, *DClCA and* $MeOH_2^+$ is calculated as $\Delta G(298)_{solv} = \Delta E(PW6B95\text{-}D3//TPSS\text{-}D3/def2\text{-}TZVP) + \Delta G^{RRHO}_{298} + \Delta G^{solv}_{298}$. Molecular structures are displayed in Figure S1.

| Compound | E(TPSS-D3) [Eh] | E(PW6B95-D3) [Eh] | $G^{RRHO}_{298}$ [kcal/mol] | $G^{solv}_{298}$ (THF) [kcal/mol] | **$\Delta G(298)_{solv}$ (THF) [kcal/mol]** |
|---|---|---|---|---|---|
| **[*epi*-4a]** | -865.938391 | -866.874960 | 180.666 | -11.288 | 17.5 |
| **[4a]** | -865.940172 | -866.876726 | 180.885 | -11.218 | 16.7 |
| **[*epi*-5a]** | -1325.544272 | -1326.808467 | 173.067 | -10.925 | 0.0 |
| **[5a]** | -1325.544722 | -1326.808512 | 173.292 | -10.888 | 0.2 |
| **[*epi*-6a]** | -865.151876 | -866.082908 | 175.724 | -44.104 | -27.9[b] |
| **[6a]** | -865.153038 | -866.084180 | 176.076 | -43.760 | -28.0[b] |
| **TS (*epi*-5a-6a)** | -1325.509579 | -1326.760388 | 171.755 | -16.653 | 23.1 |
| **TS (5a-6a)** | -1325.509035 | -1326.758756 | 171.331 | -18.147 | 22.2 |
| **TS (*epi*-5a-6a, syn N-Cl)** | -1325.501969 | -1326.752592 | 171.540 | -16.882 | 27.6 |
| **TS (5a-6a, syn N-Cl)** | -1325.501050 | -1326.750471 | 171.121 | -18.279 | 27.1 |
| **TS (*epi*-5a, Cl-Inv)** | -1325.526105 | -1326.793680 | 172.743 | -10.364 | 9.5 |
| **TS (5a, Cl-Inv)** | -1325.524339 | -1326.791901 | 172.562 | -10.390 | 10.4 |
| **[*epi*-7a]** | -1325.909778 | -1327.172055 | 178.384 | -46.228 | -7.1 |
| **[*epi*-7a] (Conf2)** | -1325.907732 | -1327.169843 | 178.042 | -45.682 | -5.5 |
| **[7a]** | -1325.909420 | -1327.171795 | 178.313 | -46.327 | -7.1 |
| **[7a] (Conf2)** | -1325.909179 | -1327.171665 | 178.606 | -45.721 | -6.1 |
| MeOH | -115.787855 | -115.901212 | 17.310 | -1.607 | |
| $MeOH_2^+$ | -116.089711 | -116.200291 | 25.167 | -72.818 | |
| TClCA | -1885.169984 | -1886.679728 | 5.549 | -6.127 | |
| DClCA | -1425.590918 | -1426.770712 | 13.476 | -8.964 | |
| Chloride | -460.289014 | -460.623501 | 10.860 | -72.240 | |

[a] all calculations were performed with the def2-TZVP basis set
[b] free energy includes $G(298)_{sol}$ of the free chloride anion in THF and should be used with caution

**Figure S1** Optimized molecular structures (TPSS-D3/def2-TZVP) of intermediates and transition structures reported in Table S1. Distances are given in Å, the dihedral angle in the cyclobutane ring ist given in degrees. Relative free energies in kcal/mol in square brackets (as taken from Table S1).

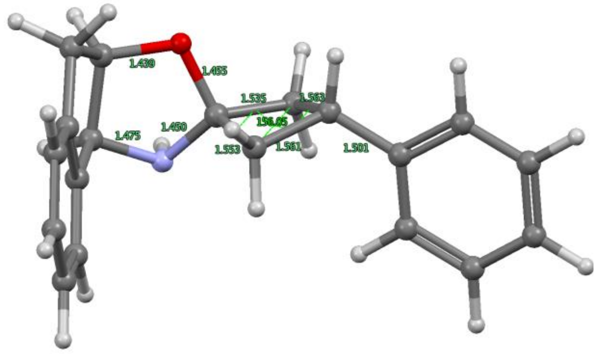

**[*epi*-4a] [+17.5]**

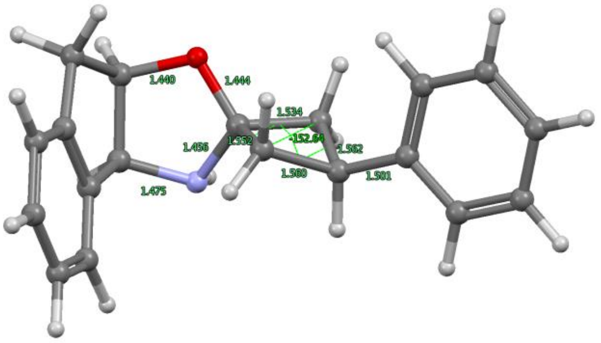

**[4a] [+16.7]**

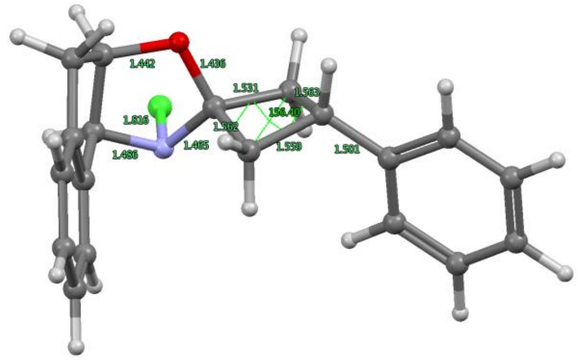

**[*epi*-5a] [0.0]**

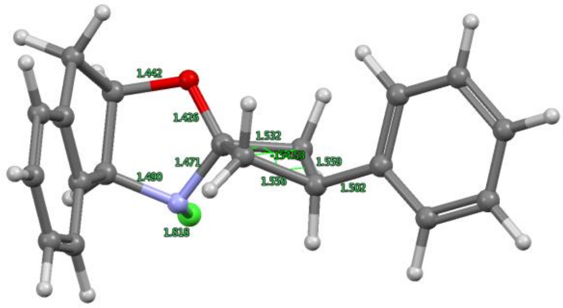

**[5a] [+0.2]**

**Transition structures of N-Cl inversion:**

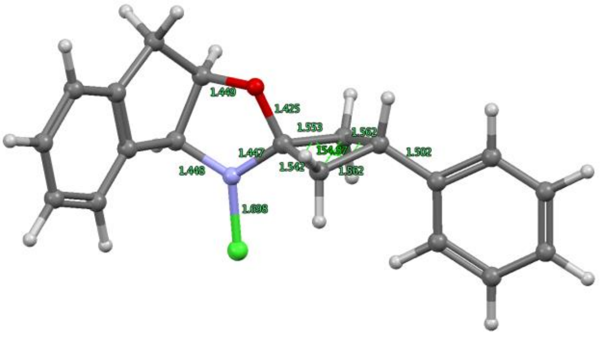

**TS (*epi*-5a, Cl-Inv) [+9.5]**

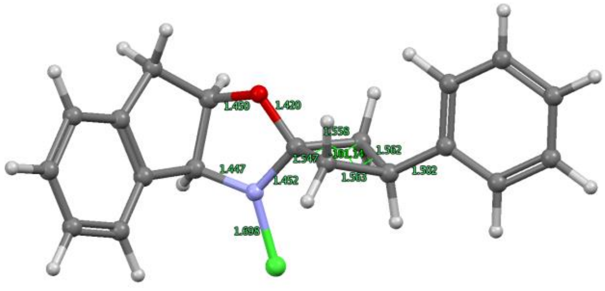

**TS (5a, Cl-Inv) [+10.4]**

**Figure S1** (continued)

**Transition structures of ring expansion**

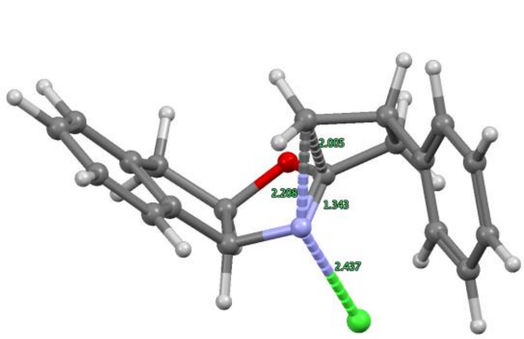

**TS (*epi*-5a-6a) [+23.1]**

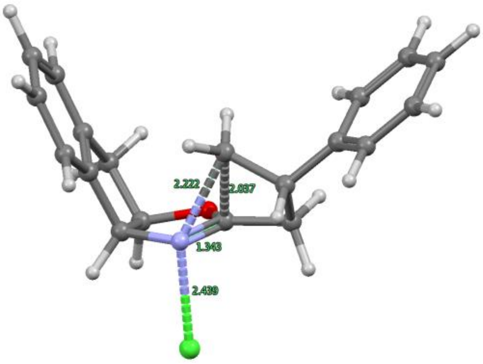

**TS (5a-6a) [+22.2]**

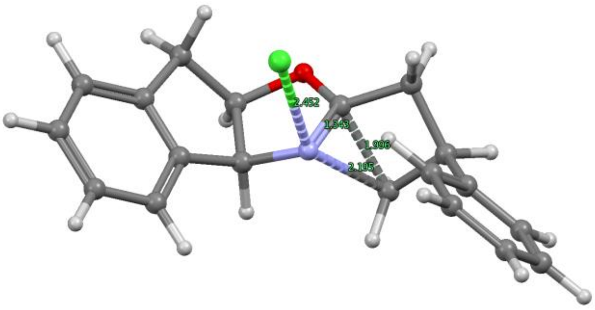

**TS (*epi*-5a-6a, syn N-Cl) [+27.6]**

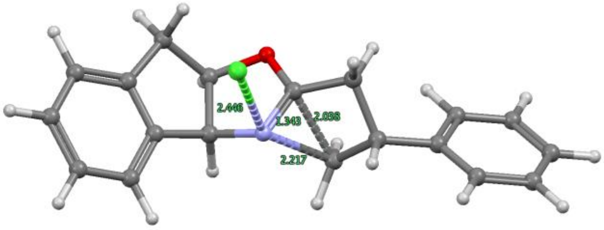

**TS (5a-6a, syn N-Cl) [+27.1]**

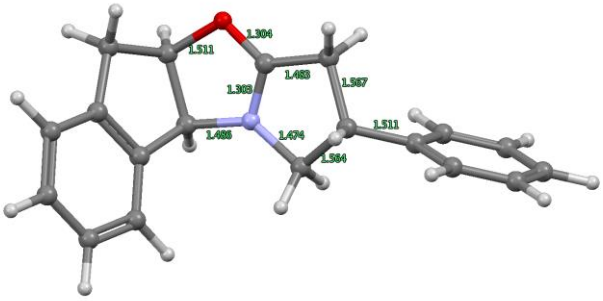

**[*epi*-6a] [-27.9]**

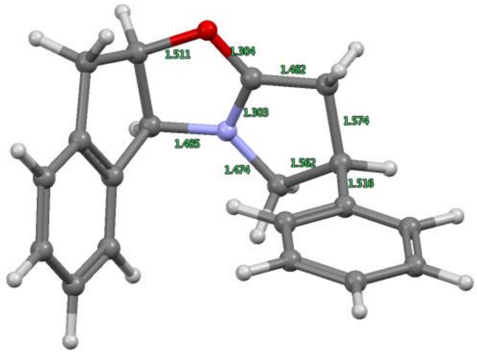

**[6a] [-28.0]**

**Figure S1** (continued)

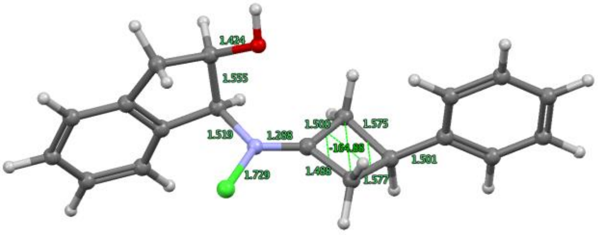

**[7a] (Conformer 1) [-7.1]**

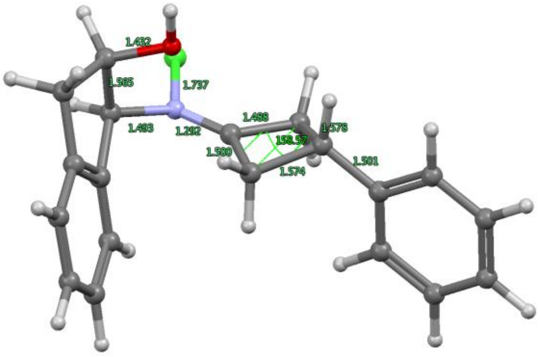

**[7a] (Conformer 2) [-6.1]**

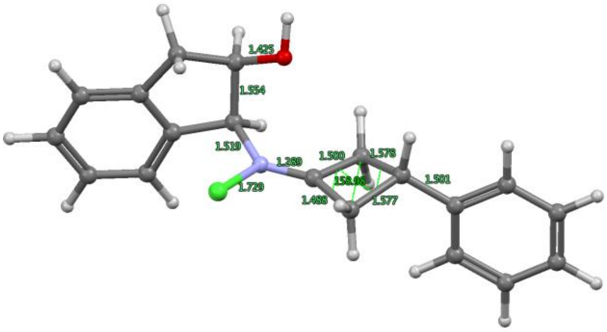

**[*epi*-7a] (Conformer 1) [-7.1]**

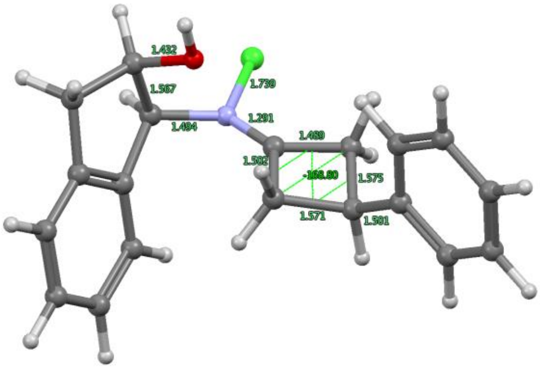

**[*epi*-7a] (Conformer 2) [-5.5]**

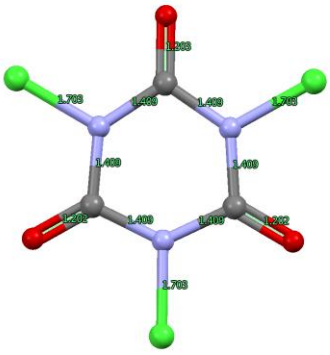

**TClCA**

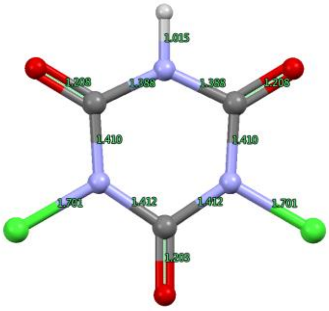

**DClCA**

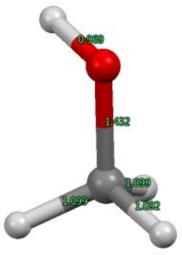

**MeOH**

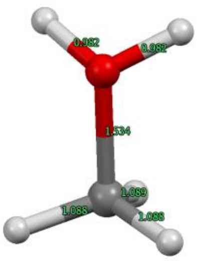

**$MeOH_2^+$**

**Figure S2.** Calculated NMR chemical shifts (B3LYP//TPSS-D3/def2-TZVP) of **6a** and ***epi*-6a**.

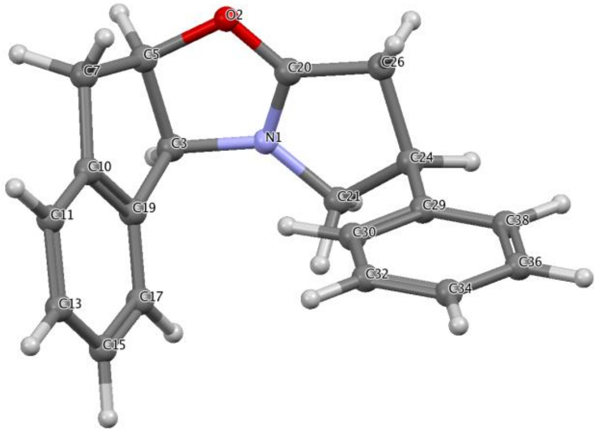

**6a**

| **[6a]** | σ[a] [ppm] | **δ[b] [ppm]** | **exp δ [ppm]** |
|---|---|---|---|
| C20 (= C-1) | -10.82 | **193.78** | **184.1** |
| C26 (= C-2) | 143.82 | **39.15** | **32.8** |
| C24 (= C-3) | 131.86 | **51.11** | **45.5** |
| C21 (= C-4) | 124.58 | **58.38** | **53.5** |
| C3 (= C-5) | 107.92 | **75.05** | **68.8** |
| C5 (= C-6) | 70.72 | **112.25** | **101.8** |
| C7 (= C-7) | 138.83 | **44.14** | **39.7** |

[a] isotropic chemical shielding (B3LYP/def2-TZVP)
[b] calculated chemical shift (Reference: TMS)

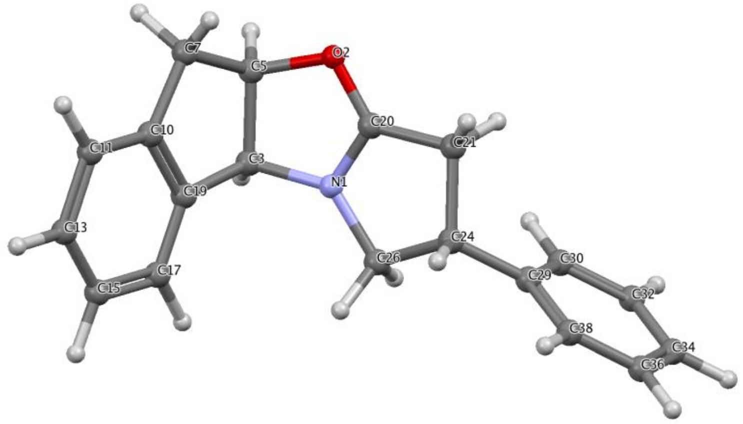

***epi*-6a**

| **[*epi*-6a]** | σ[a] [ppm] | **δ[b] [ppm]** | **exp δ [ppm][c]** |
|---|---|---|---|
| C20 (= C-1) | -9.91 | **192.88** | **184.0** |
| C21 (= C-2) | 144.02 | **38.94** | **33.0** |
| C24 (= C-3) | 129.78 | **53.19** | **45.1** |
| C26 (= C-4) | 123.43 | **59.53** | **53.6** |
| C3 (= C-5) | 107.83 | **75.14** | **67.3** |
| C5 (= C-6) | 71.46 | **111.50** | **101.8** |
| C7 (= C-7) | 139.14 | **43.83** | **38.3** |

[a] isotropic chemical shielding (B3LYP/def2-TZVP)
[b] calculated chemical shift (Reference: TMS)
[c] characteristic peaks assignable from crude mixture

## 6. Substrate scope

### General procedure D for the asymmetric ring-expansion of cyclobutanones to 4-substituted γ-lactams

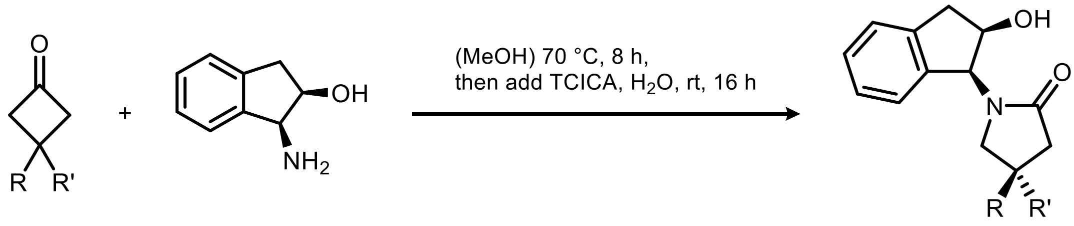


An oven dried Schlenk tube was charged with the corresponding cyclobutanone **[1]** (1.0 mmol, 1.0 equiv.) and (1*S*,2*R*)-1-amino-2,3-dihydro-1*H*-inden-2-ol (149 mg, 1.0 mmol, 1.0 equiv.). MeOH (5 mL, 0.20 M) was added and the mixture was heated to 70 °C and stirred for 8 h. The solution was allowed to cool to room temperature and trichloroisocyanuric acid (TCICA) (77.5 mg, 0.33 mmol, 0.33 equiv.) was added under vigorous stirring. After 5 min, water (0.18 mL, 180 mg, 10 mmol, 10 equiv.) was added and the reaction mixture was stirred at room temperature for 16 h. The crude suspension was added to water (100 mL) and extracted with $CH_2Cl_2$ (5 × 50 mL). The combined organic extracts were dried over $MgSO_4$, filtered, and concentrated under reduced pressure. NMR yield and dr of the crude reaction mixture was determined by $^1$H NMR using 1,3,5-trimethylbenzene (28.0 µL, 24.5 mg, 0.20 mmol) as the internal standard. The product diastereomers were separated by silica gel column chromatography.

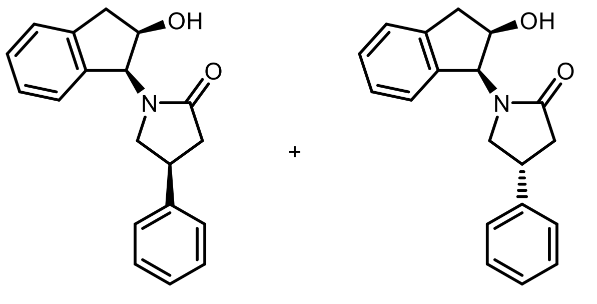


major minor

**1-((1*S*,2*R*)-2-Hydroxy-2,3-dihydro-1*H*-inden-1-yl)-4-phenylpyrrolidin-2-one [3a, *epi*-3a]:**

Following the general procedure **D** using 3-phenylcyclobutan-1-one **[1a]** (146 mg, 1.0 mmol, 1.0 equiv.) the major diastereomer (206 mg, 0.70 mmol, 70%) and the minor diastereomer (57.0 mg, 0.19 mmol, 19%) were obtained by flash column chromatography ($SiO_2$, EtOAc, stained with $KMnO_4$) both as white solids.

**Major diastereomer [3a]: M.P.**: 126 – 128 °C. **IR (neat)**: $\tilde{\nu}$ = 3381 (br), 3030 (w), 2918 (w), 1666 (s), 1486 (m), 1435 (m), 1255 (m), 1097 (w), 751 (m), 700 (m). **$^1$H NMR (600 MHz, $CDCl_3$)**: $\delta$ = 7.32 – 7.20 (m, 9H), 5.56 (d, $J$ = 6.6 Hz, 1H), 4.84 (*app.* dt, $J \approx$ 7.4, 6.7 Hz, 1H), 3.49 (*app.* p, $J \approx$ 8.7 Hz, 1H), 3.40-3.35 (m, 2H), 3.25 (ddd, $J$ = 16.3, 7.4, 0.9 Hz, 1H), 2.91 (dd, $J$ = 16.9, 8.9 Hz, 1H), 2.90 (ddd, $J$ = 16.3, 6.8, 1.1 Hz, 1H), 2.72 (dd, $J$ = 16.9, 9.4 Hz, 1H). **$^{13}$C NMR (151 MHz, $CDCl_3$)**: $\delta$ = 175.5, 141.9, 141.3, 137.9, 129.1, 128.9, 127.5, 127.2, 127.1, 125.9, 125.5, 73.8, 59.2, 53.3, 39.9, 39.4, 38.4. **HRMS (ESI)**: Calculated for $C_{19}H_{19}NO_2Na$ $[M+Na]^+$: 316.1308, Found: 316.1309.

**Optical Rotation**: $[\alpha]_D^{25}$ = −154.5 ($c$ = 0.50, $CHCl_3$). Absolute configuration was determined unambiguously by X-ray diffraction.

**Minor diastereomer [*epi*-3a]: M.P.**: 113 – 115 °C. **IR (neat)**: $\tilde{\nu}$ = 3376 (br), 2972 (m), 2901 (m), 1662 (s), 1482 (m), 1436 (m), 1291 (m), 1257 (m), 1076 (m), 1066 (m), 1057 (s), 749 (m), 700 (m). **$^1$H NMR (500 MHz, $CDCl_3$)**: $\delta$ = 7.34 – 7.10 (m, 9H), 5.58 (d, $J$ = 6.7 Hz, 1H), 4.84 (*app.* dt, $J \approx$ 7.4, 6.6 Hz, 1H), 3.67 (dd, $J$ = 9.6, 8.1 Hz, 1H), 3.61 (*app.* qd, $J \approx$ 8.2, 6.2 Hz, 1H), 3.29 (dd, $J$ = 16.3, 7.4 Hz, 1H), 3.10 (dd, $J$ = 9.6, 6.3 Hz, 1H), 2.98 (dd, $J$ = 16.3, 6.5 Hz, 1H), 2.93 (dd, $J$ = 16.9, 8.8 Hz, 1H), 2.68 (dd, $J$ = 16.9, 7.7 Hz, 1H).**$^{13}$C NMR (151 MHz, $CDCl_3$)**: $\delta$ = 175.5, 142.6, 141.2, 137.8, 129.0, 128.9, 127.4, 127.1, 126.8, 125.9, 125.5, 73.3, 59.1, 53.5, 40.0, 39.2, 37.5. **HRMS (ESI)**: Calculated for $C_{19}H_{19}NO_2Na$ $[M+Na]^+$: 316.1308, Found: 316.1304. **Optical Rotation**: $[\alpha]_D^{25}$ = +22.6 ($c$ = 1.00, $CHCl_3$). Absolute configuration was determined unambiguously by X-ray diffraction.

***Gram scale:*** 3-Phenylcyclobutan-1-one **[1a]** (1.32 g, 9.00 mmol, 1.00 equiv) and (1*S*,2*R*)-1-amino-2,3-dihydro-1H-inden-2-ol (1.47 g, 9.90 mmol, 1.10 equiv) were used as described above. Purification by flash column chromatography ($SiO_2$, EtOAc to EtOAc:MeOH 98:2, stained with $KMnO_4$) provided the major diastereomer **[3a]** (1.81 g, 6.18 mmol, 68% yield) and the minor diastereomer **[*epi*-3a]** (320 mg, 1.09 mmol, 12% yield) as white solids.

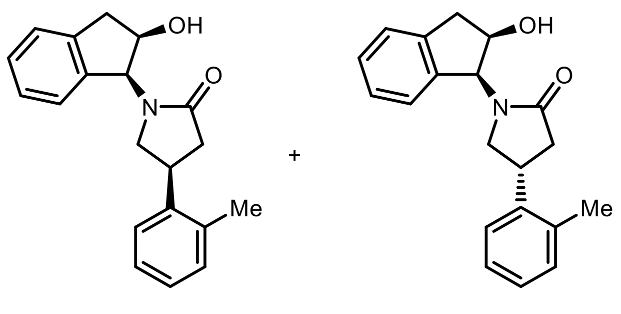


**1-((1*S*,2*R*)-2-Hydroxy-2,3-dihydro-1*H*-inden-1-yl)-4-(2-methylphenyl)pyrrolidin-2-one [3b, *epi*-3b]:**

Following the general procedure **D** using 3-(2-methylphenyl)cyclobutan-1-one **[1b]** (160 mg, 1.0 mmol, 1.0 equiv.) the major diastereomer (221 mg, 0.71 mmol, 71%) was obtained as an off-white solid by flash column chromatography ($SiO_2$, EtOAc, stained with $KMnO_4$) whereas the minor diastereomer (42.0 mg, 0.13 mmol, 13%) was obtained as a yellow oil. **Major diastereomer [3b]: M.P.**: 109 – 111 °C. **IR (neat)**: $\tilde{\nu}$ = 3354 (br), 2917 (br), 1664 (s), 1486 (w), 1436 (w), 1352 (w), 1292 (w), 1257 (w), 1097 (w), 753 (m). **$^1$H NMR (500 MHz, $CDCl_3$)**: $\delta$ = 7.35 (d, $J$ = 7.7 Hz, 1H), 7.32 – 7.21 (m, 4H), 7.17 (*app.* dp, $J \approx$ 7.9, 3.6 Hz, 1H), 7.13 – 7.08 (m, 2H), 5.57 (d, $J$ = 6.6 Hz, 1H), 4.84 (*app.* dt, $J \approx$ 7.2, 6.6 Hz, 1H), 3.70 (*app.* p, $J \approx$ 8.4 Hz, 1H), 3.47 – 3.35 (m, 2H), 3.25 (ddd, $J$ = 16.3, 7.4, 0.9 Hz, 1H), 2.96 – 2.84 (m, 2H), 2.66 (dd, $J$ = 16.9, 8.6 Hz, 1H), 2.27 (s, 3H). **$^{13}$C NMR (126 MHz, $CDCl_3$)**: $\delta$ = 175.5, 141.3, 140.2, 137.9, 135.8, 130.7, 129.0, 127.4, 126.9, 126.8, 125.8, 125.7, 125.5, 73.6, 59.2, 52.6, 40.0, 39.0, 34.1, 19.8. **HRMS (ESI)**: Calculated for $C_{20}H_{21}NO_2Na$ $[M+Na]^+$: 330.1465, Found: 330.1461. **Optical Rotation**: $[\alpha]_D^{25}$ = –77.29 ($c$ = 1.00, $CHCl_3$). Absolute configuration was determined through analogy to **[3a]**.

**Minor diastereomer [*epi*-3b]: IR (neat)**: $\tilde{\nu}$ = 3379 (br), 2916 (br), 1665 (s), 1484 (w), 1435 (w), 1272 (w), 1096 (w), 1058 (w), 751 (m). **$^1$H NMR (600 MHz, $CDCl_3$)**: $\delta$ = 7.31 – 7.10 (m, 8H), 5.59 (d, $J$ = 6.8 Hz, 1H), 4.85 (*app.* q, $J \approx$ 7.0 Hz, 1H), 3.85 – 3.76 (m,

1H), 3.72 (dd, $J$ = 9.8, 8.0 Hz, 1H), 3.29 (dd, $J$ = 16.3, 7.5 Hz, 1H), 3.06 (dd, $J$ = 9.8, 5.2 Hz, 1H), 3.02 – 2.90 (m, 2H), 2.63 (dd, $J$ = 17.0, 6.3 Hz, 1H), 2.26 (s, 3H). **$^{13}$C NMR (126 MHz, $CDCl_3$)**: $\delta$ = 175.6, 141.3, 141.0, 137.8, 135.6, 130.7, 129.0, 127.4, 126.8, 126.6, 125.9, 125.4, 125.1, 73.1, 59.0, 52.6, 40.0, 38.8, 33.1, 19.7. **HRMS (ESI)**: Calculated for $C_{20}H_{21}NO_2Na$ [M+Na]$^+$: 330.1465, Found: 330.1461. **Optical Rotation**: $[\alpha]_D^{25}$ = +43.38 ($c$ = 1.00, $CHCl_3$). Absolute configuration was determined through analogy to **[*epi*-3a]**.

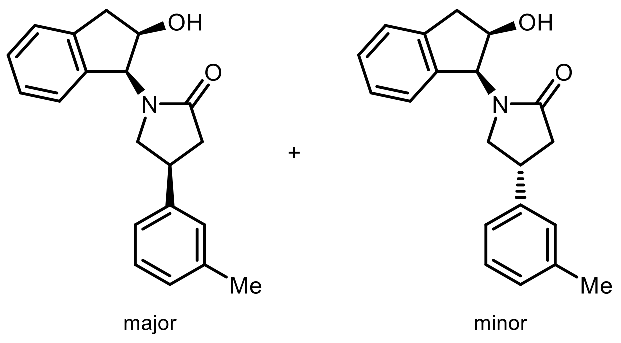


**1-((1*S*,2*R*)-2-Hydroxy-2,3-dihydro-1*H*-inden-1-yl)-4-(3-methylphenyl)pyrrolidin-2-one [3c, *epi*-3c]:**

Following the general procedure **D** using 3-(3-methylphenyl)cyclobutan-1-one **[1c]** (160 mg, 1.0 mmol, 1.0 equiv.) the major diastereomer (182 mg, 0.59 mmol, 59%) and the minor diastereomer (57.0 mg, 0.14 mmol, 14%) were obtained by flash column chromatography ($SiO_2$, EtOAc, stained with $KMnO_4$) both as yellow oils.

**Major diastereomer [3c]: IR (neat)**: $\tilde{\nu}$ = 3346 (br), 1666 (s), 1436 (w), 1258 (w), 1097 (w), 749 (w). **$^1$H NMR (600 MHz, $CDCl_3$)**: $\delta$ = 7.31 – 7.22 (m, 4H), 7.17 (t, $J$ = 7.6 Hz, 1H), 7.03 (*app.* dd, $J$ ≈ 6.9, 5.3 Hz, 3H), 5.55 (d, $J$ = 6.7 Hz, 1H), 4.83 (*app.* q, $J$ ≈ 7.0 Hz, 1H), 3.48 – 3.32 (m, 3H), 3.25 (dd, $J$ = 16.3, 7.4 Hz, 1H), 2.89 (*app.* ddd, $J$ ≈ 22.4, 16.6, 7.7 Hz, 2H), 2.71 (dd, $J$ = 16.8, 9.4 Hz, 1H), 2.30 (s, 3H). **$^{13}$C NMR (151 MHz, $CDCl_3$)**: $\delta$ = 175.5, 141.7, 141.4, 138.5, 138.0, 129.0, 128.8, 127.9, 127.8, 127.5, 125.9, 125.5, 124.1, 73.5, 59.1, 53.2, 39.9, 39.4, 38.4, 21.5. **HRMS (ESI)**: Calculated for $C_{20}H_{21}NO_2Na$ [M+Na]$^+$: 330.1465, Found: 330.1462. **Optical Rotation**: $[\alpha]_D^{25}$ = –61.88 ($c$ = 1.00, $CHCl_3$). Absolute configuration was determined through analogy to **[3a]**.

**Minor diastereomer [*epi*-3c]: IR (neat)**: $\tilde{\nu}$ = 3364 (br), 1666 (s), 1436 (w), 1262 (w), 1097 (w), 749 (w). **$^1$H NMR (600 MHz, $CDCl_3$)**: $\delta$ = 7.28 – 7.19 (m, 4H), 7.17 (t, $J$ = 7.7 Hz, 1H), 7.03 (d, $J$ = 7.6 Hz, 1H), 6.94 (*app.* d, $J$ ≈ 7.3 Hz, 2H), 5.57 (d, $J$ = 6.8 Hz, 1H), 4.83 (*app.* q, $J$ ≈ 7.0 Hz, 1H), 3.67 (dd, $J$ = 9.8, 8.1 Hz, 1H), 3.56 (*app.* p, $J$ ≈ 7.9 Hz, 1H), 3.28 (dd, $J$ = 16.4, 7.5 Hz, 1H), 3.07 (dd, $J$ = 9.8, 6.4 Hz, 1H), 2.99 – 2.88 (m, 2H), 2.66 (dd, $J$ = 16.9, 7.7 Hz, 1H), 2.31 (s, 3H). **$^{13}$C NMR (151 MHz, $CDCl_3$)**: $\delta$ = 175.6, 142.6, 141.3, 138.5, 137.8, 129.0, 128.8, 127.8, 127.5, 127.4, 126.0, 125.5, 123.8, 73.1, 58.9, 53.4, 40.0, 39.2, 37.3, 21.5. **HRMS (ESI)**: Calculated for $C_{20}H_{21}NO_2Na$ [M+Na]$^+$: 330.1465, Found: 330.1462. **Optical Rotation**: $[\alpha]_D^{25}$ = +45.31 ($c$ = 1.00, $CHCl_3$). Absolute configuration was determined through analogy to **[*epi*-3a]**.

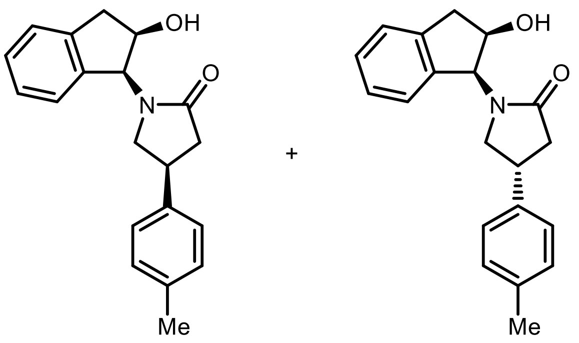

major minor

**1-((1*S*,2*R*)-2-Hydroxy-2,3-dihydro-1*H*-inden-1-yl)-4-(4-methylphenyl)pyrrolidin-2-one [3d, *epi*-3d]:**

Following the general procedure **D** using 3-(4-methylphenylcyclobutan-1-one **[1d]** (80.0 mg, 0.50 mmol, 0.5 equiv.) the major diastereomer (105 mg, 0.34 mmol, 68%) was obtained as an off-white solid by flash column chromatography ($SiO_2$, EtOAc, stained with $KMnO_4$) whereas the minor diastereomer (31.0 mg, 0.10 mmol, 20%) was obtained as a yellow oil.

**Major diastereomer [3d]: M.P.**: 127 – 129 °C. **IR (neat)**: $\tilde{\nu}$ = 3354 (br), 2922 (w), 1665 (s), 1517 (w), 1484 (w), 1436 (m, 1254 (m), 1097 (w), 816 (w), 750 (m). **$^1$H NMR (500 MHz, $CDCl_3$)**: $\delta$ = 7.33 – 7.21 (m, 4H), 7.15 – 7.07 (m, 4H), 5.56 (d, $J$ = 6.6 Hz, 1H), 4.84 (*app.* q, $J \approx$ 7.0 Hz, 1H), 3.50 – 3.31 (m, 3H), 3.25 (ddd, $J$ = 16.2, 7.5, 0.9 Hz, 1H), 2.95 – 2.84 (m, 2H), 2.70 (dd, $J$ = 16.9, 9.4 Hz, 1H), 2.30 (s, 3H).**$^{13}$C NMR (126 MHz, $CDCl_3$)**: $\delta$ = 175.4, 141.2, 138.7, 137.8, 136.6, 129.4, 128.9, 127.3, 126.8, 125.7, 125.3, 73.4, 59.0, 53.2, 39.8, 39.4, 38.0, 21.0. **HRMS (ESI)**: Calculated for $C_{20}H_{21}NO_2Na$ [M+Na]$^+$: 330.1465, Found: 330.1460. **Optical Rotation**: $[\alpha]_D^{25}$ = –98.07 ($c$ = 1.00, $CHCl_3$). Absolute configuration was determined through analogy to **[3a]**.

**Minor diastereomer [*epi*-3d]: IR (neat)**: $\tilde{\nu}$ = 3383 (br), 2924 (w), 1665 (s), 1517 (w), 1483 (w), 1436 (m), 1258 (w), 1097 (w), 817 (w), 749 (m). **$^1$H NMR (500 MHz, $CDCl_3$)**: $\delta$ = 7.29 – 7.23 (m, 2H), 7.24 – 7.17 (m, 2H), 7.13 – 7.02 (m, 4H), 5.59 (d, $J$ = 6.8 Hz, 1H), 4.85 (*app.* q, $J \approx$ 6.9 Hz, 1H), 3.68 (dd, $J$ = 9.7, 8.1 Hz, 1H), 3.63 – 3.55 (m, 1H), 3.30 (dd, $J$ = 16.3, 7.5 Hz, 1H), 3.08 (dd, $J$ = 9.7, 6.4 Hz, 1H), 2.98 (dd, $J$ = 16.4, 6.6 Hz, 1H), 2.92 (dd, $J$ = 16.9, 8.9 Hz, 1H), 2.66 (dd, $J$ = 16.8, 7.8 Hz, 1H), 2.32 (s, 3H).**$^{13}$C NMR (126 MHz, $CDCl_3$)**: $\delta$ = 175.5, 141.1, 139.5, 137.7, 136.6, 129.4, 128.8, 127.2, 126.5, 125.8, 125.3, 72.9, 58.8, 53.4, 39.9, 39.2, 37.0, 21.0. **HRMS (ESI)**: Calculated for $C_{20}H_{21}NO_2Na$ [M+Na]$^+$: 330.1465, Found: 330.1461. **Optical Rotation**: $[\alpha]_D^{25}$ = +55.12 ($c$ = 1.00, $CHCl_3$). Absolute configuration was determined through analogy to **[*epi*-3a]**.

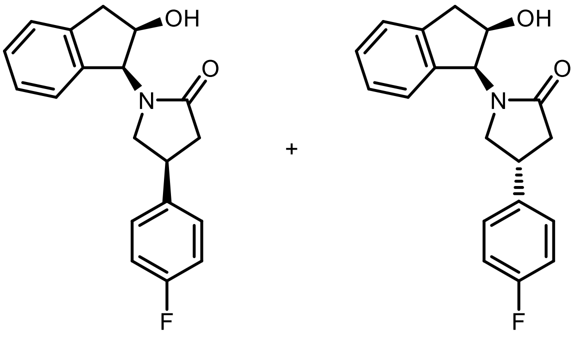

major minor

**4-(4-Fluorophenyl)-1-((1*S*,2*R*)-2-hydroxy-2,3-dihydro-1*H*-inden-1-yl)pyrrolidin-2-one [3e, *epi*-3e]:**

Following the general procedure **D** using 3-(4-fluorophenyl)cyclobutan-1-one **[1e]** (164 mg, 1.0 mmol, 1.0 equiv.) the major diastereomer (208 mg, 0.67 mmol, 67%) and the minor diastereomer (38.0 mg, 0.12 mmol, 12%) were obtained by flash column chromatography ($SiO_2$, EtOAc, stained with $KMnO_4$) both as white solids.

**Major diastereomer [3e]: M.P.**: 130 – 132 °C. **IR (neat)**: $\tilde{\nu}$ = 3363 (br), 2914 (br), 1665 (s), 1606 (w), 1513 (s), 1486 (w), 1437 (w), 1348 (w), 1294 (w), 1254 (w), 1224 (m), 1161 (w), 1060 (w), 837 (w), 750 (m). **$^{1}$H {$^{19}$F} NMR (500 MHz, $CDCl_3$)**: $\delta$ = 7.32 – 7.22 (m, 4H), 7.23 – 7.18 (m, 2H), 7.02 – 6.94 (m, 2H), 5.56 (d, *J* = 6.8, 1H), 4.84 (*app.* q, *J* = 7.1 Hz, 1H), 3.50 – 3.42 (m, 1H), 3.42 – 3.31 (m, 2H), 3.26 (dd, *J* = 16.3, 7.5 Hz, 1H), 2.90 (ddd, *J* = 16.2, 6.9, 1.2 Hz, 2H), 2.70 – 2.60 (m, 1H).**$^{13}$C {$^{19}$F} NMR (126 MHz, $CDCl_3$)**: $\delta$ = 175.2, 161.9, 141.4, 137.8, 137.7, 129.2, 128.6, 127.5, 125.9, 125.6, 115.7, 73.6, 59.1, 53.2, 40.0, 39.6, 37.7. **HRMS (ESI)**: Calculated for $C_{19}H_{18}FNO_2Na$ $[M+Na]^+$: 334.1214, Found: 334.1212. **Optical Rotation**: $[\alpha]_D^{25}$ = –83.08 (*c* = 1.00, $CHCl_3$). Absolute configuration was determined through analogy to **[3a]**.

**Minor diastereomer [*epi*-3e]: M.P.**: 112 – 114 °C. **IR (neat)**: $\tilde{\nu}$ = 3374 (br), 2914 (br), 1664 (s), 1606 (w), 1512 (s), 1483 (w), 1436 (m), 1293 (w), 1258 (m), 1161 (w), 1097 (w), 1059 (w), 836 (m), 749 (m). **$^{1}$H {$^{19}$F} NMR (500 MHz, $CDCl_3$)**: $\delta$ = 7.29 – 7.15 (m, 4H), 7.14 – 7.10 (m, 2H), 7.00 – 6.96 (m, 2H), 5.58 (d, *J* = 6.7 Hz, 1H), 4.85 (*app.* dt, *J* = 7.4, 6.7 Hz, 1H), 3.68 (dd, *J* = 9.7, 8.0 Hz, 1H), 3.60 (*app.* qd, *J* ≈ 8.0, 6.0 Hz, 1H), 3.30 (dd, *J* = 16.4, 7.4 Hz, 1H), 3.05 (dd, *J* = 9.7, 6.1 Hz, 1H), 3.00 – 2.90 (m, 2H), 2.88 (br s, 1H), 2.63 (dd, *J* = 16.8, 7.5 Hz, 1H).**$^{13}$C {$^{19}$F} NMR (126 MHz, $CDCl_3$)**: $\delta$ = 175.3, 161.9, 141.2, 138.4, 137.7, 129.1, 128.3, 127.4, 125.9, 125.5, 115.7, 73.2, 59.0, 53.5, 40.0, 39.3, 36.8. **HRMS (ESI)**: Calculated for $C_{19}H_{18}NO_2FNa$ $[M+Na]^+$: 334.1214, Found: 334.1210. **Optical Rotation**: $[\alpha]_D^{25}$ = +57.21 (*c* = 1.00, $CHCl_3$). Absolute configuration was determined through analogy to **[*epi*-3a]**.

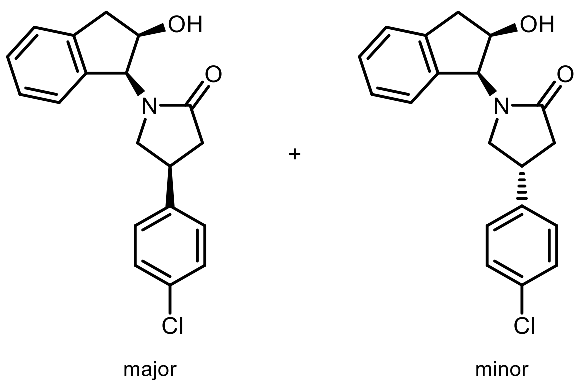


**4-(4-Chlorophenyl)-1-((1*S*,2*R*)-2-hydroxy-2,3-dihydro-1*H*-inden-1-yl)pyrrolidin-2-one [3f, *epi*-3f]:**

Following the general procedure **D** with minor alterations[e] using 3-(4-chlorophenyl)cyclobutan-1-one **[1f]** (1.44 g, 8.0 mmol, 1.0 equiv.) the major diastereomer (1.15 g, 3.5 mmol, 44%) and the minor diastereomer (462 mg, 1.4 mmol, 18%) were obtained by flash column chromatography ($SiO_2$, EtOAc, stained with $KMnO_4$) both as white solids.

[e] Hydrolysis was performed at 70 °C instead of room temperature.

**Major diastereomer [3f]: M.P.**: 134 – 136 °C. **IR (neat)**: $\tilde{\nu}$ = 3369 (br), 2916 (w), 1666 (s), 1493 (m), 1436 (m), 1254 (m), 1093 (m), 1014 (w), 825 (w), 751 (m). **$^1$H NMR (600 MHz, $CDCl_3$)**: $\delta$ = 7.34 – 7.23 (m, 6H), 7.19 – -7.14 (m, 2H), 5.55 (d, $J$ = 6.7 Hz, 1H), 4.84 (*app.* qd, $J$ ≈ 7.0, 3.9 Hz, 1H), 3.45 (*app.* p, $J$ ≈ 8.6 Hz, 1H), 3.40-3.31 (m, 2H), 3.26 (dd, $J$ = 16.3, 7.4 Hz, 1H), 2.94-2.87 (m, 2H), 2.70 (br s, 1H), 2.69-2.56 (m, 1H). **$^{13}$C NMR (151 MHz, $CDCl_3$)**: $\delta$ = 175.1, 141.3, 140.6, 137.8, 133.0, 129.2, 129.0, 128.5, 127.5, 125.9, 125.6, 73.7, 59.1, 53.0, 40.0, 39.5, 37.7. **HRMS (ESI)**: Calculated for $C_{19}H_{18}NO_2ClNa$ [M+Na]$^+$: 350.0918, Found: 350.0920. **Optical Rotation**: $[\alpha]_D^{25}$ = −113.0 (c = 1.00, $CHCl_3$). Absolute configuration was determined through analogy to **[3a]**.

**Minor diastereomer [*epi*-3f]: M.P.**: 115 – 117 °C. **IR (neat)**: $\tilde{\nu}$ = 3365 (br), 1665 (s), 1494 (m), 1437 (m), 1256 (w), 1092 (m), 1014 (w), 749 (w). **$^1$H NMR (600 MHz, $CDCl_3$)**: $\delta$ = 7.30 – 7.22 (m, 4H), 7.22 – 7.18 (m, 1H), 7.18 – 7.14 (m, 1H), 7.09 – 7.03 (m, 2H), 5.56 (d, $J$ = 6.7 Hz, 1H), 4.82 (*app.* q, $J$ ≈ 7.0 Hz, 1H), 3.69 (dd, $J$ = 9.9, 8.1 Hz, 1H), 3.61 – 3.54 (m, 1H), 3.52 (br s, 1H), 3.28 (dd, $J$ = 16.4, 7.5 Hz, 1H), 3.02 (dd, $J$ = 9.9, 6.1 Hz, 1H), 2.95 (dd, $J$ = 16.4, 6.6 Hz, 1H), 2.91 (dd, $J$ = 16.8, 8.8 Hz, 1H), 2.60 (dd, $J$ = 16.8, 7.3 Hz, 1H). **$^{13}$C NMR (151 MHz, $CDCl_3$)**: $\delta$ = 175.1, 141.2, 141.2, 137.7, 132.9, 129.1, 129.0, 128.1, 127.4, 125.8, 125.5, 73.0, 58.9, 53.2, 40.0, 39.1, 36.8. **HRMS (ESI)**: Calculated for $C_{19}H_{18}NO_2ClNa$ [M+Na]$^+$: 350.0918, Found: 350.0917. **Optical Rotation**: $[\alpha]_D^{25}$ = +77.8 (c = 0.50, $CHCl_3$). Absolute configuration was determined through analogy to **[*epi*-3a]**.

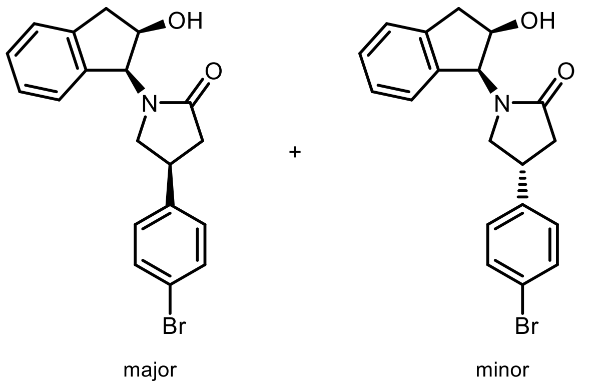


**4-(4-Bromophenyl)-1-((1*S*,2*R*)-2-hydroxy-2,3-dihydro-1*H*-inden-1-yl)pyrrolidin-2-one [3g, *epi*-3g]:**

Following the general procedure **D** with minor alterations[e] using 3-(4-bromophenyl)cyclobutan-1-one **[1g]** (225 mg, 1.0 mmol, 1.0 equiv.) the major diastereomer (223 mg, 0.60 mmol, 60%) was obtained as an off-white solid by flash column chromatography ($SiO_2$, pentane : EtOAc (1:4), stained with $KMnO_4$) whereas the minor diastereomer (70.0 mg, 0.19 mmol, 19%) was obtained as a yellow oil.

**Major diastereomer [3g]: M.P.**: 142 – 144 °C. **IR (neat)**: $\tilde{\nu}$ = 3374 (br), 1668 (s), 1489 (w), 1437 (w), 1253 (w), 1010 (w), 749 (w). **$^1$H NMR (600 MHz, $CDCl_3$)**: $\delta$ = 7.41 – 7.37 (m, 2H), 7.31 – 7.21 (m, 4H), 7.11 (d, $J$ = 8.2 Hz, 2H), 5.55 (d, $J$ = 6.7 Hz, 1H), 4.83 (*app.* q, $J$ ≈ 7.0 Hz, 1H), 3.42 (*app.* p, $J$ ≈ 8.6 Hz, 1H), 3.35 (d, $J$ = 7.6 Hz, 2H), 3.25 (dd, $J$ = 16.3, 7.5 Hz, 1H), 2.92 – 2.85 (m, 2H), 2.76 (*br.* s, 1H), 2.62 (dd, $J$ = 16.9, 9.1 Hz, 1H).**$^{13}$C NMR (151 MHz, $CDCl_3$)**: $\delta$ = 175.0, 141.3, 141.1, 137.8, 132.0, 129.1, 128.8, 127.5, 125.9, 125.5, 120.9, 73.4, 59.0, 52.8, 40.0, 39.4, 37.8. **HRMS (ESI)**: Calculated for $C_{19}H_{18}NO_2BrNa$ [M+Na]$^+$: 394.0413, 396.0395, Found: 394.0413,

[e] Hydrolysis was performed at 70 °C instead of room temperature.

396.0391. **Optical Rotation**: $[\alpha]_D^{25}$ = –103.33 ($c$ = 1.00, $CHCl_3$). Absolute configuration was determined through analogy to **[3a]**.
**Minor diastereomer [*epi*-3g]: IR (neat)**: $\tilde{\nu}$ = 3379 (br), 1667 (s), 1490 (w), 1439 (w), 1254 (w), 1010 (w), 822 (w), 751 (w). **$^1$H NMR (600 MHz, $CDCl_3$)**: $\delta$ = 7.40 (d, $J$ = 8.4 Hz, 2H), 7.29 – 7.23 (m, 2H), 7.20 (td, $J$ = 7.2, 1.7 Hz, 1H), 7.16 (d, $J$ = 7.5 Hz, 1H), 7.02 (d, $J$ = 8.4 Hz, 2H), 5.56 (d, $J$ = 6.7 Hz, 1H), 4.83 (q, $J$ = 6.9 Hz, 1H), 3.67 (dd, $J$ = 9.9, 8.0 Hz, 1H), 3.60 – 3.51 (m, 1H), 3.29 (dd, $J$ = 16.4, 7.5 Hz, 1H), 3.03 (dd, $J$ = 9.9, 6.0 Hz, 1H), 2.93 (ddd, $J$ = 16.7, 13.4, 7.8 Hz, 2H), 2.61 (dd, $J$ = 16.9, 7.3 Hz, 1H). **$^{13}$C NMR (151 MHz, $CDCl_3$)**: $\delta$ = 175.2, 141.8, 141.2, 137.6, 132.0, 129.1, 128.5, 127.5, 125.8, 125.5, 120.9, 73.2, 59.0, 53.2, 40.0, 39.1, 36.9. **HRMS (ESI)**: Calculated for $C_{19}H_{18}NO_2BrNa$ $[M+Na]^+$: 394.0413, 396.0395, Found: 394.0413, 396.0391. **Optical Rotation**: $[\alpha]_D^{25}$ = +76.25 ($c$ = 1.00, $CHCl_3$). Absolute configuration was determined through analogy to **[*epi*-3a]**.

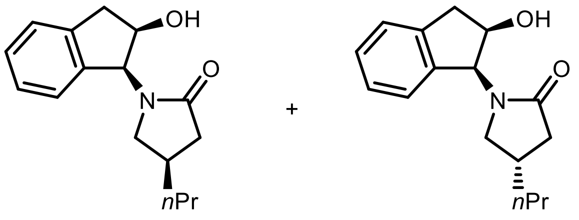

major minor

**1-((1*S*,2*R*)-2-Hydroxy-2,3-dihydro-1*H*-inden-1-yl)-4-propylpyrrolidin-2-one [3h, *epi*-3h]:**
Following the general procedure **D** using 3-propylcyclobutan-1-one **[1h]** (665 mg, 5.9 mmol, 1.0 equiv.) the major diastereomer (802 mg, 3.1 mmol, 53%) was obtained as an off-white solid by flash column chromatography ($SiO_2$, EtOAc, stained with $KMnO_4$) whereas the minor diastereomer (252 mg, 0.97 mmol, 16%) was obtained as colorless oil.
**Major diastereomer [3h]: M.P.**: 125 – 127 °C. **IR (neat)**: $\tilde{\nu}$ = 3364 (br), 2957 (w), 2925 (w), 1665 (s), 1484 (w), 1440 (w), 1287 (w), 1242 (w), 1096 (w), 740 (w). **$^1$H NMR (600 MHz, $CDCl_3$)**: $\delta$ = 7.45 – 7.36 (m, 3H), 7.34 (d, $J$ = 7.4 Hz, 1H), 5.61 (d, $J$ = 6.7 Hz, 1H), 4.93 (*app.* q, $J \approx$ 6.9 Hz, 1H), 3.39 (dd, $J$ = 16.2, 7.3 Hz, 1H), 3.24 (dd, $J$ = 9.6, 7.7 Hz, 1H), 3.13 (dd, $J$ = 9.6, 7.6 Hz, 1H), 3.06 (dd, $J$ = 16.2, 6.7 Hz, 1H), 2.73 (dd, $J$ = 16.0, 7.8 Hz, 1H), 2.41 – 2.28 (m, 2H), 1.61 – 1.47 (m, 2H), 1.46 – 1.33 (m, 2H), 1.01 (t, $J$ = 7.3 Hz, 3H). **$^{13}$C NMR (151 MHz, $CDCl_3$)**: $\delta$ = 176.3, 141.3, 138.2, 128.9, 127.4, 125.8, 125.5, 73.6, 58.9, 51.9, 40.0, 38.3, 36.7, 32.8, 20.8, 14.1. **HRMS (ESI)**: Calculated for $C_{16}H_{21}NO_2Na$ $[M+Na]^+$: 282.1465, Found: 282.1463. **Optical Rotation**: $[\alpha]_D^{25}$ = –32.6 ($c$ = 1.00, $CHCl_3$). Absolute configuration was determined through analogy to **[3a]**.
**Minor diastereomer [*epi*-3h]: IR (neat)**: $\tilde{\nu}$ = 3372 (br), 2956 (w), 2926 (w), 1663 (s), 1484 (w), 1442 (w), 1287 (w), 1245 (w), 1096 (w), 745 (w). **$^1$H NMR (600 MHz, $CDCl_3$)**: $\delta$ = 7.33 – 7.22 (m, 4H), 7.20 (dd, $J$ = 7.5, 1.2 Hz, 1H), 5.49 (d, $J$ = 6.7 Hz, 1H), 4.85 – 4.72 (m, 1H), 3.38 (dd, $J$ = 9.7, 7.7 Hz, 1H), 3.27 (dd, $J$ = 16.3, 7.4 Hz, 1H), 2.94 (dd, $J$ = 16.2, 6.5 Hz, 1H), 2.72 (dd, $J$ = 9.8, 6.0 Hz, 1H), 2.61 (dd, $J$ = 16.7, 8.5 Hz, 1H), 2.40 – 2.31 (m, 1H), 2.17 (dd, $J$ = 16.7, 7.1 Hz, 1H), 1.42 – 1.15 (m, 4H), 0.87 (t, $J$ = 7.2 Hz, 3H). **$^{13}$C NMR (151 MHz, $CDCl_3$)**: $\delta$ = 176.2, 141.3, 138.1, 128.9, 127.3, 125.9, 125.5, 73.2, 58.9, 51.9, 40.0, 38.0, 36.7, 31.9, 20.6, 14.1. **HRMS (ESI)**: Calculated for $C_{16}H_{21}NO_2Na$ $[M+Na]^+$: 282.1465, Found: 282.1465. **Optical Rotation**: $[\alpha]_D^{25}$ = +14.96 ($c$ = 1.00, $CHCl_3$). Absolute configuration was determined through analogy to **[*epi*-3a]**.

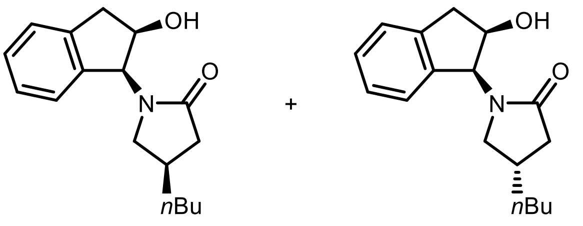

major minor

**4-Butyl-1-((1*S*,2*R*)-2-hydroxy-2,3-dihydro-1*H*-inden-1-yl)pyrrolidin-2-one [3i, *epi*-3i]:**

Following the general procedure **D** with minor alterations[f] using 3-butylcyclobutanone **[1i]** (126 mg, 1.0 mmol, 1.0 equiv.) the major diastereomer (162 mg, 0.59 mmol, 59%) and the minor diastereomer (41.5 mg, 0.15 mmol, 15%) were obtained by flash column chromatography (SiO2, EtOAc, stained with KMnO4) both as colorless oils.

**Major diastereomer [3i]: IR (neat)**: $\tilde{\nu}$ = 3355 (br), 2920 (w), 1661 (s), 1435 (m), 1272 (m), 1095 (m), 863 (w), 817 (w), 739 (m). **$^1$H NMR (500 MHz, $CDCl_3$)**: $\delta$ = 7.31 − 7.21 (m, 3H), 7.21 − 7.16 (m, 1H), 5.47 (d, $J$ = 6.7 Hz, 1H), 4.79 (*app.* q, $J \approx$ 6.9 Hz, 1H), 3.24 (dd, $J$ = 16.2, 7.3 Hz, 1H), 3.09 (dd, $J$ = 9.6, 7.8 Hz, 1H), 2.99 (dd, $J$ = 9.5, 7.8 Hz, 1H), 2.92 (dd, $J$ = 16.2, 6.7 Hz, 1H), 2.63 − 2.52 (m, 1H), 2.26 − 2.13 (m, 2H), 1.47 − 1.35 (m, 2H), 1.31 − 1.11 (m, 4H), 0.85 (t, $J$ = 7.1 Hz, 3H). **$^{13}$C NMR (126 MHz, $CDCl_3$)**: $\delta$ = 176.2, 141.3, 138.2, 128.9, 127.4, 125.8, 125.4, 73.5, 58.9, 51.8, 40.0, 38.3, 34.2, 33.0, 29.9, 22.7, 14.1. **HRMS (ESI)**: Calculated for $C_{17}H_{23}NO_2Na$ $[M+Na]^+$: 296.1621, Found: 296.1617. **Optical Rotation**: $[\alpha]_D^{20}$ = −19.8 ($c$ = 1.00, MeOH). Absolute configuration was determined through analogy to **[3a]**.

**Minor diastereomer [*epi*-3i]: IR (neat)**: $\tilde{\nu}$ = 3353 (br), 2921 (m), 1661 (s), 1437 (m), 1273 (m), 1095 (m), 1058 (m), 865 (w), 817 (w), 743 (m). **$^1$H NMR (600 MHz, $CDCl_3$)**: $\delta$ = 7.31 − 7.22 (m, 3H), 7.21 − 7.15 (m, 1H), 5.47 (d, $J$ = 6.7 Hz, 1H), 4.78 (*app.* dt, $J \approx$ 7.3, 6.6 Hz, 1H), 3.37 (ddd, $J$ = 9.5, 7.6, 1.7 Hz, 1H), 3.25 (dd, $J$ = 16.3, 7.4 Hz, 1H), 2.93 (dd, $J$ = 16.3, 6.5 Hz, 1H), 2.73 − 2.69 (m, 1H), 2.64 − 2.58 (m, 1H), 2.36 − 2.27 (m, 1H), 2.17 (dd, $J$ = 16.8, 7.0 Hz, 1H), 1.40 − 1.10 (m, 6H), 0.85 (t, $J$ = 7.2 Hz, 3H). **$^{13}$C NMR (151 MHz, $CDCl_3$)**: $\delta$ = 176.2, 141.3, 138.1, 128.9, 127.3, 125.9, 125.5, 73.3, 59.0, 52.0, 40.0, 38.1, 34.2, 32.1, 29.6, 22.7, 14.1. **HRMS (ESI)**: Calculated for $C_{17}H_{23}NO_2Na$ $[M+Na]^+$: 296.1621, Found: 296.1617. **Optical Rotation**: $[\alpha]_D^{20}$ = +11.5 ($c$ = 1.00, $CHCl_3$). Absolute configuration was determined through analogy to **[*epi*-3a]**.

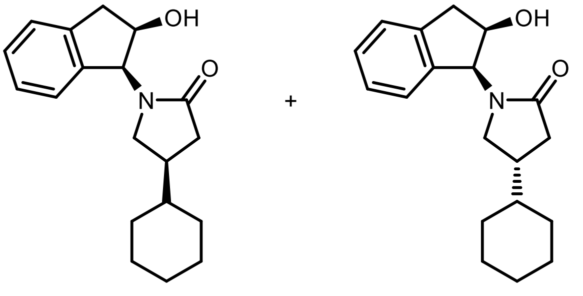

major minor

**4-Cyclohexyl-1-((1*S*,2*R*)-2-hydroxy-2,3-dihydro-1*H*-inden-1-yl)pyrrolidin-2-one [3j, *epi*-3j]:**

[f] Ring expansion was performed with TCICA (116 mg, 0.50 mmol, 0.50 equiv.).

Following the general procedure **D** with minor alterations[f] using 3-cyclohexylcyclobutanone **[1j]** (152 mg, 1.0 mmol, 1.0 equiv.) the major diastereomer (190 mg, 0.63 mmol, 63%) was obtained by flash column chromatography (SiO2, EtOAc, stained with $KMnO_4$) as a white solid, whereas the minor diastereomer (20.4 mg, 68 µmol, 7%) was obtained as a colorless oil.

**Major diastereomer [3j]: M.P.**: 64 − 66°C. **IR (neat)**: $\tilde{\nu}$ = 3356 (br), 2921 (m), 2850 (m), 1660 (s), 1443 (m), 1295 (m), 1263 (m), 1094 (m), 909 (w), 818 (w), 731 (s). **$^1$H NMR (500 MHz, $CDCl_3$)**: $\delta$ = 7.32 − 7.22 (m, 3H), 7.21 − 7.15 (m, 1H), 5.47 (d, $J$ = 6.7 Hz, 1H), 4.78 (*app.* qd, $J \approx$ 7.0, 2.9 Hz, 1H), 3.24 (dd, $J$ = 16.2, 7.3 Hz, 1H), 3.10 − 3.01 (m, 2H), 2.98 (br s, 1H), 2.92 (dd, $J$ = 16.2, 6.8 Hz, 1H), 2.52 (dd, $J$ = 16.7, 8.6 Hz, 1H), 2.23 (dd, $J$ = 16.7, 10.5 Hz, 1H), 2.03 − 1.91 (m, 1H), 1.74 − 1.59 (m, 4H), 1.51 (d, $J$ = 13.1 Hz, 1H), 1.28 − 1.05 (m, 4H), 0.95 − 0.75 (m, 2H). **$^{13}$C NMR (126 MHz, $CDCl_3$)**: $\delta$ = 176.4, 141.3, 138.2, 128.9, 127.4, 125.8, 125.5, 73.6, 59.0, 50.3, 42.3, 40.0, 39.2, 36.6, 31.4, 31.0, 26.4, 26.1, 26.0. **HRMS (ESI)**: Calculated for $C_{19}H_{25}NO_2Na$ $[M+Na]^+$: 322.1778, Found: 322.1774. **Optical Rotation**: $[\alpha]_D^{20}$ = −25.0 ($c$ = 1.00, MeOH). Absolute configuration was determined through analogy to **[3a]**.

**Minor diastereomer [*epi*-3j]: IR (neat)**: $\tilde{\nu}$ = 3334 (br), 2921 (m), 2851 (m), 1661 (s), 1445 (m), 1296 (m), 1274 (m), 1095 (m), 908 (w), 817 (w), 732 (s). **$^1$H NMR (500 MHz, $CDCl_3$)**: $\delta$ = 7.31 − 7.21 (m, 3H), 7.21 − 7.16 (m, 1H), 5.46 (d, $J$ = 6.7 Hz, 1H), 4.77 (*app.* q, $J \approx$ 6.9 Hz, 1H), 3.31 − 3.15 (m, 2H), 2.94 (dd, $J$ = 16.3, 6.4 Hz, 1H), 2.79 (dd, $J$ = 9.7, 7.8 Hz, 1H), 2.52 (dd, $J$ = 16.5, 8.5 Hz, 1H), 2.22 (dd, $J$ = 16.5, 9.3 Hz, 1H), 2.13 (*app.* sext, $J \approx$ 8.4 Hz, 1H), 1.75 − 1.57 (m, 4H), 1.51 − 1.43 (m, 1H), 1.23 − 1.02 (m, 4H), 0.96 − 0.74 (m, 2H). **$^{13}$C NMR (126 MHz, $CDCl_3$)**: $\delta$ = 176.4, 141.3, 138.0, 128.9, 127.3, 126.0, 125.5, 73.2, 58.9, 50.5, 42.1, 40.0, 38.3, 36.2, 31.0, 30.7, 26.4, 26.1, 26.0. **HRMS (ESI)**: Calculated for $C_{19}H_{25}NO_2Na$ $[M+Na]^+$: 322.1778, Found: 322.1774. **Optical Rotation**: $[\alpha]_D^{20}$ = +13.9 ($c$ = 1.00, $CHCl_3$). Absolute configuration was determined through analogy to **[*epi*-3a]**.

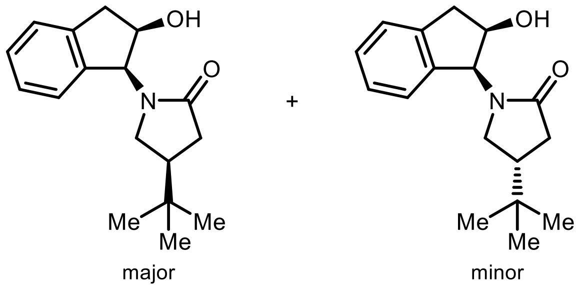


**4-(*tert*-Butyl)-1-((1*S*,2*R*)-2-hydroxy-2,3-dihydro-1*H*-inden-1-yl)pyrrolidin-2-one [3k, *epi*-3k]:**

Following the general procedure **D** using 3-(*tert*-butyl)cyclobutanone **[1k]** (126 mg, 1.0 mmol, 1.0 equiv.) the major diastereomer (184 mg, 0.67 mmol, 67%) was obtained by gradient flash column chromatography ($SiO_2$, EtOAc : pentane (9:1 to 1:0), stained with $KMnO_4$) as a white solid, whereas the minor diastereomer (22.3 mg, 82 µmol, 8%) was obtained as a colorless oil.

**Major diastereomer [3k]: M.P.**: 159 − 161°C. **IR (neat)**: $\tilde{\nu}$ = 3358 (br), 2957 (m), 2088 (w), 1663 (s), 1443 (m), 1282 (m), 1250 (m), 1186 (w), 1096 (m), 914 (w), 748 (m), 680 (w). **$^1$H NMR (500 MHz, $CDCl_3$)**: $\delta$ = 7.33 − 7.22 (m, 3H), 7.19 (d, $J$ = 7.3 Hz, 1H), 5.48 (d, $J$ = 6.7 Hz, 1H), 4.79 (*app.* dt, $J \approx$ 7.3, 6.6 Hz, 1H), 3.25 (dd, $J$ = 16.3, 7.3 Hz, 1H),

[f] Ring expansion was performed with TCICA (116 mg, 0.50 mmol, 0.50 equiv.).

3.16 (*app.* t, $J \approx 9.3$ Hz, 1H), 2.97 (dd, $J = 9.8, 8.6$ Hz, 1H), 2.94 (dd, $J = 16.2, 6.5$ Hz, 1H), 2.63 (br. s, 1H), 2.46 − 2.31 (m, 2H), 2.14 (*app.* dq, $J \approx 9.8, 8.8$ Hz, 1H), 0.84 (s, 9H). **$^{13}$C NMR (126 MHz, $CDCl_3$)**: $\delta$ = 176.3, 141.2, 138.1, 128.9, 127.4, 125.8, 125.5, 73.6, 59.1, 47.6, 43.2, 40.1, 33.5, 31.6, 27.0. **HRMS (ESI)**: Calculated for $C_{17}H_{23}NO_2Na$ $[M+Na]^+$: 296.1621, Found: 296.1616. **Optical Rotation**: $[\alpha]_D^{20}$ = −29.8 (*c* = 1.00, $CHCl_3$). Absolute configuration was determined through analogy to **[3a]**.

**Minor diastereomer [*epi*-3k]: IR (neat)**: $\tilde{\nu}$ = 3354 (br), 2957 (m), 2034 (w), 1663 (s), 1444 (m), 1367 (m), 1283 (m), 1186 (w), 1096 (m), 1022 (w), 864 (w), 748 (m), 679 (w). **$^1$H NMR (500 MHz, $CDCl_3$)**: $\delta$ = 7.35 − 7.22 (m, 3H), 7.20 (d, $J = 7.4$ Hz, 1H), 5.48 (d, $J = 6.8$ Hz, 1H), 4.79 (*app.* q, $J \approx 6.9$ Hz, 1H), 3.26 (dd, $J = 16.3, 7.5$ Hz, 1H), 3.20 (dd, $J = 10.0, 8.6$ Hz, 1H), 2.95 (dd, $J = 16.3, 6.7$ Hz, 1H), 2.86 (dd, $J = 10.0, 7.3$ Hz, 1H), 2.60 (br s, 1H), 2.45 (dd, $J = 17.0, 9.5$ Hz, 1H), 2.35 (dd, $J = 17.0, 8.7$ Hz, 1H), 2.24 (*app.* dt, $J \approx 16.9, 8.8$ Hz, 1H), 0.79 (s, 9H). **$^{13}$C NMR (126 MHz, $CDCl_3$)**: $\delta$ = 176.3, 141.3, 137.9, 129.0, 127.3, 125.9, 125.5, 73.2, 59.0, 47.8, 42.1, 40.0, 33.4, 31.8, 26.8. **HRMS (ESI)**: Calculated for $C_{17}H_{23}NO_2Na$ $[M+Na]^+$: 296.1621, Found: 296.1614. **Optical Rotation**: $[\alpha]_D^{20}$ = +6.67 (*c* = 1.00, $CHCl_3$). Absolute configuration was determined through analogy to **[*epi*-3a]**.

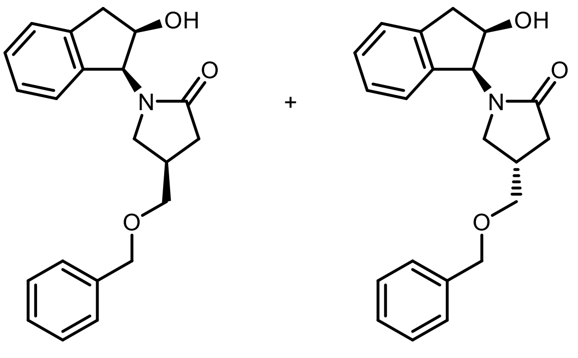


**4-((Benzyloxy)methyl)-1-((1*S*,2*R*)-2-hydroxy-2,3-dihydro-1*H*-inden-1-yl)pyrrolidin-2-one [3l, *epi*-3l]:**

Following the general procedure **D** with minor alterations[f] using 3-benzyloxymethylcyclobutanone **[1l]** (190 mg, 1.0 mmol, 1.0 equiv.) the major diastereomer (161 mg, 0.48 mmol, 48%) and the minor diastereomer (79.3 mg, 0.24 mmol, 24%) were obtained by flash column chromatography (SiO2, EtOAc, stained with KMnO4) both as colorless oils.

**Major diastereomer [3l]: IR (neat)**: $\tilde{\nu}$ = 3370 (br), 2914 (w), 1662 (s), 1440 (m), 1365 (w), 1273 (m), 1093 (s), 909 (m), 816 (w), 734 (s), 699 (m). **$^1$H NMR (500 MHz, $CDCl_3$)**: $\delta$ = 7.38 − 7.32 (m, 2H), 7.31 − 7.24 (m, 5H), 7.24 − 7.16 (m, 2H), 5.49 (d, $J = 6.2$ Hz, 1H), 4.72 (*app.* q, $J \approx 6.5$ Hz, 1H), 4.56 − 4.44 (m, 2H), 3.49 (dd, $J = 9.1, 4.4$ Hz, 1H), 3.43 − 3.37 (m, 2H), 3.24 (br s, 1H), 3.23 − 3.14 (m, 2H), 2.91 (dd, $J = 16.5, 5.0$ Hz, 1H), 2.68 (dd, $J = 16.8, 9.2$ Hz, 1H), 2.54 − 2.45 (m, 1H), 2.33 (dd, $J = 16.8, 4.4$ Hz, 1H). **$^{13}$C NMR (126 MHz, $CDCl_3$)**: $\delta$ = 175.4, 141.4, 137.8, 137.4, 128.7, 128.7, 128.1, 128.1, 127.1, 125.7, 125.6, 73.7, 73.6, 73.3, 59.5, 49.9, 39.9, 35.3, 31.9. **HRMS (ESI)**: Calculated for $C_{21}H_{23}NO_3Na$ $[M+Na]^+$: 360.1570, Found: 360.1568. **Optical Rotation**: $[\alpha]_D^{20}$ = −30.8 (*c* = 1.00, MeOH). Absolute configuration was determined through analogy to **[3a]**.

---

[f] Ring expansion was performed with TCICA (116 mg, 0.50 mmol, 0.50 equiv.).

**Minor diastereomer [*epi*-3l]: IR (neat)**: $\tilde{\nu}$ = 3354 (br), 2920 (w), 1659 (s), 1441 (m), 1362 (w), 1273 (m), 1199 (w), 1093 (s), 905 (w), 816 (w), 734 (s), 698 (s). **$^{1}$H NMR (500 MHz, CDCl$_3$)**: $\delta$ = 7.37 − 7.20 (m, 8H), 7.16 (d, $J$ = 7.5 Hz, 1H), 5.47 (d, $J$ = 6.7 Hz, 1H), 4.78 (*app.* q, $J$ ≈ 6.6 Hz, 1H), 4.48 − 4.40 (m, 2H), 3.41 (dd, $J$ = 10.0, 7.4 Hz, 1H), 3.38 − 3.29 (m, 2H), 3.25 (dd, $J$ = 16.3, 7.4 Hz, 1H), 3.00 − 2.88 (m, 3H), 2.69 − 2.58 (m, 2H), 2.36 − 2.26 (m, 1H). **$^{13}$C NMR (126 MHz, CDCl$_3$)**: $\delta$ = 175.6, 141.3, 138.0, 137.9, 129.0, 128.6, 127.9, 127.8, 127.4, 125.9, 125.5, 73.4, 73.4, 72.1, 59.0, 48.8, 39.9, 34.8, 31.7. **HRMS (ESI)**: Calculated for $C_{21}H_{23}NO_3Na$ [M+Na]$^+$: 360.1570, Found: 360.1568. **Optical Rotation**: $[\alpha]_D^{20}$ = +33.4 ($c$ = 1.00, MeOH). Absolute configuration was determined through analogy to **[*epi*-3a]**.

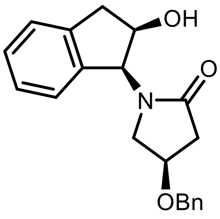

major

**4-(Benzyloxy)-1-((1*S*,2*R*)-2-hydroxy-2,3-dihydro-1*H*-inden-1-yl)pyrrolidin-2-one [3m]:**

Following the general procedure **D** using 3-(benzyloxy)cyclobutan-1-one **[1m]** (176 mg, 1.0 mmol, 1.0 equiv.) the major diastereomer (175 mg, 0.54 mmol, 54%) was obtained by flash column chromatography ($SiO_2$, EtOAc, stained with $KMnO_4$) as clear oil.

**Major diastereomer [3m]: IR (neat)**: $\tilde{\nu}$ = 3376 (br), 3028 (w), 2913 (br), 1668 (s), 1481 (w), 1454 (m), 1435 (m), 1350 (m), 1300 (m), 1280 (w), 1096 (m), 1028 (m), 1028 (w), 813 (w), 742 (m), 699 (m). **$^{1}$H NMR (600 MHz, CDCl$_3$)**: $\delta$ = 7.33 – 7.24 (m, 7H), 7.25 –7.19 (m, 1H), 7.16 (d, $J$ = 7.5 Hz, 1H), 5.51 (d, $J$ = 6.1 Hz, 1H), 4.79 (td, $J$ = 6.5, 4.6 Hz, 1H), 4.54 – 4.35 (m, 2H), 4.15 (tt, $J$ = 6.1, 3.1 Hz, 1H), 3.44 (dd, $J$ = 10.9, 2.9 Hz, 1H), 3.40 (dd, $J$ = 10.9, 5.3 Hz, 1H), 3.21 (dd, $J$ = 16.5, 6.8 Hz, 1H), 2.95 (dd, $J$ = 16.6, 4.7 Hz, 1H), 2.72 (dd, $J$ = 17.1, 6.2 Hz, 1H), 2.64 (dd, $J$ = 17.2, 3.2 Hz, 1H), 2.50 (s, 1H). **$^{13}$C NMR (151 MHz, CDCl$_3$)**: $\delta$ = 174.0, 141.3, 137.8, 137.4, 128.8, 128.6, 128.1, 127.8, 127.2, 125.7, 125.5, 73.8, 72.1, 70.9, 59.2, 52.5, 39.9, 38.6. **HRMS (ESI)**: Calculated for $C_{20}H_{21}NO_3Na$ [M+Na]$^+$: 346.1414, Found: 316.1411. **Optical Rotation**: $[\alpha]_D^{25}$ = –44.71 ($c$ = 1.00, $CHCl_3$). Absolute configuration was determined through analogy to **[3a]**.

The minor diastereomer **[*epi*-3m]** was not isolated.

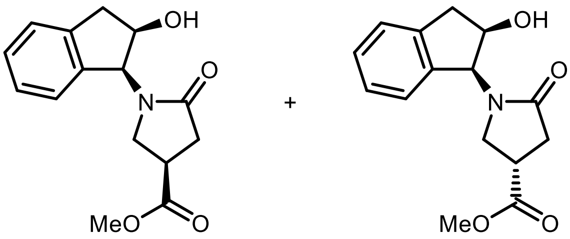

major minor

**1-Methyl-((1*S*,2*R*)-2-hydroxy-2,3-dihydro-1*H*-inden-1-yl)-5-oxopyrrolidine-3-carboxylate [3n, *epi*-3n]:**

Following the general procedure **D** with minor alterations[f] using methyl 3-oxocyclobutane-1-carboxylate **[1n]** (128 mg, 1.0 mmol, 1.0 equiv.) the major diastereomer (80.0 mg, 0.29 mmol, 29%) was obtained by flash column chromatography (SiO2, EtOAc, stained with $KMnO_4$) as a white solid, whereas the minor diastereomer (41.0 mg, 0.15 mmol, 15%) was obtained as a colorless oil.

**Major diastereomer [3n]: IR (neat)**: **M.P.**: 99 − 101°C. $\tilde{\nu}$ = 3365 (br), 2920 (w), 1735 (s), 1668 (s), 1436 (m), 1366 (w), 1271 (m), 1197 (m), 1057 (w), 937 (w), 749 (m), 680 (w). **$^1$H NMR (500 MHz, $CDCl_3$)**: $\delta$ = 7.30 − 7.19 (m, 3H), 7.19 − 7.15 (m, 1H), 5.49 (d, $J$ = 6.4 Hz, 1H), 4.77 (*app.* ddd, $J \approx$ 7.0, 6.3, 5.5 Hz, 1H), 3.69 (s, 3H), 3.54 (dd, $J$ = 10.3, 5.4 Hz, 1H), 3.34 (dd, $J$ = 10.3, 8.0 Hz, 1H), 3.23 (dd, $J$ = 16.4, 7.0 Hz, 1H), 3.14 (*app.* qd, $J \approx$ 7.8, 5.5 Hz, 1H), 2.93 (dd, $J$ = 16.5, 5.5 Hz, 1H), 2.76 (d, $J$ = 7.9 Hz, 2H). **$^{13}$C NMR (126 MHz, $CDCl_3$)**: $\delta$ = 174.0, 173.7, 141.4, 137.5, 128.9, 127.3, 125.7, 125.6, 73.6, 59.2, 52.7, 48.0, 40.0, 37.0, 34.7. **HRMS (ESI)**: Calculated for $C_{15}H_{17}NO_4Na$ $[M+Na]^+$: 298.1050, Found: 298.1047. **Optical Rotation**: $[\alpha]_D^{20}$ = −48.5 ($c$ = 1.00, $CHCl_3$). Absolute configuration was determined through analogy to **[3a]**.

**Minor diastereomer [*epi*-3n]: IR (neat)**: $\tilde{\nu}$ = 3356 (br), 2951 (w), 1734 (s), 1663 (s), 1436 (m), 1359 (w), 1272 (m), 1200 (m), 1057 (m), 940 (w), 816 (w), 748 (m). **$^1$H NMR (600 MHz, $CDCl_3$)**: $\delta$ = 7.33 − 7.22 (m, 4H), 5.50 (d, $J$ = 6.5 Hz, 1H), 4.79 (*app.* q, $J \approx$ 6.7 Hz, 1H), 3.68 (s, 3H), 3.53 (dd, $J$ = 10.1, 8.5 Hz, 1H), 3.32 (dd, $J$ = 10.1, 5.4 Hz, 1H), 3.26 (dd, $J$ = 16.4, 7.4 Hz, 1H), 3.24 − 3.21 (m, 1H), 2.92 (dd, $J$ = 16.4, 6.1 Hz, 1H), 2.85 − 2.72 (m, 2H). **$^{13}$C NMR (125 MHz, $CDCl_3$)**: $\delta$ = 174.1, 173.5, 141.1, 137.5, 129.1, 127.5, 126.0, 125.5, 73.2, 59.1, 52.5, 48.1, 40.1, 36.5, 34.5. **HRMS (ESI)**: Calculated for $C_{15}H_{17}NO_4Na$ $[M+Na]^+$: 298.1050, Found: 298.1045. **Optical Rotation**: $[\alpha]_D^{20}$ = +15.8 ($c$ = 1.00, MeOH). Absolute configuration was determined through analogy to **[*epi*-3a]**.

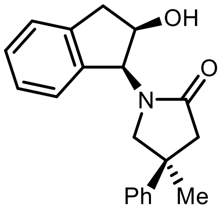

major

**1-((1*S*,2*R*)-2-Hydroxy-2,3-dihydro-1*H*-inden-1-yl)-4-methyl-4-phenylpyrrolidin-2-one [3o]:**

Following the general procedure **D** using 3-methyl-3-phenylcyclobutan-1-one **[1o]** (160 mg, 1.0 mmol, 1.0 equiv.) the major diastereomer (174 mg, 0.57 mmol, 57%) was obtained by flash column chromatography ($SiO_2$, pentane : EtOAc (1:2), stained with $KMnO_4$) as an off-white solid.

**Major diastereomer [3o]: M.P.**: 163 – 165 °C. **IR (neat)**: $\tilde{\nu}$ = 3367 (br), 2959 (w), 1665 (s), 1497 (w), 1446 (m), 1374 (w), 1323 (m), 1253 (m), 1201 (w), 1095 (m), 1060 (w), 1030 (w), 751 (m), 700 (m). **$^1$H NMR (600 MHz, $CDCl_3$)**: $\delta$ = 7.38 – 7.24 (m, 6H), 7.20 (t, $J$ = 7.4 Hz, 1H), 7.15 – 7.08 (m, 2H), 5.60 (d, $J$ = 6.7 Hz, 1H), 4.81 (*app.* q, $J \approx$ 6.9 Hz, 1H), 3.70 (d, $J$ = 9.5 Hz, 1H), 3.25 (dd, $J$ = 16.3, 7.4 Hz, 1H), 3.07 (d, $J$ = 9.5 Hz, 1H), 2.95 (d, $J$ = 16.2 Hz, 1H), 2.90 (dd, $J$ = 16.3, 6.7 Hz, 1H), 2.79 (*br.* s, 1H), 2.59 (d, $J$ = 16.2 Hz, 1H), 1.37 (s, 3H). **$^{13}$C NMR (151 MHz, $CDCl_3$)**: $\delta$ = 175.1, 146.7, 141.5, 137.9, 129.0, 128.7, 127.5, 126.6, 125.8, 125.5 (3C), 73.2, 58.9, 57.7, 45.5, 41.1, 39.9, 29.7. **HRMS (ESI)**: Calculated for $C_{20}H_{21}NO_2Na$ $[M+Na]^+$: 330.1465, Found: 330.1462.

[f] Ring expansion was performed with TCICA (116 mg, 0.50 mmol, 0.50 equiv.).

**Optical Rotation**: $[\alpha]_D^{25}$ = –77.95 ($c$ = 1.00, $CHCl_3$). Absolute configuration was determined unambiguously by X-ray diffraction.
The minor diastereomer **[*epi*-3o]** was not isolated.

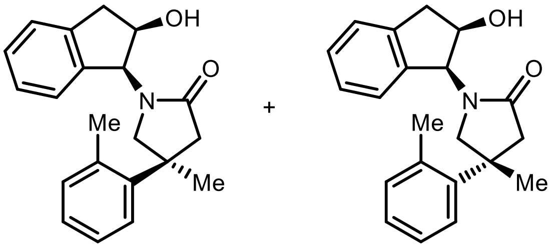


major minor

**1-((*1S,2R*)-2-hydroxy-2,3-dihydro-1*H*-inden-1-yl)-4-methyl-4-(o-tolyl)pyrrolidin-2-one [3p, *epi*-3p]:**
Following the general procedure **D** using 3-methyl-3-(o-tolyl)cyclobutanone **[1p]** (186 mg, 1.0 mmol, 1.0 equiv.) a mixture of diastereomers **[3p, *epi*-3p]** (252 mg, 0.78 mmol, 78 %) was obtained by flash column chromatography ($SiO_2$, EtOAc, stained with $KMnO_4$) as white solid.
**IR (neat, mix of diastereomers)**: $\tilde{\nu}$ = 3381 (br), 2960 (br), 1668 (s), 1447 (w), 1317 (w), 1249 (w), 1201 (w), 1095 (w), 1055 (w), 910 (w), 728 (m).
**Major diastereomer [3p]: $^1$H NMR (600 MHz, $CDCl_3$)**: $\delta$ = 7.36 – 7.23 (m, 4H), 7.13 – 6.89 (m, 4H), 5.60 (d, $J$ = 6.7 Hz, 1H), 4.83 (*app.* q, $J \approx$ 6.9 Hz, 1H), 3.87 (d, $J$ = 9.4 Hz, 1H), 3.27 (dd, $J$ = 16.3, 7.3 Hz, 1H), 3.18 (d, $J$ = 9.3 Hz, 1H), 2.97 (d, $J$ = 16.1 Hz, 1H), 2.93 (dd, $J$ = 16.4, 6.8 Hz, 1H), 2.73 (d, $J$ = 16.1 Hz, 1H), 2.25 (s, 3H), 1.35 (s, 3H). **$^{13}$C NMR (151 MHz, $CDCl_3$)**: δ = 174.8, 144.3, 141.4, 137.9, 135.4, 132.5, 129.0, 127.6, 126.8, 126.5, 126.4, 125.7, 125.5, 73.2, 58.8, 57.7, 46.3, 42.2, 40.0, 27.6, 21.9. Absolute configuration was determined through analogy to **[3o]**.
**Minor diastereomer [*epi*-3p]: $^1$H NMR (600 MHz, $CDCl_3$)**: $\delta$ = 7.36 – 6.89 (m, 8H), 5.54 (d, $J$ = 6.7 Hz, 1H), 4.87 (*app.* q, $J$ = 6.9 Hz, 1H), 3.51 (d, $J$ = 9.6 Hz, 1H), 3.44 (d, $J$ = 9.6 Hz, 1H), 3.30 (dd, $J$ = 16.3, 7.2 Hz, 1H), 3.01 (dd, $J$ = 17.0, 6.0 Hz, 1H), 2.99 (d, $J$ = 16.4 Hz, 1H), 2.69 (d, $J$ = 16.1 Hz, 1H), 2.24 (s, 3H), 1.52 (s, 3H). **$^{13}$C NMR (151 MHz, $CDCl_3$)**: δ = 175.0, 144.5, 141.1, 137.8, 135.4, 132.5, 129.0, 127.3, 126.8, 126.4 (2C), 125.9, 125.5, 73.7, 58.7, 58.6, 46.1, 41.9, 40.1, 28.4, 21.8.
**HRMS (ESI)**: Calculated for $C_{21}H_{23}NO_2Na$ $[M+Na]^+$: 344.1621, Found: 344.1615.

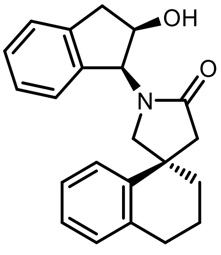


major

**1'-((1*S*,2*R*)-2-hydroxy-2,3-dihydro-1*H*-inden-1-yl)-3,4-dihydro-2H-spiro[naphthalene-1,3'-pyrrolidin]-5'-one [3q]:**
Following the general procedure **D** using 3',4'-dihydro-2'H-spiro[cyclobutane-1,1'-naphthalen]-3-one **[1q]** (186 mg, 1.0 mmol, 1.0 equiv.) the major diastereomer

(144 mg, 0.43 mmol, 43%) was obtained by flash column chromatography ($SiO_2$, EtOAc, stained with $KMnO_4$) as an off-white solid.
**Major diastereomer [3q]: M.P.**: 159 – 161 °C. **IR (neat)**: $\tilde{\nu}$ = 3373 (br), 2929 (w), 1662 (s), 1485 (w), 1446 (w), 1320 (w), 1266 (w), 1098 (w), 1059 (w), 756 (m). **$^1$H NMR (500 MHz, $CDCl_3$)**: $\delta$ = 7.49 (dd, $J$ = 8.0, 1.3 Hz, 1H), 7.33 – 7.21 (m, 4H), 7.20 – 7.13 (m, 1H), 7.10 (td, $J$ = 7.4, 1.3 Hz, 1H), 7.03 (dd, $J$ = 7.6, 1.4 Hz, 1H), 5.61 (d, $J$ = 6.7 Hz, 1H), 4.88 (*app.* q, $J$ ≈ 7.1 Hz, 1H), 3.61 (d, $J$ = 10.1 Hz, 1H), 3.33 – 3.24 (m, 1H), 3.04 – 2.96 (m, 2H), 2.96 (br s, 1H), 2.90 (ddd, $J$ = 16.2, 7.0, 1.1 Hz, 1H), 2.83 – 2.67 (m, 2H), 2.64 (d, $J$ = 17.2 Hz, 1H), 1.87 – 1.71 (m, 3H), 1.69 – 1.57 (m, 1H). **$^{13}$C NMR (126 MHz, $CDCl_3$)**: $\delta$ = 175.0, 141.4, 141.1, 137.8, 137.2, 129.3, 129.0, 127.5, 127.1, 126.7, 126.5, 125.8, 125.5, 73.4, 60.7, 59.1, 48.8, 39.9, 39.6, 36.9, 30.2, 19.9. **HRMS (ESI)**: Calculated for $C_{22}H_{23}NO_2Na$ $[M+Na]^+$: 356.1621, Found: 356.1620. **Optical Rotation**: $[\alpha]_D^{25}$ = –90.85 ($c$ = 1.00, $CHCl_3$). Absolute configuration was determined through analogy to **[3o]**.
The minor diastereomer **[*epi*-3q]** was not isolated.

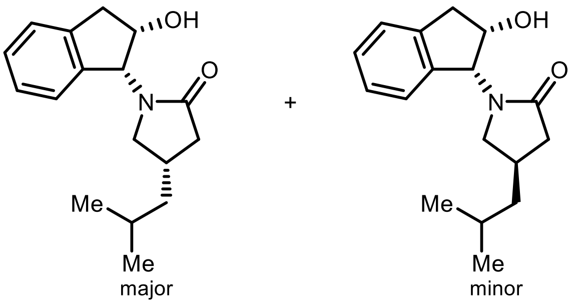


**1-((1*S*,2*R*)-2-Hydroxy-2,3-dihydro-1*H*-inden-1-yl)-4-isobutylpyrrolidin-2-one [3r, *epi*-3r]:**
Following the general procedure **D** using 3-isobutylcyclobutanone (126 mg, 1.0 mmol, 1.0 equiv.) **[1r]** and (1*R*,2*S*)-1-amino-2,3-dihydro-1*H*-inden-2-ol (149 mg, 1.0 mmol, 1.0 equiv.) the major diastereomer (169 mg, 0.62 mmol, 62%) and the minor diastereomer (35.2 mg, 13 μmol, 13%) were obtained by gradient flash column chromatography (SiO2, EtOAc, stained with $KMnO_4$) both as colorless oils.
**Major diastereomer [3r]: IR (neat)**: $\tilde{\nu}$ = 3363 (br), 2952 (m), 2869 (m), 2114 (w), 1662 (s), 1439 (m), 1293 (m), 1246 (m), 1095 (m), 866 (w), 817 (w), 740 (s). **$^1$H NMR (600 Hz, $CDCl_3$)**: $\delta$ = 7.32 − 7.22 (m, 3H), 7.20 (d, $J$ = 7.4 Hz, 1H), 5.46 (d, $J$ = 6.7 Hz, 1H), 4.79 (*app.* q, $J$ ≈ 6.9 Hz, 1H), 3.24 (dd, $J$ = 16.2, 7.4 Hz, 1H), 3.08 (dd, $J$ = 9.6, 7.9 Hz, 1H), 2.97 (dd, $J$ = 9.6, 7.9 Hz, 1H), 2.91 (dd, $J$ = 16.2, 6.7 Hz, 1H), 2.59 (dd, $J$ = 16.6, 8.4 Hz, 1H), 2.31 (*app.* sept., $J$ ≈ 8.1 Hz, 1H), 2.18 (dd, $J$ = 16.6, 9.1 Hz, 1H), 1.55 − 1.45 (m, 1H), 1.36 − 1.23 (m, 2H), 0.84 (dd, $J$ = 6.6, 4.9 Hz, 6H). **$^{13}$C NMR (151 MHz, $CDCl_3$)**: $\delta$ = 176.3, 141.3, 138.1, 129.0, 127.4, 125.8, 125.5, 73.6, 59.0, 52.0, 43.8, 40.0, 38.5, 31.1, 26.3, 22.9, 22.6. **HRMS (ESI)**: Calculated for $C_{17}H_{23}NO_2Na$ $[M+Na]^+$: 296.1621, Found: 296.1619. **Optical Rotation**: $[\alpha]_D^{20}$ = +34.4 ($c$ = 1.00, $CHCl_3$). Absolute configuration was determined through analogy to **[3a]**.
**Minor diastereomer [*epi*-3r]: IR (neat)**: $\tilde{\nu}$ = 3330 (br), 2954 (m), 2869 (m), 2243 (w), 1662 (s), 1441 (m), 1293 (m), 1197 (w), 1095 (m), 910 (w), 817 (w), 733 (s). **$^1$H NMR (600 MHz, $CDCl_3$)**: $\delta$ = 7.31 − 7.22 (m, 3H), 7.19 (d, $J$ = 7.4 Hz, 1H), 5.47 (d, $J$ = 6.7 Hz, 1H), 4.78 (*app.* q, $J$ ≈ 6.8 Hz, 1H), 3.34 (dd, $J$ = 9.6, 7.7 Hz, 1H), 3.25 (dd, $J$ = 16.3, 7.3 Hz, 1H), 2.93 (dd, $J$ = 16.3, 6.5 Hz, 1H), 2.70 (dd, $J$ = 9.6, 6.4 Hz, 1H), 2.60 (dd, $J$ = 16.7, 8.5 Hz, 1H), 2.42 (*app.* sept., $J$ ≈ 7.7 Hz, 1H), 2.15 (dd, $J$ = 16.7, 7.4 Hz, 1H), 1.52 − 1.41 (m, 1H), 1.30 − 1.16 (m, 2H), 0.84 (dd, $J$ = 10.0, 6.6 Hz, 6H). **$^{13}$C NMR (150 MHz, $CDCl_3$)**: $\delta$ = 176.3, 141.2, 138.0, 128.9, 127.3, 125.9, 125.5, 73.4, 59.0,

52.2, 43.8, 40.0, 38.3, 30.2, 26.1, 22.8, 22.6. **HRMS (ESI)**: Calculated for $C_{17}H_{23}NO_2Na$ $[M+Na]^+$: 296.1621, Found: 296.1617. **Optical Rotation**: $[\alpha]_D^{20}$ = −4.51 (*c* = 0.50, $CHCl_3$). Absolute configuration was determined through analogy to **[*epi*-3a]**.

## 7. Deprotection of hydroxyindenyl γ-lactams

## Identification of suitable conditions

*Table S2: Initial screening for deprotection of γ-lactam **[3a]**.*

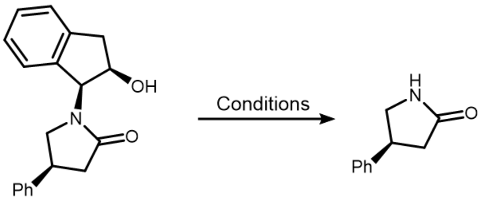


| Entry | Conditions | Result |
|---|---|---|
| 1 | Pd/C, $H_2$ (MeOH) rt, 16 h | - |
| 2 | Pd/C, $H_2$ (50 bar) (MeOH) 80 °C, 16 h | - |
| 3 | DDQ ($CH_2Cl_2$/$H_2O$) rt, 16 h | - |
| 4 | AIBN, NBS (DCB) 120 °C | decomp. |
| 5 | KOH, $O_2$ ($Et_2O$) rt, 16 | <10% |
| 6 | CAN (4 equiv.) (MeCN/$H_2O$) rt, 16 h | 30% |
| 7 | CAN (4 equiv.)(MeCN/$H_2O$) 70 °C, 4 h | 40% |

*Table S3: Optimization for deprotection of γ-lactam **[3a]**.*

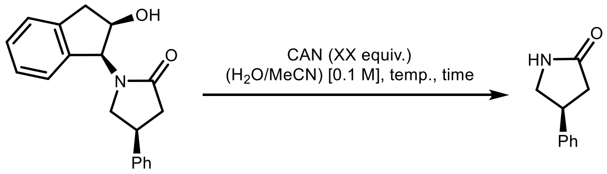


| entry | equiv. CAN | solvent ratio | temperature (°C) | time (h) | yield (%) |
|---|---|---|---|---|---|
| 1 | 3 | 3:1 (MeCN:$H_2O$) | 70 | 4 | 47 |
| 2 | 3 | 3:1 | 80 | 4 | 33 |
| 3 | 2 | 1:1 | 70 | 4 | 57 |
| 4 | 4 | 3:1 | 70 | 4 | 33 |
| 5 | 3 | 3:1 | 70 | 8 | 43 |
| 6 | 2.3 | 3:1 | 70 | 4 | 47 |
| 7 | 2.3 | 1:1 | 70 | 4 | 55 |
| 8 | 3 | methanol | 70 | 4 | -* |
| 9 | 3 | 3:1 | rt | 16 | very low** |
| 10 | 1 | 1:1 | 60 | 16 | 31 |
| 11 | 2 | 1:1 | 60 | 16 | 68 |
| 12 | 2 | 1:1, $O_2$ atm | 60 | 16 | 53 |
| 13 | 2 | 1:1, Ar atm | 60 | 16 | 66 |
| 14 | 2 (slow addition) | 1:1 | 60 | 16 | 29 |

**Studying the role of the hydroxy group for deprotection**

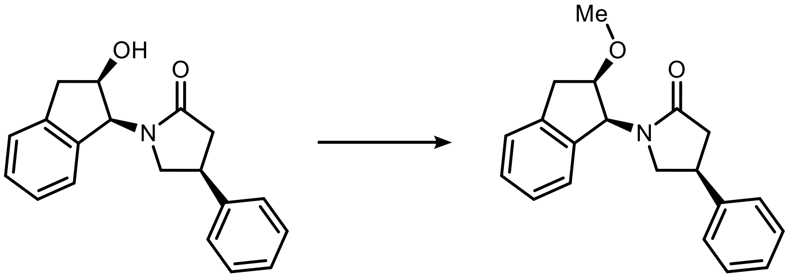

**(*S*)-1-((1*S*,2*R*)-2-methoxy-2,3-dihydro-1*H*-inden-1-yl)-4-phenylpyrrolidin-2-one [9a]**

Indanol **[3a]** (58.8 mg, 0.20 mmol, 1.0 equiv) was dissolved in DMF (5.0 mL). NaH (8.00 mg, 60% dispersion in mineral oil, 0.20 mmol, 1.0 equiv) was added followed by MeI (28.0 mg, 0.20 mmol, 1.0 equiv) and the reaction mixture stirred at room temperature for 16 h. Purification by flash column chromatography ($SiO_2$ pentane/EtOAc) gave the methyl ether **[9a]** (36 mg, 116 µmol, 58% yield) as a colorless oil.

**$^1$H NMR (500 MHz, $CDCl_3$)** $\delta$ = 7.33-7.18 (m, 9H), 5.79 (d, $J$ = 6.9 Hz, 1H), 4.28 (*app.* q, $J \approx$ 7.2 Hz, 1H), 3.47 (dd, $J$ = 9.2, 7.8 Hz, 1H), 3.44 (s, 3H), 3.41 (p, $J \approx$ 8.5 Hz, 1H), 3.28 (dd, $J$ = 9.2, 7.9 Hz, 1H), 3.20 (dd, $J$ = 16.3, 7.4 Hz, 1H), 2.93 (dd, $J$ = 16.1, 7.0 Hz, 1H), 2.89 (dd, $J$ = 16.7, 8.5 Hz, 1H), 2.66 (dd, $J$ = 16.7, 9.1 Hz, 1H). **$^{13}$C NMR (126 MHz, $CDCl_3$)** $\delta$ = 174.3, 142.3, 140.8, 138.3, 129.0, 128.8, 127.6, 127.1, 127.1, 125.8, 125.3, 81.6, 58.3, 56.2, 52.2, 39.4, 38.1, 37.8.

When **[9a]** was subjected to general protocol E (vide infra), <10% of product was detected with >90% starting material remaining.

**General procedure E: amine deprotection with cerium ammonium nitrate (CAN)**

Indanol protected γ-lactam **[3]** (1.0 equiv.) was dissolved in MeCN (0.2 M). Water (0.2 M) was added followed by cerium ammonium nitrate (CAN) (2.0 equiv.). The reaction mixture was heated to 60 °C and stirred for 16 h. The reaction was allowed to cool down to room temperature followed by addition of water (50 mL). The aqueous layer was extracted with $CH_2Cl_2$ (7 × 30 mL) and the combined organic layers were dried over $MgSO_4$, filtered and the solvent was removed under reduced pressure. The yield of the crude reaction mixture was determined by $^1$H NMR using 1,3,5-trimethylbenzene (14.0 µL, 12.3 mg, 0.10 mmol) as the internal standard.

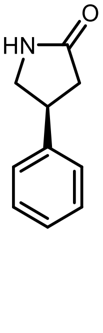

**(*S*)-4-Phenylpyrrolidin-2-one [8a]:**

Following the general procedure **E** using **[3a]** (147 mg, 0.50 mmol, 1.0 equiv.) lactam **[8a]** (49.0 mg, 0.30 mmol, 60%) was obtained by flash column chromatography ($SiO_2$, EtOAc, stained with $KMnO_4$) as a white crystalline solid. Spectroscopic data was in agreement with that previously reported.[27]
**$^1$H NMR (600 MHz, $CDCl_3$)**: $\delta$ = 7.37 – 7.32 (m, 2H), 7.30 – 7.24 (m, 3H), 6.35 (br s, 1H), 3.79 (dd, $J$ = 9.5, 8.2 Hz, 1H), 3.70 (*app.* p, $J \approx$ 8.4 Hz, 1H), 3.43 (dd, $J$ = 9.5, 7.3 Hz, 1H), 2.74 (dd, $J$ = 16.9, 8.9 Hz, 1H), 2.52 (dd, $J$ = 16.9, 8.9 Hz, 1H). **$^{13}$C NMR (151 MHz, $CDCl_3$)**: $\delta$ = 177.8, 142.2, 129.0, 127.3, 126.9, 49.7, 40.5, 38.1. **Optical Rotation**: $[\alpha]_D^{20}$ = +27.1 ($c$ = 1.00, $CHCl_3$). The enantiomeric ratio was >99:1 er as determined by HPLC analysis using a chiral column (Daicel Chiralcel OJ-H column, 20 °C, 1.0 mL/min, 95:5 hexane : isopropanol, 210 nm, $t_{minor}$ = 23.7 min, $t_{major}$ = 25.3 min). Absolute stereochemistry was determined through analogy with **[3a]**, which matched the literature known optical rotation for **[8a]**.

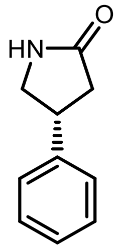


**(*R*)-4-Phenylpyrrolidin-2-one [*epi*-8a]:**
Following the general procedure **E** using the minor diastereomer (*R*)-1-((1*S*,2*R*)-2-hydroxy-2,3-dihydro-*1H*-inden-1-yl)-4-phenylpyrrolidin-2-one **[*epi*-3a]** (29.4 mg, 0.10 mol, 1.00 equiv.) lactam **[*epi*-8a]** (8.2 mg, 50 µmol, 50% yield) was isolated as a white solid.

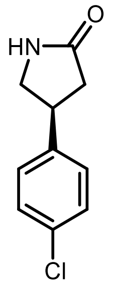


**(*S*)-4-(4-Chlorophenyl)pyrrolidin-2-one [8f]:**
Following the general procedure **E** using **[3f]** (50.0 mg, 0.15 mmol, 1.0 equiv.) lactam **[8f]** (21.0 mg, 0.11 mmol, 70%) was obtained by flash column chromatography ($SiO_2$, EtOAc, stained with $KMnO_4$) as a white solid. Spectroscopic data was in agreement with that previously reported.[28]
**$^1$H NMR (400 MHz, $CDCl_3$)**: $\delta$ = 7.34 – 7.27 (m, 2H), 7.23 – 7.14 (m, 2H), 6.83 – 6.75 (br s, 1H), 3.78 (ddd, $J$ = 9.4, 8.2, 0.8 Hz, 1H), 3.72 – 3.61 (m, 1H), 3.38 (dd, $J$ = 9.5, 7.1 Hz, 1H), 2.73 (dd, $J$ = 16.9, 8.9 Hz, 1H), 2.45 (dd, $J$ = 16.9, 8.6 Hz, 1H).**$^{13}$C NMR (101 MHz, $CDCl_3$)**: $\delta$ = 177.7, 140.7, 133.0, 129.1, 128.2, 49.6, 39.8, 38.1. **HRMS (ESI)**: Calculated for $C_{10}H_{10}NOClNa$ $[M+Na]^+$: 218.0343, Found: 218.0342. **Optical Rotation**: $[\alpha]_D^{25}$ = +41.46 ($c$ = 1.00, $CHCl_3$).

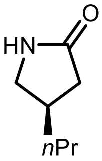


**(*R*)-4-Propylpyrrolidin-2-one [8h]:**
Following the general procedure **E** using **[3h]** (700 mg, 2.7 mmol, 1.0 equiv.) lactam **[8h]** (189 mg, 1.5 mmol, 55%) was obtained by flash column chromatography ($SiO_2$, EtOAc, stained with $KMnO_4$) as a white solid. Spectroscopic data was in agreement with that previously reported.[29]
**$^1$H NMR (400 MHz, $CDCl_3$)**: $\delta$ = 6.28 (s, 1H), 3.47 (ddd, $J$ = 9.1, 7.8, 1.1 Hz, 1H), 3.00 (dd, $J$ = 9.5, 6.7 Hz, 1H), 2.51 – 2.35 (m, 2H), 2.05 – 1.94 (m, 1H), 1.43 (q, $J$ = 7.3 Hz, 2H), 1.37 – 1.27 (m, 2H), 0.91 (t, $J$ = 7.3 Hz, 3H). **$^{13}$C NMR (101 MHz, $CDCl_3$)**: $\delta$ = 178.6, 48.2, 36.9, 36.9, 34.9, 20.8, 14.1. **Optical Rotation**: $[\alpha]_D^{25}$ = +6.39 ($c$ = 1.00, $CHCl_3$).

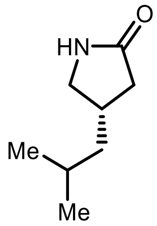


**(*S*)-4-Isobutylpyrrolidin-2-one [8r]:**
Following the general procedure **E** using **[3r]** (95.7 mg, 0.35 mmol, 1.0 equiv.) the free lactam **[8r]** (31.3 mg, 0.22 mmol, 63%) was obtained by flash column chromatography (SiO2, EtOAc, stained with $KMnO_4$) as a white solid. Spectroscopic data was in agreement with that previously reported.[30]
**$^1$H NMR (400 MHz, $CDCl_3$)**: $\delta$ = 6.16 (br s, 1H), 3.47 (dd, $J$ = 9.4, 7.8 Hz, 1H), 2.99 (dd, $J$ = 9.4, 7.2 Hz, 1H), 2.63 – 2.46 (m, 1H), 2.41 (dd, $J$ = 16.6, 8.6 Hz, 1H), 1.98 (dd, $J$ = 16.6, 8.5 Hz, 1H), 1.65 − 1.49 (m, 1H), 1.43 − 1.28 (m, 2H), 0.90 (d, $J$ = 6.6 Hz, 3H), 0.89 (d, $J$ = 6.6 Hz, 3H). **$^{13}$C NMR (101 MHz, $CDCl_3$)**: $\delta$ = 178.6, 48.4, 44.0, 37.1, 33.2, 26.3, 22.8, 22.6. **Optical Rotation**: $[\alpha]_D^{20}$ = −2.42 ($c$ = 1.00, $CHCl_3$).

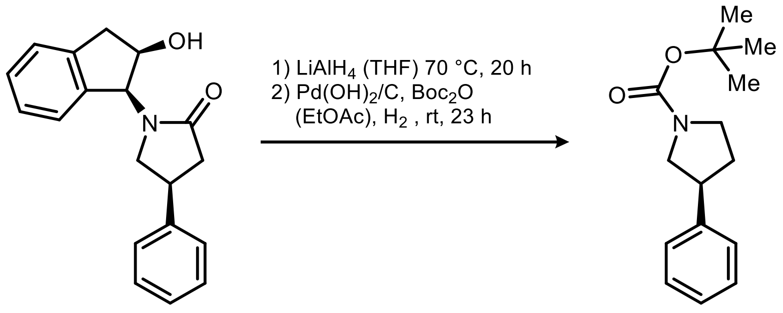


**Synthesis of (1*S*,2*R*)-1-((*S*)-3-Phenylpyrrolidin-1-yl)-2,3-dihydro-1*H*-inden-2-ol [S5]:**
$LiAlH_4$ (47.0 mg, 1.24 mmol, 2.0 equiv.) was added to a solution of protected γ-lactam **[3a]** (180 mg, 0.61 mmol, 1.00 equiv.) inTHF (2 mL). The mixture was stirred at 50 °C for 20 h. The reaction mixture was allowed to cool down to room temperature and water (5 mL) and $CH_2Cl_2$ (5 mL) were added. The layers were separated and the aqueous

phase was extracted with $CH_2Cl_2$ (3 × 5 mL). The combined organic layers were dried over $MgSO_4$, filtered and concentrated under reduced pressure to obtain the title compound **[S5]** (151 mg, 0.54 mmol, 89%) as a brown oil which was used without further purification for the next step.
**$^1$H NMR (400 MHz, $CDCl_3$)**: $\delta$ =7.40 (d, *J* = 7.3 Hz, 1H), 7.37 − 7.28 (m, 3H), 7.31 − 7.19 (m, 5H), 4.51 (*app.* q, *J* ≈ 7.4 Hz, 1H), 4.38 (d, *J* = 7.2 Hz, 1H), 3.33 (*app.* p, *J* ≈ 8.2 Hz, 1H), 3.25 (dd, *J* = 16.1, 7.6 Hz, 1H), 3.12 (*app.* t, *J* ≈ 8.6 Hz, 1H), 3.10 − 2.99 (m, 1H), 2.95 (*app.* t, *J* ≈ 8.6 Hz, 1H), 2.88 − 2.76 (m, 2H), 2.38 − 2.25 (m, 1H), 1.97 − 1.83 (m, 1H). **$^{13}$C NMR (101 MHz, $CDCl_3$)**: $\delta$ = 144.2, 142.1, 138.3, 128.8, 128.6, 127.2, 126.8, 126.4, 126.3, 125.6, 70.5, 66.6, 58.5, 50.6, 43.1, 40.7, 32.8.

**Synthesis of *tert*-Butyl (*S*)-3-phenylpyrrolidine-1-carboxylate [10a]:**
The synthesis of **[10a]** was followed by a protocol by Fukuyama *et al.*[31] Pyrrolidin **[S5]** (70.0 mg, 0.25 mmol, 1.00 equiv.) was dissolved in EtOAc and di-*tert*-butyl dicarbonate (107 mg, 0.49 mmol, 1.95 equiv.) was added. To the solution palladium hydroxide (20% on carbon, 34.4 mg, 33 µmol, 0.13 equiv.) was added and the atmosphere was changed to hydrogen gas by feeding hydrogen gas into the solution. The mixture was stirred at room temperature for 23 h. The reaction mixture was filtered through a pad of Celite® and washed with $CH_2Cl_2$. The filtrate was concentrated under reduced pressure. The yield of the crude reaction mixture was determined by $^1$H NMR using 1,3,5-trimethylbenzene (7.0 µL, 6.01 mg, 50 µmol) as the internal standard. The residue was separated by flash column chromatography ($SiO_2$, $CH_2Cl_2$ : EtOAc (19:1), stained with $KMnO_4$) provided the desired product **[10a]** (46.4 mg, 18.8 mmol, 75%) as a colorless oil. The overall yield was 67%. Spectroscopic data was in agreement with that previously reported.[32]
**$^1$H NMR (400 MHz, $CDCl_3$)**: $\delta$ = 7.37 − 7.28 (m, 2H), 7.27 − 7.18 (m, 3H), 3.86 − 3.77 (m, 1H), 3.69 − 3.54 (m, 1H), 3.46 − 3.25 (m, 3H), 2.35 − 2.20 (m, 1H), 2.04 − 1.93 (m, 1H), 1.48 (s, 9H). **$^{13}$C NMR (101 MHz, $CDCl_3$)**: $\delta$ = 154.7, 141.6, 128.7, 127.2, 126.9, 79.4, 52.4, 45.9, 44.0, 33.0, 28.7. **Optical Rotation**: $[\alpha]_D^{20}$ = -11.96 (*c* = 1.00, $CHCl_3$).

## 8. Crystallographic data

**X-ray crystal structure analysis of [3a] (9795):** A colorless needle-like specimen of $C_{19}H_{19}NO_2$, approximate dimensions 0.047 mm x 0.099 mm x 0.237 mm, was used for the X-ray crystallographic analysis. The X-ray intensity data were measured on a Bruker D8 Venture PHOTON III Diffractometer system equipped with a micro focus tube Cu Ims (CuKα, λ = 1.54178 Å) and a MX mirror monochromator. A total of 1101 frames were collected. The total exposure time was 20.90 hours. The frames were integrated with the Bruker SAINT software package using a wide-frame algorithm. The integration of the data using a monoclinic unit cell yielded a total of 24528 reflections to a maximum θ angle of 66.65° (0.84 Å resolution), of which 5463 were independent (average redundancy 4.490, completeness = 99.0%, $R_{int}$ = 9.79%, $R_{sig}$ = 7.24%) and 4199 (76.86%) were greater than 2σ($F^2$). The final cell constants of a = 6.9858(2) Å, b = 30.4324(8) Å, c = 7.3808(2) Å, β = 90.297(2)°, volume = 1569.10(7) $Å^3$, are based upon the refinement of the XYZ-centroids of 4752 reflections above 20 σ(I) with 5.808° < 2θ < 132.5°. Data were corrected for absorption effects using the Multi-Scan method (SADABS). The ratio of minimum to maximum apparent transmission was 0.872. The calculated minimum and maximum transmission coefficients (based on crystal size) are 0.8640 and 0.9710. The structure was solved and refined using the Bruker SHELXTL Software Package, using the space group $P2_1$, with Z = 4 for the formula unit, $C_{19}H_{19}NO_2$. The final anisotropic full-matrix least-squares refinement on $F^2$ with 400 variables converged at R1 = 6.63%, for the observed data and wR2 = 16.42% for all data. The goodness-of-fit was 1.076. The largest peak in the final difference electron density synthesis was 0.311 $e^-/Å^3$ and the largest hole was -0.296 $e^-/Å^3$ with an RMS deviation of 0.063 $e^-/Å^3$. On the basis of the final model, the calculated density was 1.242 $g/cm^3$ and F(000), 624 $e^-$. Flack parameter was refined to -0.0(2). CCDC Nr.: 2051970.

**X-ray crystal structure analysis of [*epi*-3a] (9714):** A colorless plate-like specimen of $C_{19}H_{19}NO_2$, approximate dimensions 0.071 mm x 0.136 mm x 0.150 mm, was used for the X-ray crystallographic analysis. The X-ray intensity data were measured. A total of 1444 frames were collected. The total exposure time was 17.65 hours. The frames were integrated with the Bruker SAINT software package using a wide-frame algorithm. The integration of the data using a monoclinic unit cell yielded a total of 11408 reflections to a maximum θ angle of 69.98° (0.82 Å resolution), of which 2852 were independent (average redundancy 4.000, completeness = 97.7%, $R_{int}$ = 2.50%, $R_{sig}$ = 2.28%) and 2839 (99.54%) were greater than 2σ($F^2$). The final cell constants of a = 10.0012(2) Å, b = 7.2606(2) Å, c = 10.6793(3) Å, β = 92.3840(10)°, volume = 774.80(3) $Å^3$, are based upon the refinement of the XYZ-centroids of 9926 reflections above 20 σ(I) with 14.75° < 2θ < 140.0°. Data were corrected for absorption effects using the multi-scan method (SADABS). The ratio of minimum to maximum apparent transmission was 0.904. The calculated minimum and maximum transmission coefficients (based on crystal size) are 0.9090 and 0.9560. The structure was solved and refined using the Bruker SHELXTL Software Package, using the space group $P2_1$, with Z = 2 for the formula unit, $C_{19}H_{19}NO_2$. The final anisotropic full-matrix least-squares refinement on $F^2$ with 203 variables converged at R1 = 2.44%, for the observed data and wR2 = 5.84% for all data. The goodness-of-fit was 1.069. The largest peak in the final difference electron density synthesis was 0.141 $e^-/Å^3$ and the largest hole was -0.115 $e^-/Å^3$ with an RMS deviation of 0.024 $e^-/Å^3$. On the basis of the final model, the calculated density was 1.257 $g/cm^3$ and F(000), 312 $e^-$. Flack

parameter was refined to 0.05(5). Hydrogen at O2 atom was refined freely. CCDC Nr.: 2051971.

**X-ray crystal structure analysis of [4a] (wie9857):** A colorless needle-like specimen of $C_{19}H_{19}NO$, approximate dimensions 0.038 mm x 0.039 mm x 0.100 mm, was used for the X-ray crystallographic analysis. The X-ray intensity data were measured (λ = 1.54178 Å). A total of 1028 frames were collected. The total exposure time was 13.84 hours. The frames were integrated with the Bruker SAINT software package using a wide-frame algorithm. The integration of the data using a monoclinic unit cell yielded a total of 22479 reflections to a maximum θ angle of 66.57° (0.84 Å resolution), of which 5146 were independent (average redundancy 4.368, completeness = 100.0%, $R_{int}$ = 15.96%, $R_{sig}$ = 10.28%) and 3191 (62.01%) were greater than $2\sigma(F^2)$. The final cell constants of a = 11.9997(4) Å, b = 8.8730(3) Å, c = 14.0416(4) Å, β = 103.429(2)°, volume = 1454.18(8) $Å^3$, are based upon the refinement of the XYZ-centroids of 1963 reflections above 20 σ(I) with 7.574° < 2θ < 125.2°. Data were corrected for absorption effects using the Multi-Scan method (SADABS). The ratio of minimum to maximum apparent transmission was 0.900. The calculated minimum and maximum transmission coefficients (based on crystal size) are 0.9420 and 0.9770. The structure was solved and refined using the Bruker SHELXTL Software Package, using the space group $P2_1$, with Z = 4 for the formula unit, $C_{19}H_{19}NO$. The final anisotropic full-matrix least-squares refinement on $F^2$ with 468 variables converged at R1 = 6.33%, for the observed data and wR2 = 18.41% for all data. The goodness-of-fit was 0.983. The largest peak in the final difference electron density synthesis was 0.331 $e^-/Å^3$ and the largest hole was -0.322 $e^-/Å^3$ with an RMS deviation of 0.087 $e^-/Å^3$. On the basis of the final model, the calculated density was 1.267 $g/cm^3$ and F(000), 592 $e^-$. Hydrogens at N1A and N1B atoms were refined freely, but with N-H distance restraints (SADI). Flack parameter was refined to 0.3(4). CCDC Nr.: 2051972.

**X-ray crystal structure analysis of [3o] (wie9904):** A colorless prism-like specimen of $C_{20}H_{21}NO_2$, approximate dimensions 0.075 mm x 0.107 mm x 0.184 mm, was used for the X-ray crystallographic analysis. The X-ray intensity data were measured on a Bruker D8 Venture PHOTON III Diffractometer system equipped with a micro focus tube Cu Ims ($CuK_\alpha$, λ = 1.54178 Å) and a MX mirror monochromator. A total of 1337 frames were collected. The total exposure time was 16.84 hours. The frames were integrated with the Bruker SAINT software package using a wide-frame algorithm. The integration of the data using a monoclinic unit cell yielded a total of 12439 reflections to a maximum θ angle of 68.28° (0.83 Å resolution), of which 2802 were independent (average redundancy 4.439, completeness = 97.5%, $R_{int}$ = 2.95%, $R_{sig}$ = 2.48%) and 2748 (98.07%) were greater than $2\sigma(F^2)$. The final cell constants of a = 10.1767(3) Å, b = 7.5326(2) Å, c = 10.4476(3) Å, β = 93.7010(10)°, volume = 799.21(4) $Å^3$, are based upon the refinement of the XYZ-centroids of 9896 reflections above 20 σ(I) with 11.76° < 2θ < 136.5°. Data were corrected for absorption effects using the Multi-Scan method (SADABS). The ratio of minimum to maximum apparent transmission was 0.906. The calculated minimum and maximum transmission coefficients (based on crystal size) are 0.8900 and 0.9530. The structure was solved and refined using the Bruker SHELXTL Software Package, using the space group $P2_1$, with Z = 2 for the formula unit, $C_{20}H_{21}NO_2$. The final anisotropic full-matrix least-squares refinement on $F^2$ with 213 variables converged at R1 = 2.74%, for the observed data and wR2 = 6.52% for all data. The goodness-of-fit was 1.052. The largest peak in the final difference electron density synthesis was 0.380 $e^-/Å^3$ and the largest hole was -0.122 $e^-/Å^3$ with an RMS deviation of 0.028 $e^-/Å^3$. On the basis of the final model, the calculated density was

1.277 g/cm$^3$ and F(000), 328 e$^-$. Hydrogen at O1 atom was refined freely. Flack parameter was refined to -0.10(7). CCDC Nr.: 2051973.

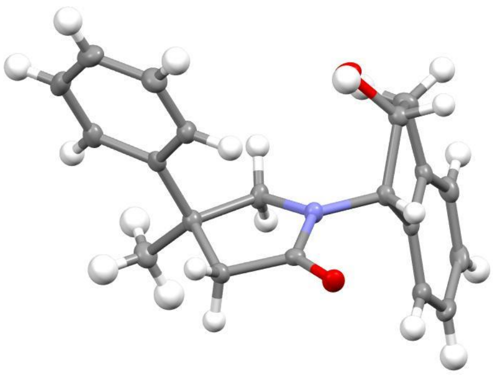

## 9. References


1 *APEX3* (**2016**), *SAINT* (**2015**) and *SADABS* (**2015**), Bruker AXS Inc., Madison, Wisconsin, USA.

2 Sheldrick, G. M., *SHELXT – Integrated space-group and crystal-structure determination*, *Acta Cryst.,* **2015**, *A71*, 3-8.

3 Sheldrick, G.M., *Crystal structure refinement with SHELXL*, *Acta Cryst.*, **2015**, *C71 (1)*, 3-8.

4 *XP – Interactive molecular graphics, Version 5.1,* Bruker AXS Inc., Madison, Wisconsin, USA, **1998**.

5 TURBOMOLE V7.4 2019, a development of University of Karlsruhe and Forschungszentrum Karlsruhe GmbH, 1989-2007, TURBOMOLE GmbH, since 2007; available from http://www.turbomole.com

6 J. Tao, J. P. Perdew, V. N. Staroverov and G. E. Scuseria, *Phys. Rev. Lett.*, **2003**, *91*, 146401.

7 a) S. Grimme, J. Antony, S. Ehrlich, H. Krieg, *J. Chem. Phys.* **2010**, *132*, 154104. b) S. Grimme, S. Ehrlich, L. Goerigk, *J. Comput. Chem.* **2011**, *32*, 1456–1465.

8 F. Weigend; R. Ahlrichs. *Phys. Chem. Chem. Phys.* **2005**, *7*, 3297–3305.

9 S. Grimme, *Chem. Eur. J.* **2012**, *18*, 9955-9964.

10 Y. Zhao, D. G. Truhlar, *J. Phys. Chem. A* **2005**, *109*, 5656-5667.

11 a) A. Klamt, *J. Phys. Chem.* **1995**, *99*, 2224-2235. b) F. Eckert and A. Klamt, COSMO*therm*, Version C3.0; COSMO*logic* GmbH & Co. KG, Leverkusen, Germany, **2013**.

12 a) A. D. Becke, J. Chem. Phys. **1993**, *98*, 5648-5652. b) P. J. Stephens, F. J. Devlin, C. F. Chabalowski, M. J. Frisch, J. Phys. Chem. **1994**, *98*, 11623-11627.

13 S. Serra, *Tetrahedron: Asymmetry*, **2011**, *22*, 619-628.

14 T. W. Liwosz, S. R. Chemler, *Chem. Eur. J.*, **2013**, *19*, 12771-12777.

15 A. V. Chernykh, D. S. Radchenko, A. V. Chernykh, I. S. Kondratov, N. A. Tolmachova, O. P. Datsenko, M. A. Kurkunov, S. X. Zozulya, Y. P. Kheylik, K. Bartels, C. G. Daniliuc, G. Haufe, *Eur. J. Org. Chem.*, **2015**, 6466–6471.

16 A. V. Malkov, F. Friscourt, M. Bell, M. E. Swarbrick, P. Kočovský, *J. Org. Chem.*, **2008**, *73*, 3996–4003.

17 J. Guo, X. Xu, Q. Xing, Z. Gao, J. Gou, B. Yu, *Org. Lett.*, **2018**, *20*, 7410-7414.

18 S. J., Brickner, J. M. Chen, Z. B. Li, A. Marfat, M. J. Mitton-Fry, M. A. Plotkin, U. D. Reilly, C. Subramanyam, Z. Zhang, S. Robinson, Substituted heterocyclic derivatives as antimicrobial agents and their preparation, pharmaceutical compositions and use in the treatment of bacterial infection. US2008/0280879 A1, November 13rd, 2008.

19 A. Drożdż, M. B. Foreiter, A. Chrobok, *Synlett*, **2014**, *25*, 559-563.

20 Z. Du, M. J. Haglund, L. A. Pratt, K. L. Erickson, *J. Org. Chem.* **1998**, *63*, 8880-8887.

21 S. Baj, R. Słupska,A. Chrobok; A. Drożdż, *J. Mol. Catal. A: Chem.*, **2013**, *376*, 120–126.

22 H.-J. Xu, F.-F. Zhu, Y.-Y. Shen, X. Wan, Y.-S. Feng, *Tetrahedron*, **2012**, *68*, 4145–4151.

23 T. T. Wager, et al., *J. Med. Chem.* **2011**, *54*, 7602–7620.

24 A. Bashir-Hashemi, J. R. Hardee, N. Gelber, L. Qi, T. Axenrod, *J. Org. Chem.*, **1994**, *59*, 2132–2134.

[25] B.D. W. Allen, M. D. Hareram, A. C. Seastram, T. McBride, T. Wirth, D. L. Browne, L. C. Morrill, *Org. Lett.*, **2019**, *21*, , 9241-9246.
[26] T. Matsuda, I. Yuihara, *Chem. Commun.*, **2015**, *51*, 7393-7396
[27] I. Fujimori, T. Mita, K. Maki, M. Shiro, A. Sato, S. Furusho, M. Kanai, M. Shibasaki, *Tetrahedron*, **2007**, *63*, 5820-5831.
[28] K. L. Jensen, P. H. Poulsen, B. S. Donslund, F. Morana, K. A. Jørgensen, *Org. Lett.*, **2012,** *14*, 1516-1519.
[29] C. S. Fuchs, J. E. Farnberger, G. Steinkellner, J. H. Sattler, M. Pickl, R. C. Simon, F. Zepeck, K. Gruber, W. Kroutil, *Adv. Synth. Catal.*, **2018**, *360*, 768.
[30] J.-m. Liu, X. Wang, Z.-m. Ge, Q. Sun, T.-m. Cheng, R.-t. Li, *Tetrahedron*, **2011**, *67*, 636-640.
[31] N. Shimada, Y. Abe, S. Yokoshima, T. Fukuyama, *Angew. Chem. Int. Ed.* **2012**, *51*,11824-11826.
[32] D. Kim, G. S. Lee, D. Kim, S. H. Hong, *Nat. Commun.*, **2020**, *11*, 5266.

## 10. NMR spectra, HPLC traces, and cartesian coordinates

**[S1]**, $^{1}H$, $CDCl_3$, 400 MHz

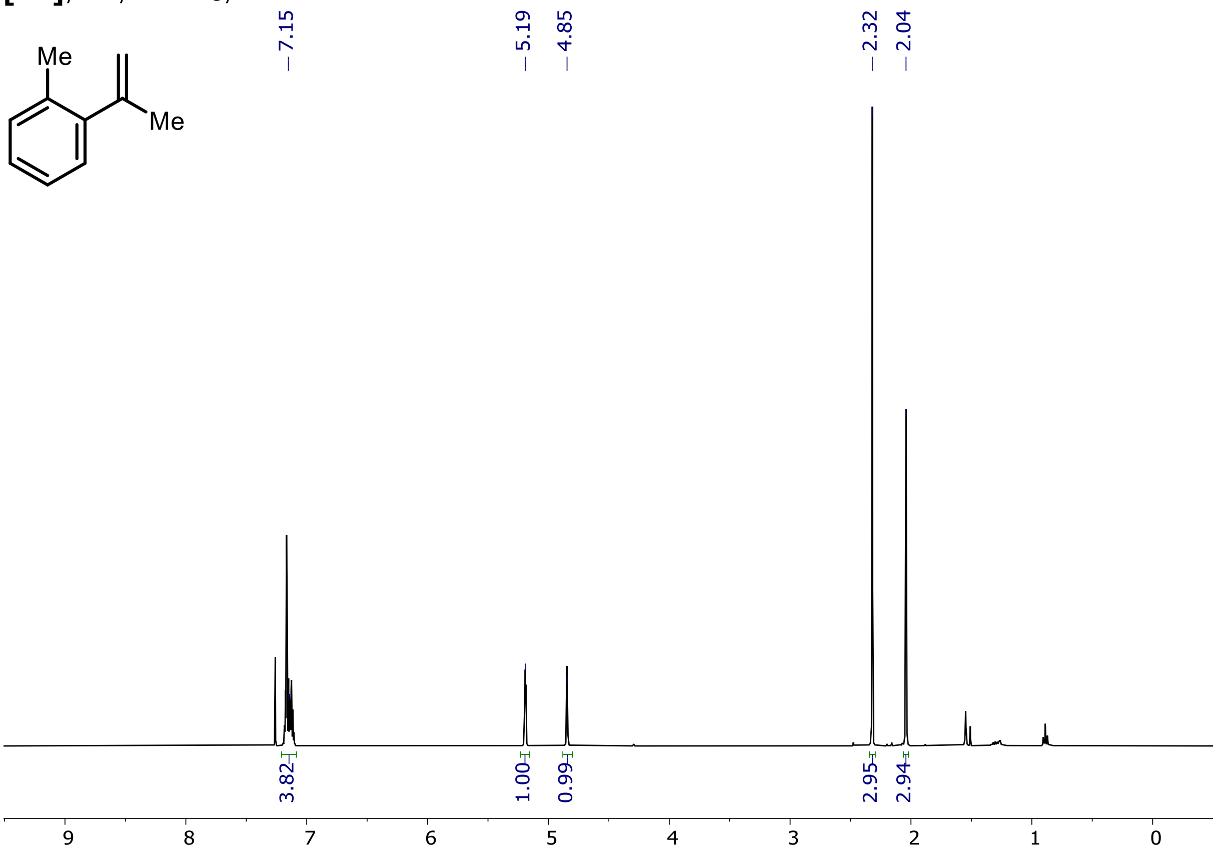


**[S1]**, $^{13}C$, $CDCl_3$, 101 MHz

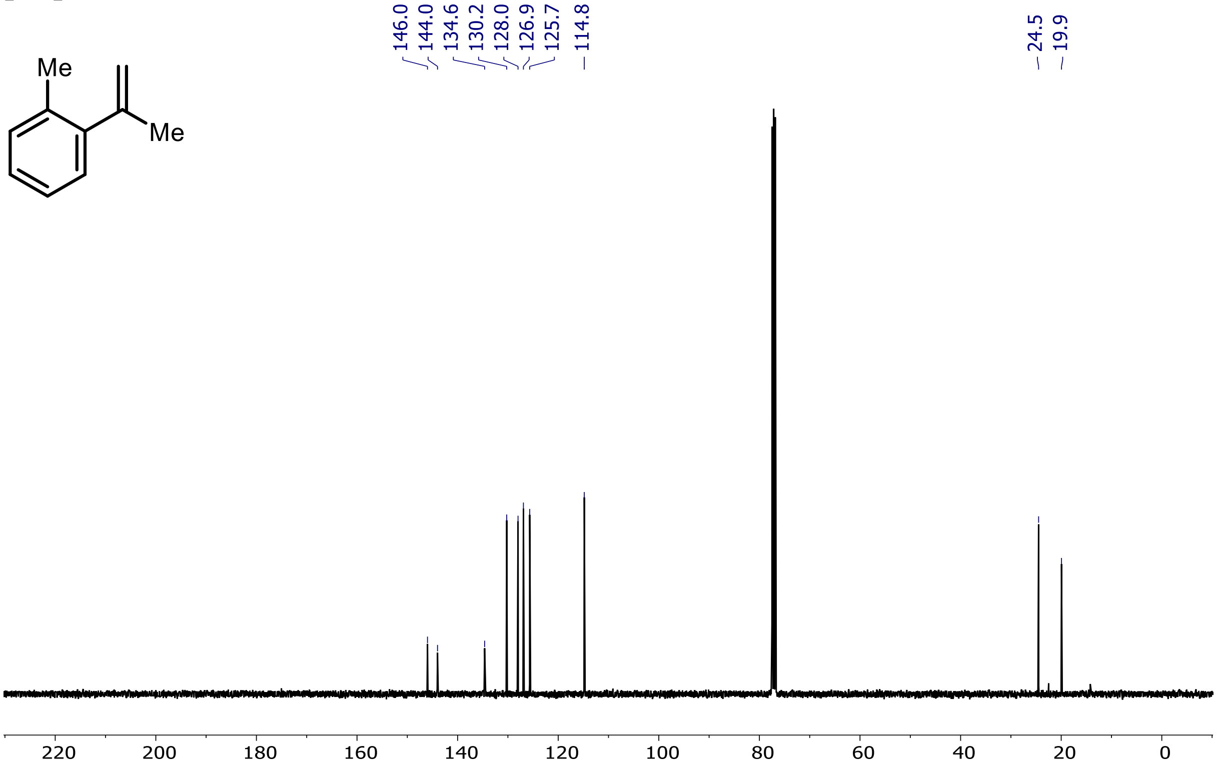

**[S2]**, $^{1}H$ NMR, $C_6D_6$, 400 MHz

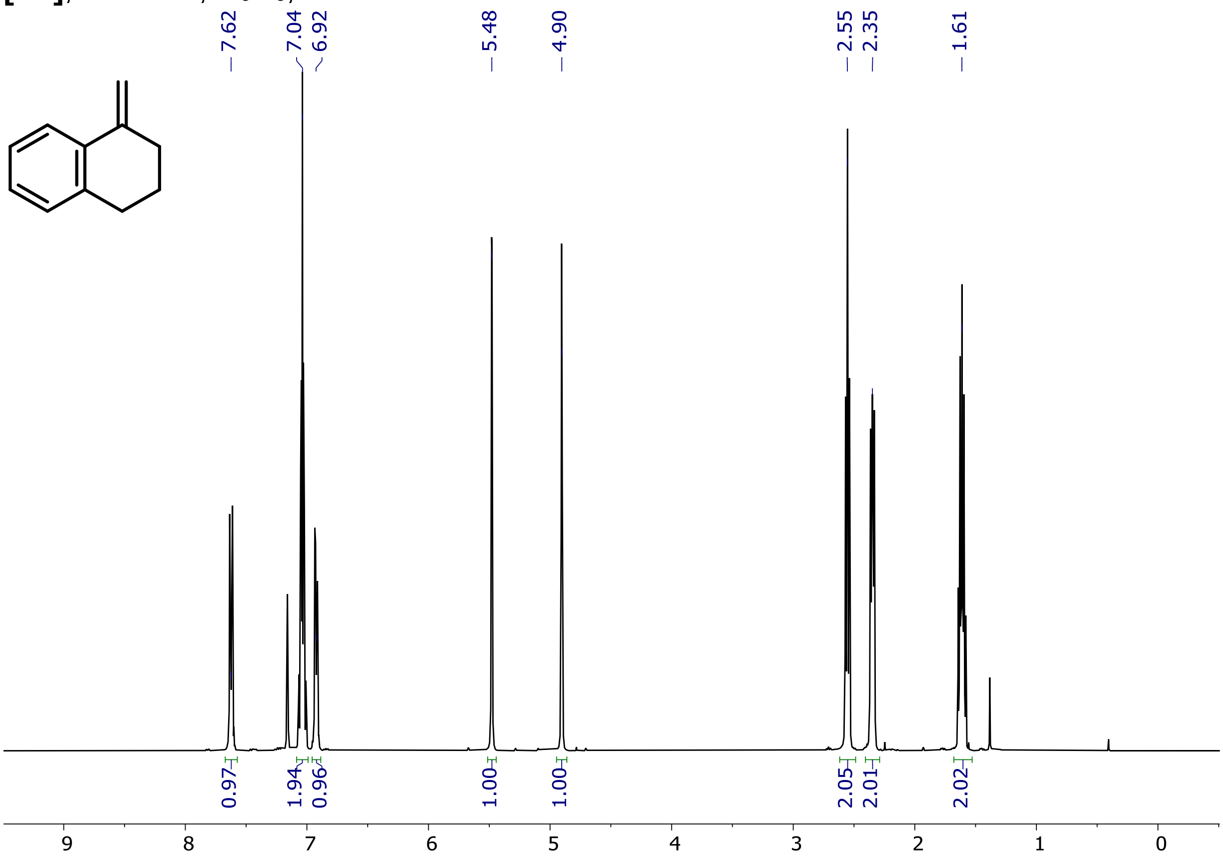


**[S2]**, $^{13}C$ NMR, $C_6D_6$, 101 MHz

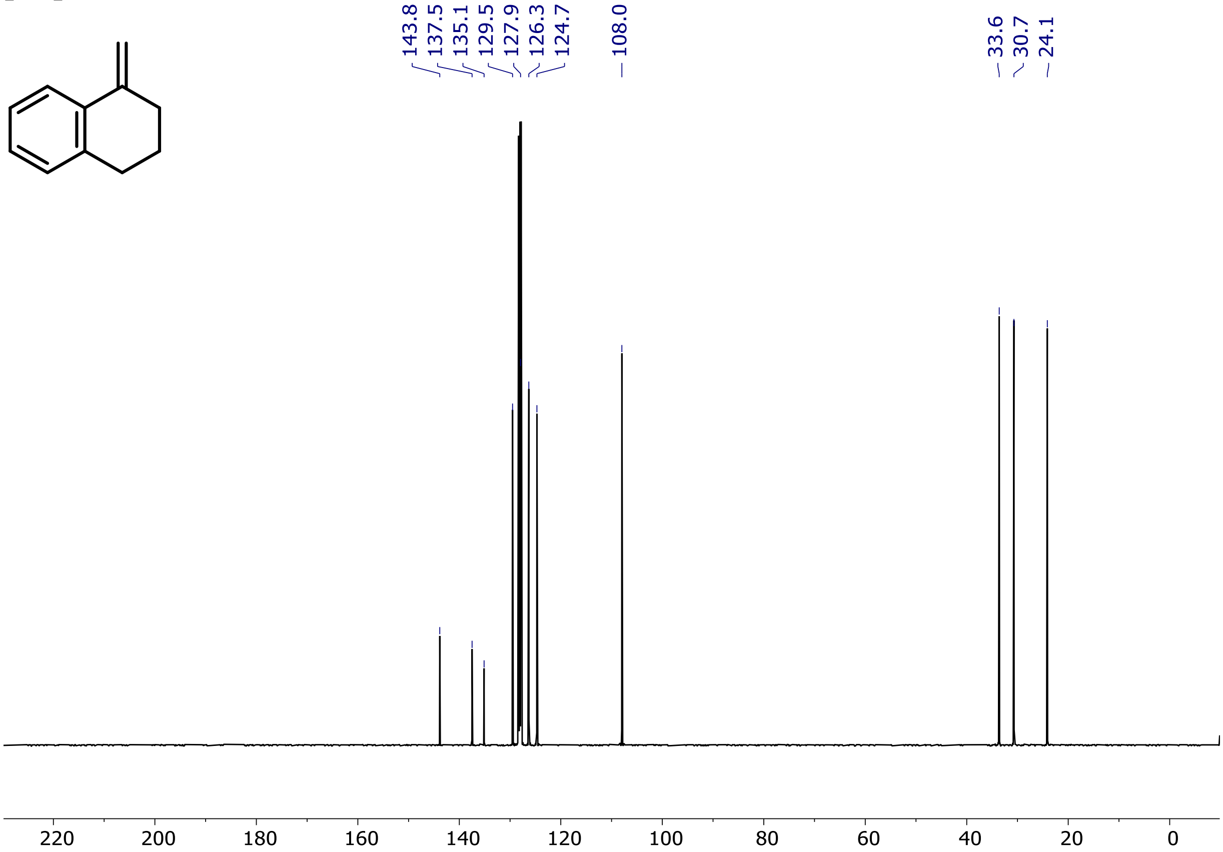

**[1a]**, $^{1}H$ NMR, $CDCl_3$, 400 MHz

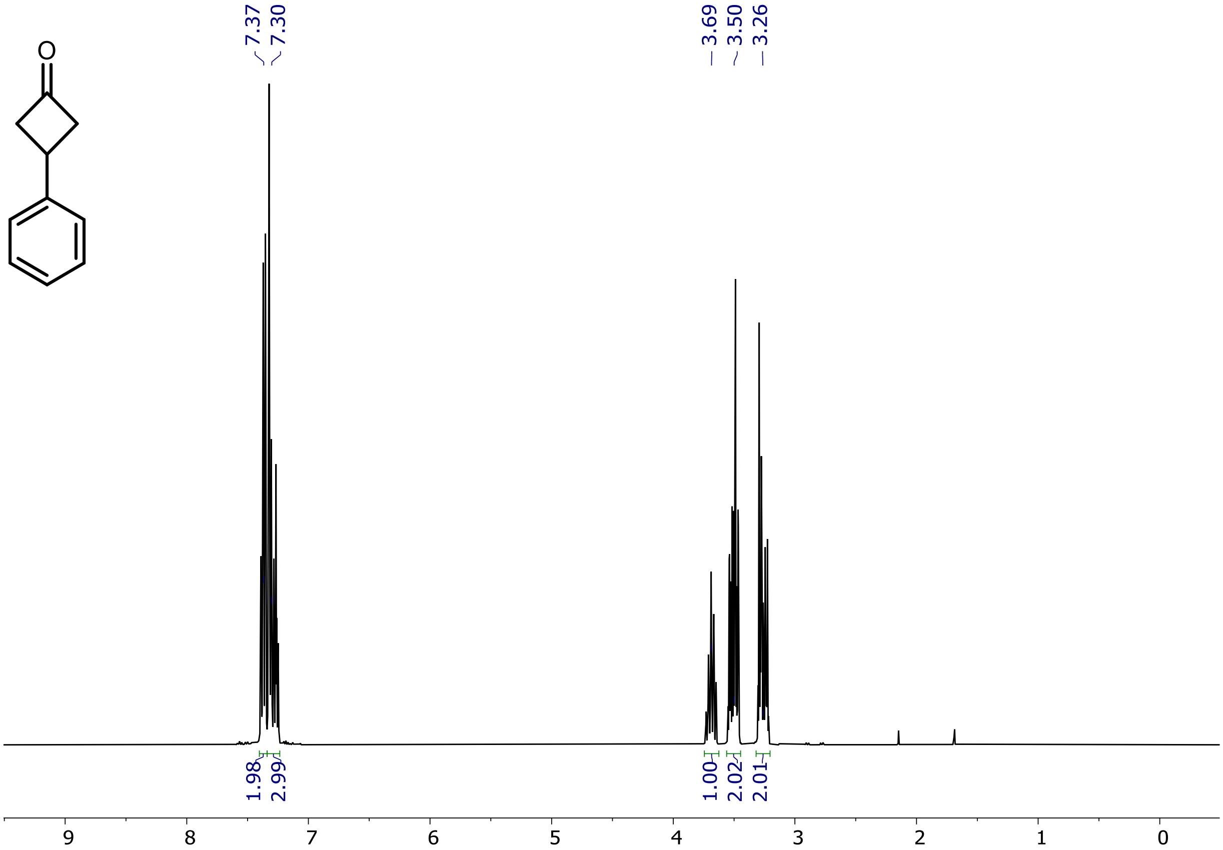


**[1a]**, $^{13}C$ NMR, $CDCl_3$, 101 MHz

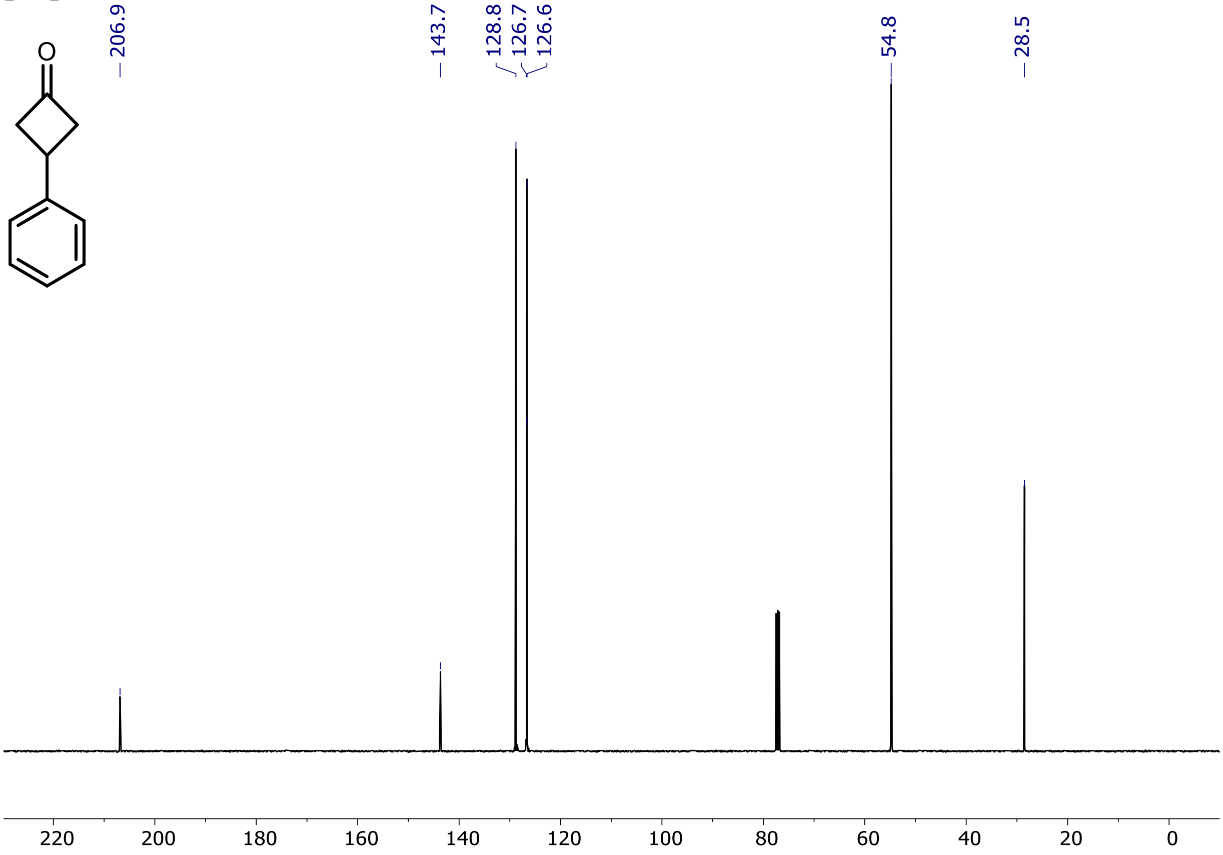

**[1b]**, $^{1}H$ NMR, $CDCl_3$, 400 MHz

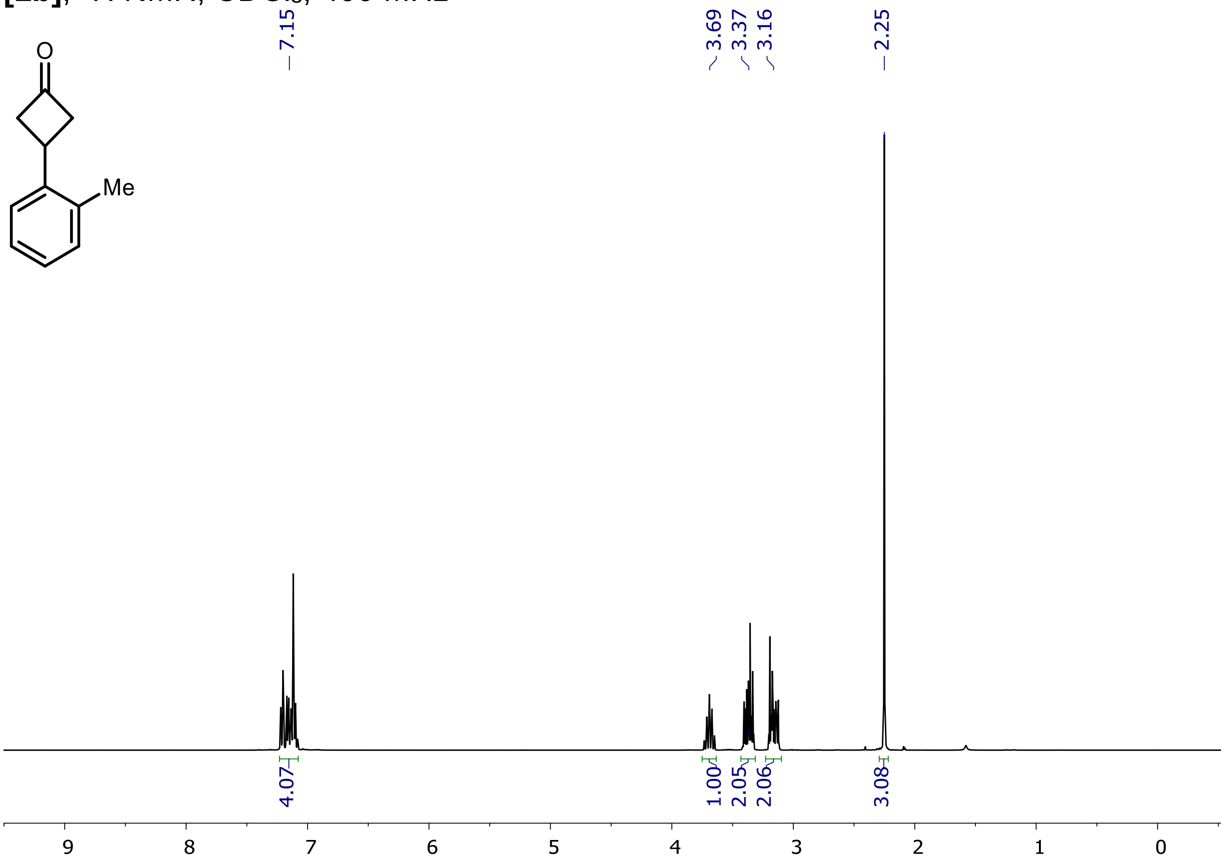


**[1b]**, $^{13}C$ NMR, $CDCl_3$, 101 MHz

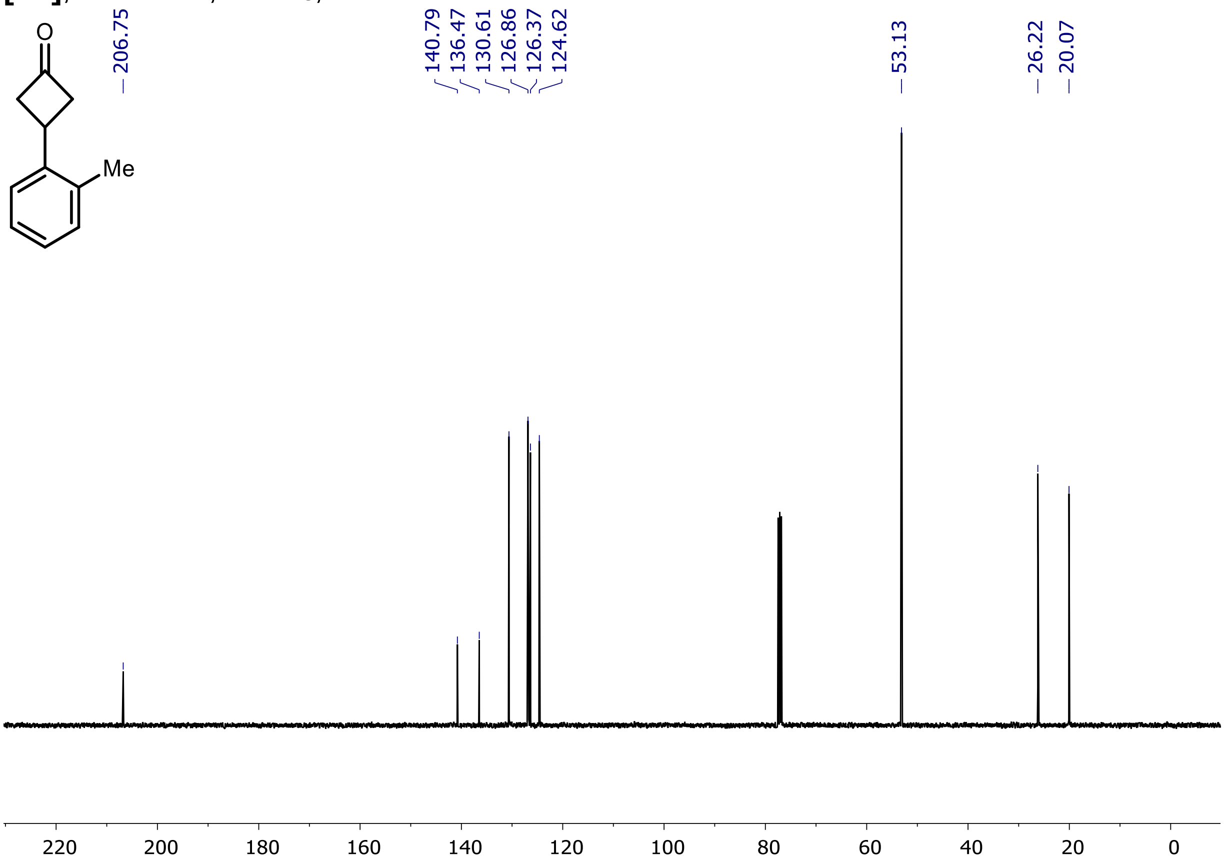

[1c], $^{1}H$ NMR, $CDCl_3$, 400 MHz

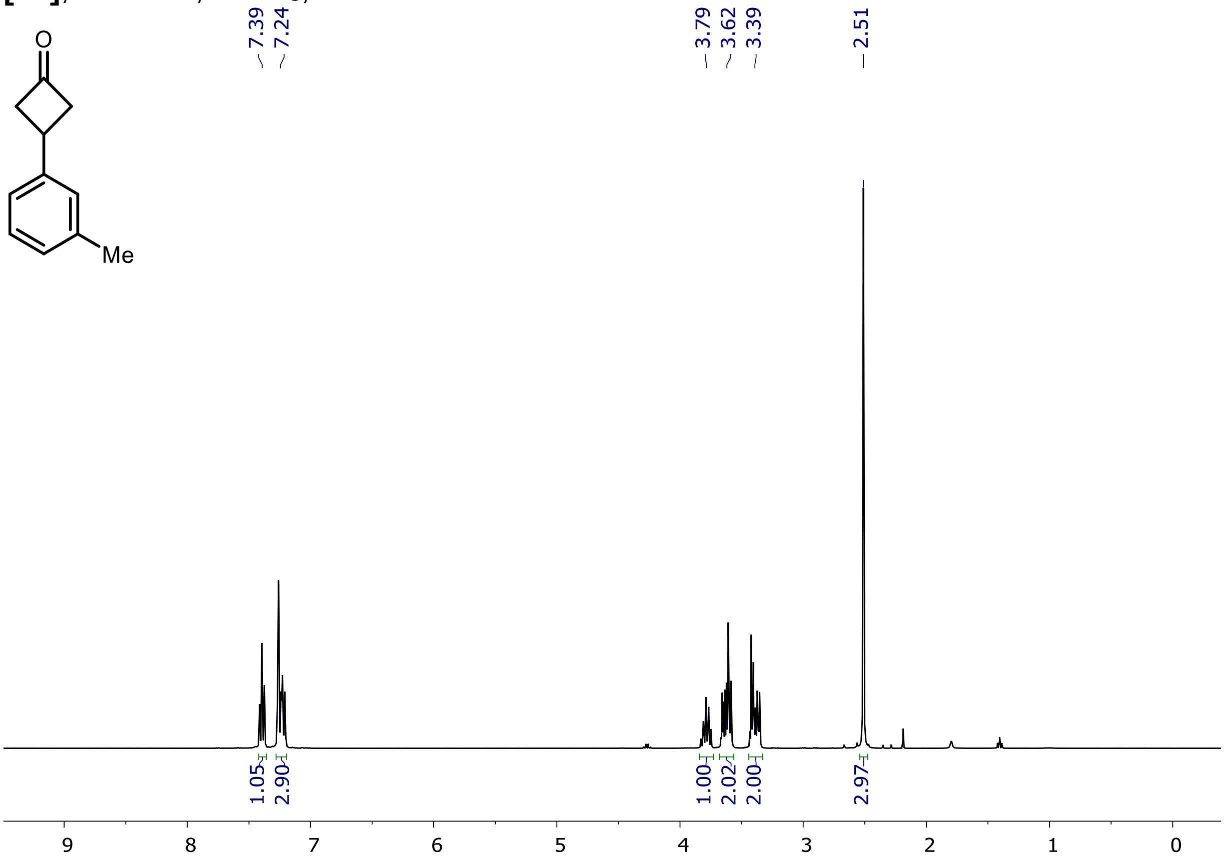


[1c], $^{13}C$ NMR, $CDCl_3$, 101 MHz

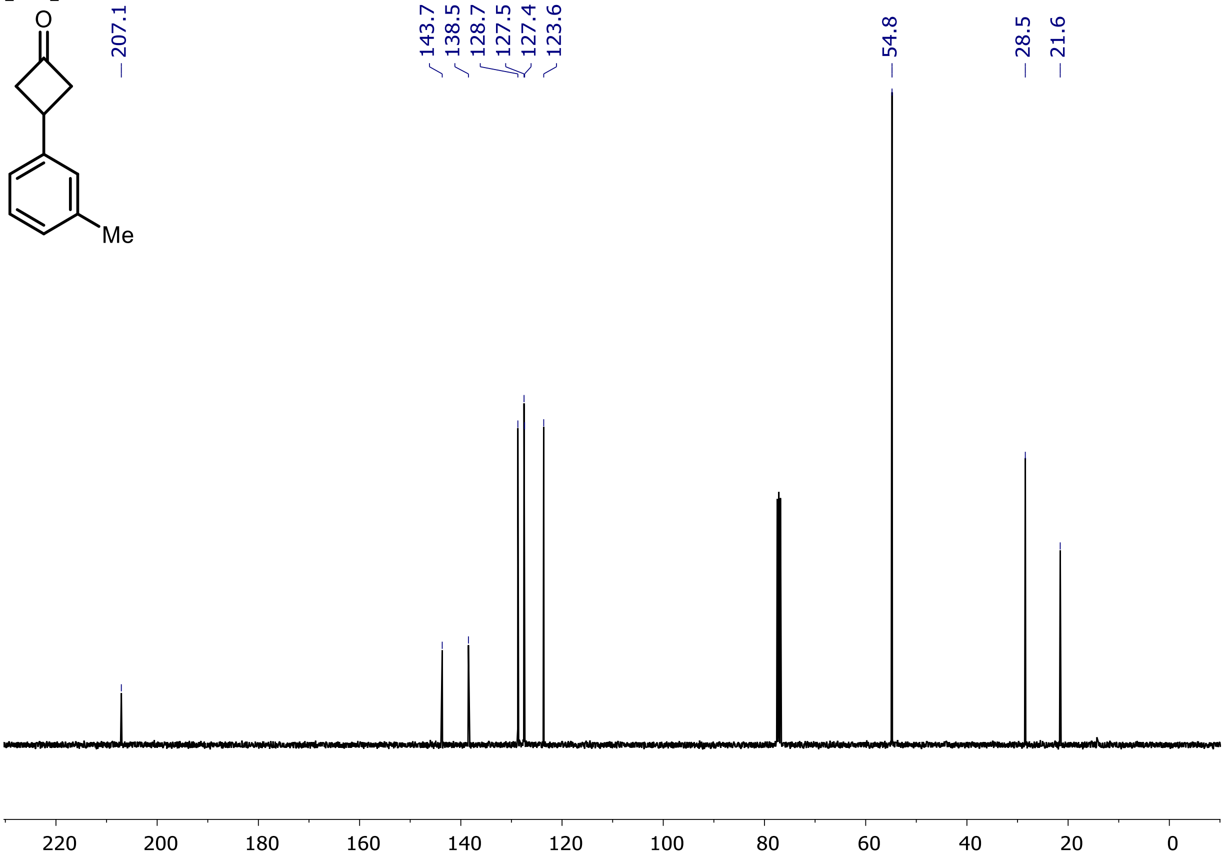

**[1d]**, $^{1}H$ NMR, $CDCl_3$, 400 MHz

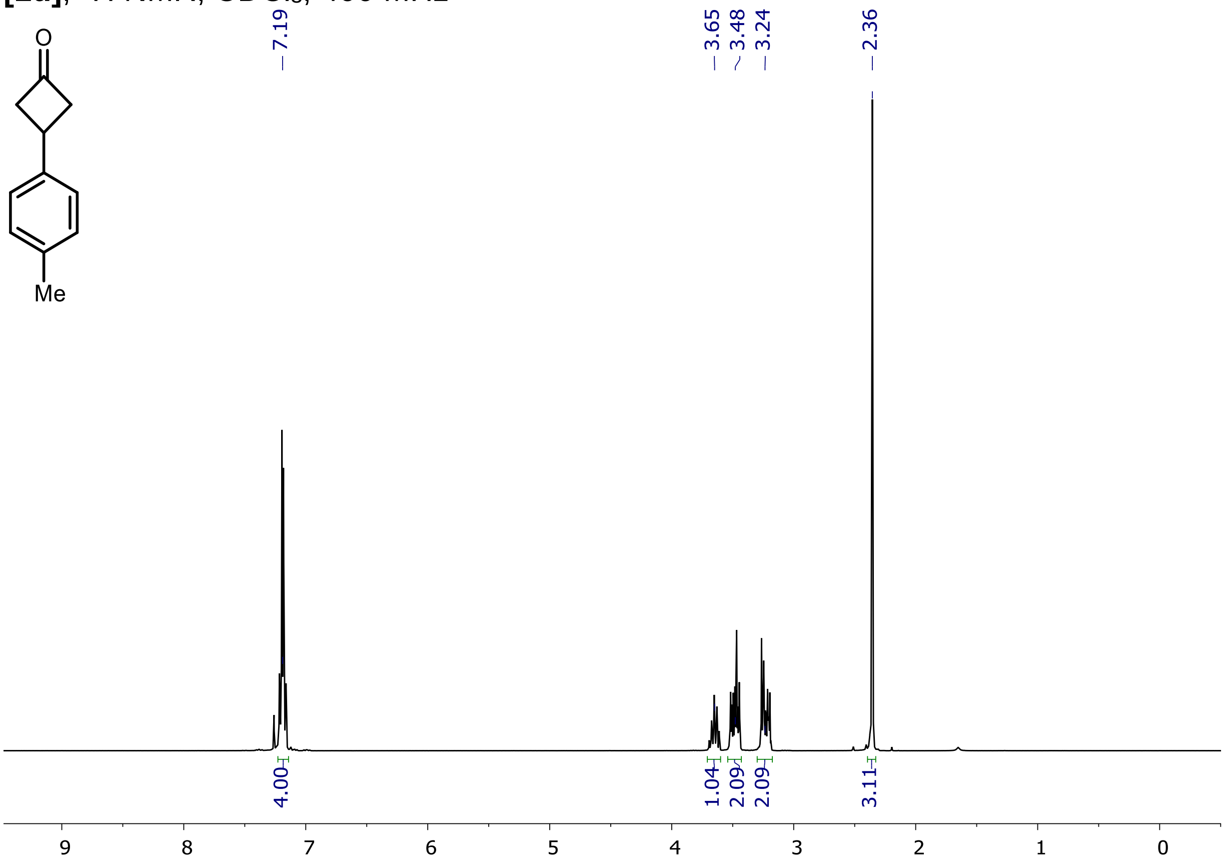


**[1d]**, $^{13}C$ NMR, $CDCl_3$, 101 MHz

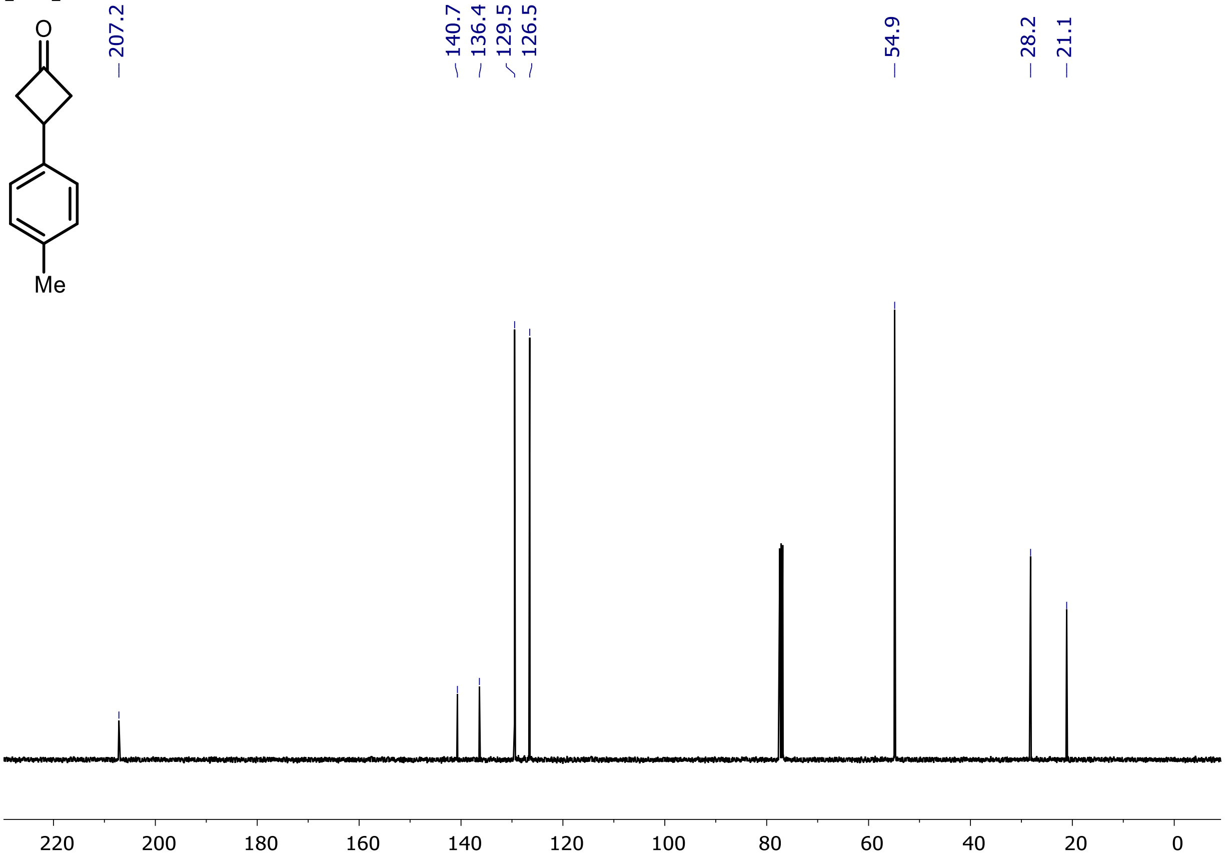

**[1e]**, $^{1}H$ NMR, $CDCl_3$, 400 MHz

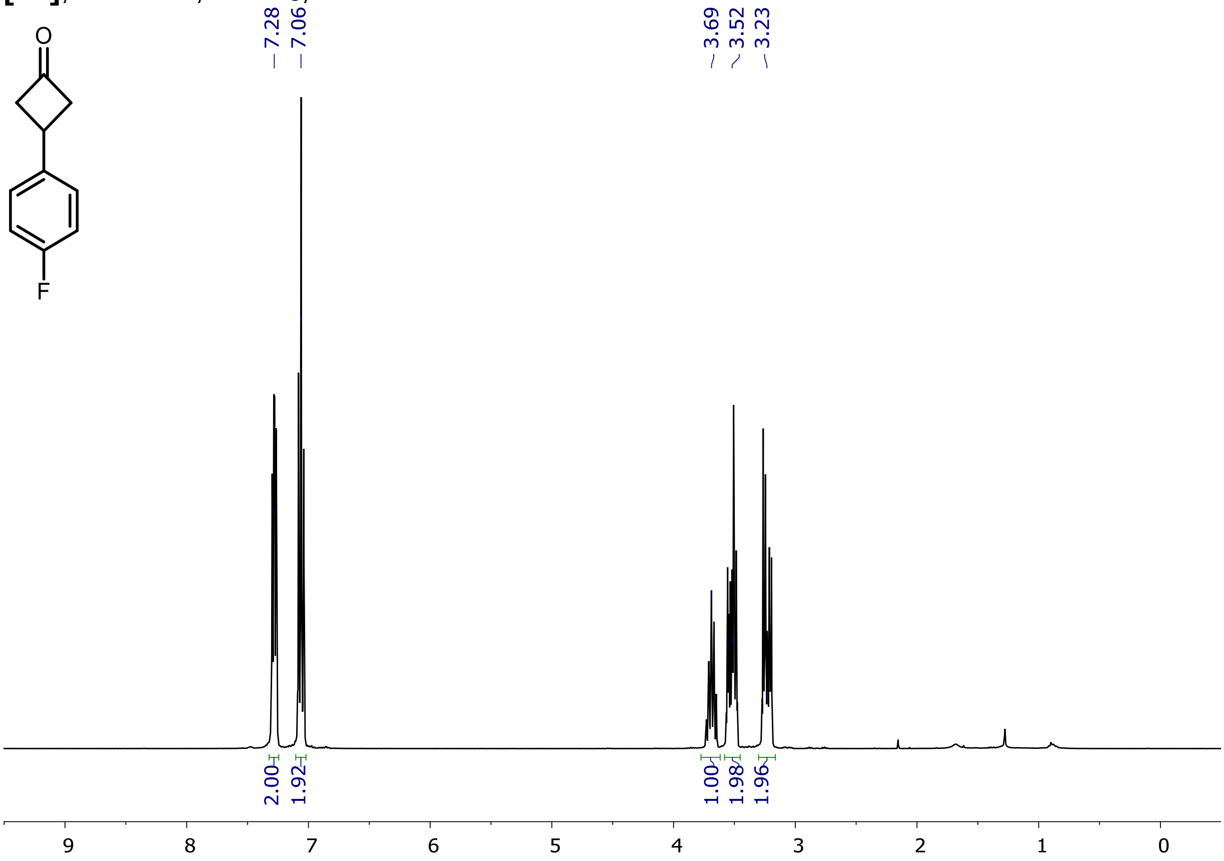


**[1e]**, $^{13}C$ NMR, $CDCl_3$, 101 MHz

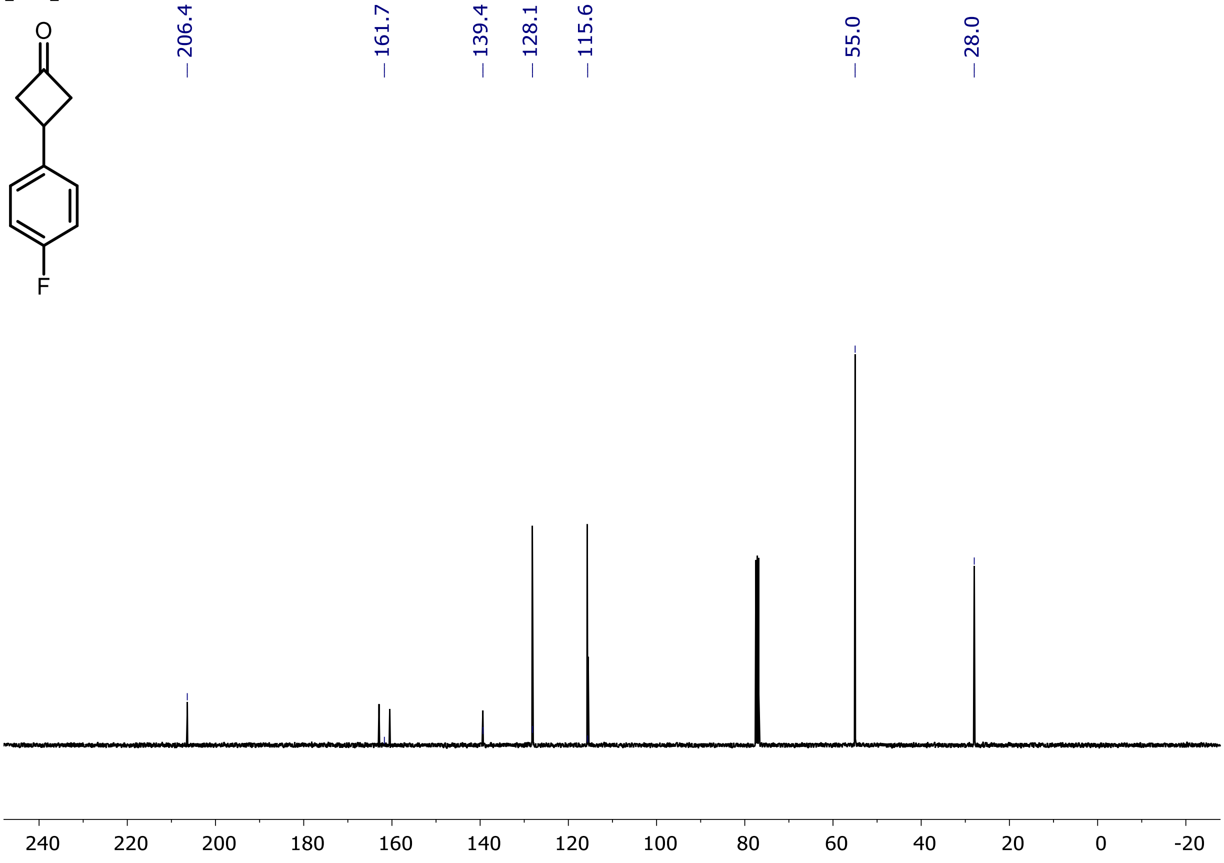

**[1f]**, $^{1}H$ NMR, $CDCl_3$, 400 MHz

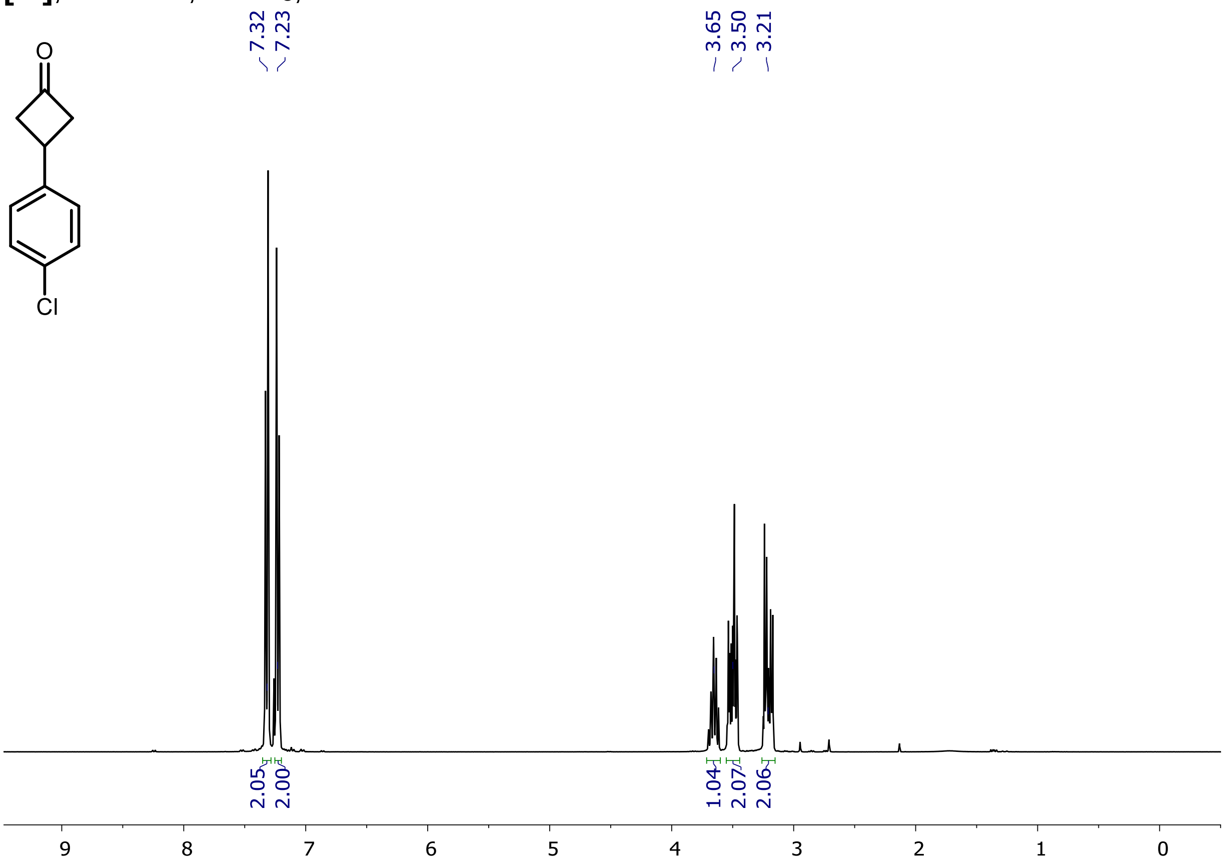


**[1f]**, $^{13}C$ NMR, $CDCl_3$, 101 MHz

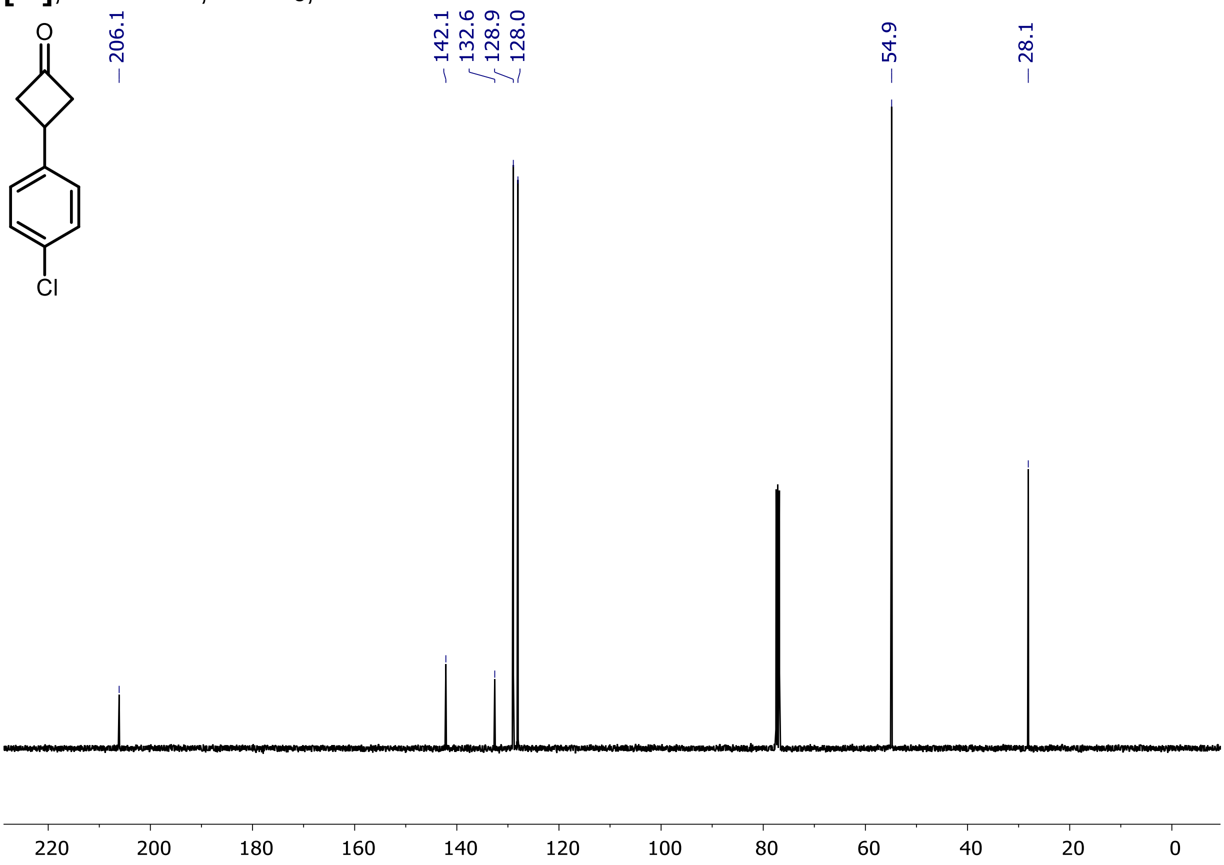

**[1g]**, $^{1}H$ NMR, $CDCl_3$, 400 MHz

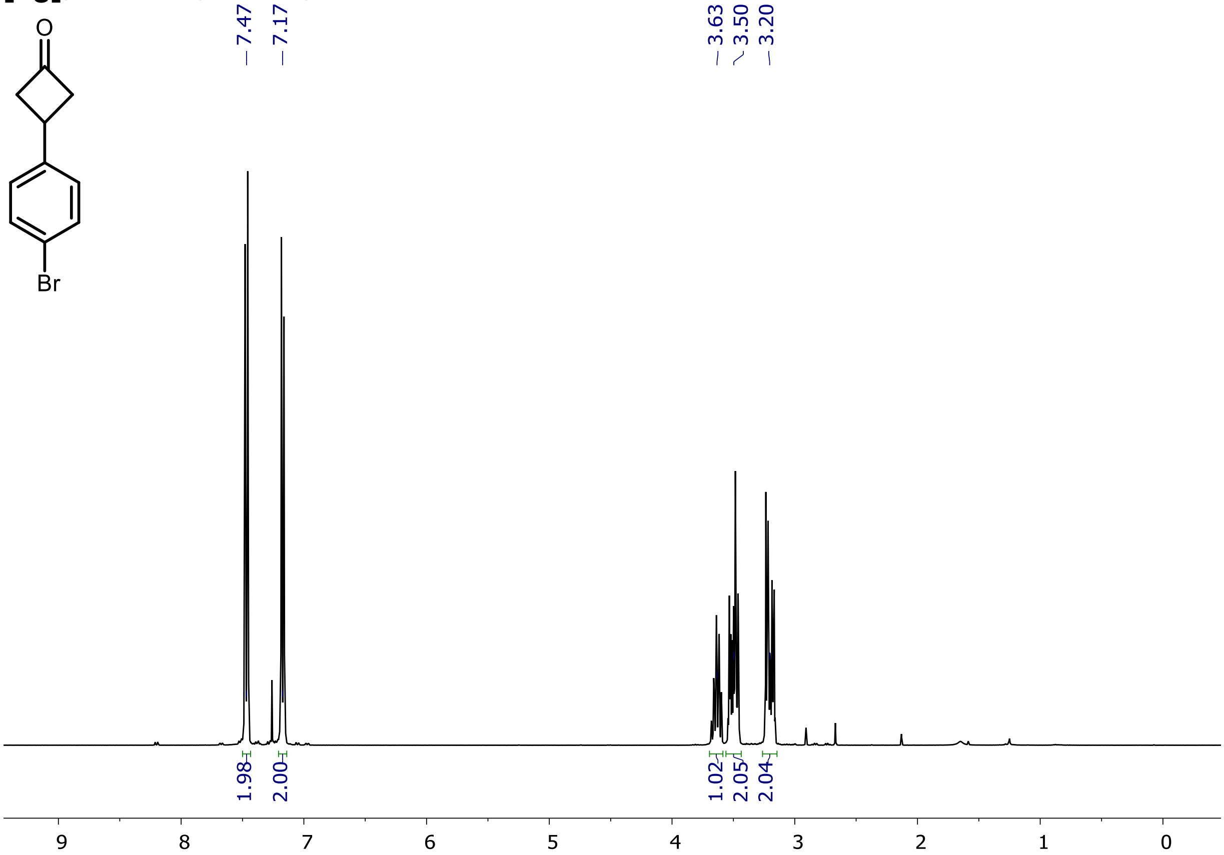


**[1g]**, $^{13}C$ NMR, $CDCl_3$, 101 MHz

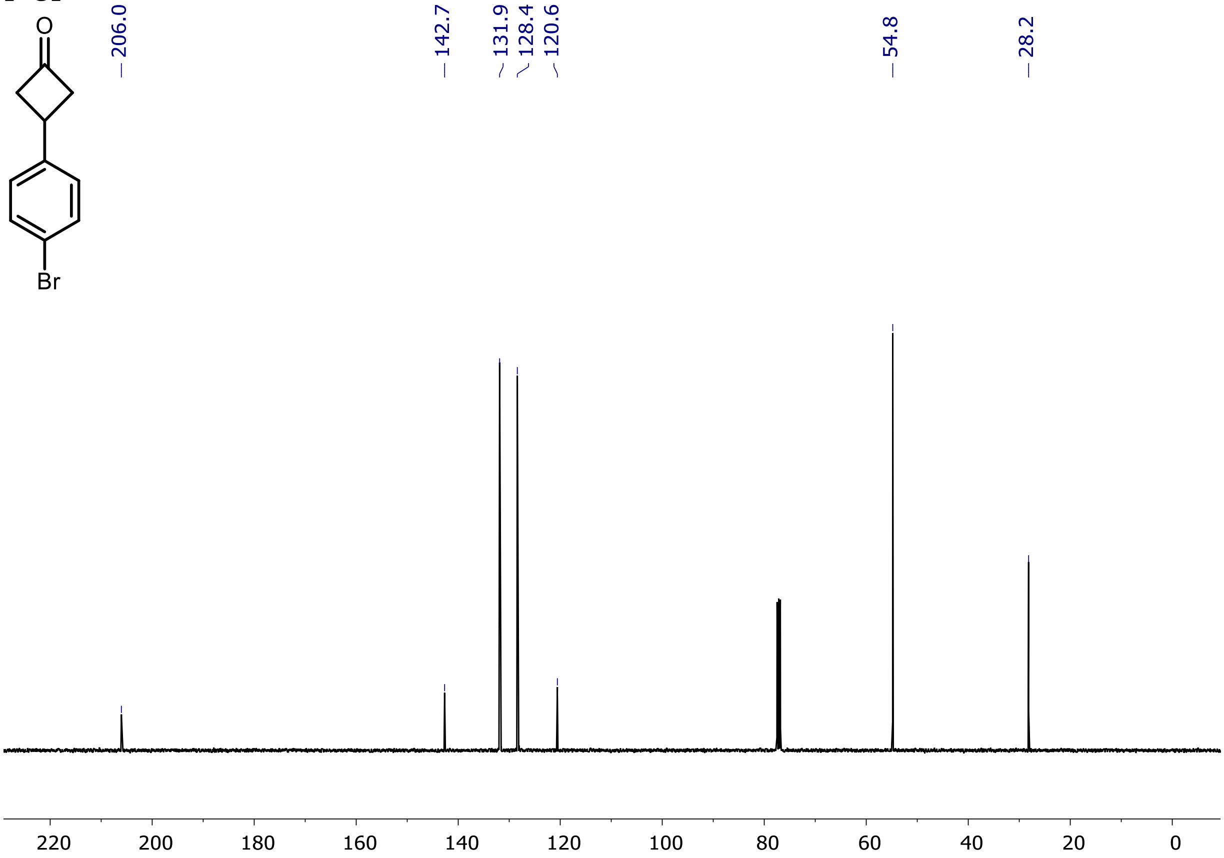

**[1h]**, $^1$H NMR, $CDCl_3$, 500 MHz

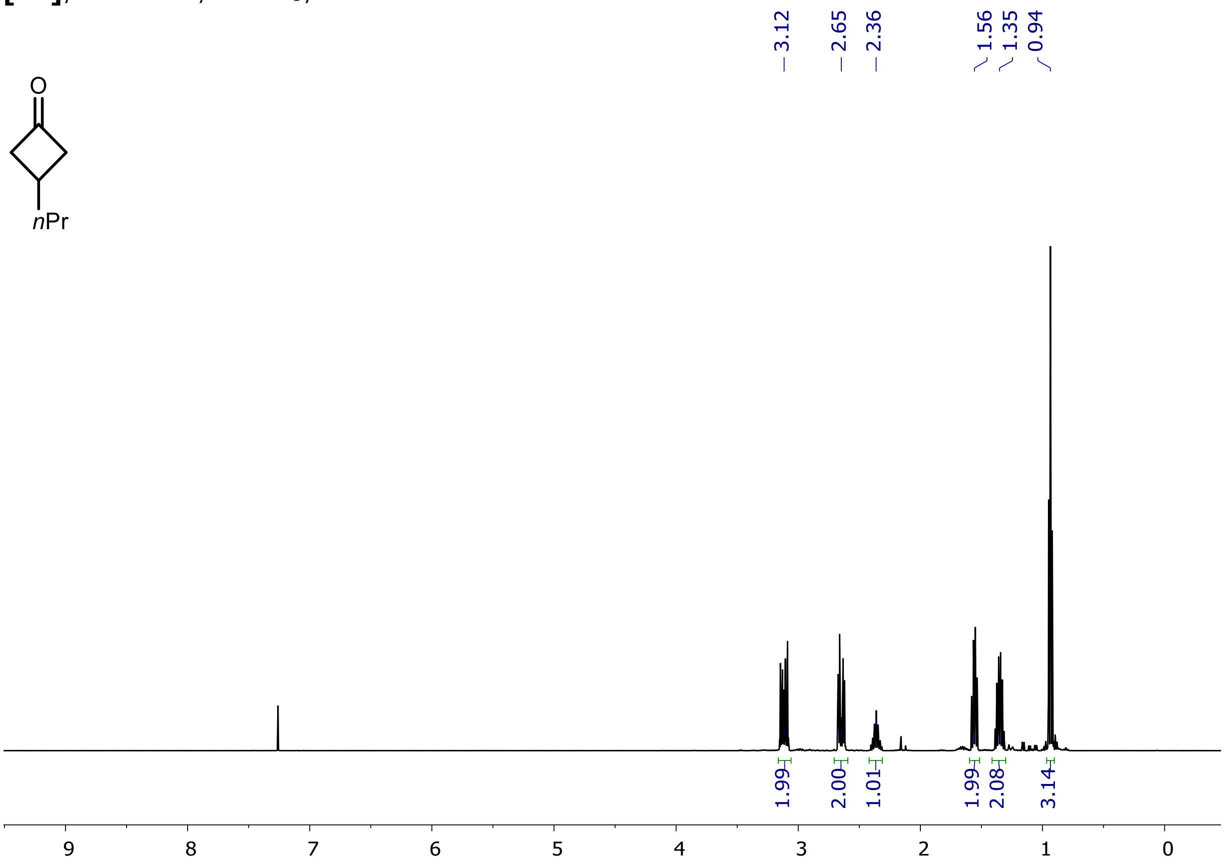


**[1h]**, $^{13}$C NMR, $CDCl_3$, 126 MHz

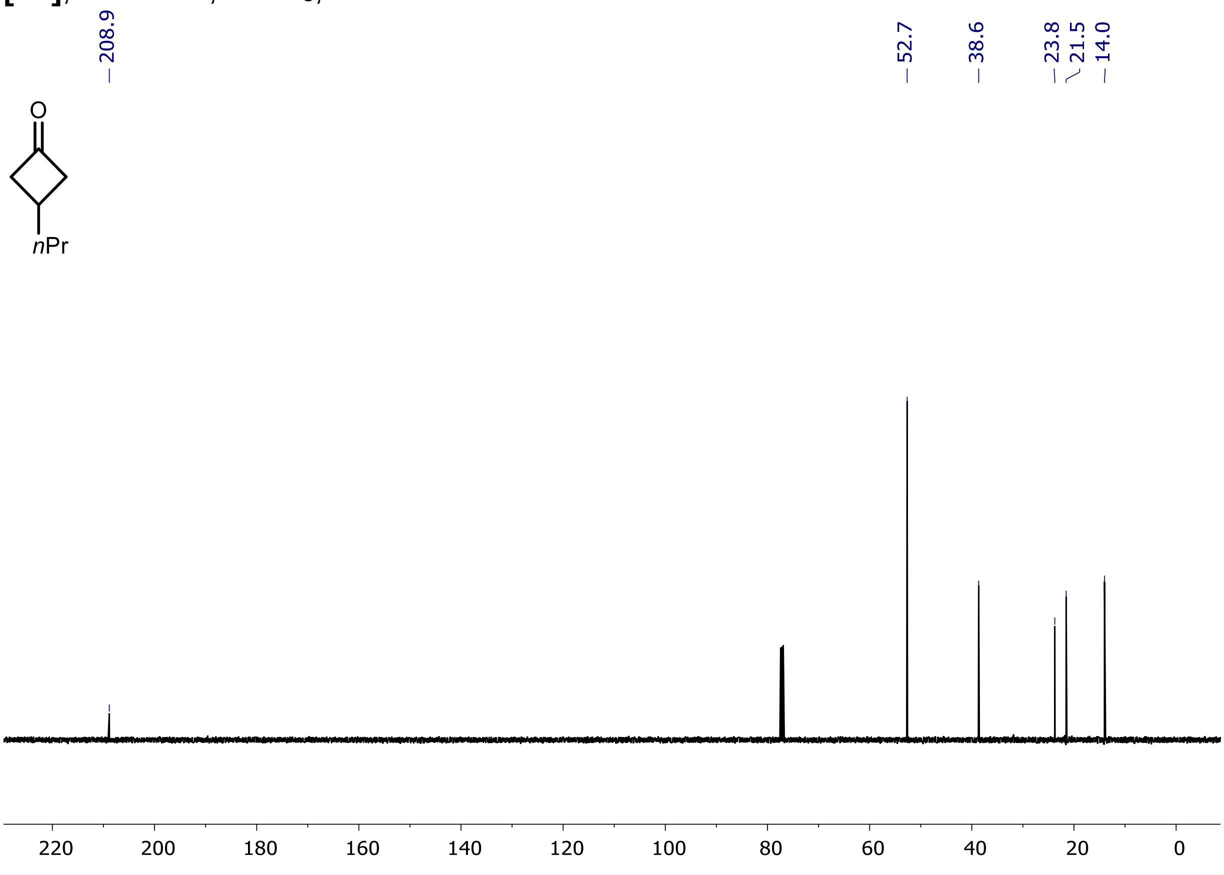

**[1i]**, $^{1}$H NMR, CDCl$_3$, 400 MHz

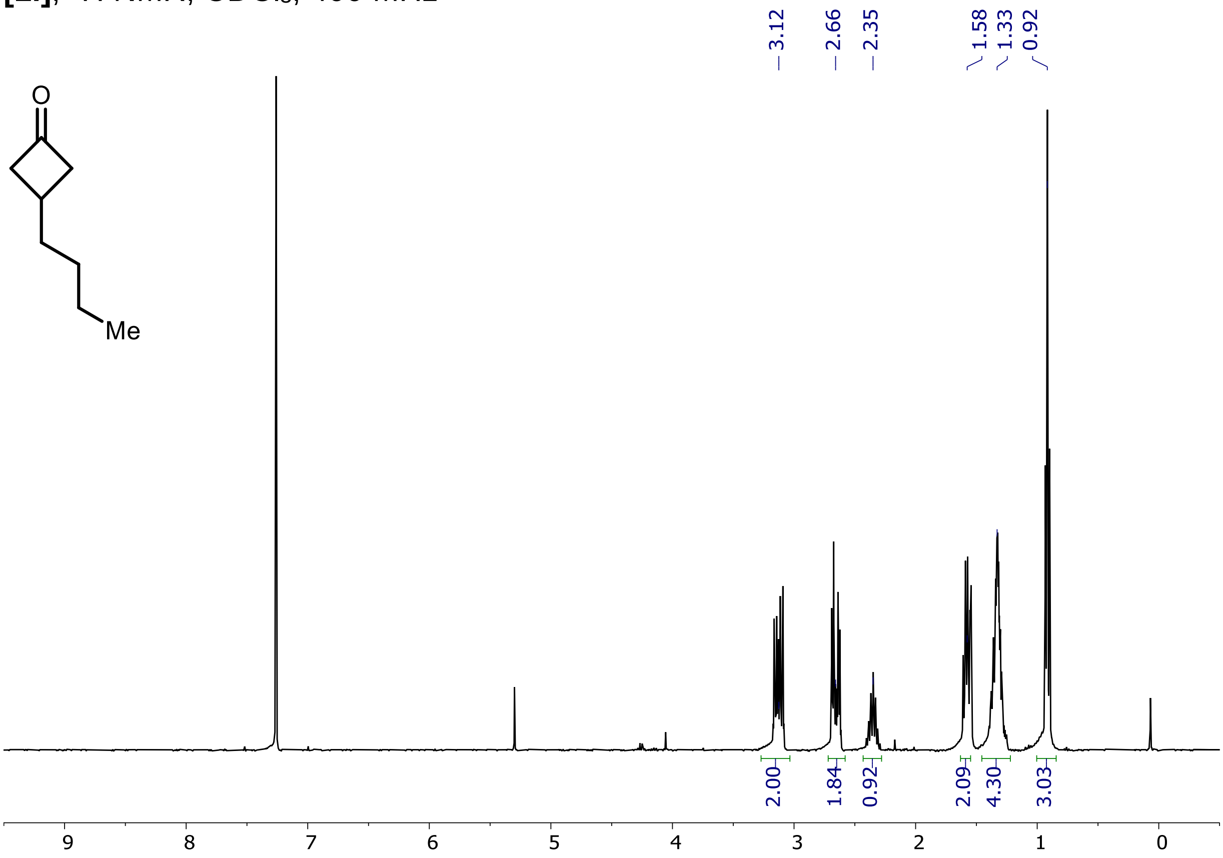


**[1i]**, $^{13}$C NMR, CDCl$_3$, 125 MHz

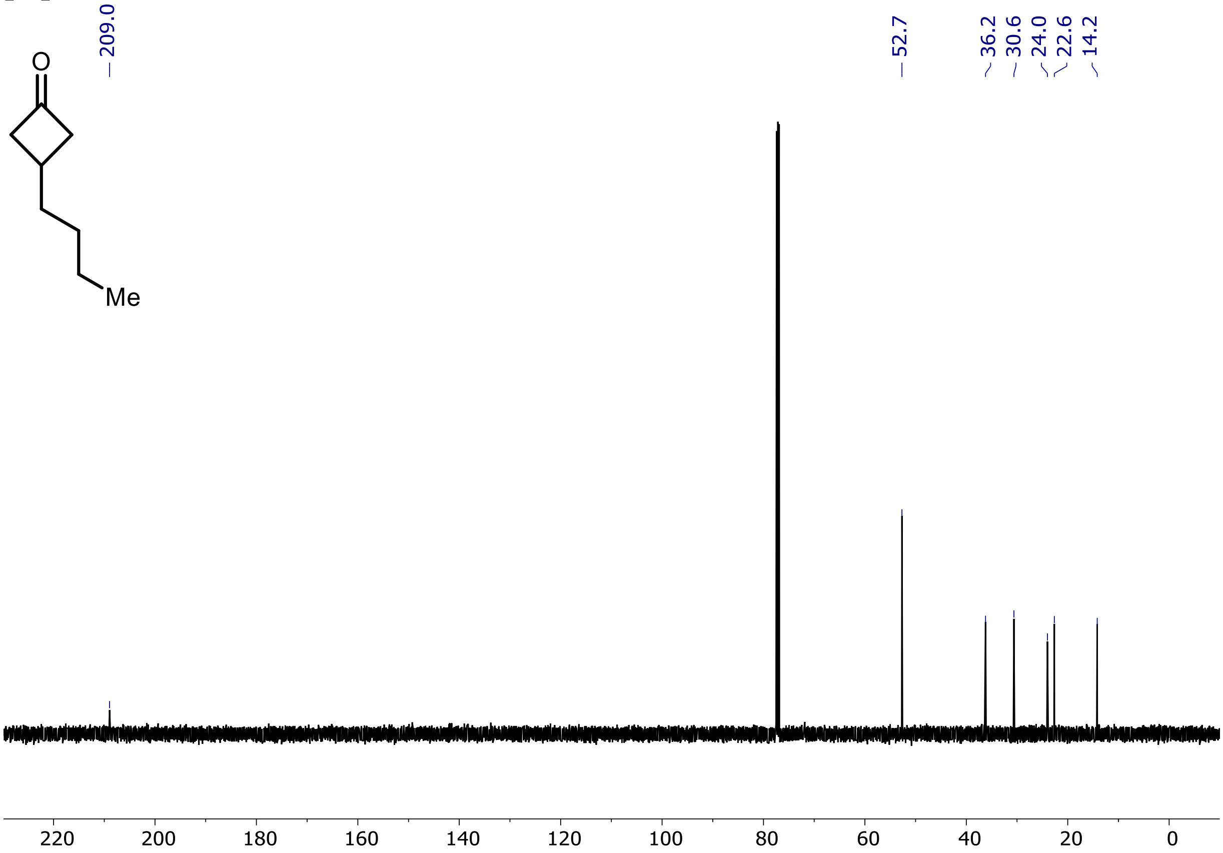

**[1j]**, $^{1}$H NMR, $CDCl_3$, 400 MHz

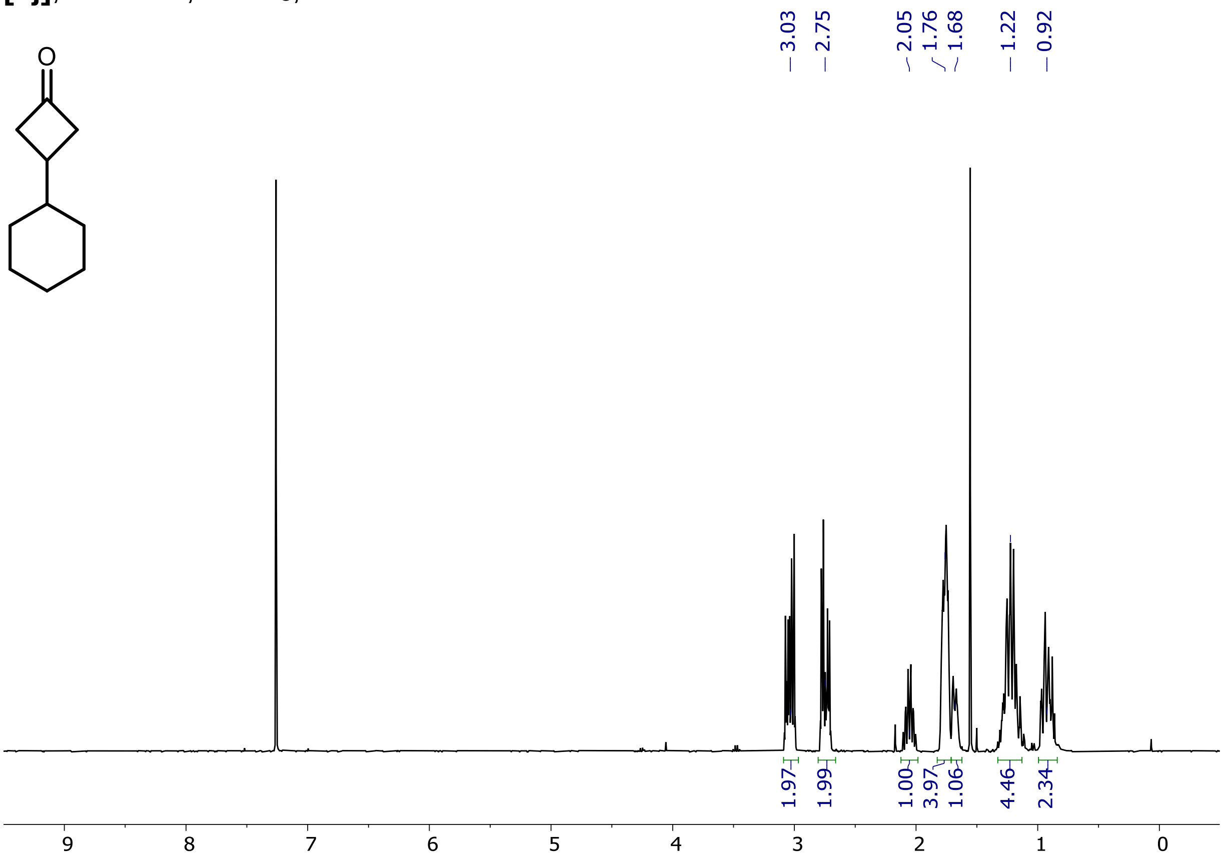


**[1j]**, $^{13}$C NMR, $CDCl_3$, 125 MHz

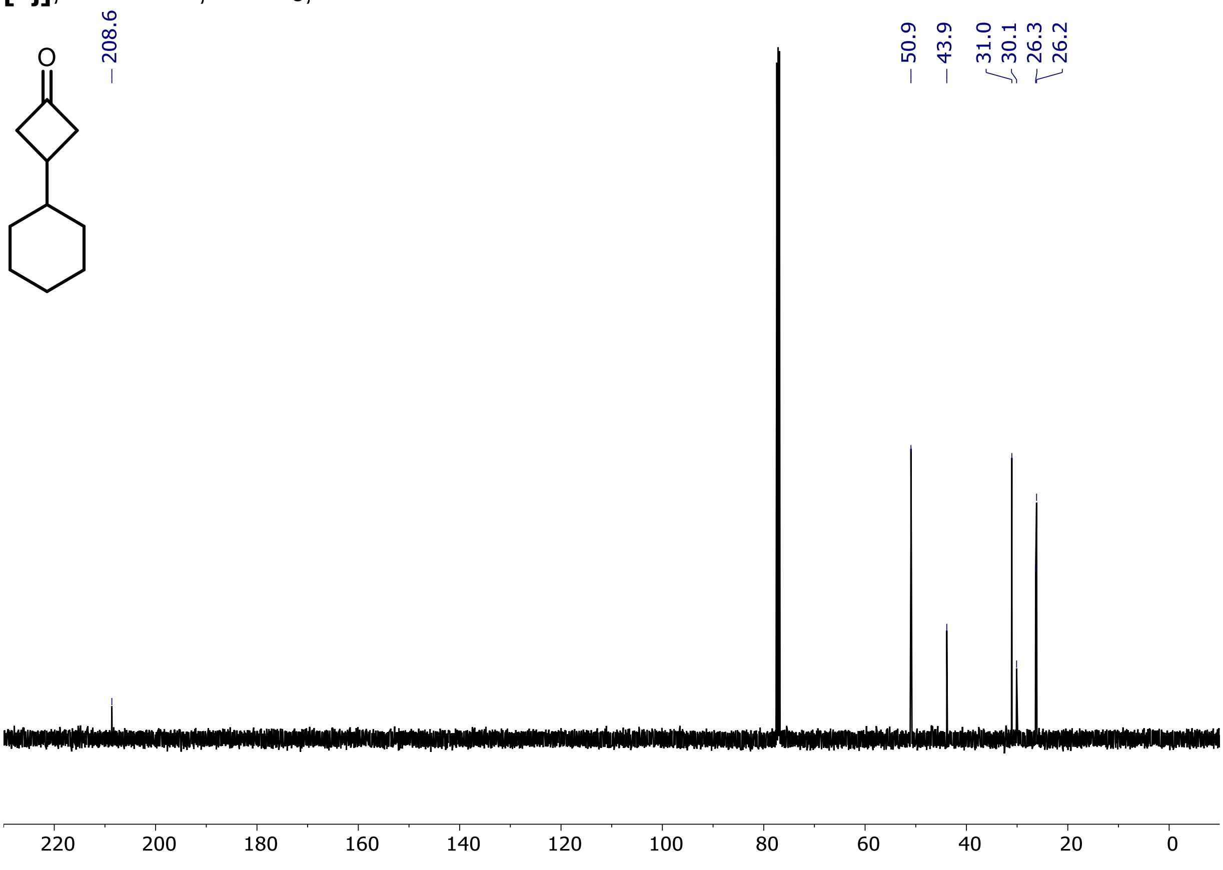

**[1k]**, $^{1}$H NMR, $CDCl_3$, 400 MHz

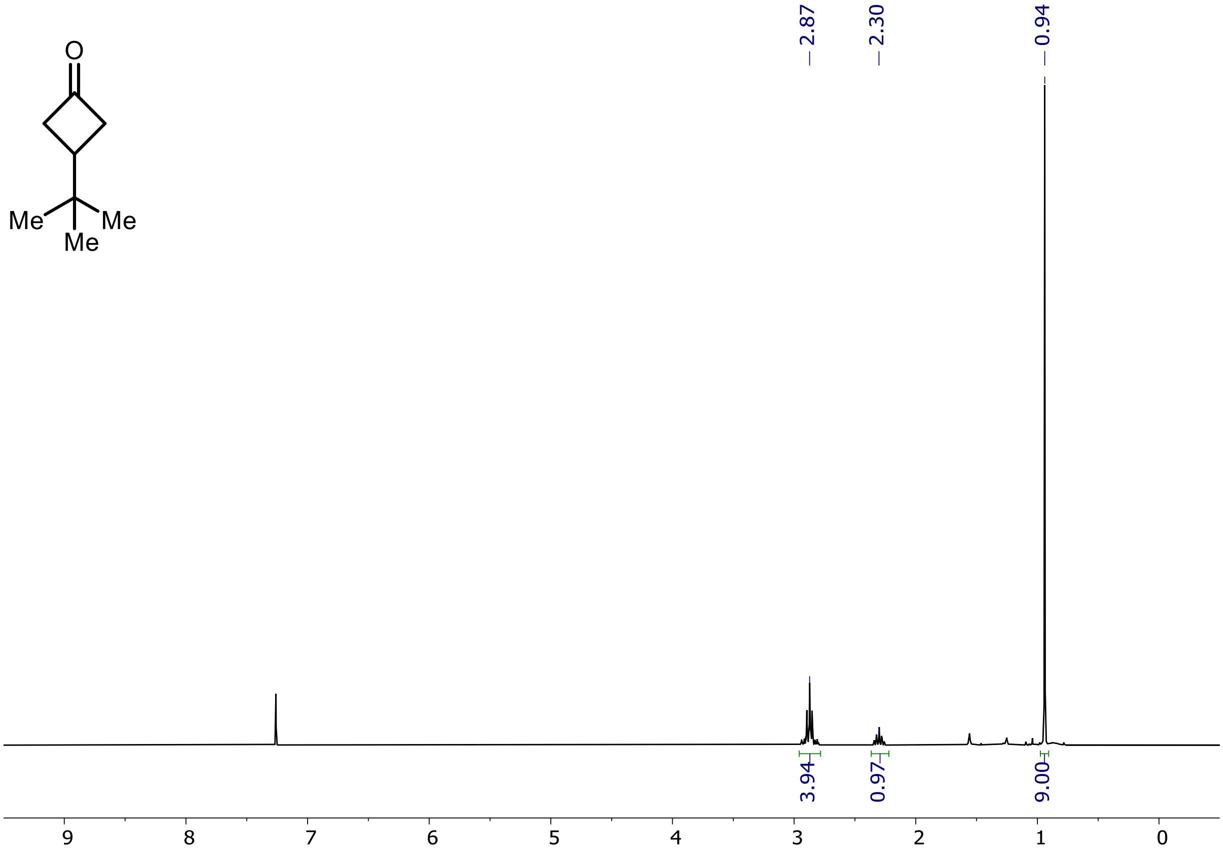


**[1k]**, $^{13}$C NMR, $CDCl_3$, 101 MHz

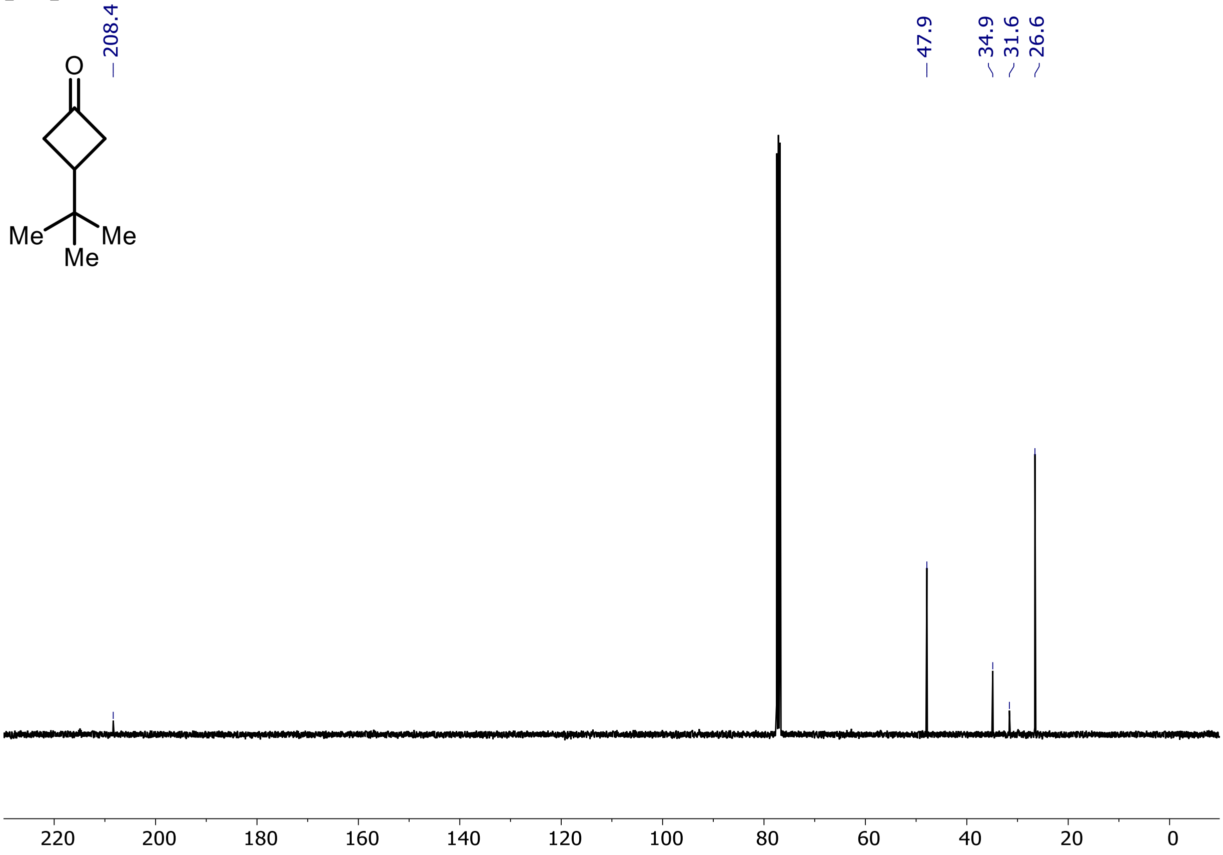

**[1l]**, $^{1}$H NMR, $CDCl_3$, 400 MHz

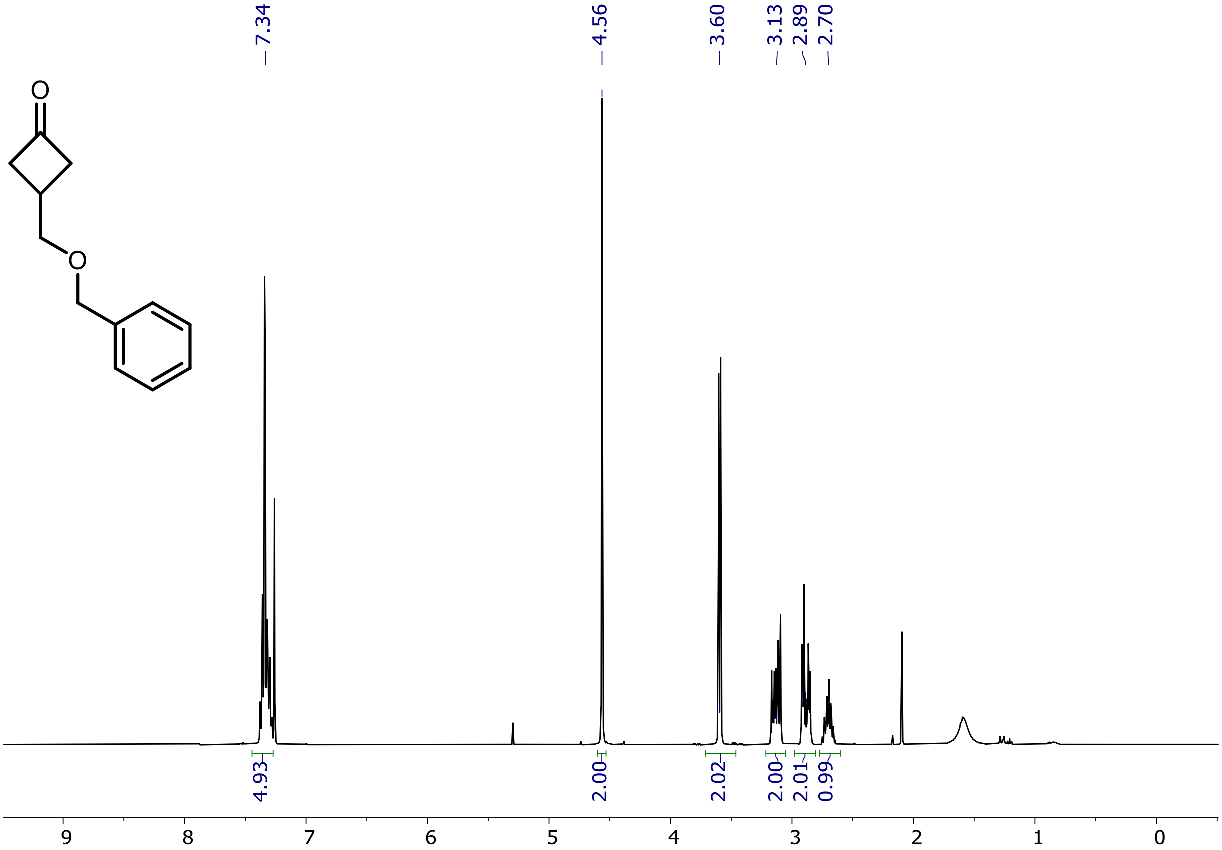


**[1l]**, $^{13}$C NMR, $CDCl_3$, 101 MHz

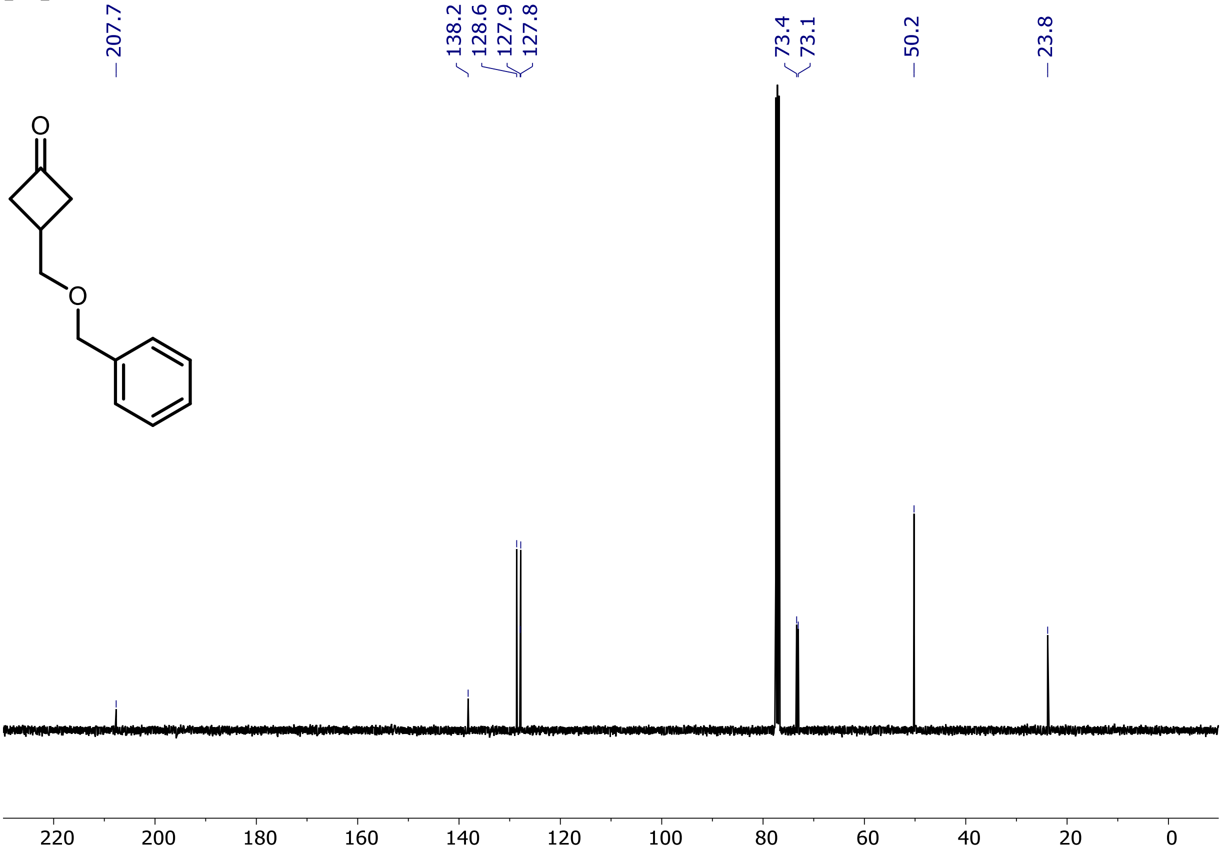

[1n], $^{1}$H NMR, $CDCl_3$, 400 MHz

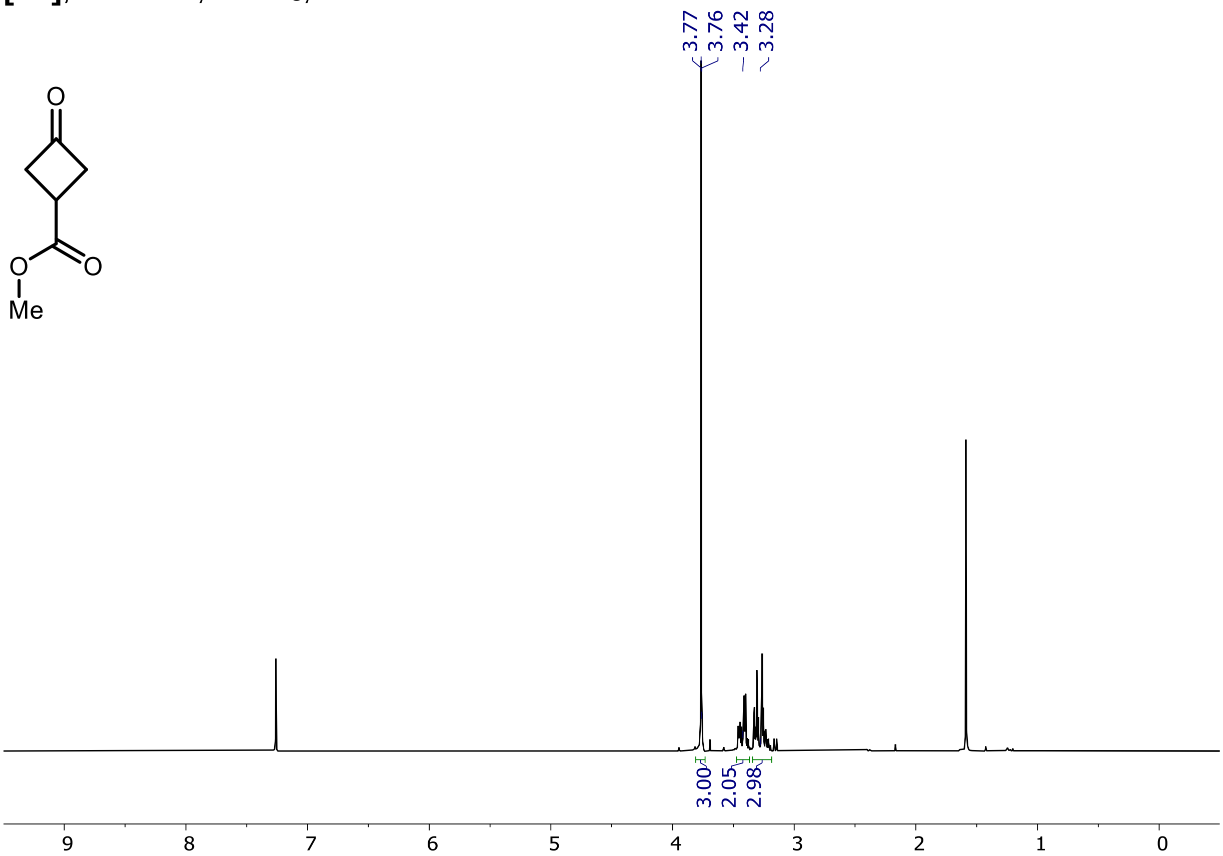


[1n], $^{13}$C NMR, $CDCl_3$, 101 MHz

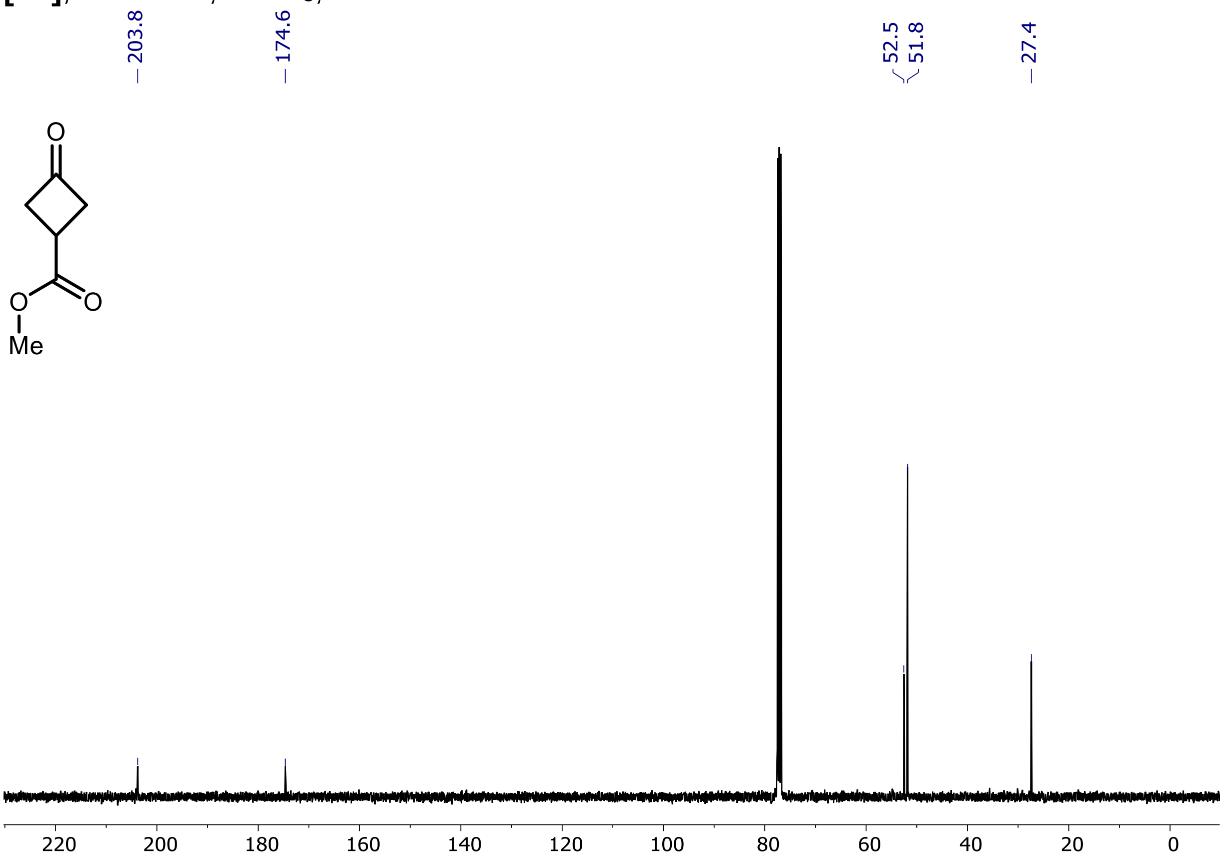

**[1o]**, $^{1}$H NMR, $CDCl_3$, 400 MHz

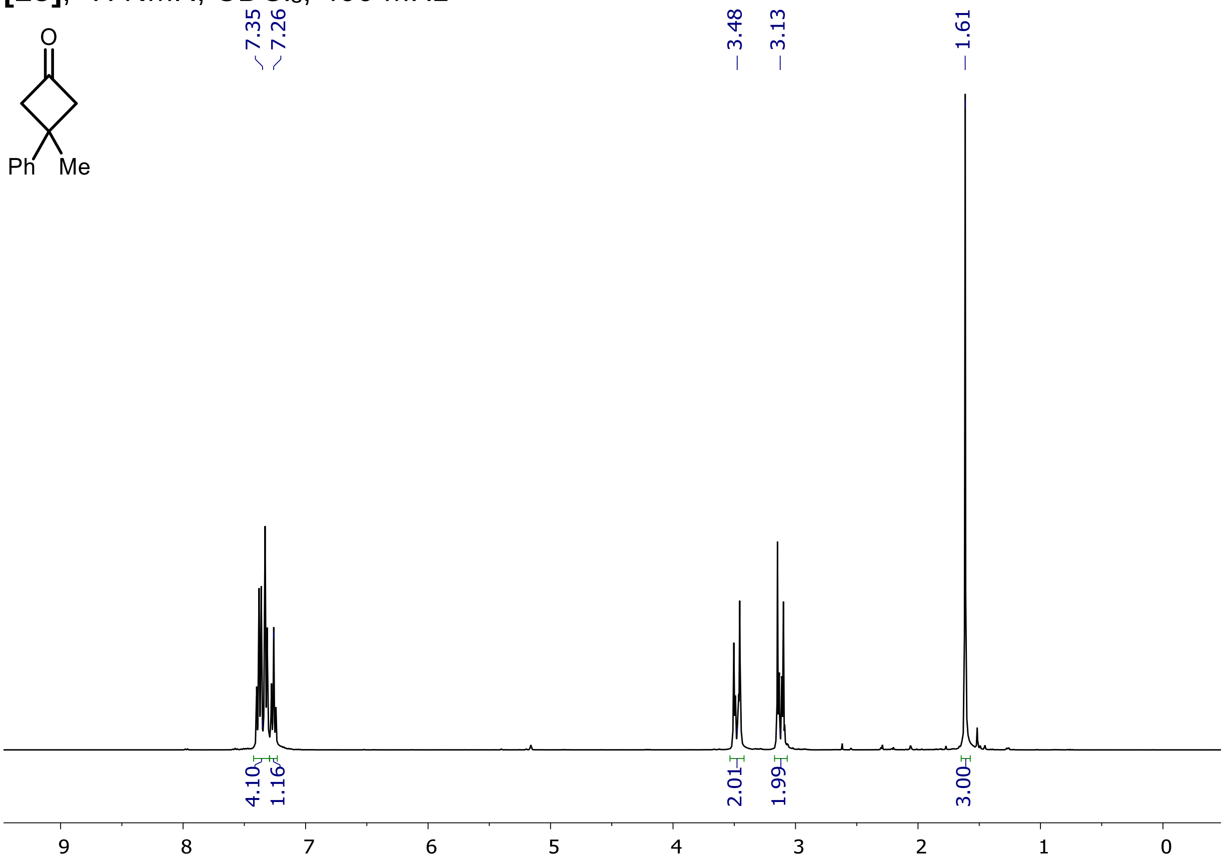


**[1o]**, $^{13}$C NMR, $CDCl_3$, 101 MHz

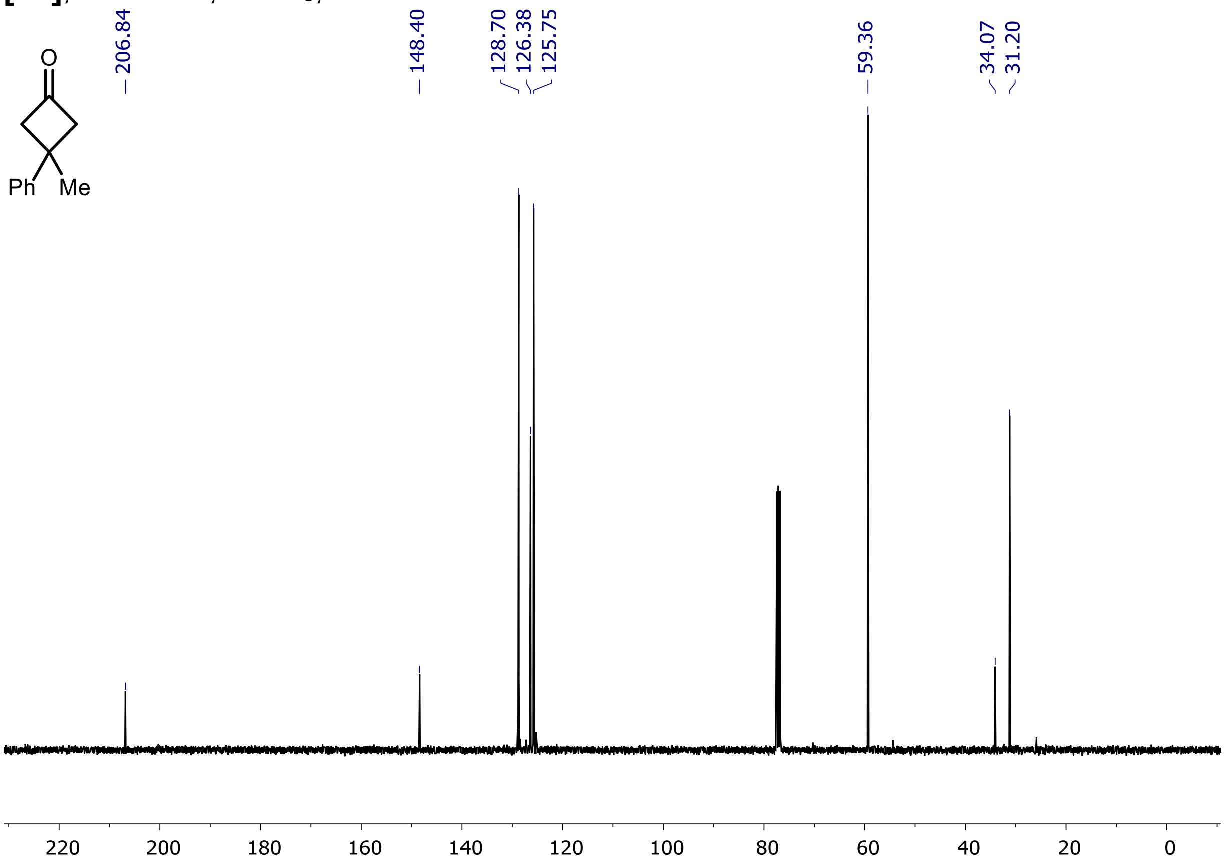

**[1p]**, $^{1}H$, $CDCl_3$, 400 MHz

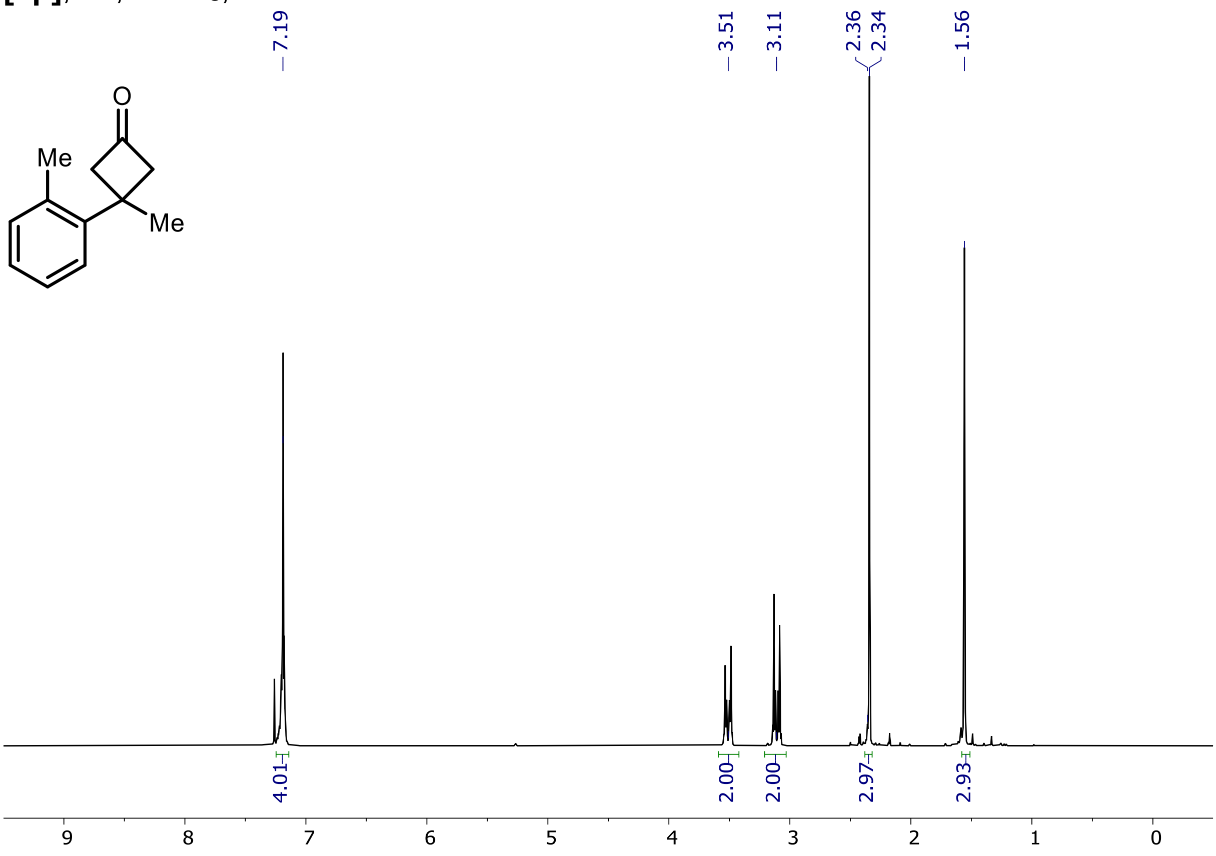


**[1p]**, $^{13}C$, $CDCl_3$, 101 MHz

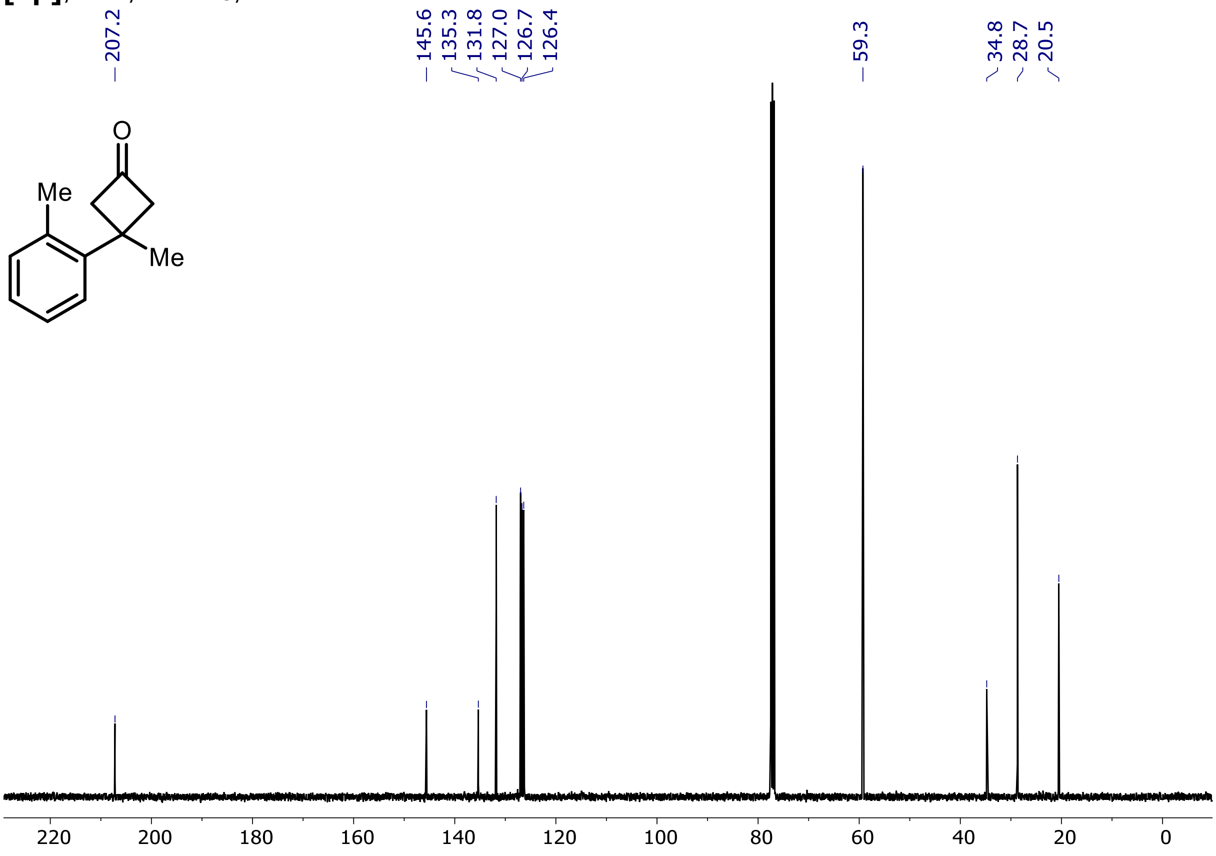

**[1q]**, $^{1}$H NMR, $CDCl_3$, 500 MHz

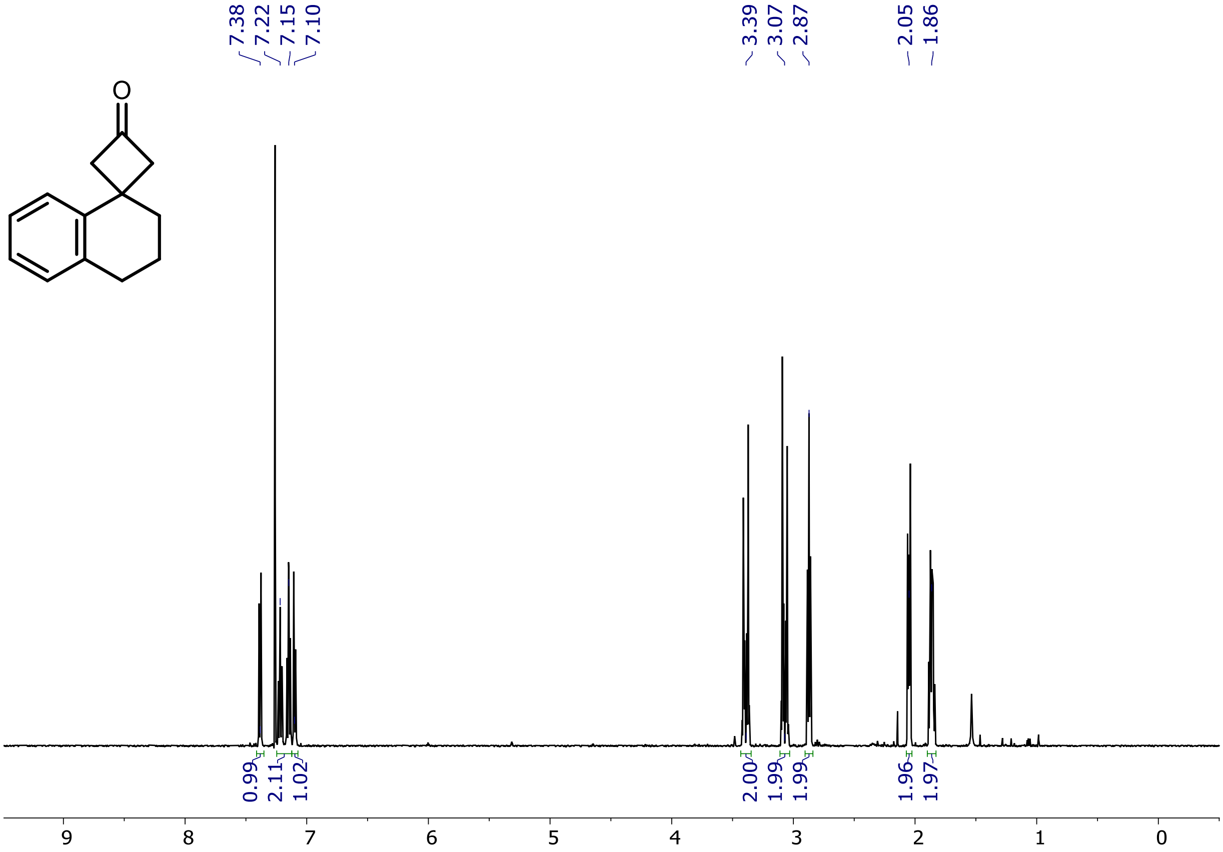


**[1q]**, $^{13}$C NMR, $CDCl_3$, 126 MHz

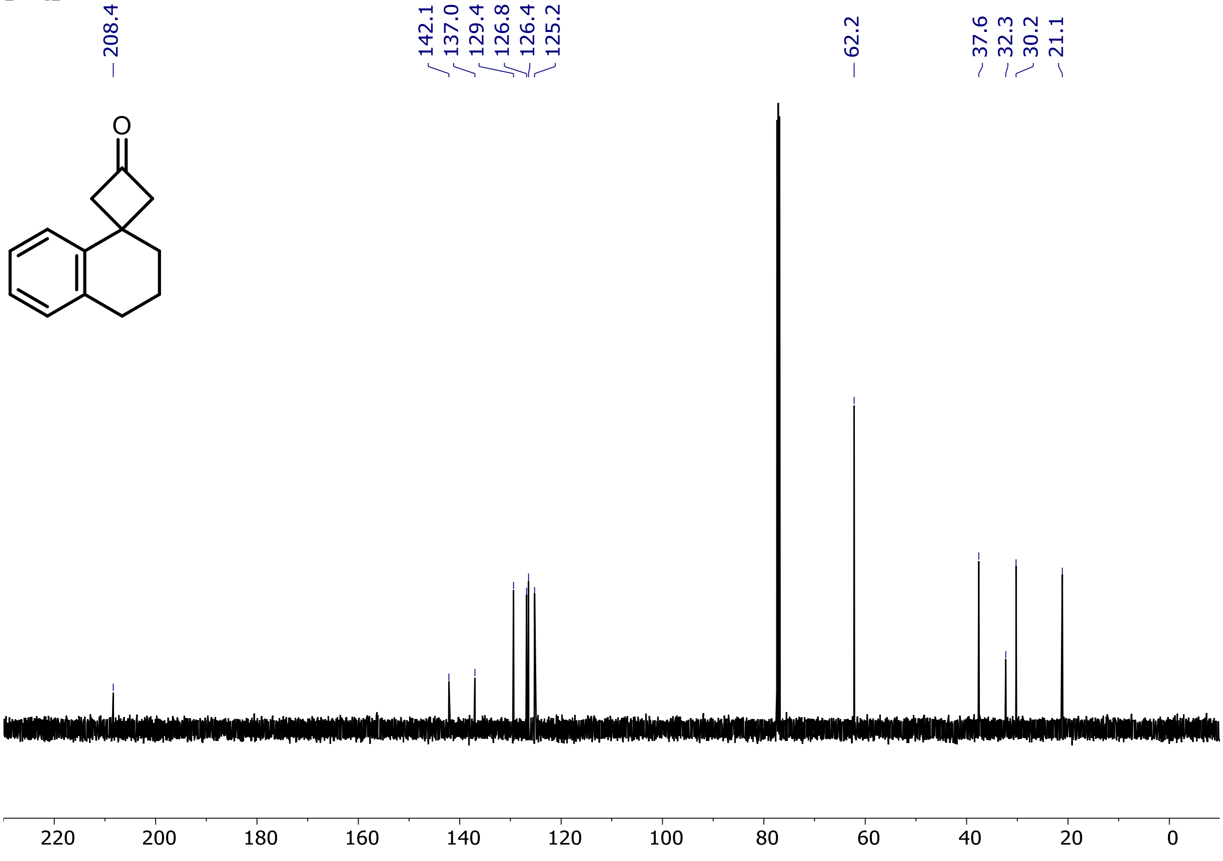

[1r], $^{1}H$ NMR, $CDCl_3$, 400 MHz

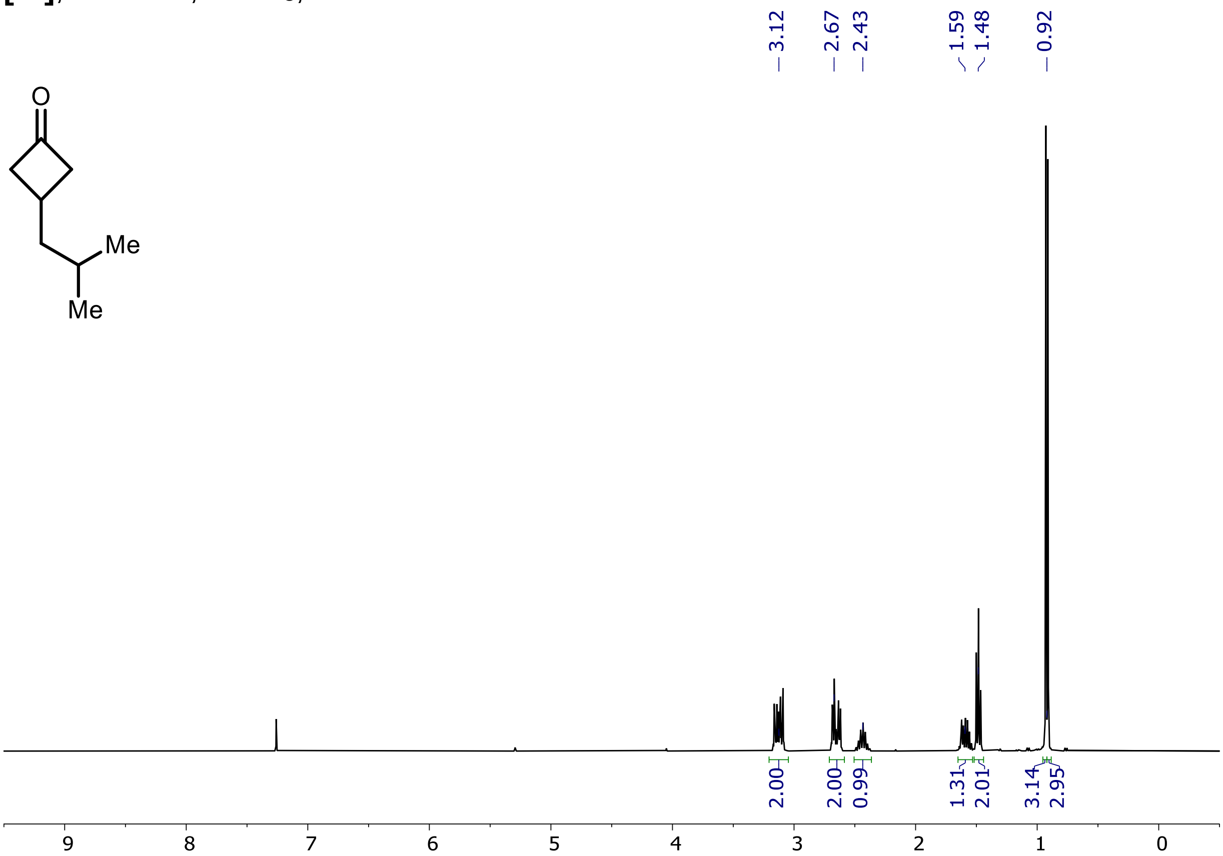


[1r], $^{13}C$ NMR, $CDCl_3$, 101 MHz

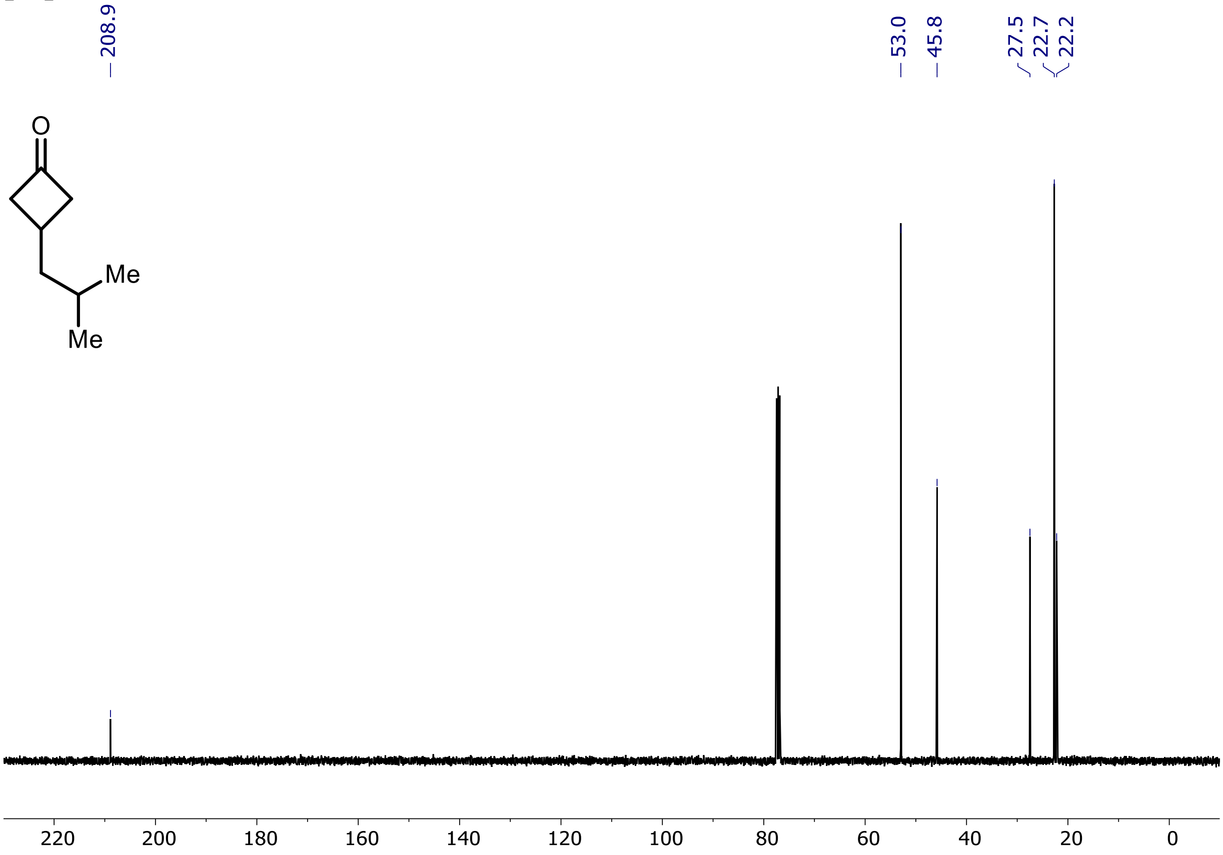

**[4a, *epi*-4a]**, $^{1}$H NMR, DMSO-d6, 600 MHz

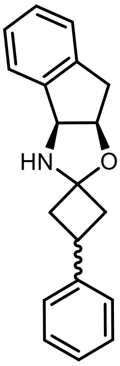


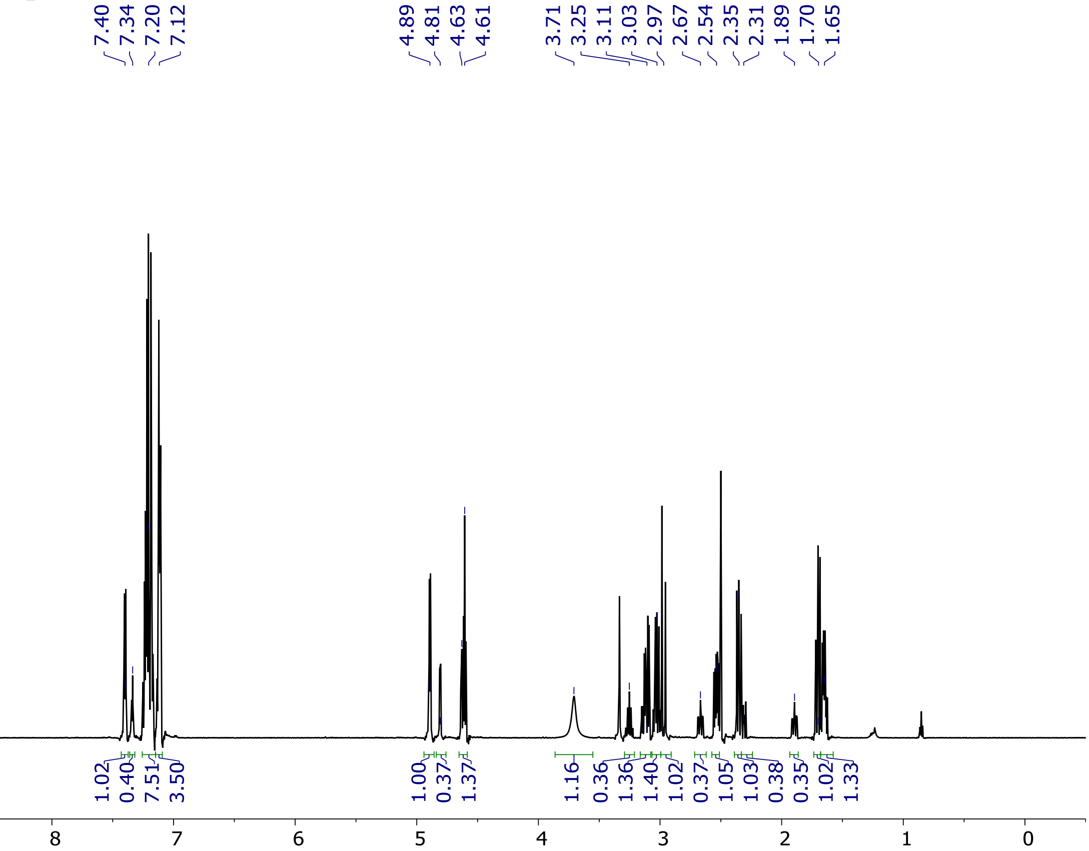


**[4a, *epi*-4a]**, $^{13}$C NMR, DMSO-d6, 151 MHz

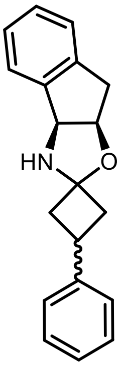


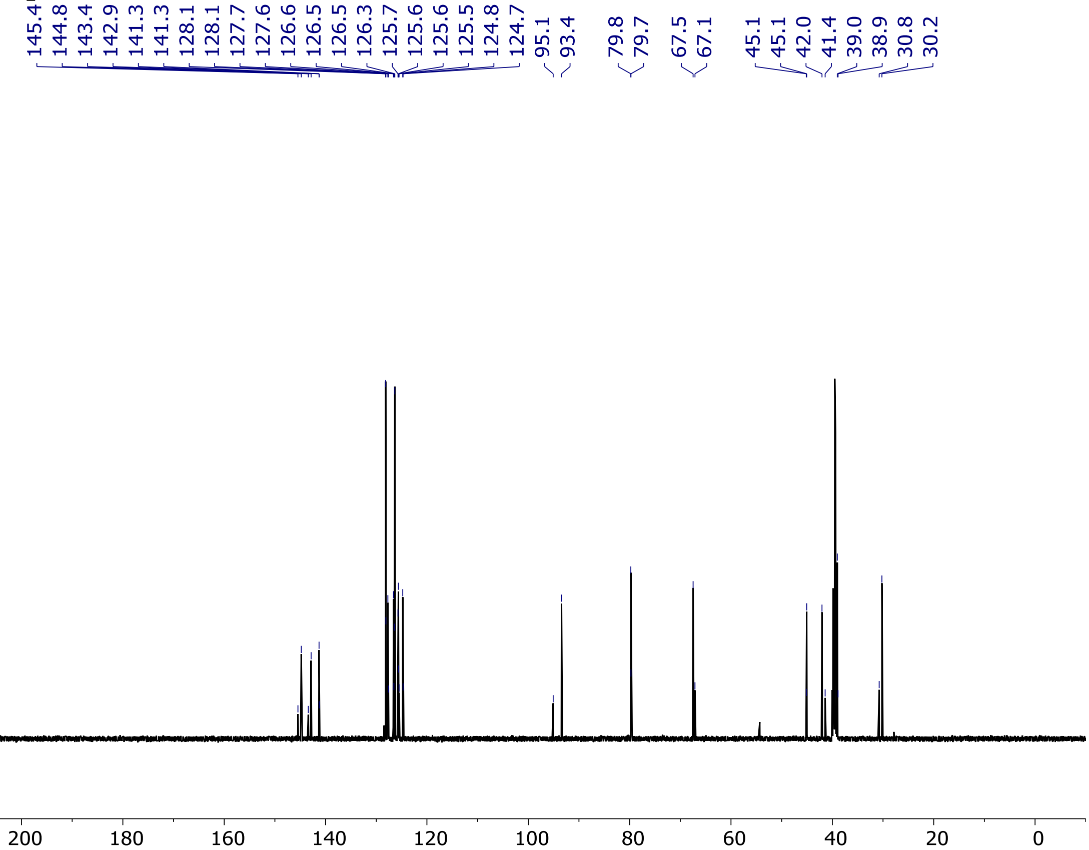

**[4a]** and **[*epi*-4a]**, $^{1}$H NMR, DMSO-d6, 600 MHz

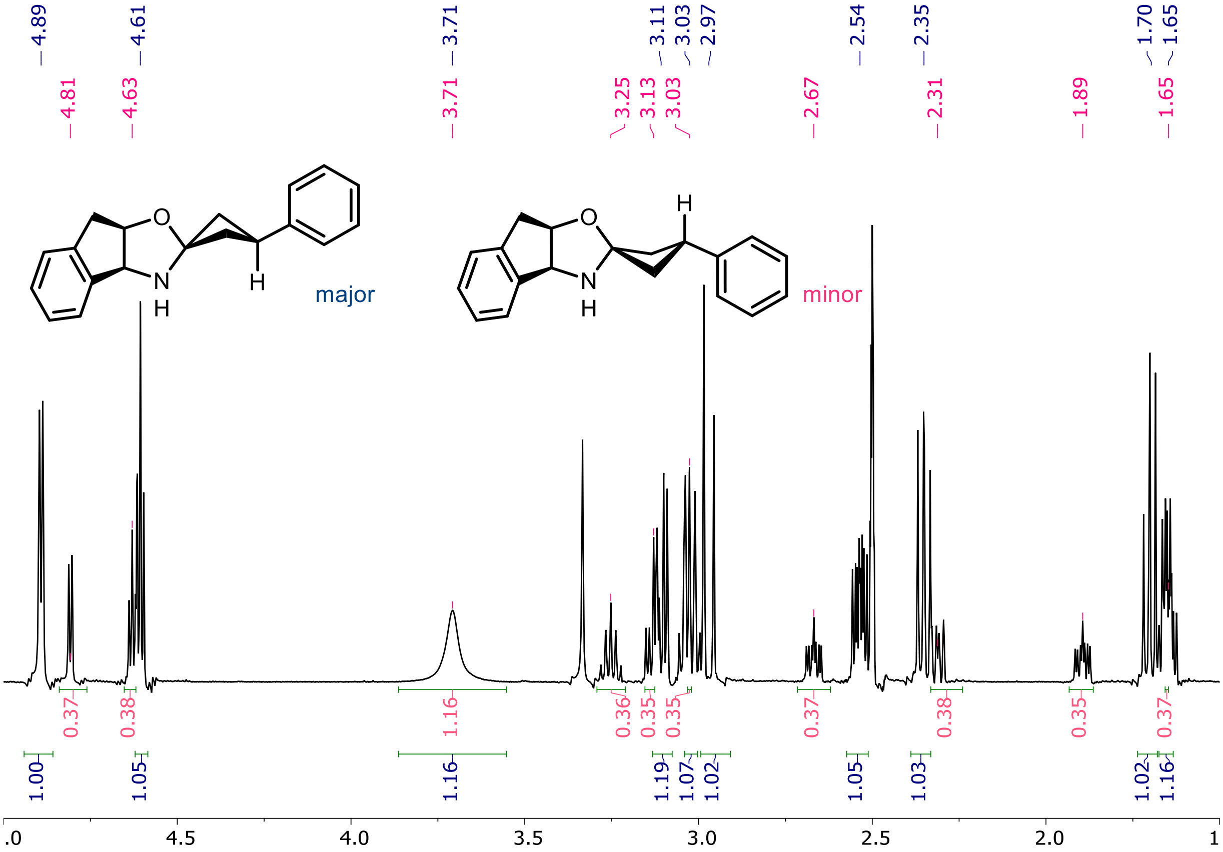


**[4a]** and **[*epi*-4a]**, $^{13}$C NMR, DMSO-d6, 150 MHz

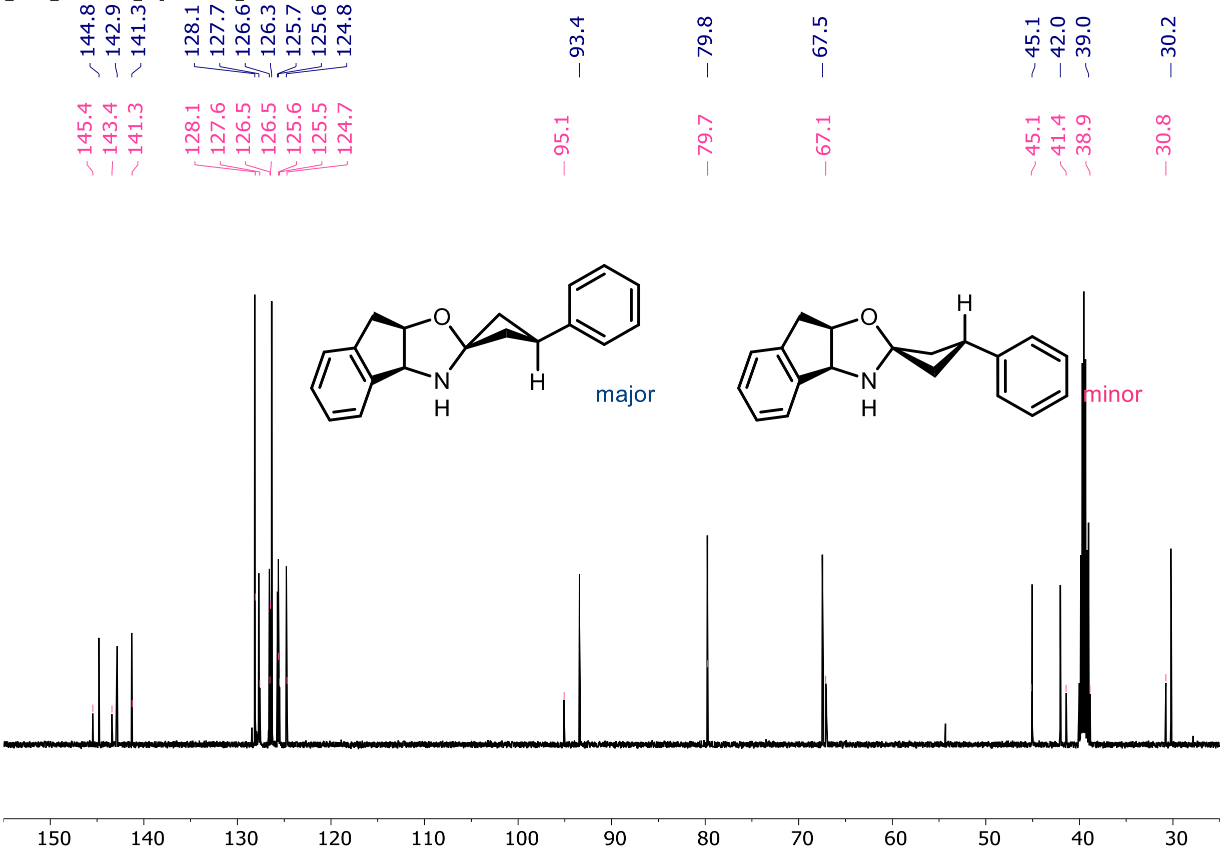

**[4a, *epi*-4a]**, COSY, DMSO-d6

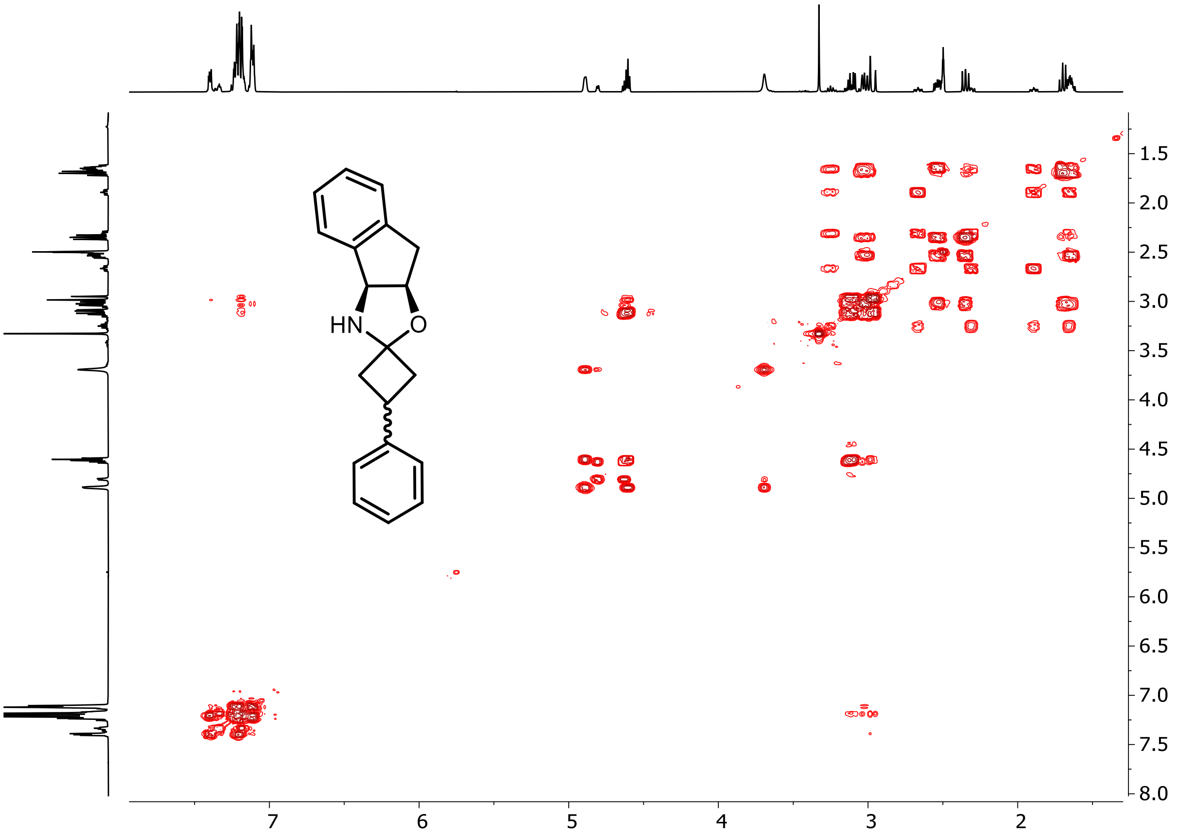


**[4a, *epi*-4a]**, HSQC, DMSO-d6

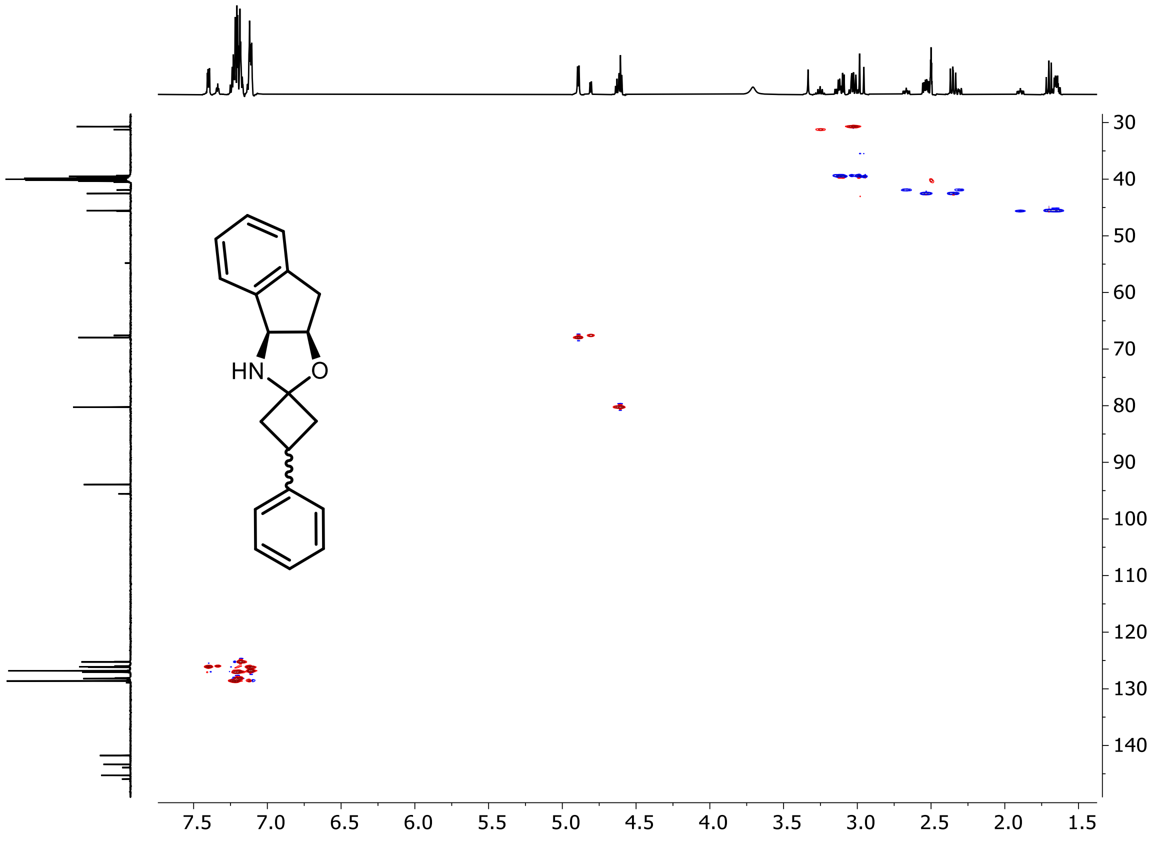

**[4a, *epi*-4a]**, HMBC, DMSO-d6

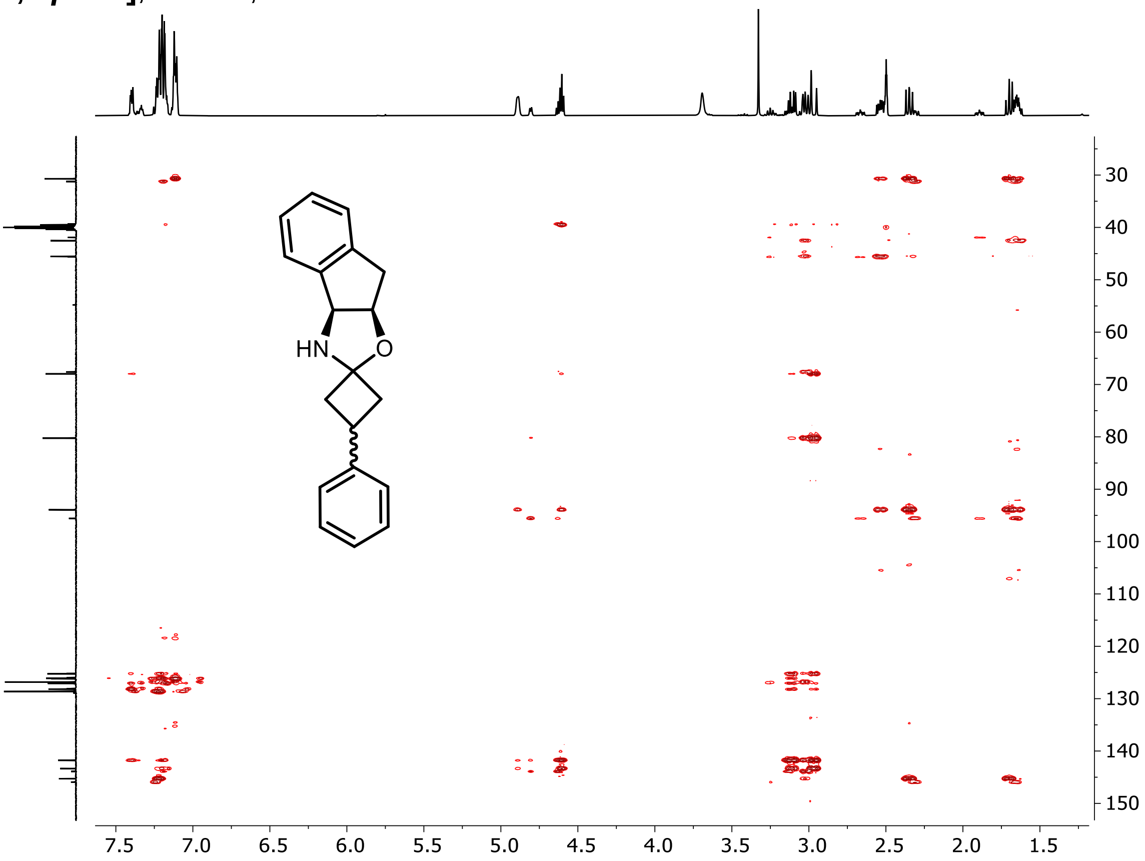


**[4a]**, 1D-NOESY, DMSO-d6

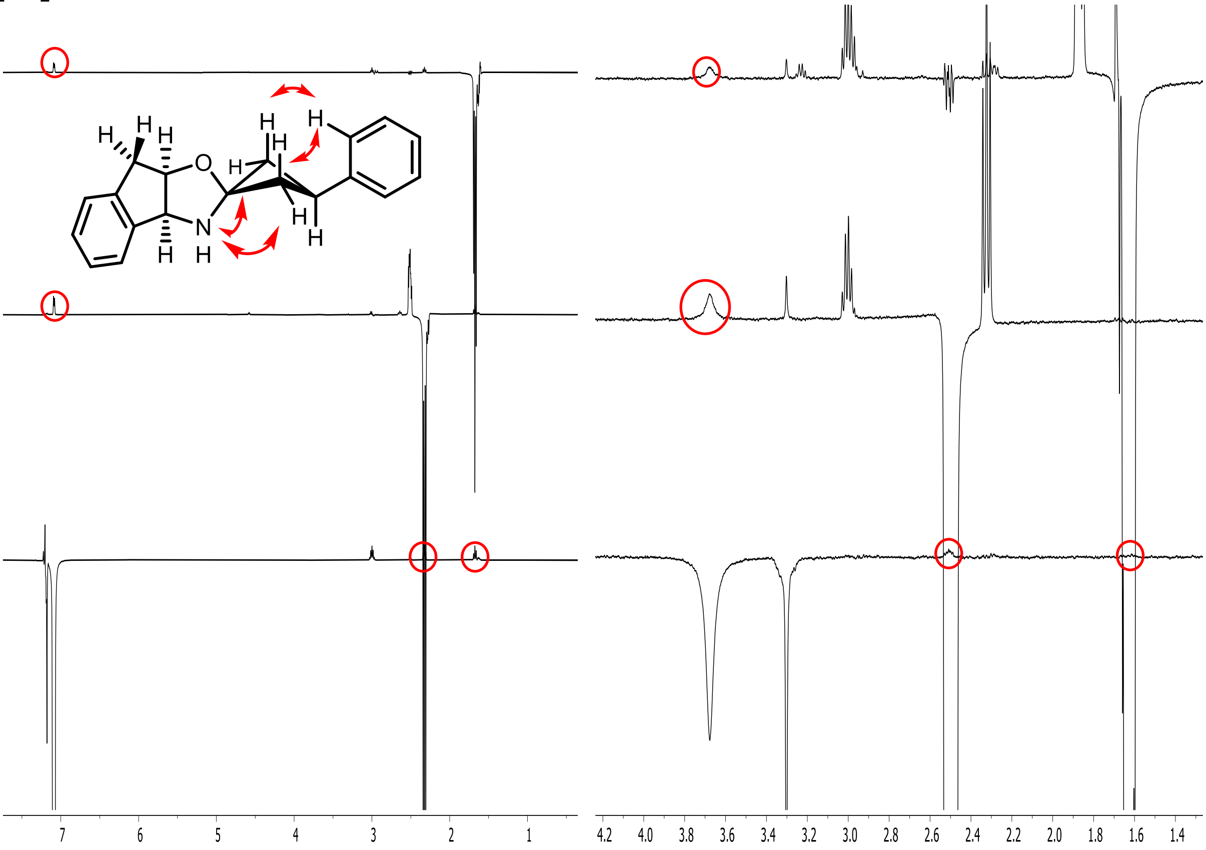

**[5a, *epi*-5a]**, $^{1}$H NMR, $C_6D_6$, 600 MHz

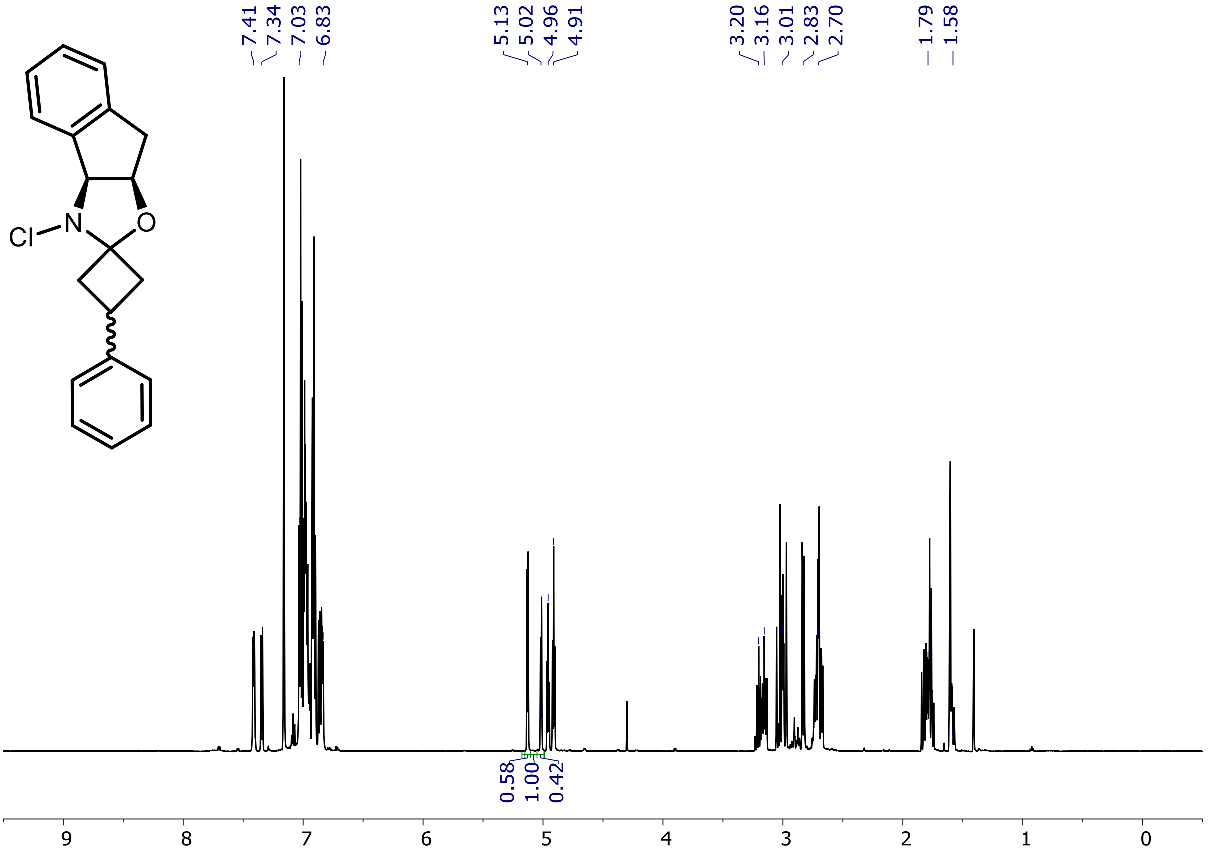


**[5a, *epi*-5a]**, $^{13}$C NMR, $C_6D_6$, 150 MHz

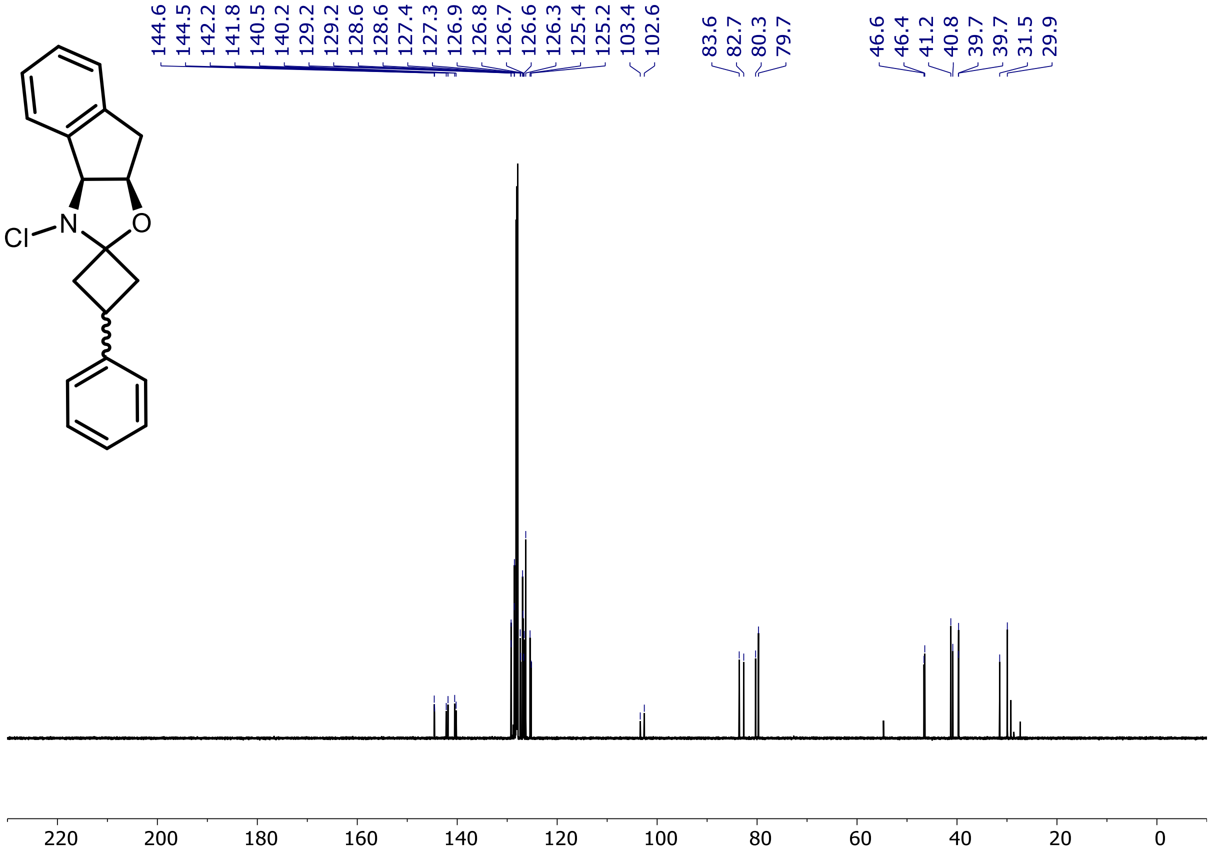

**[5a, *epi*-5a]**, COSY, $C_6D_6$

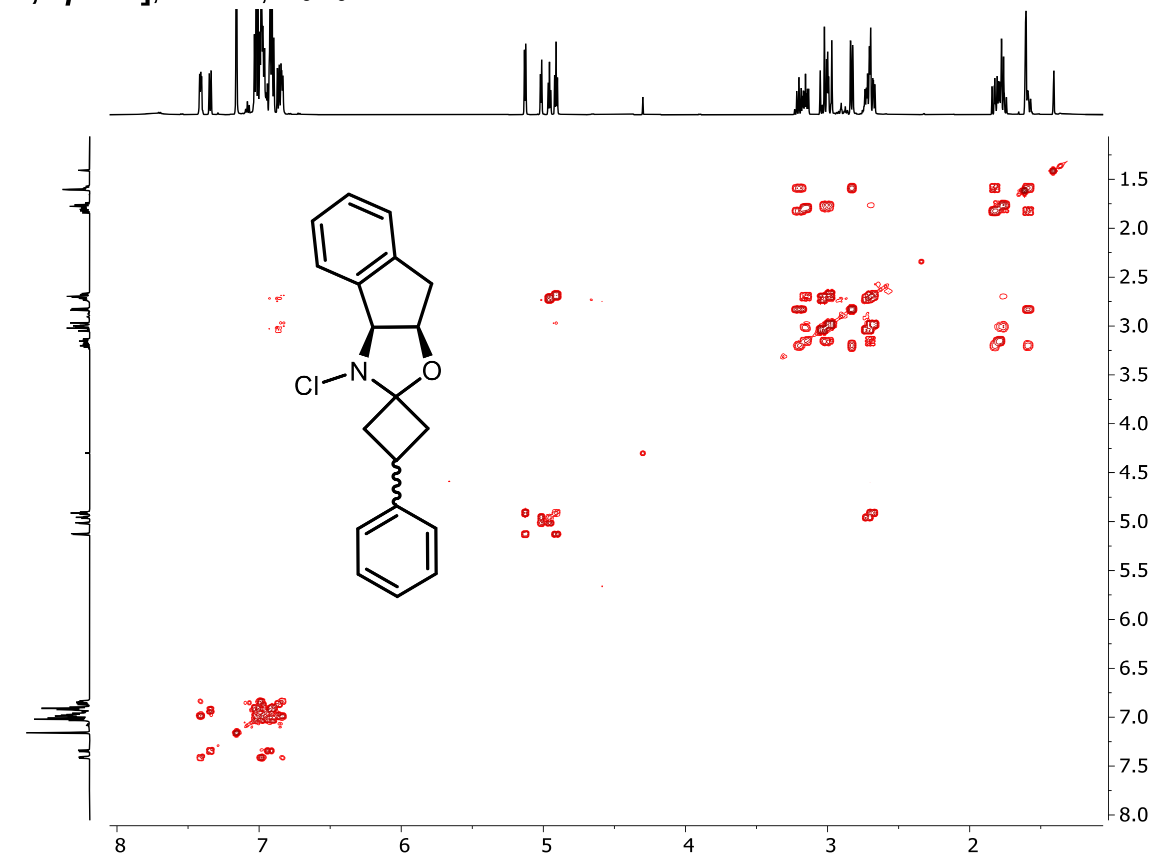


**[5a, *epi*-5a]**, HSQC, $C_6D_6$

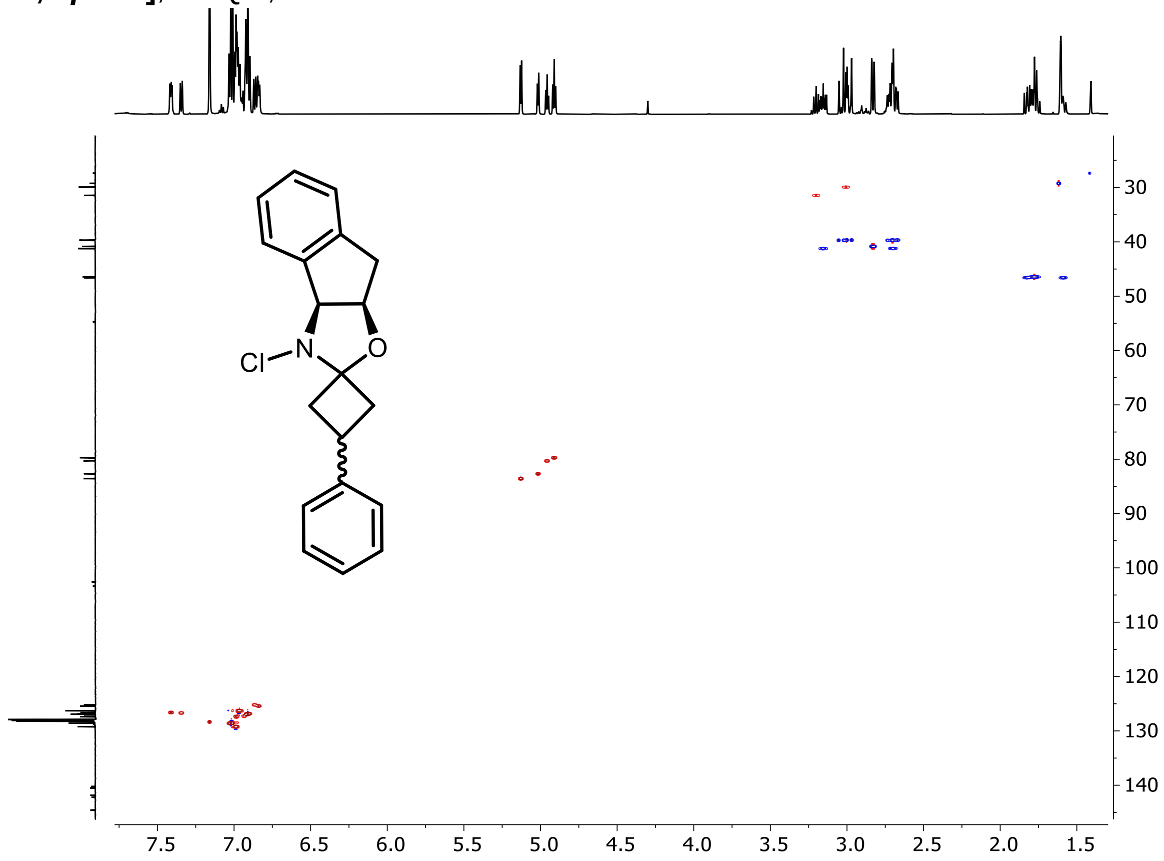

**[5a, *epi*-5a]**, HMBC, $C_6D_6$

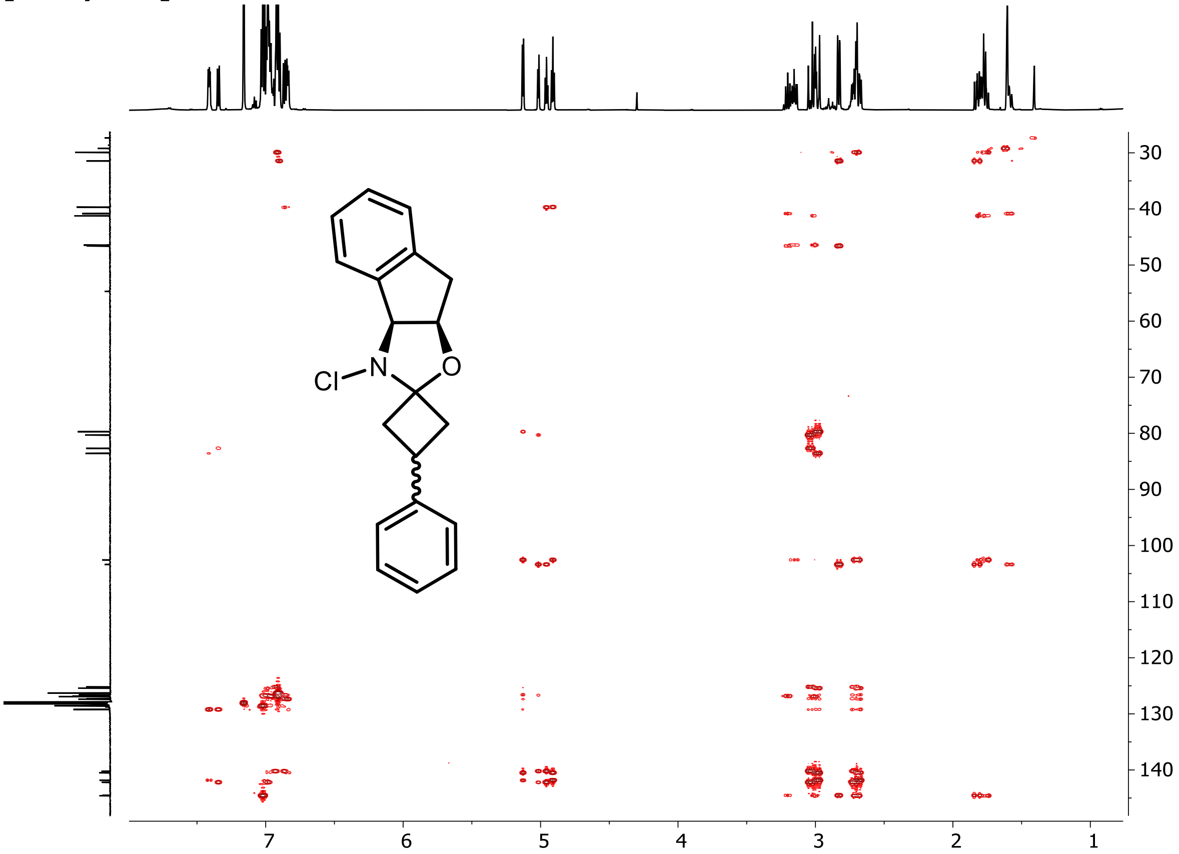


**[5a, *epi*-5a]**, NOESY, $C_6D_6$

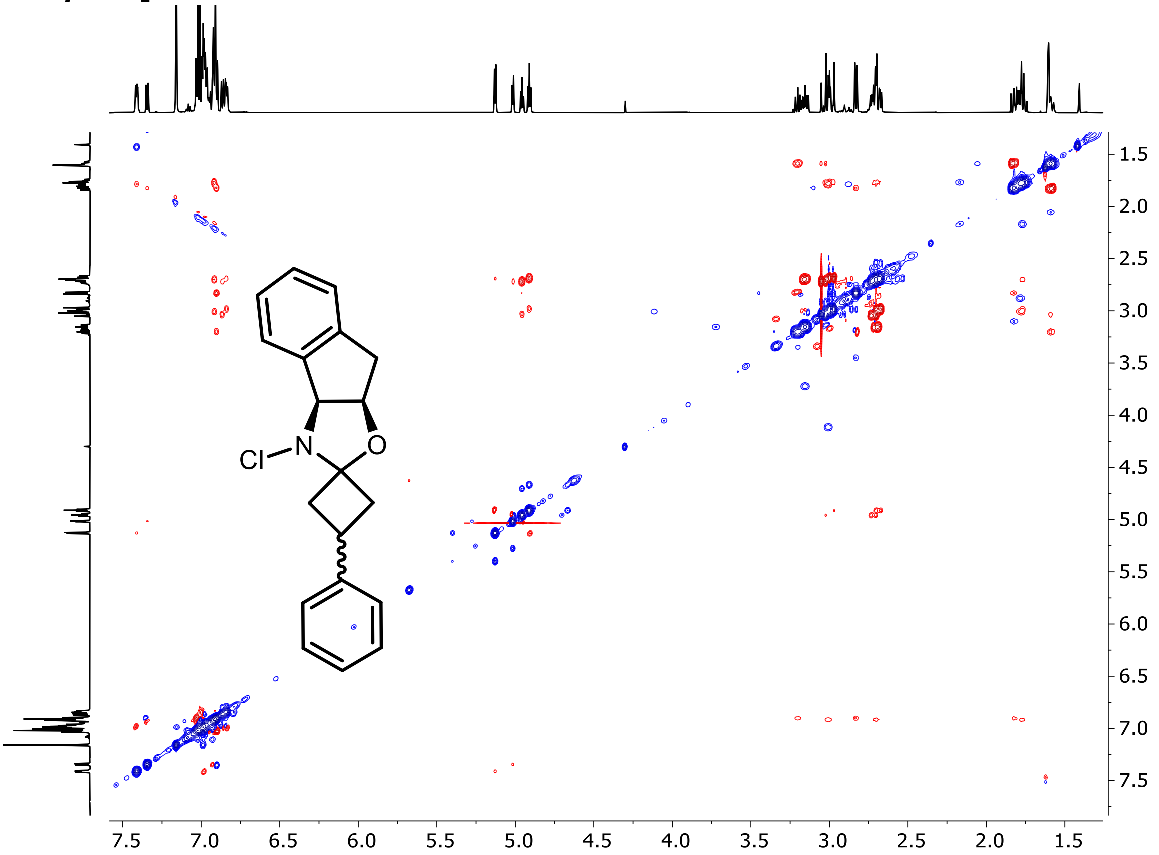

**[4a, *epi*-4a]**, $^{1}$H NMR, $CD_3OD$, 600 MHz

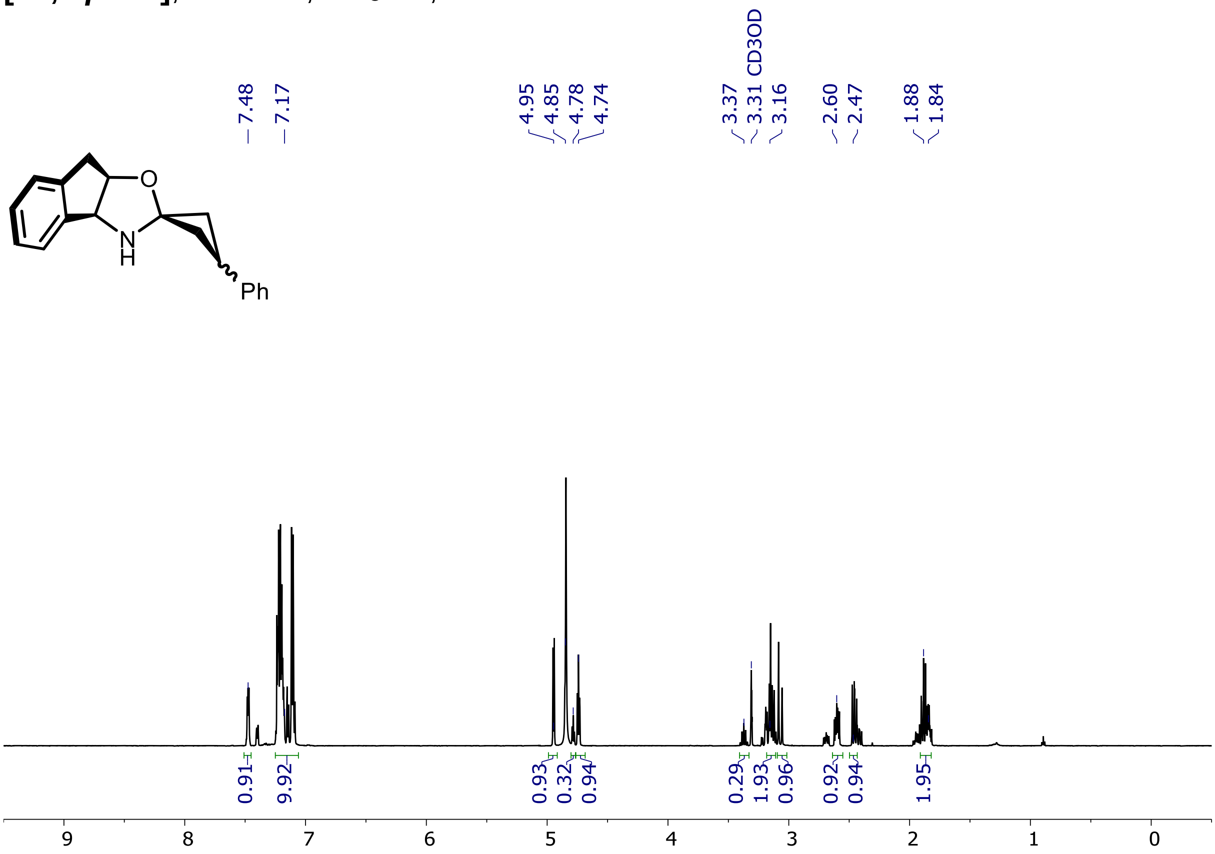


**[4a, *epi*-4a]**, $^{13}$C NMR, $CD_3OD$, 150 MHz

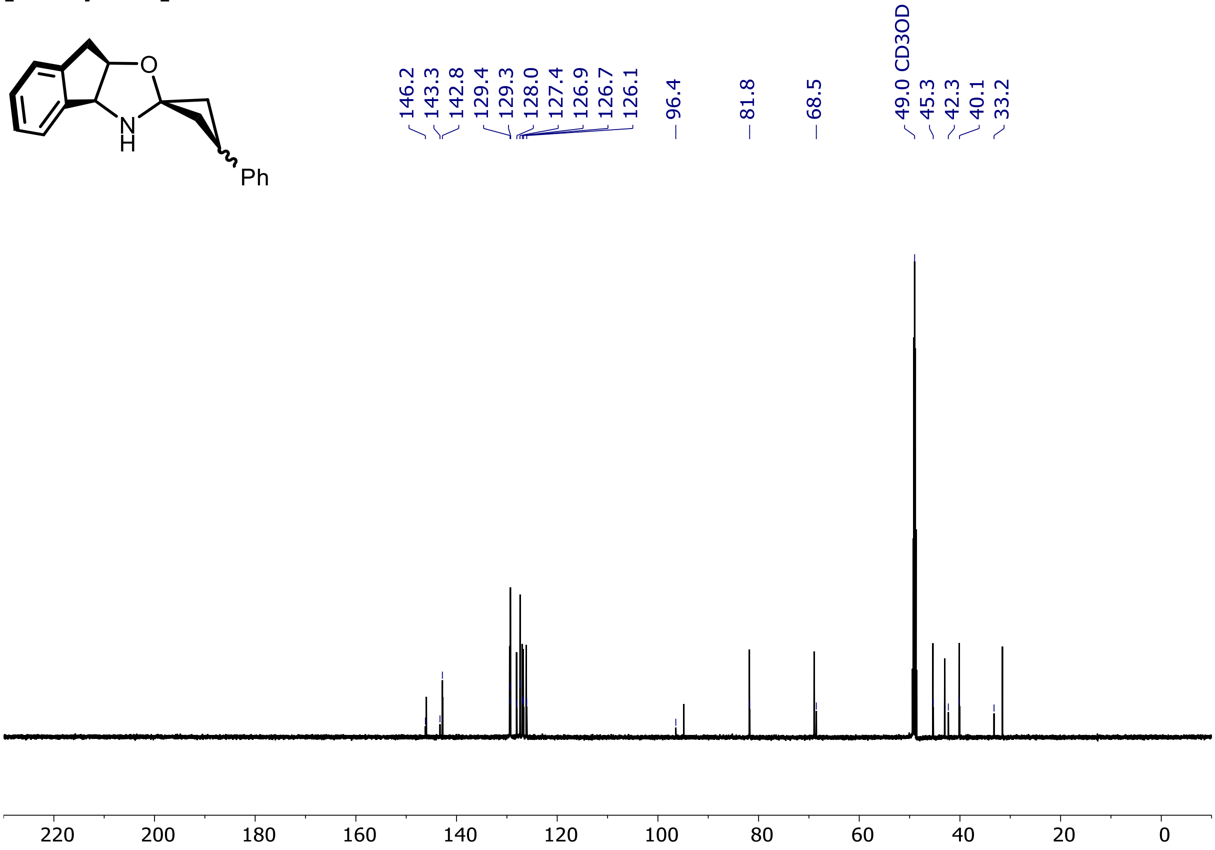

NMRs ($CD_3OD$) of reaction mixture containing carbenium **[6a, *epi*-6a]**

$^1H$ NMR, $CD_3OD$, 600 MHz

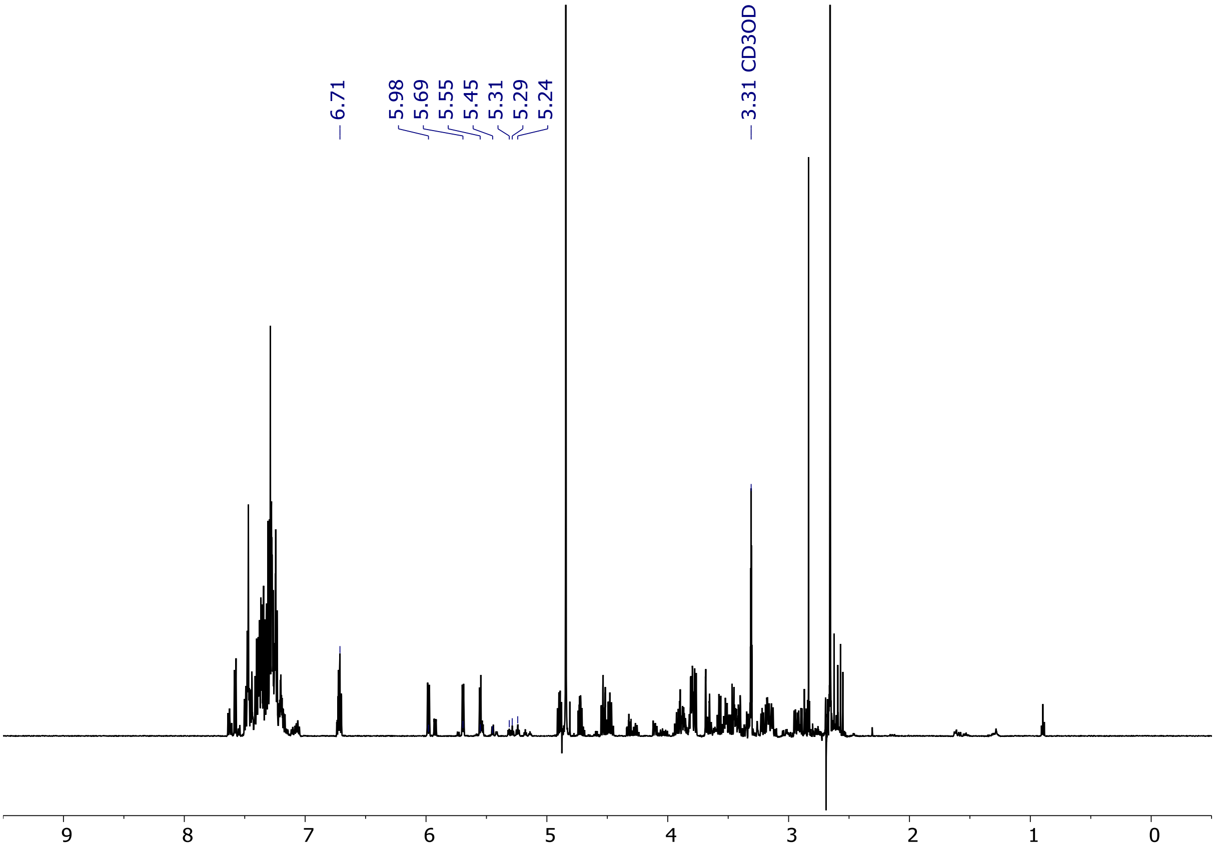


$^{13}C$ NMR, $CD_3OD$, 150 MHz

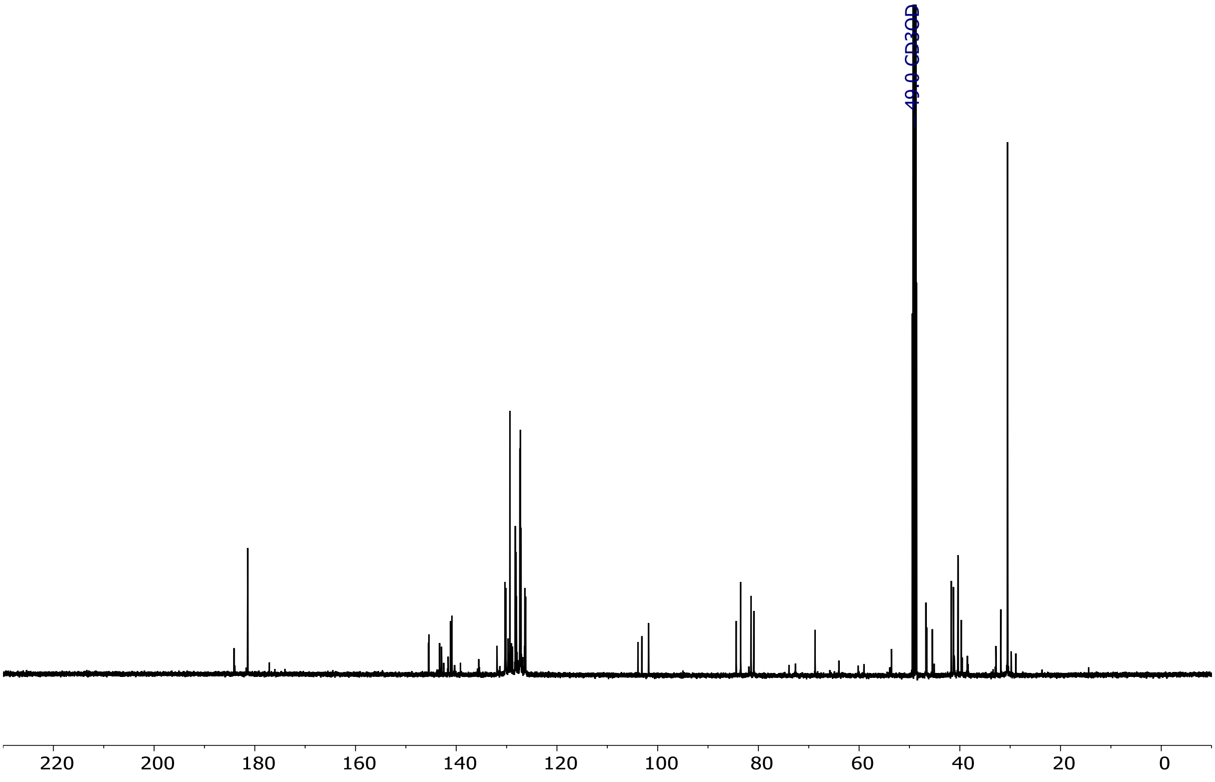

COSY, $CD_3OD$:

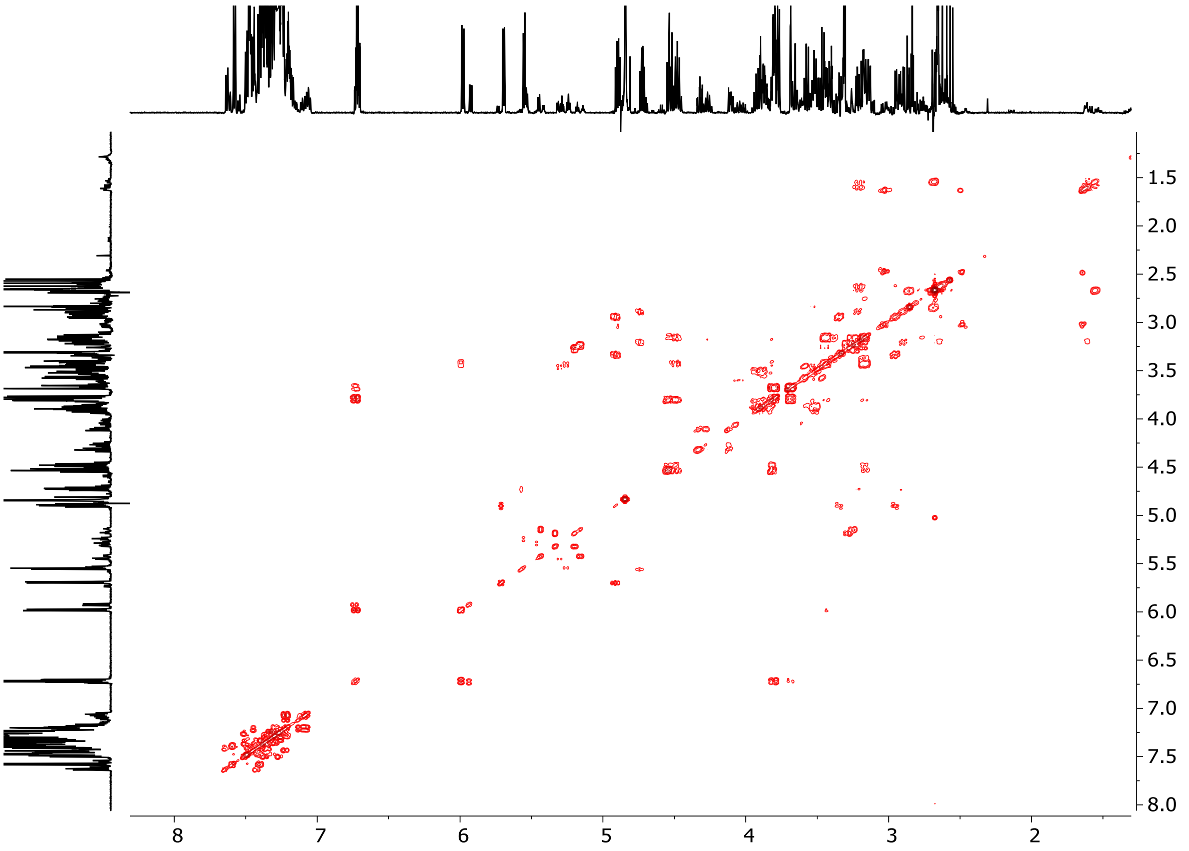


HSQC, $CD_3OD$:

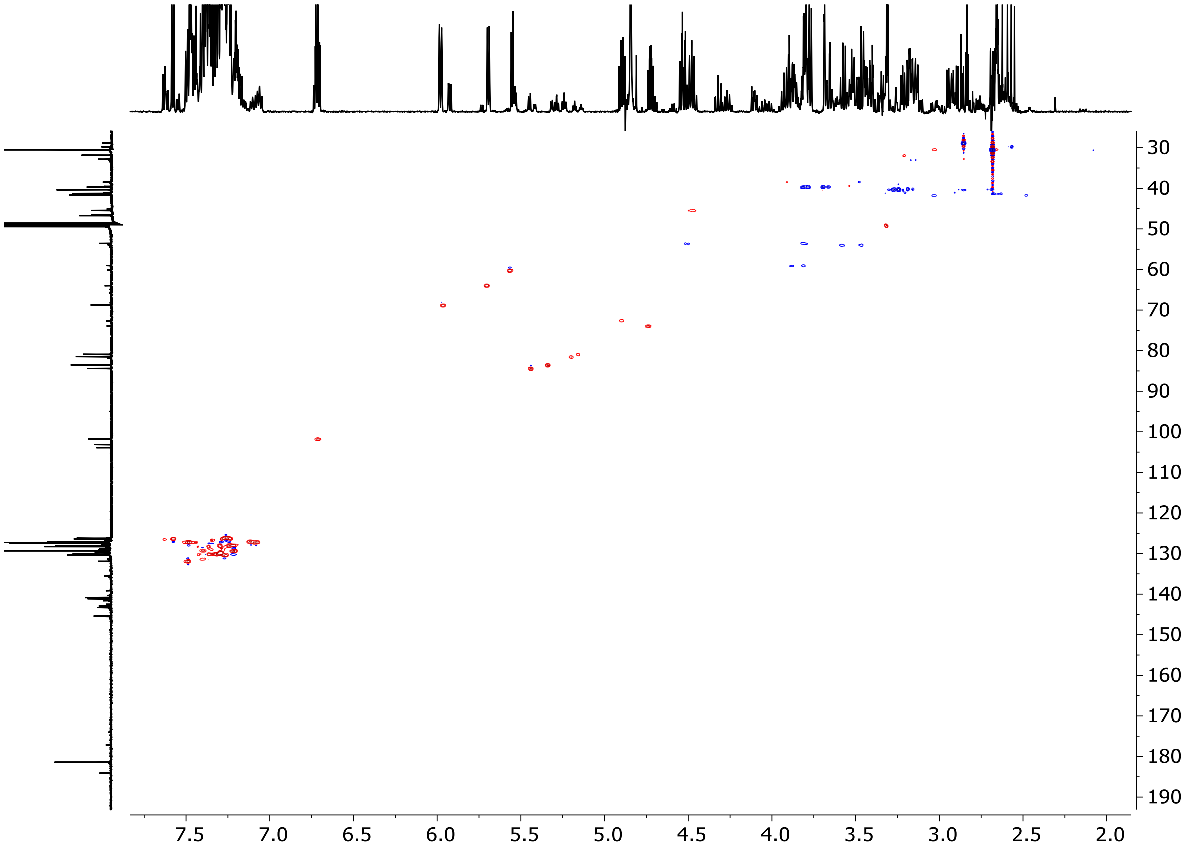

HMBC, CD3OD:

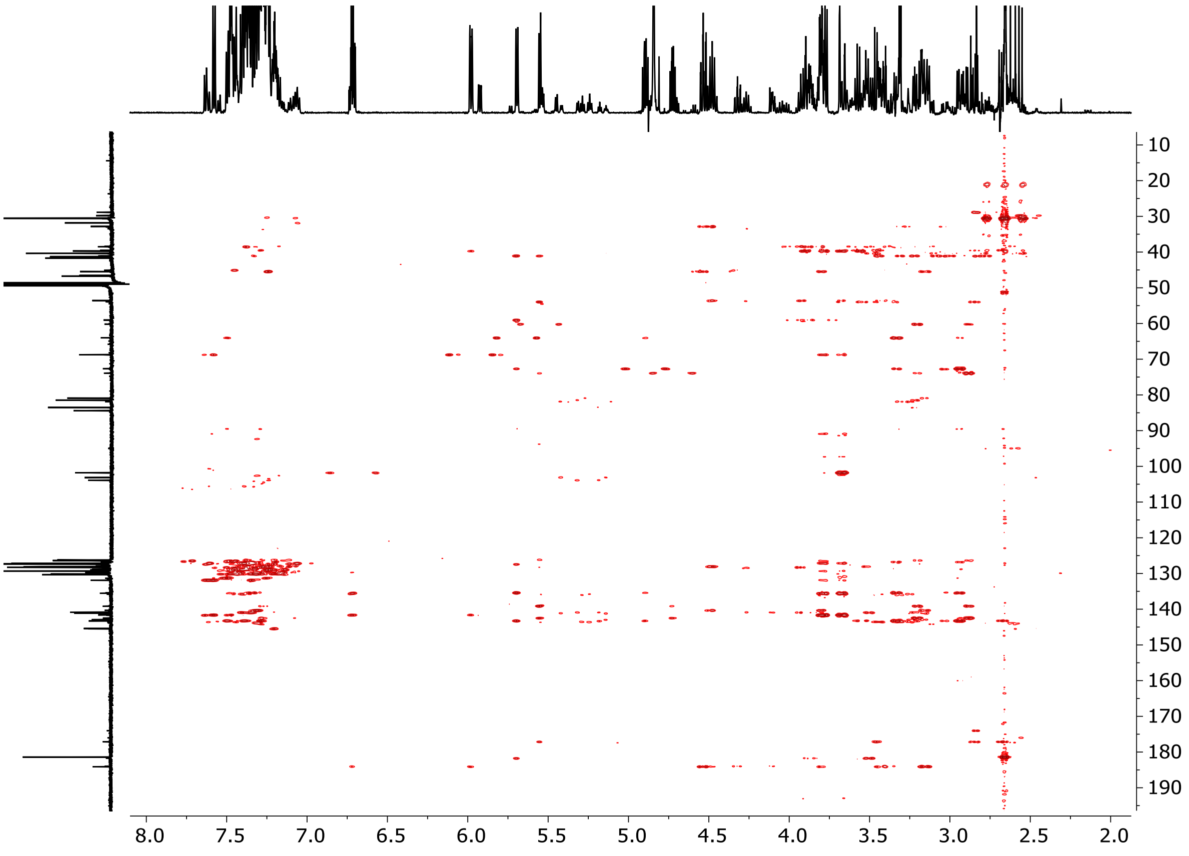

10
20
30
40
50
60
70
80
90
100
110
120
130
140
150
160
170
180
190
8.0
7.5
7.0
6.5
6.0
5.5
5.0
4.5
4.0
3.5
3.0
2.5
2.0

**Clean spectra in d4-MeOH for comparison: [3a]**, $^{1}$H NMR, $CD_3OD$, 600 MHz

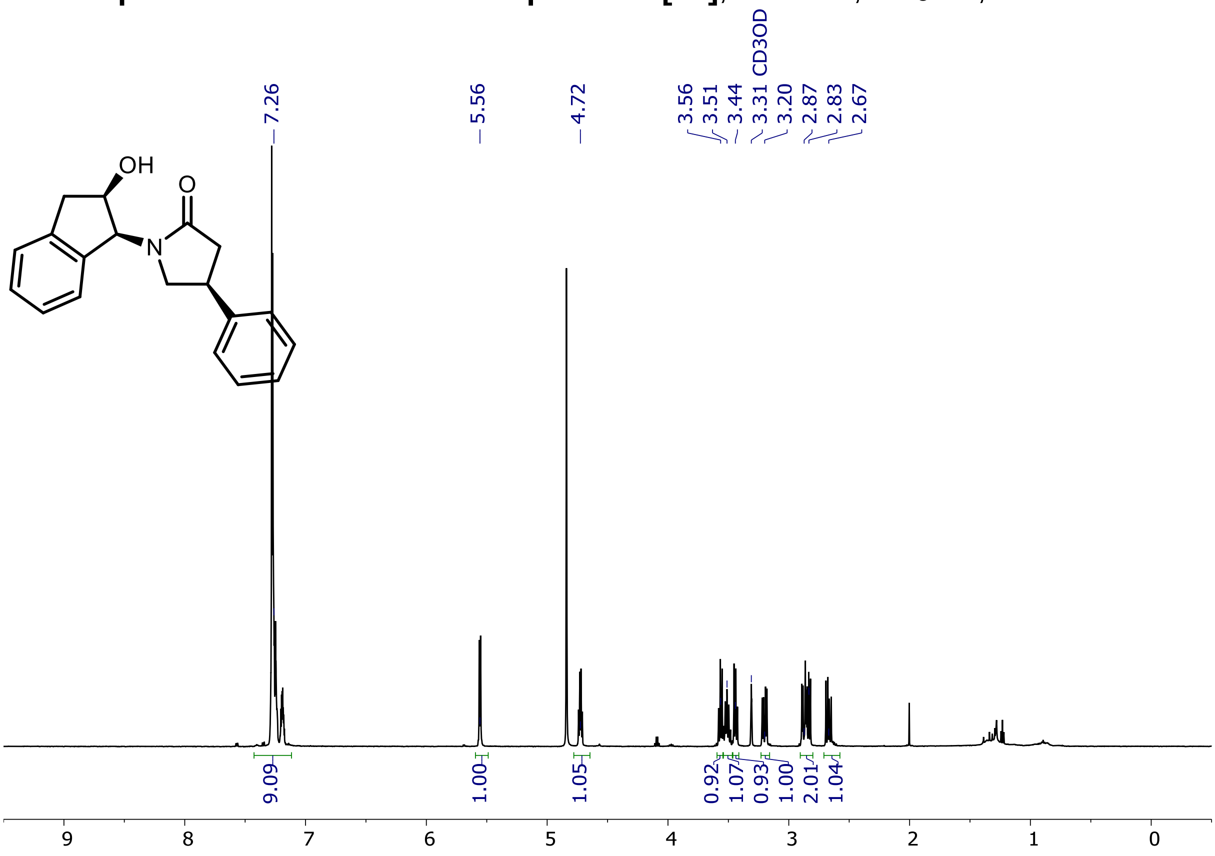


**[3a]**, $^{13}$C NMR, $CD_3OD$, 150 MHz

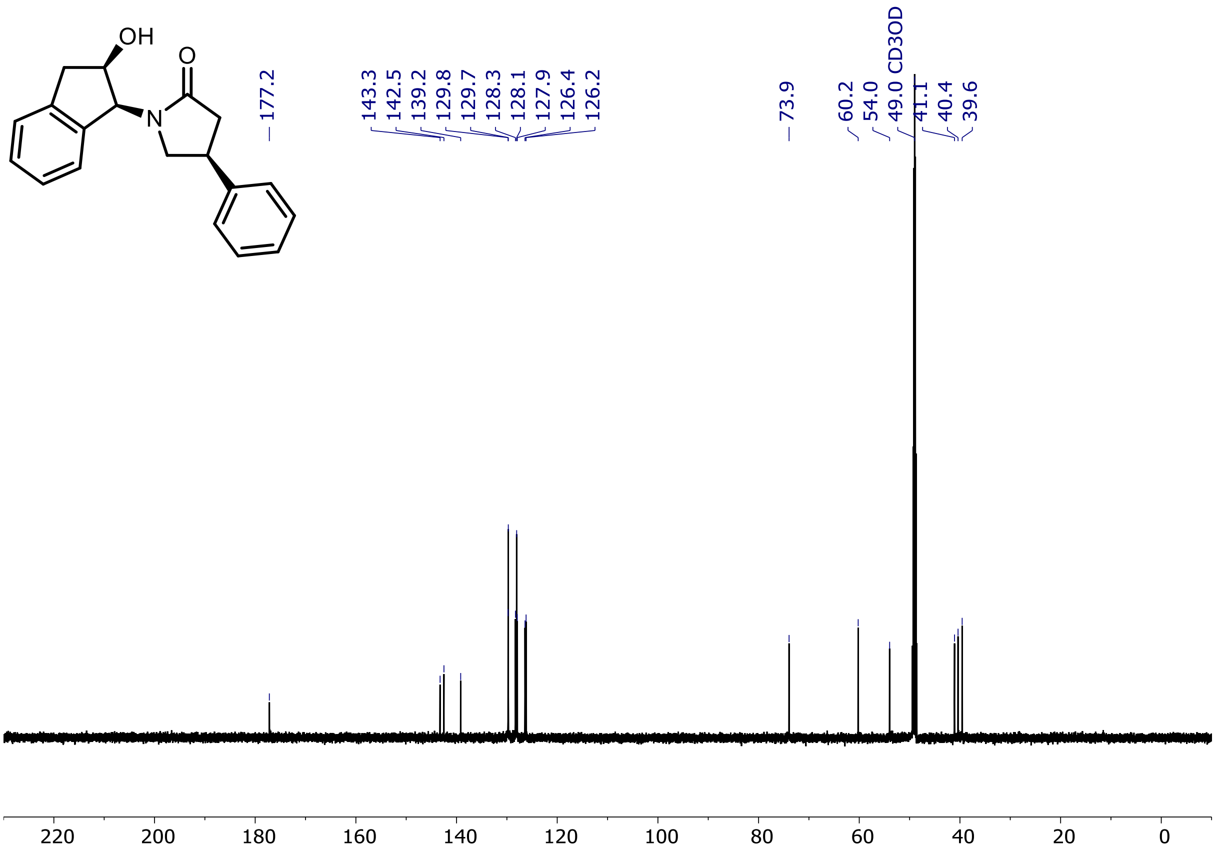

**[*epi*-3a]**, $^{1}$H NMR, $CD_3OD$, 600 MHz

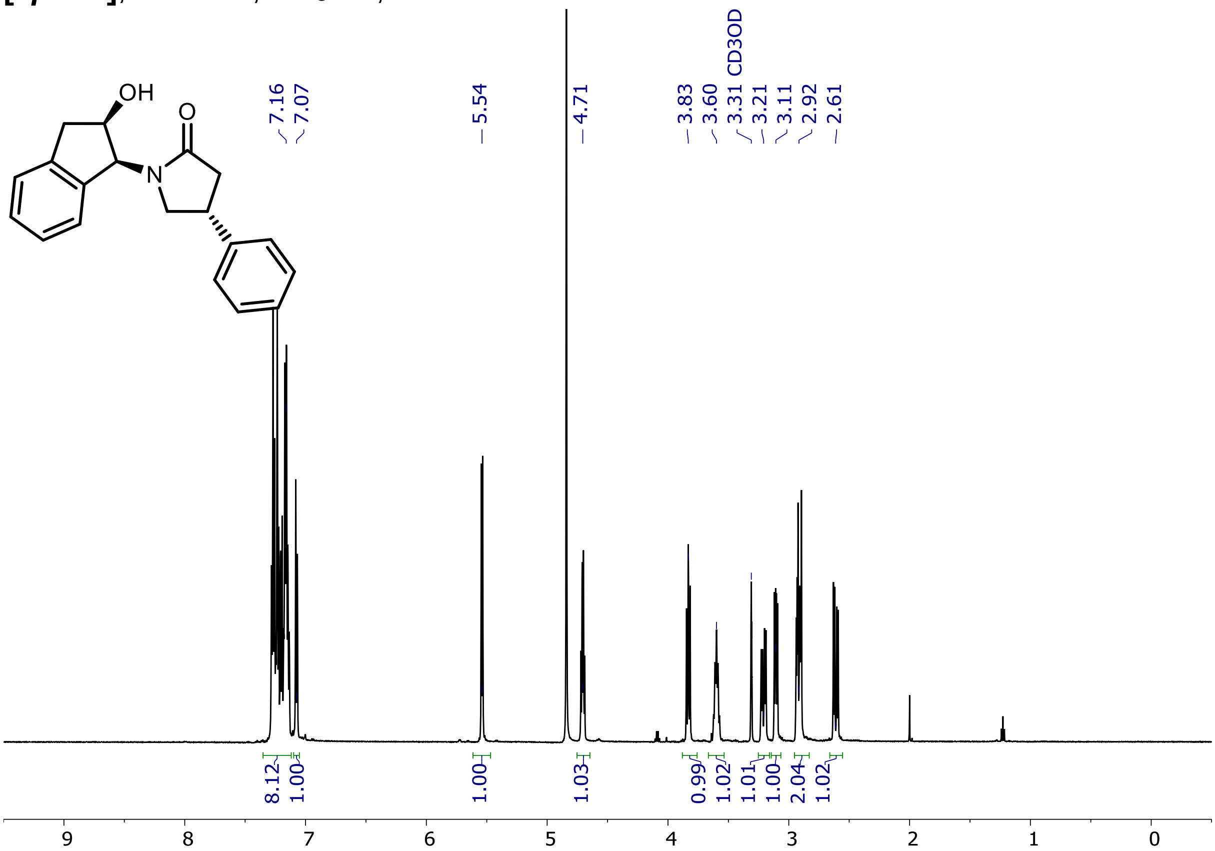


**[*epi*-3a]**, $^{13}$C NMR, $CD_3OD$, 150 MHz

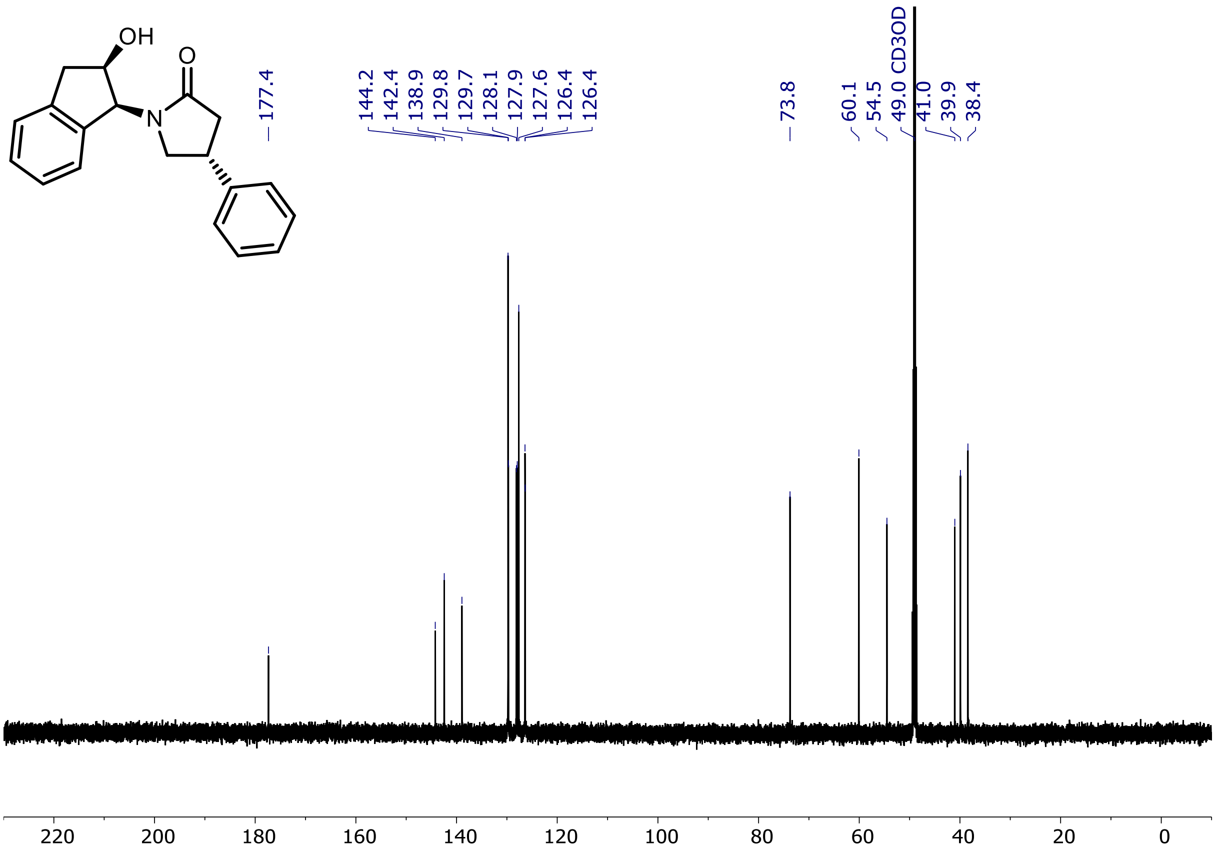

**[S4, *epi*-S4]**, $^{1}$H NMR, $CD_3OD$, 600 MHz

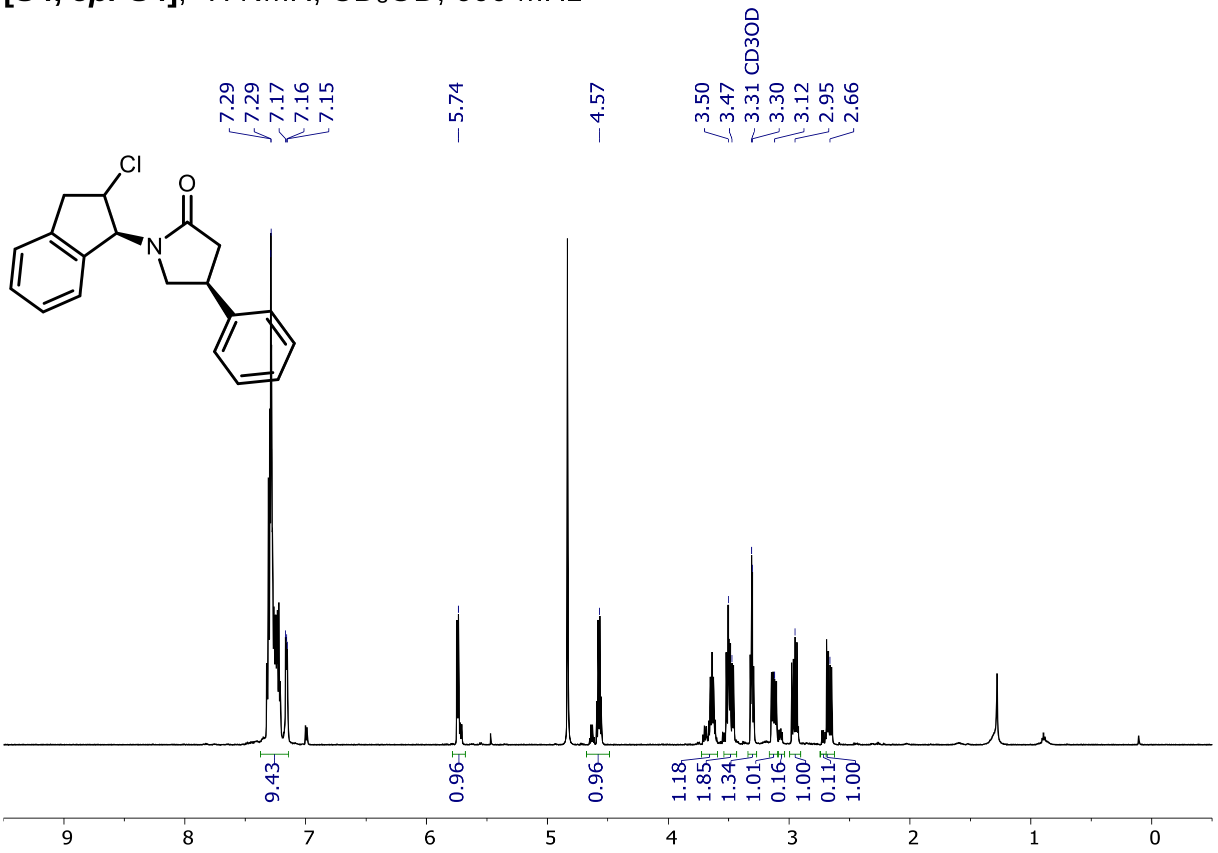


**[S4, *epi*-S4],** $^{13}$C NMR NMR, $CD_3OD$, 150 MHz

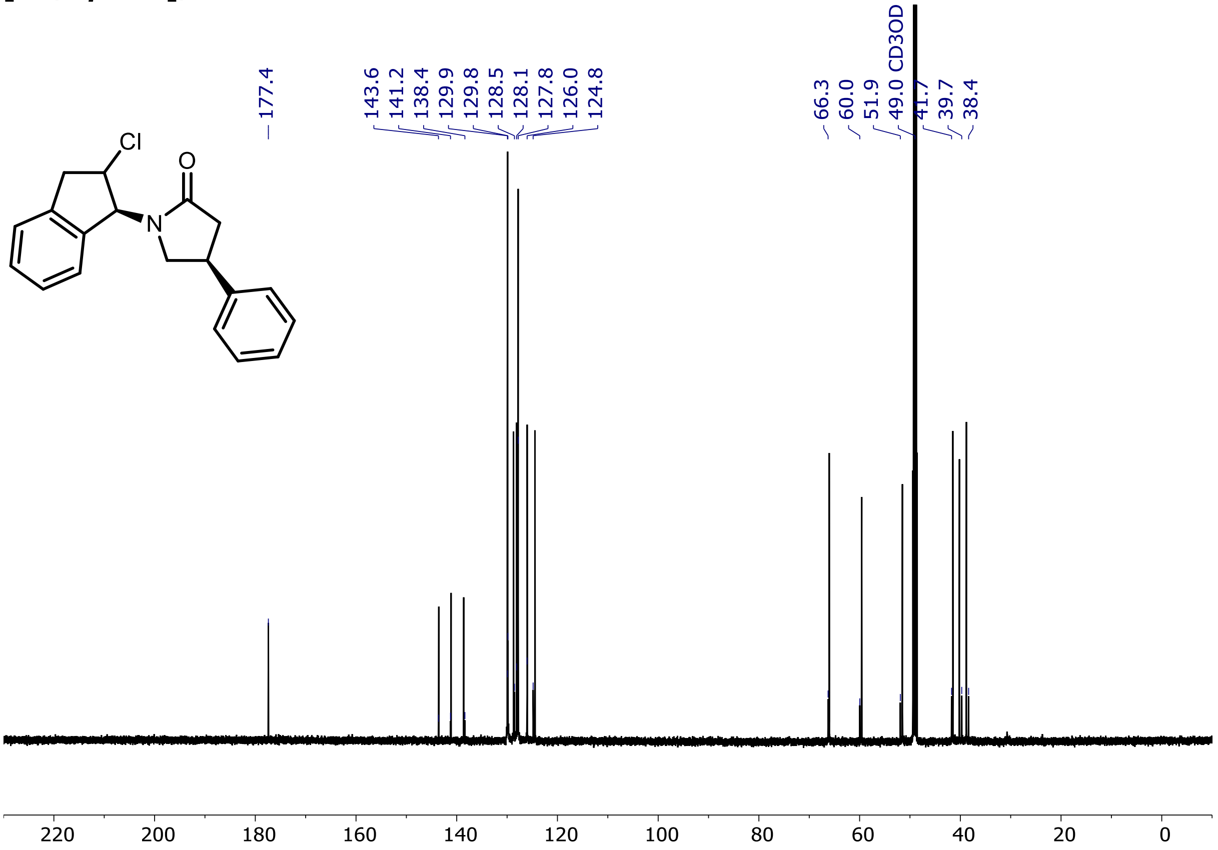

**[3a]**, $^{1}H$ NMR, $CDCl_3$, 600 MHz

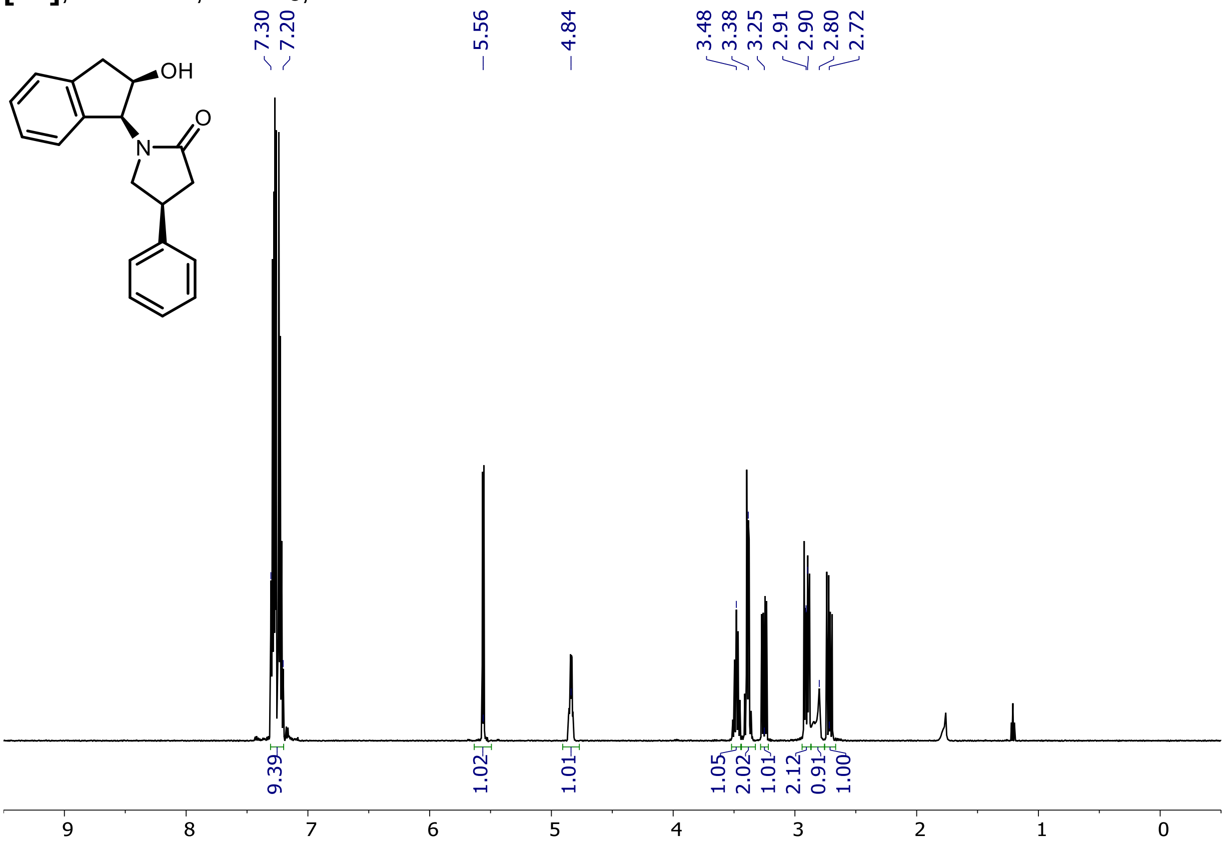


**[3a]**, $^{13}C$ NMR, $CDCl_3$, 151 MHz

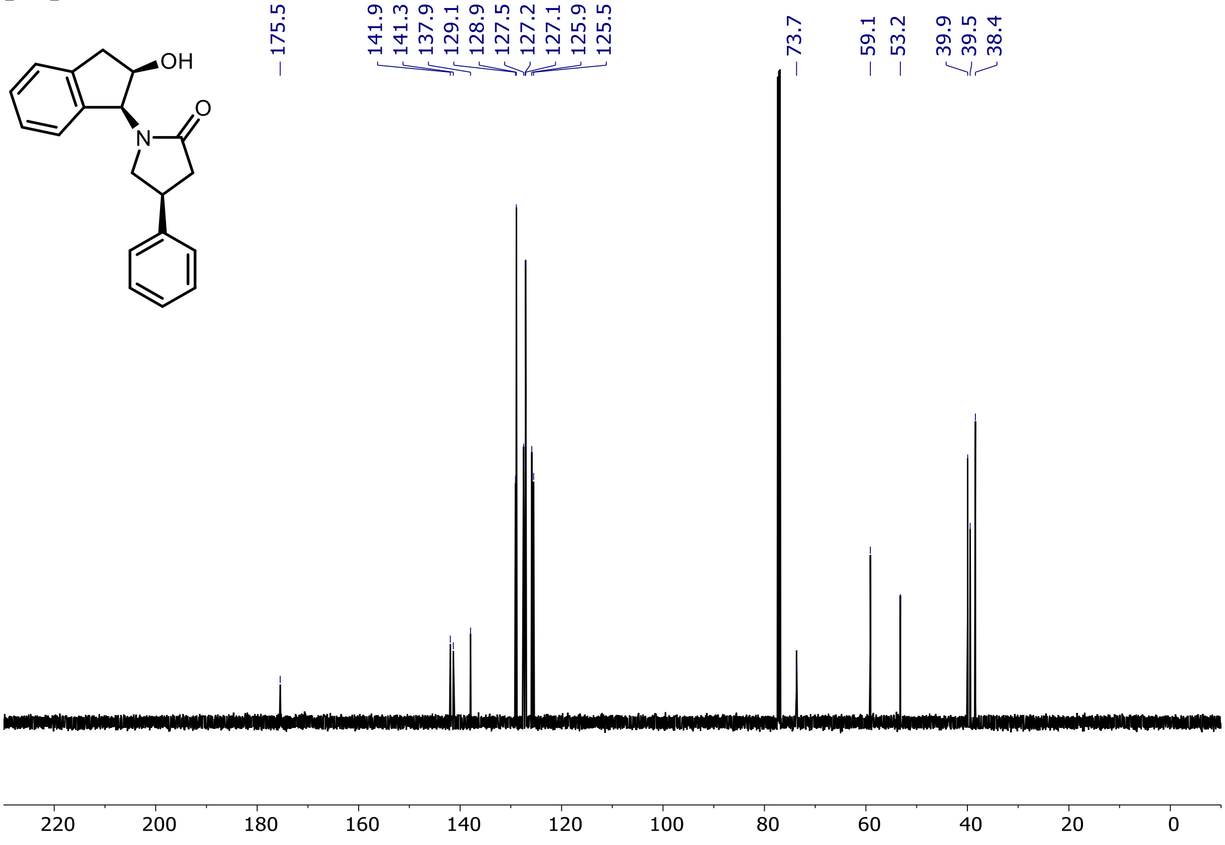

[*epi*-3a], $^{1}H$ NMR, $CDCl_3$, 500 MHz

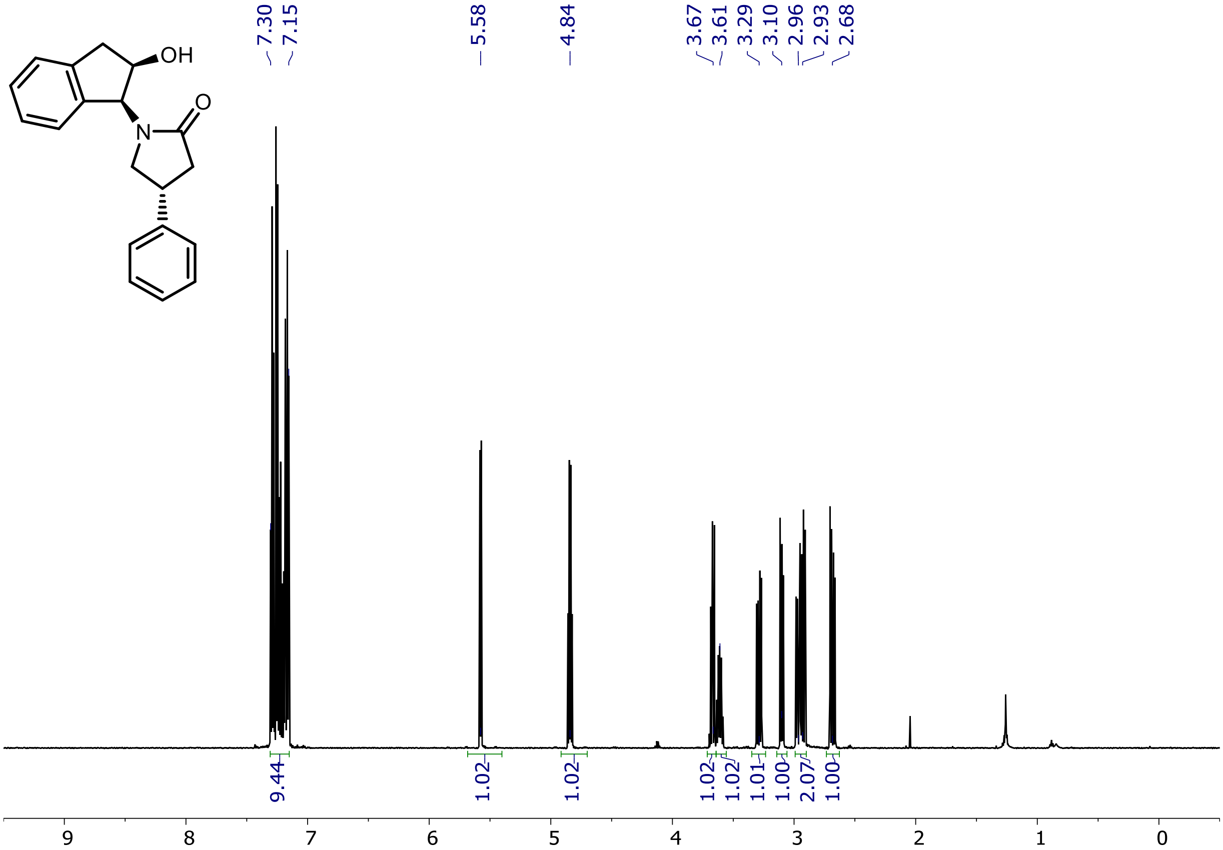


[*epi*-3a], $^{13}C$ NMR, $CDCl_3$, 150 MHz

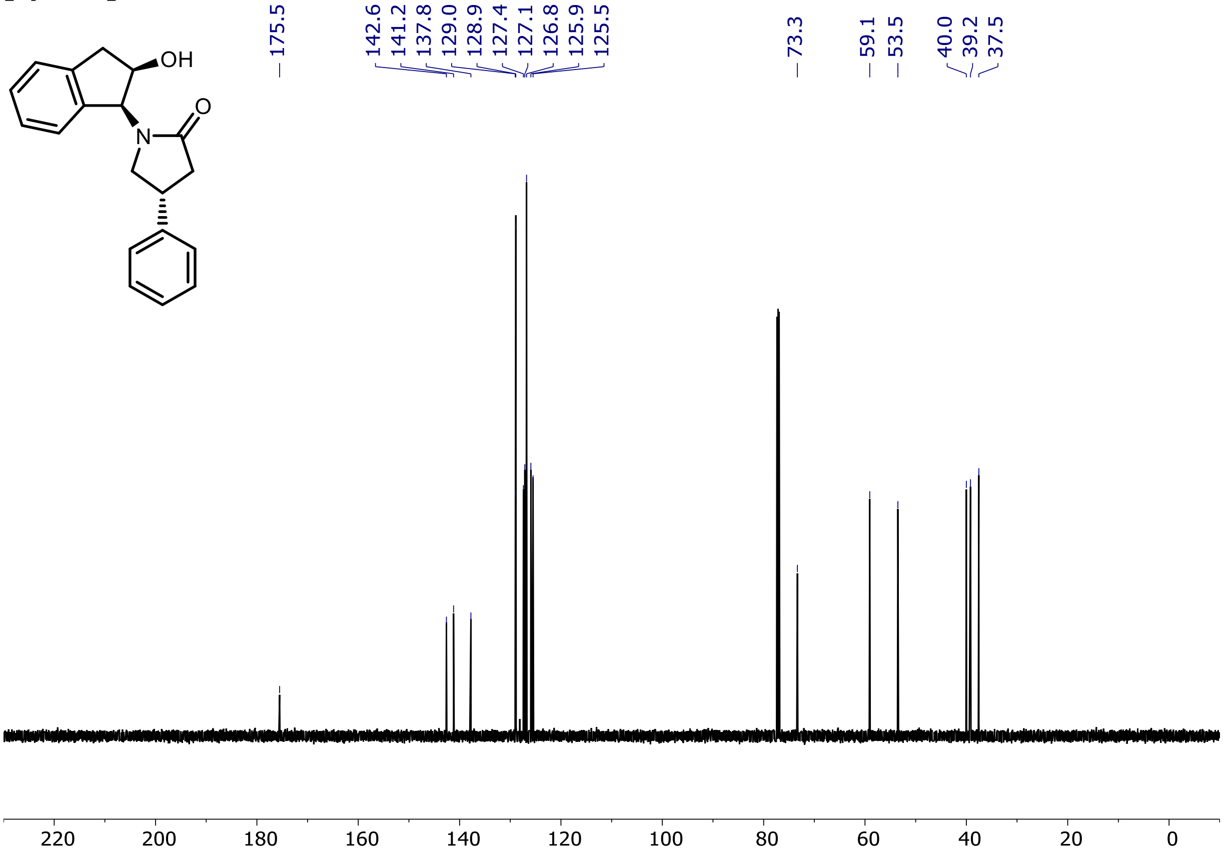

**[3b]**, $^{1}$H NMR, $CDCl_3$, 500 MHz

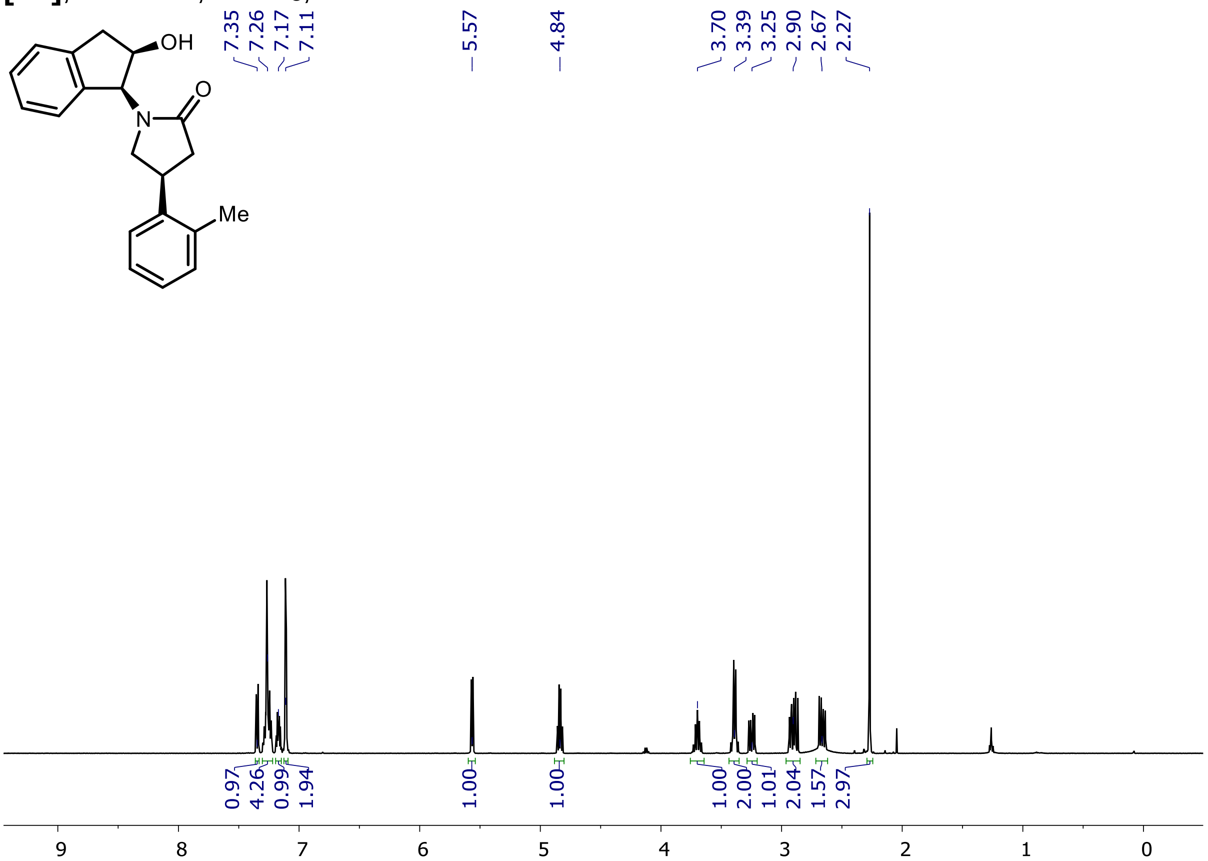


**[3b]**, $^{13}$C NMR, $CDCl_3$, 126 MHz

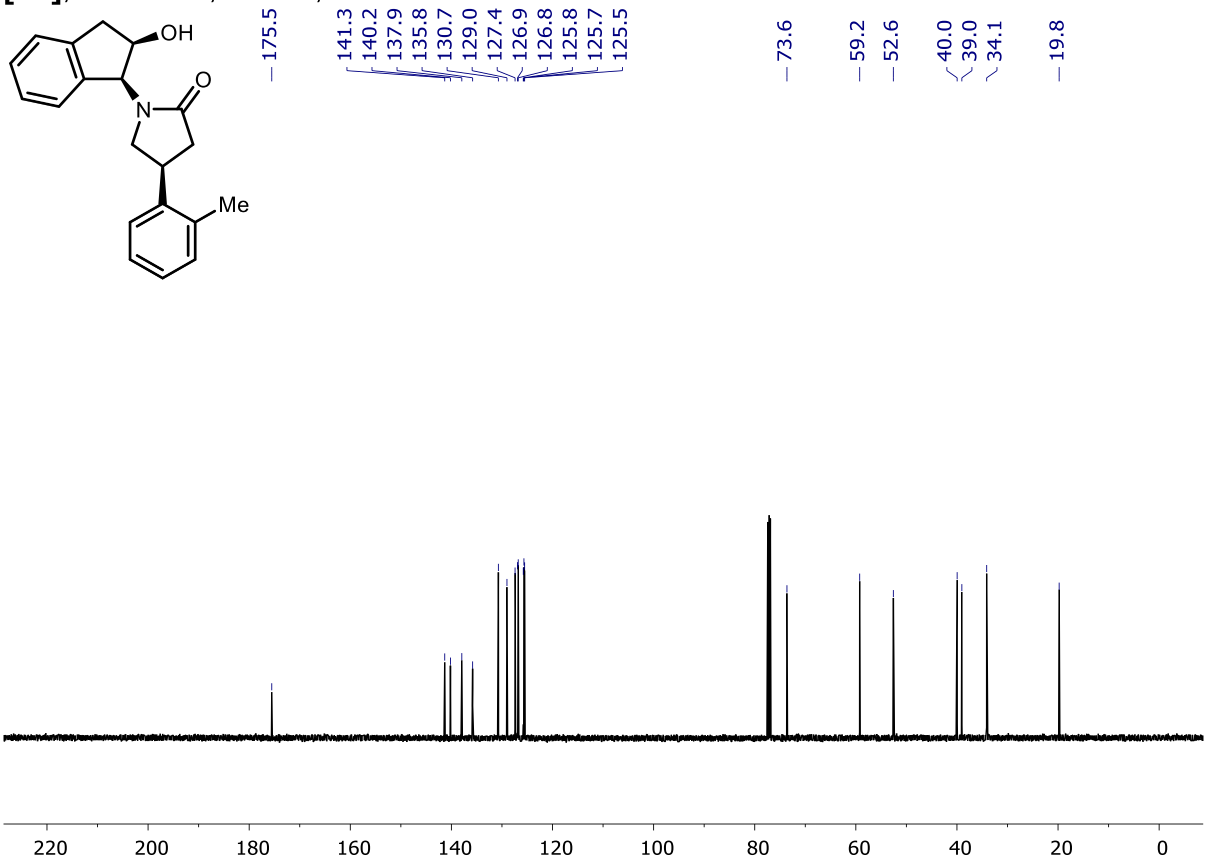

**[*epi*-3b]**, $^{1}$H NMR, $CDCl_3$, 600 MHz

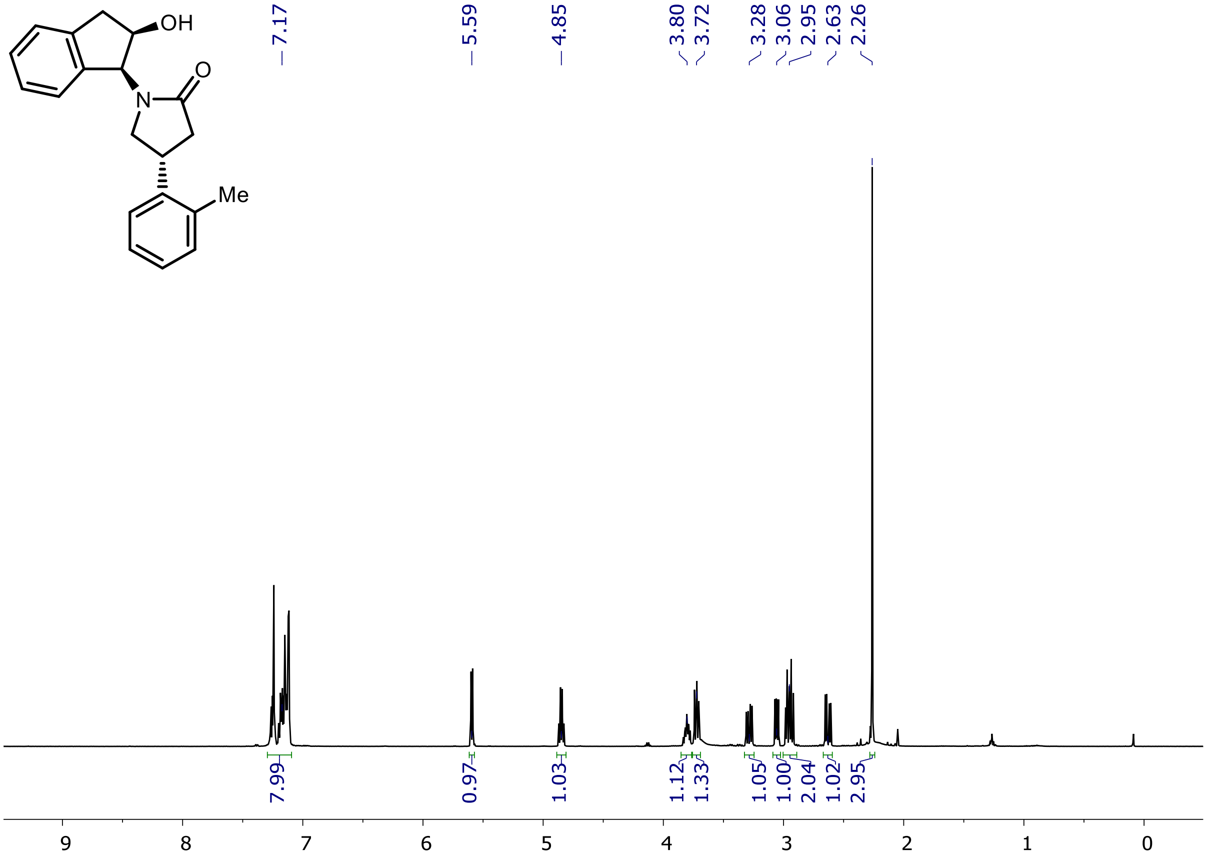


**[*epi*-3b]**, $^{13}$C NMR, $CDCl_3$, 126 MHz

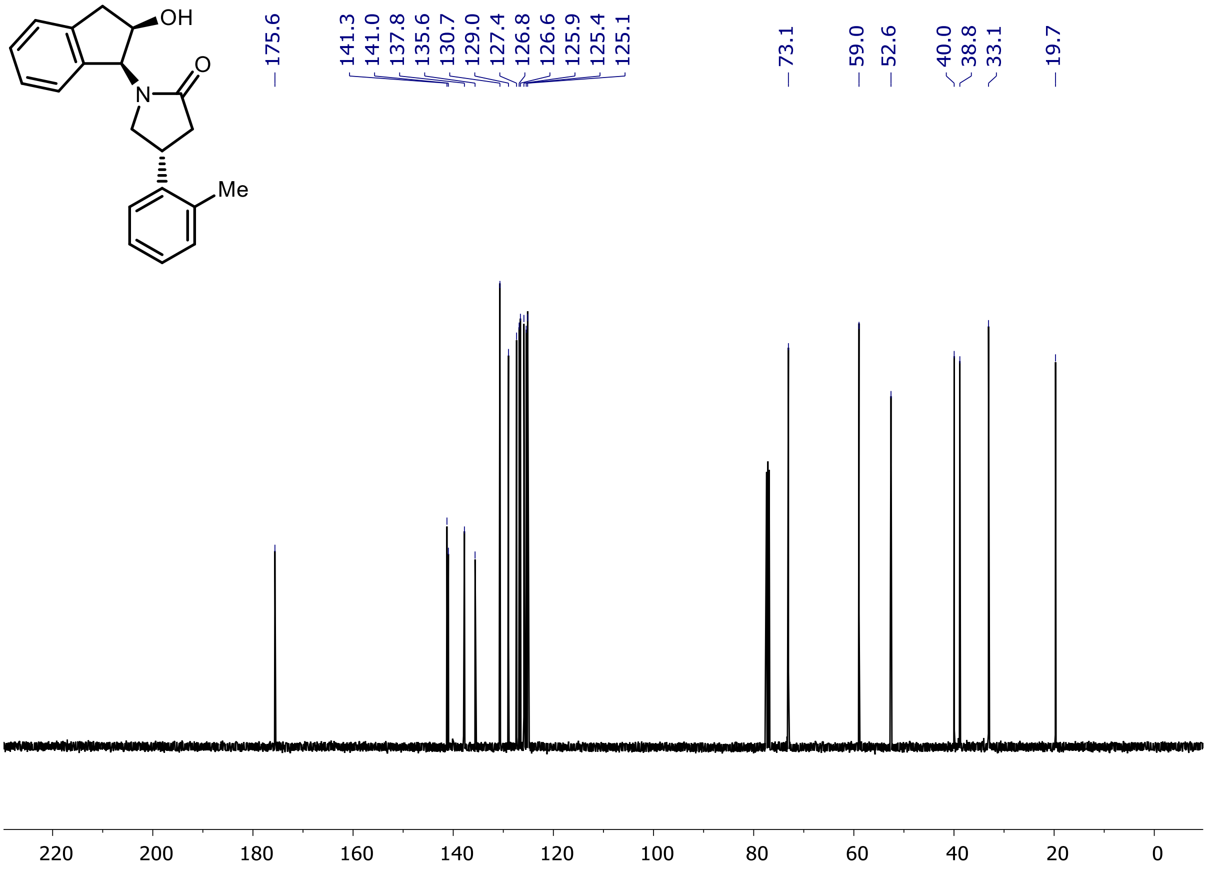

**[3c]**, $^{1}$H NMR, $CDCl_3$, 600 MHz

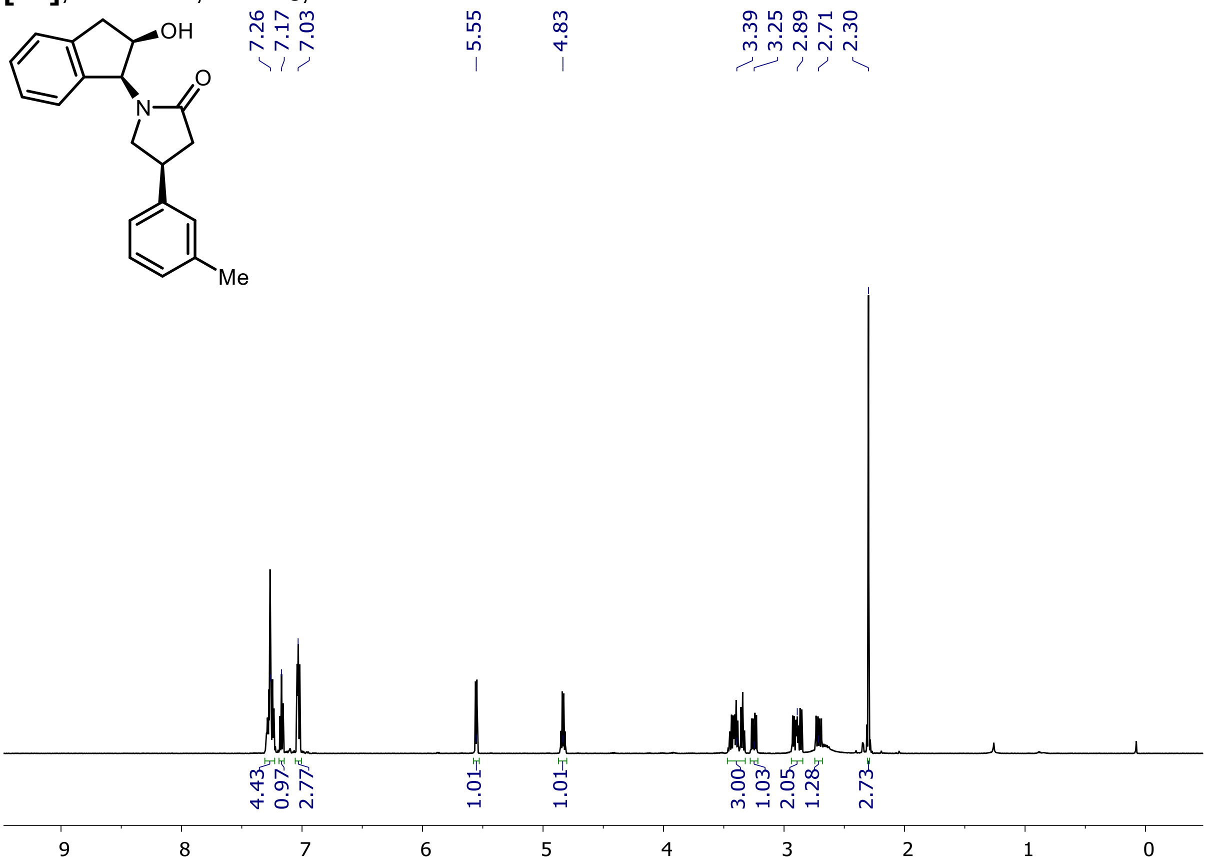


**[3c]**, $^{13}$C NMR, $CDCl_3$, 151 MHz

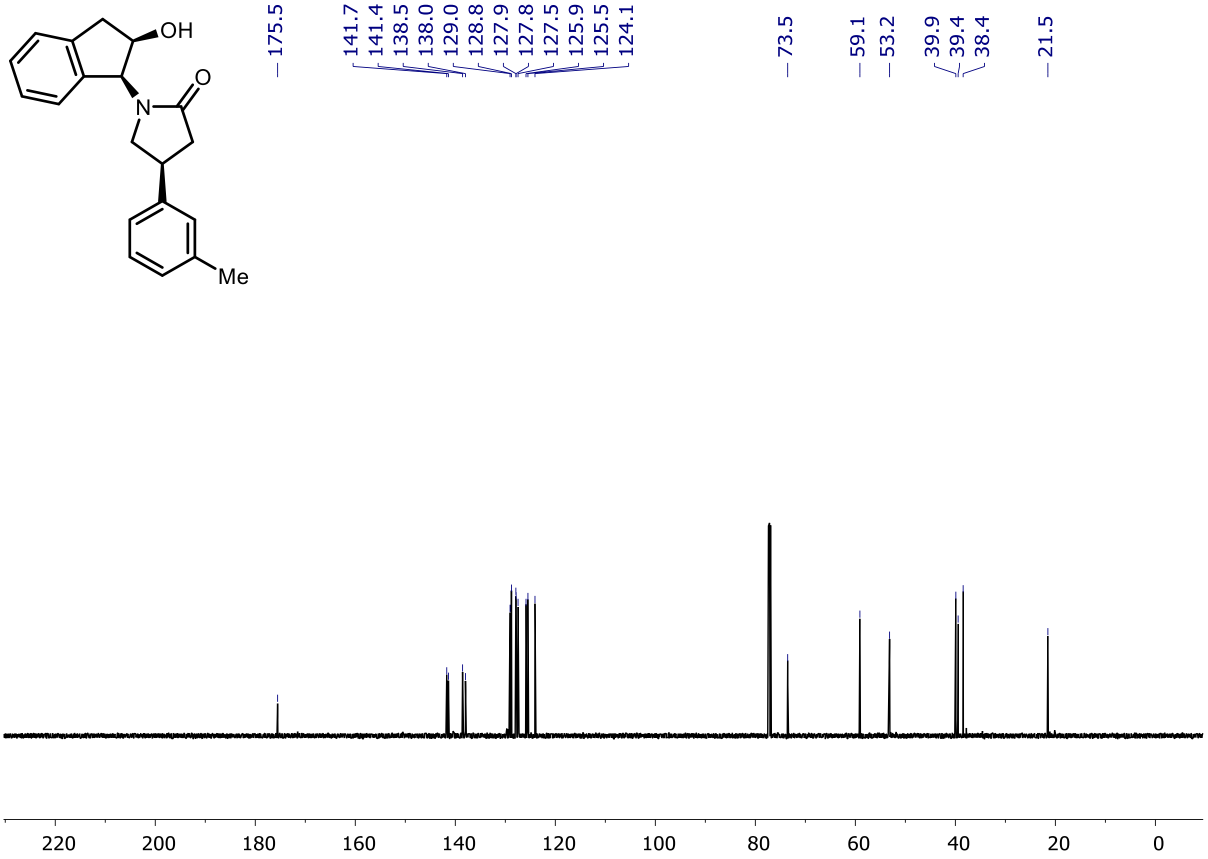

**[*epi*-3c]**, $^{1}H$ NMR, $CDCl_3$, 600 MHz

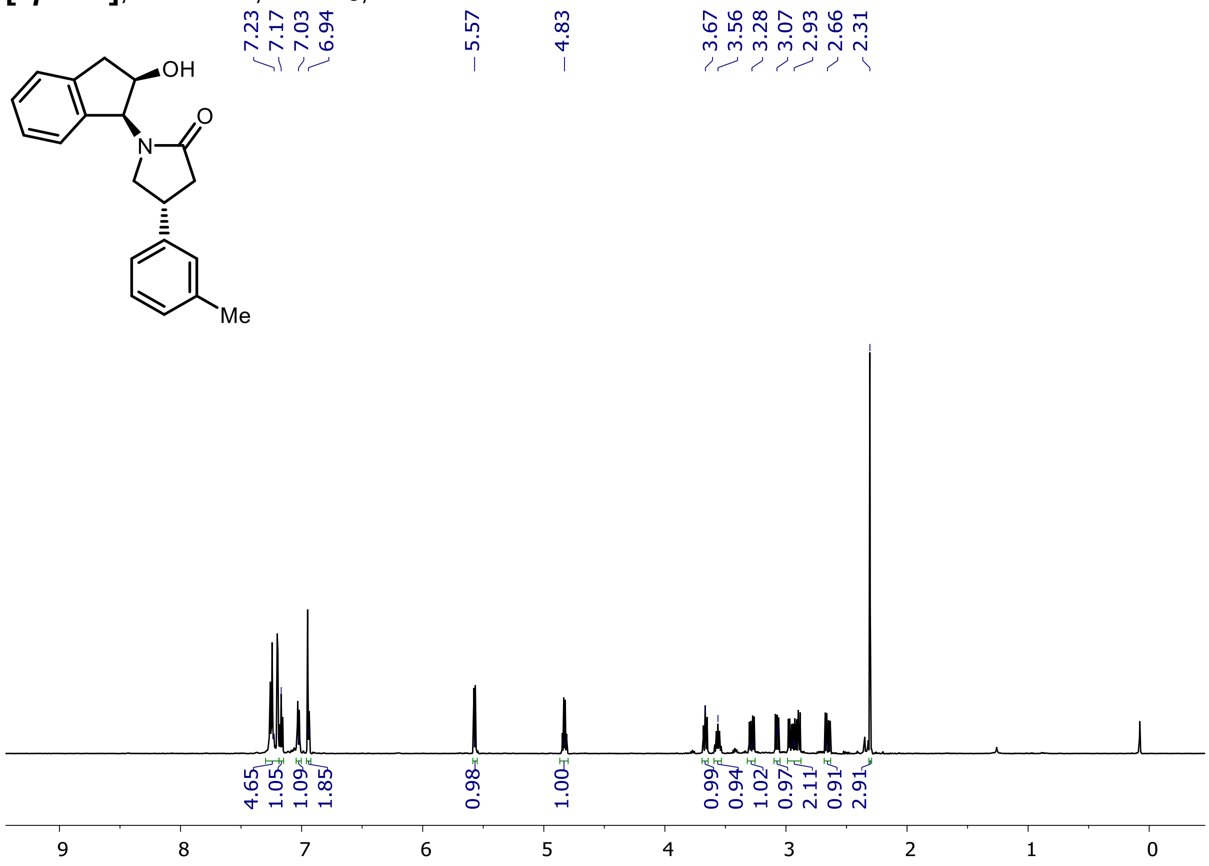


**[*epi*-3c]**, $^{13}C$ NMR, $CDCl_3$, 151 MHz

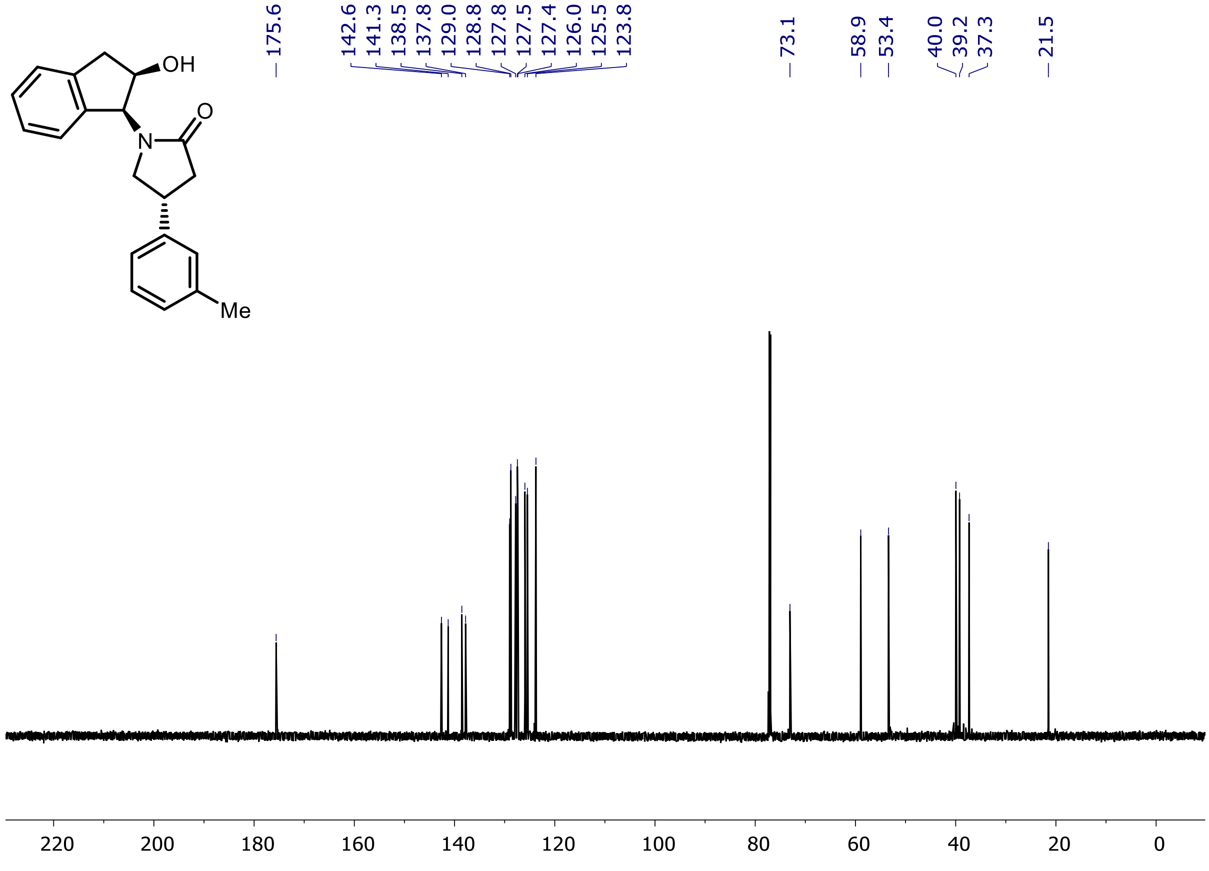

**[3d]**, $^{1}$H NMR, $CDCl_3$, 500 MHz

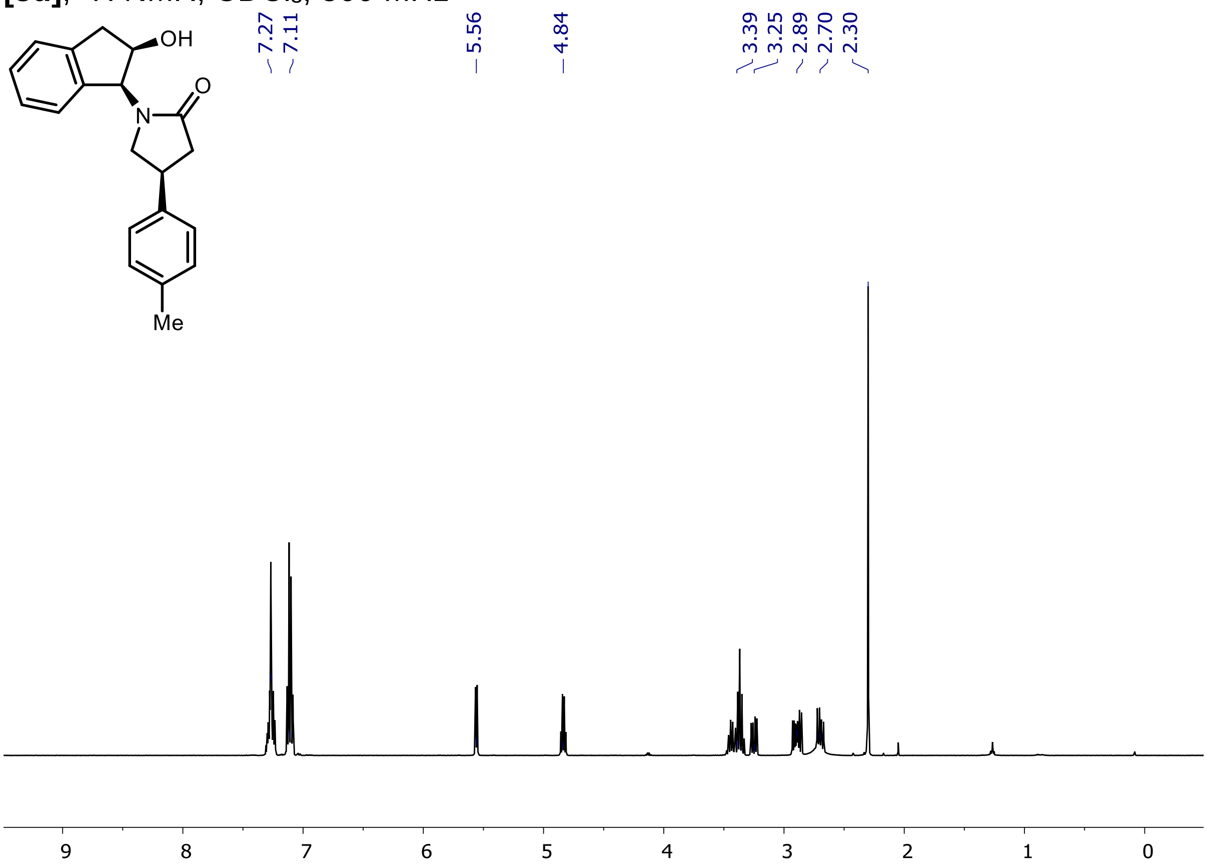


**[3d]**, $^{13}$C NMR, $CDCl_3$, 126 MHz

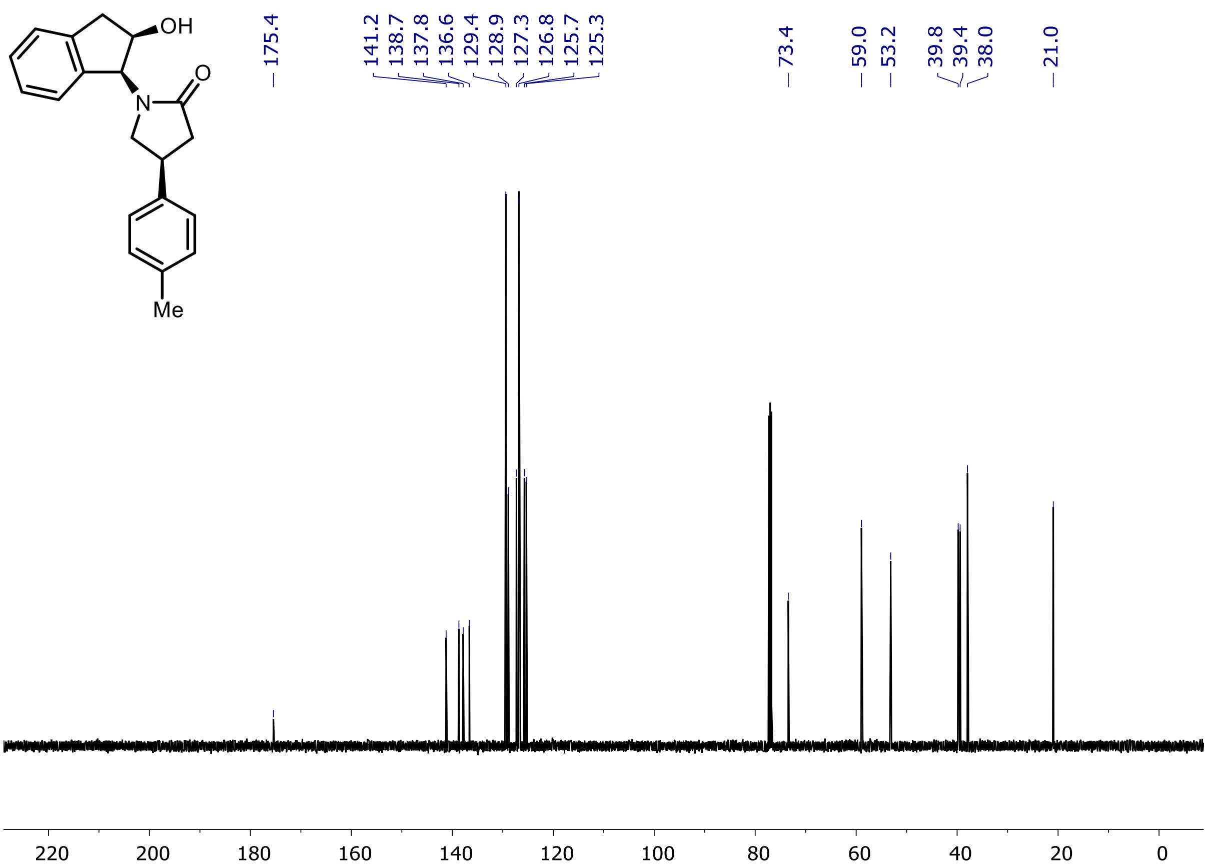

**[*epi*-3d]**, $^{1}$H NMR, CDCl$_3$, 500 MHz

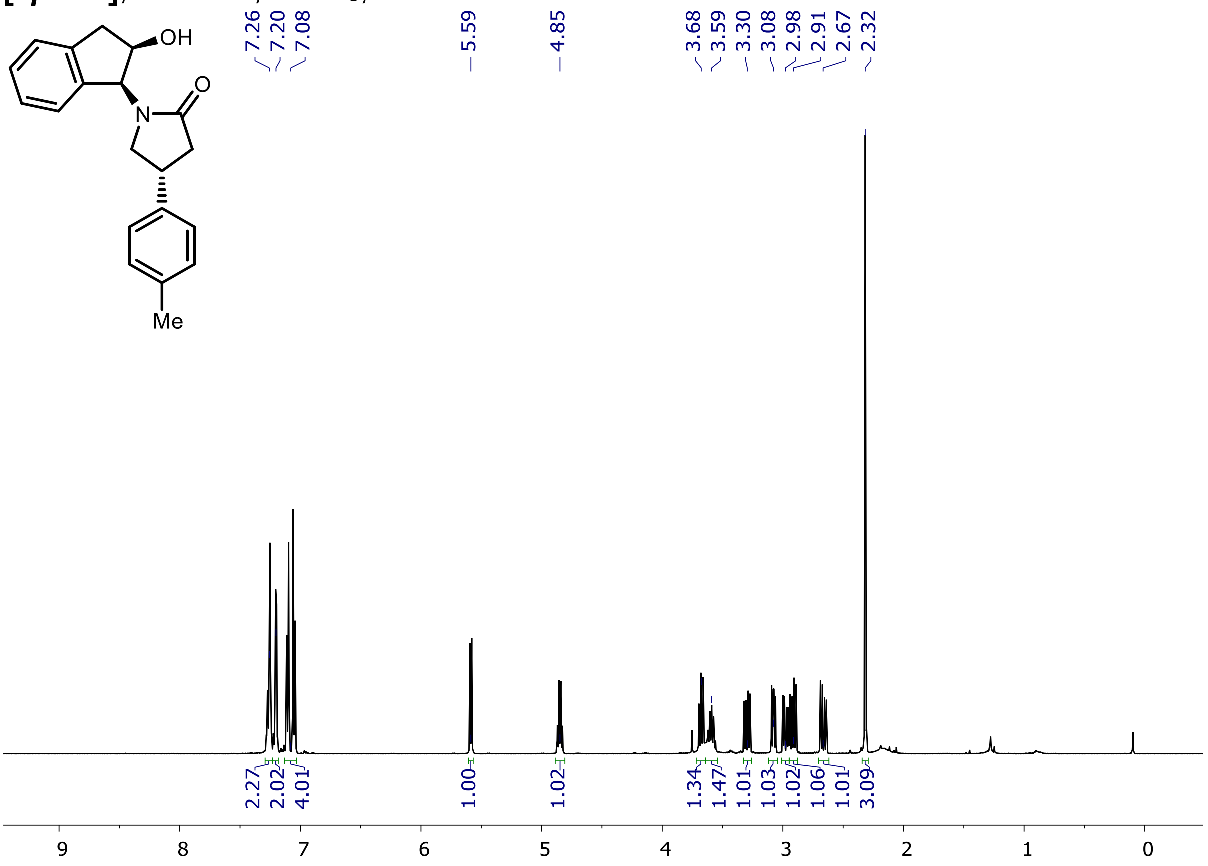


**[*epi*-3d]**, $^{13}$C NMR, CDCl$_3$, 126 MHz

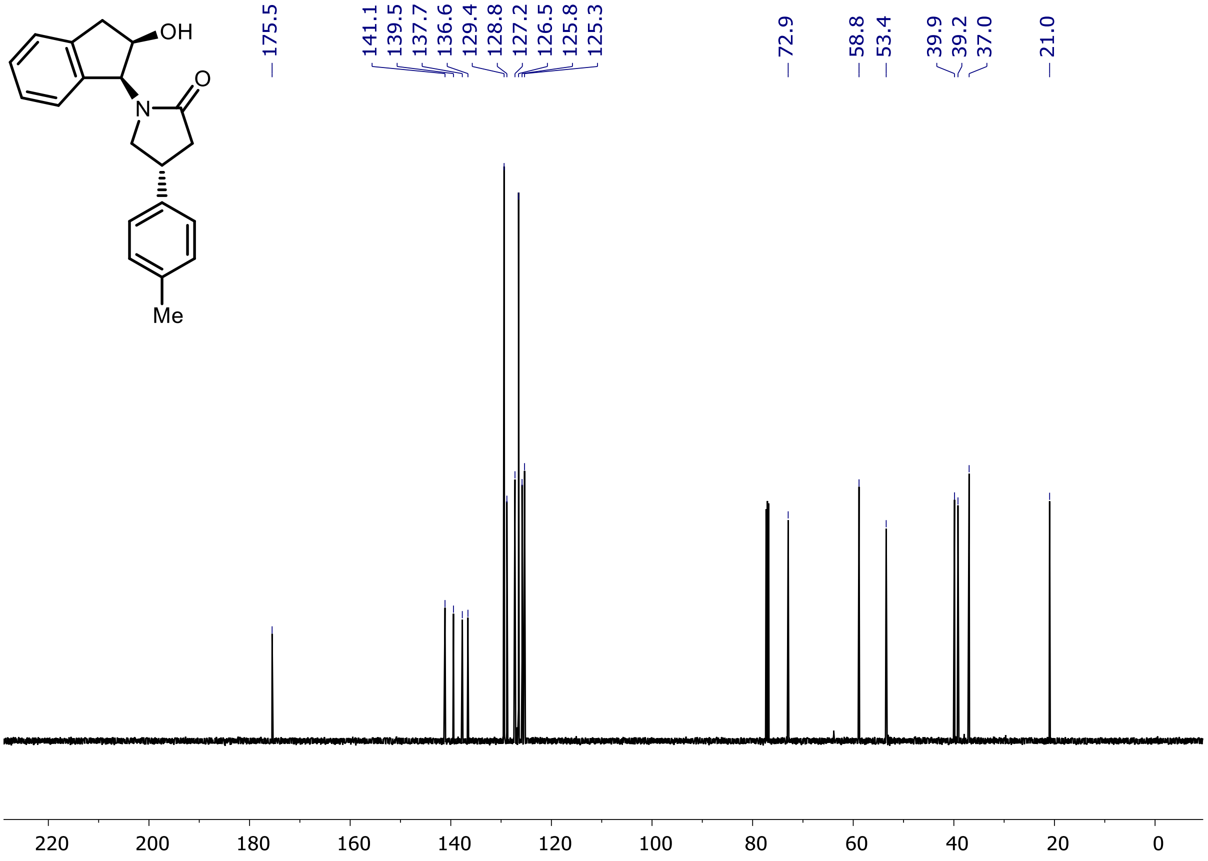

[3e], $^{1}H\{^{19}F\}$ NMR, $CDCl_3$, 500 MHz

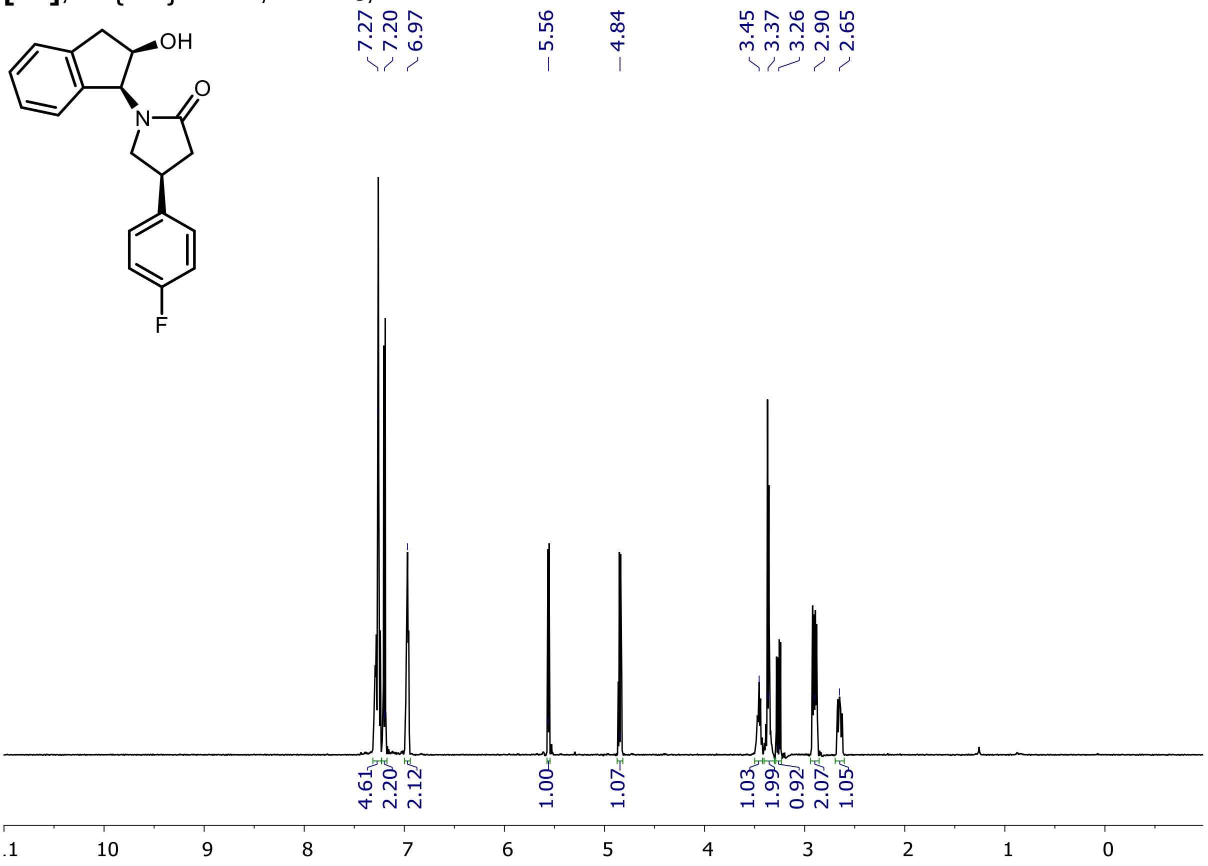


[3e], $^{13}C\{^{19}F\}$ NMR, $CDCl_3$, 126 MHz

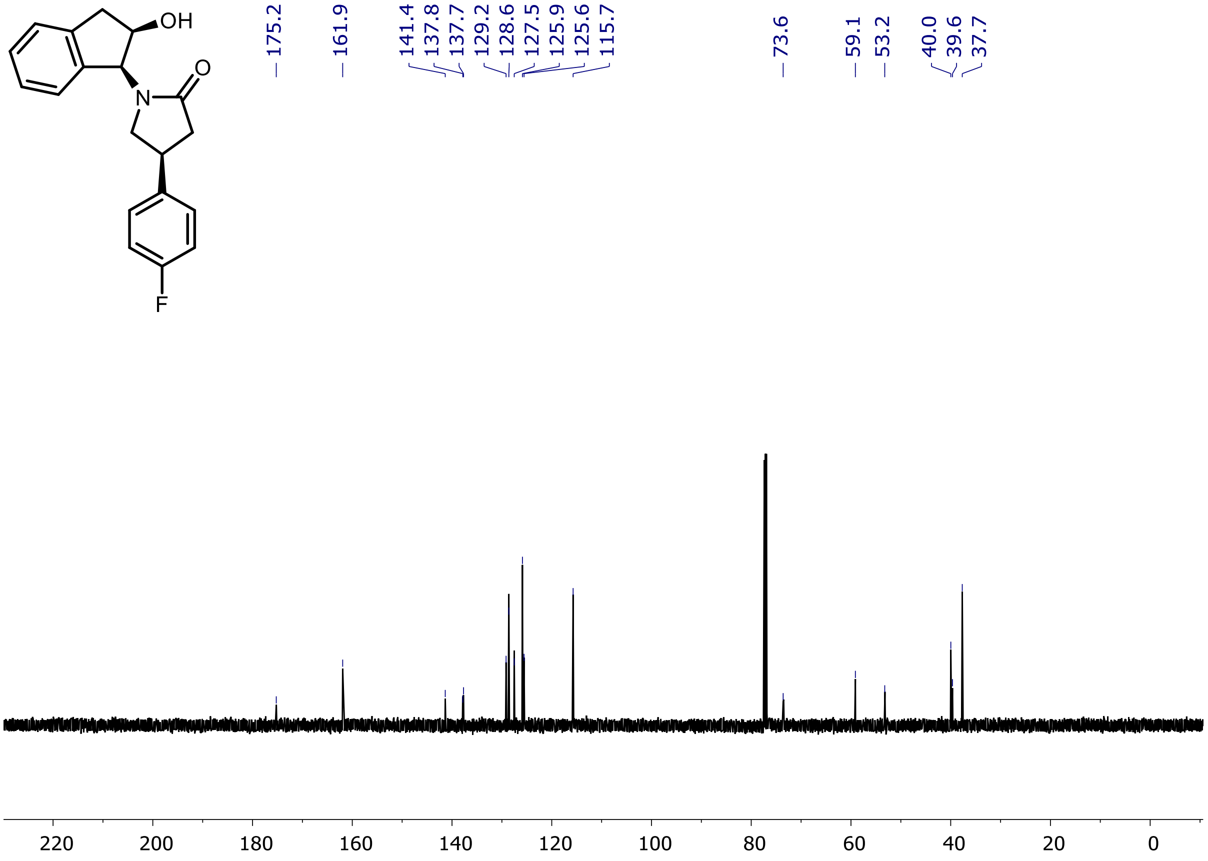

**[*epi*-3e]**, $^{1}H\{^{19}F\}$ NMR, $CDCl_3$, 500 MHz

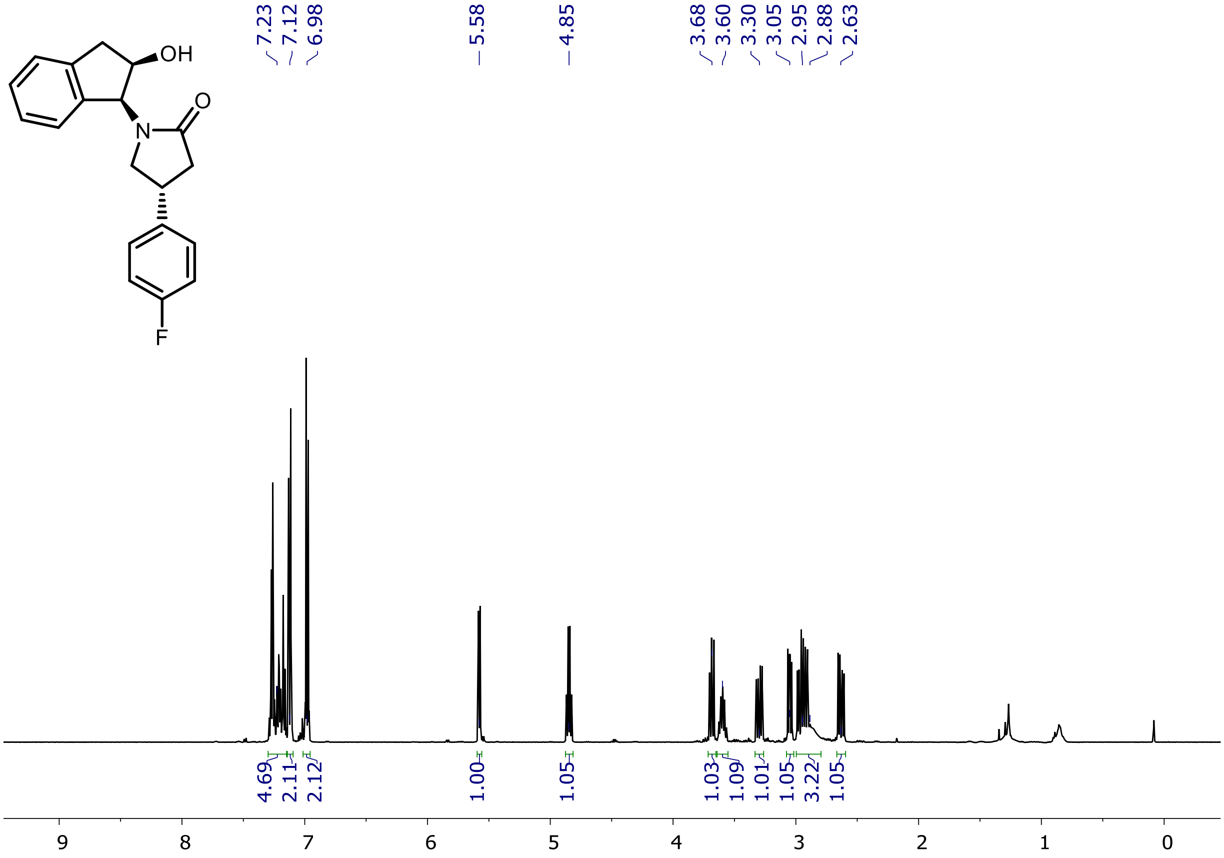


**[*epi*-3e]**, $^{13}C\{^{19}F\}$ NMR, $CDCl_3$, 126 MHz

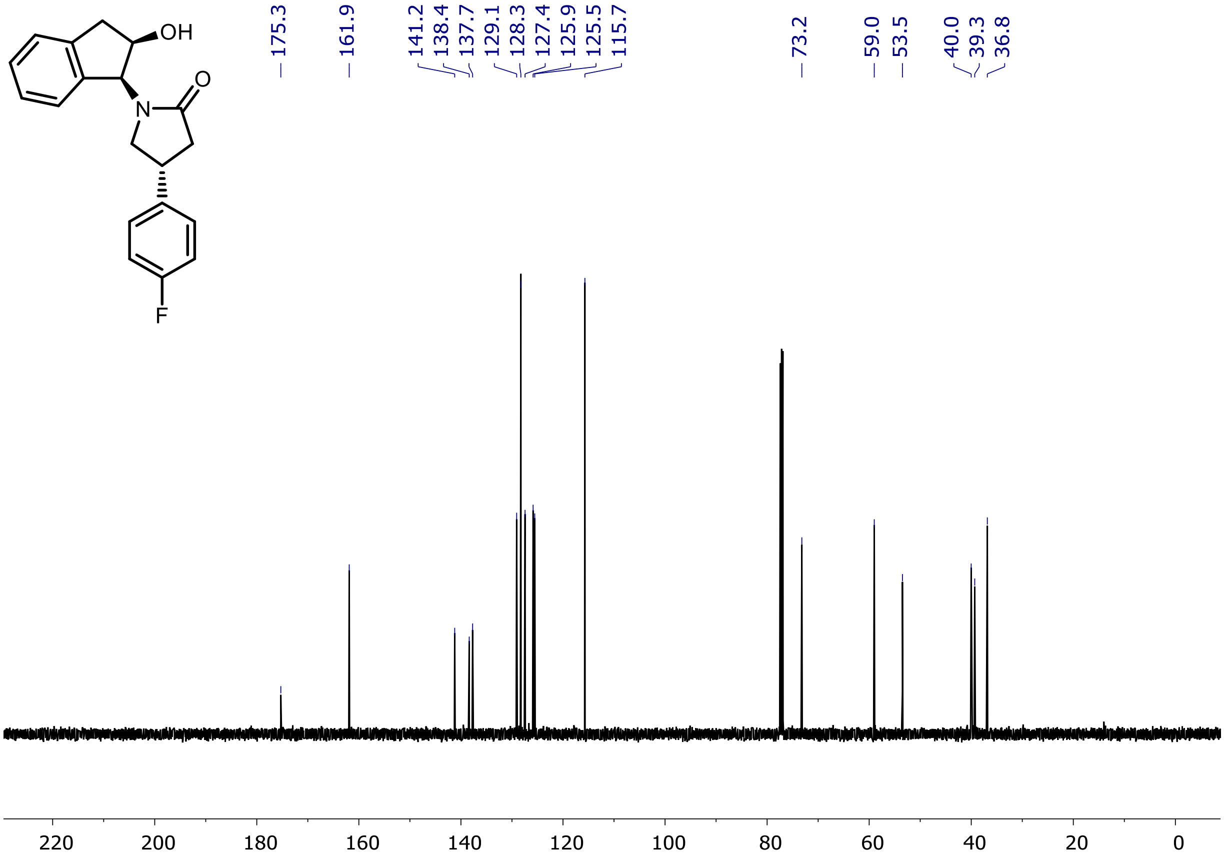

**[3f]**, $^{1}$H NMR, CDCl$_3$, 600 MHz

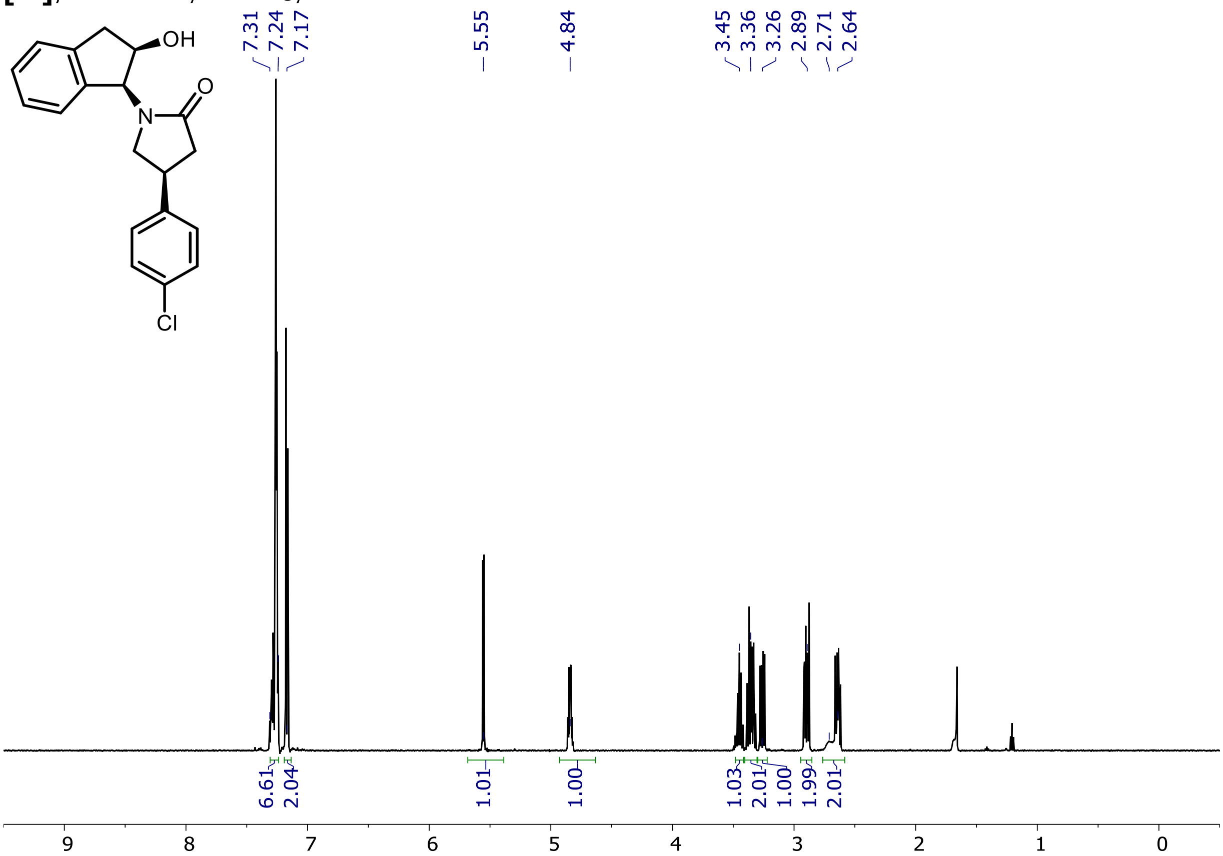


**[3f]**, $^{13}$C NMR, CDCl$_3$, 151 MHz

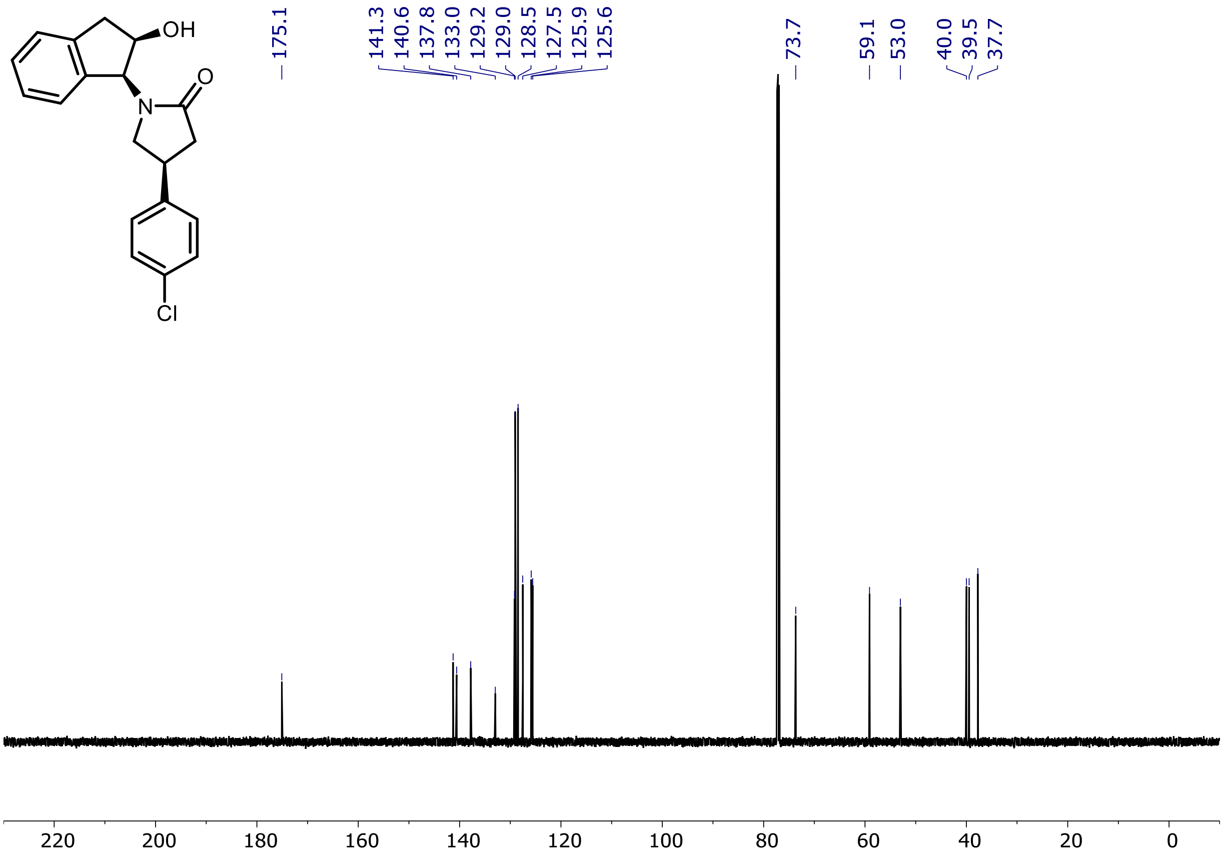

**[*epi*-3f]**, $^{1}$H NMR, CDCl$_3$, 600 MHz

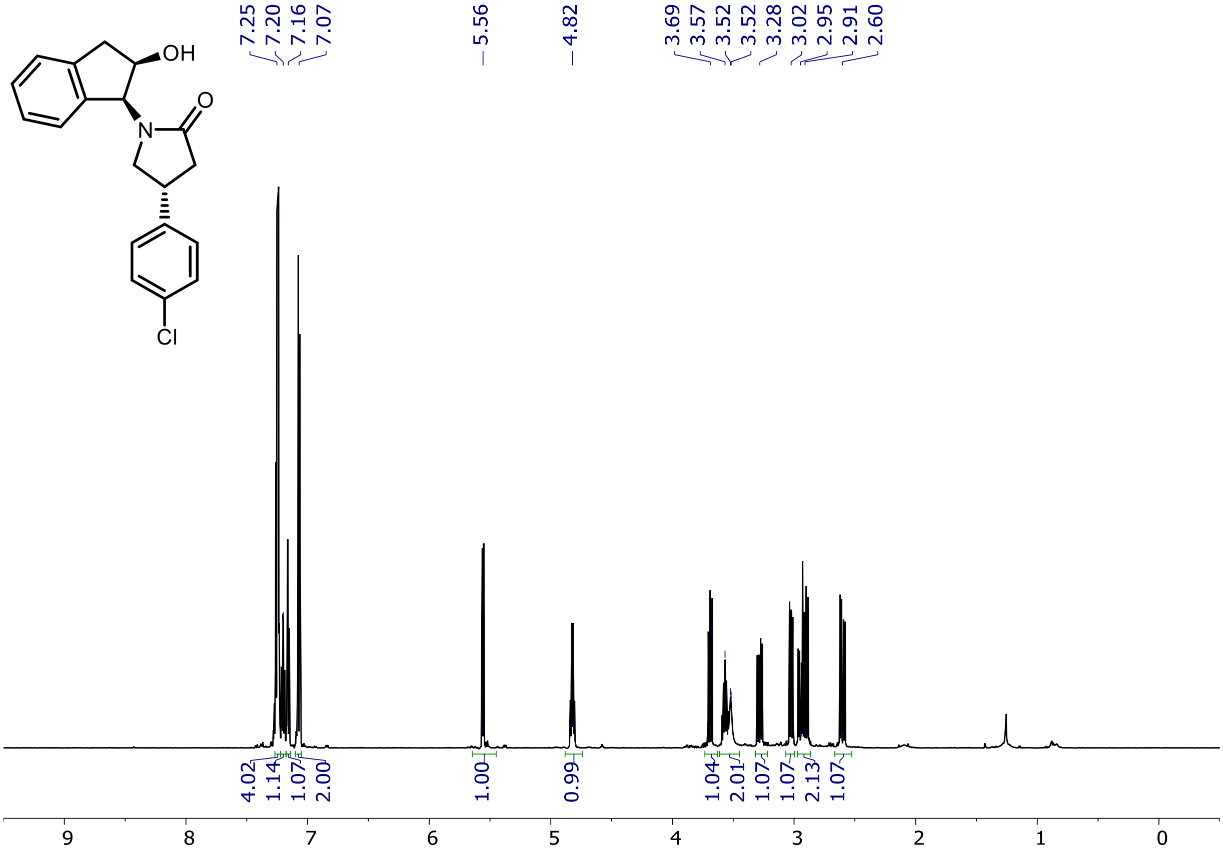


**[*epi*-3f]**, $^{13}$C NMR, CDCl$_3$, 151 MHz

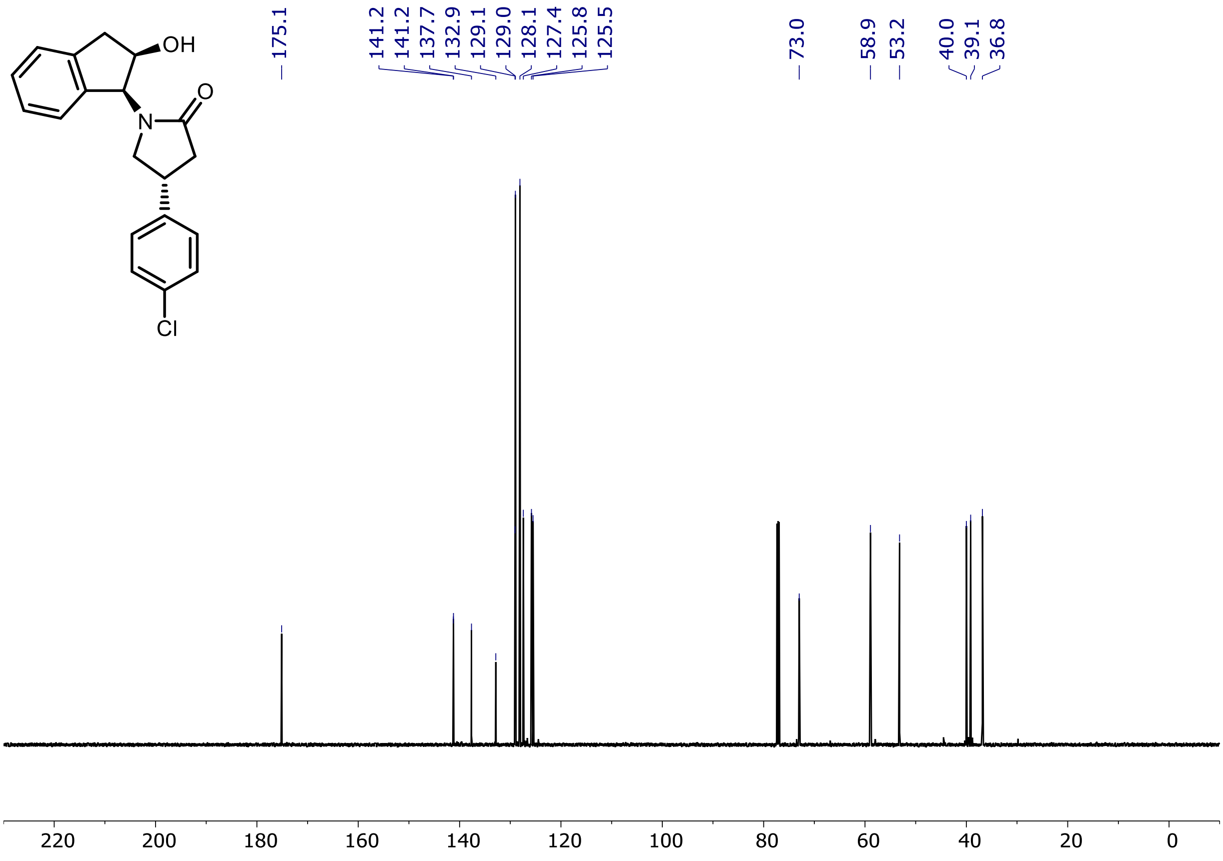

**[3g]**, $^{1}$H NMR, $CDCl_3$, 600 MHz

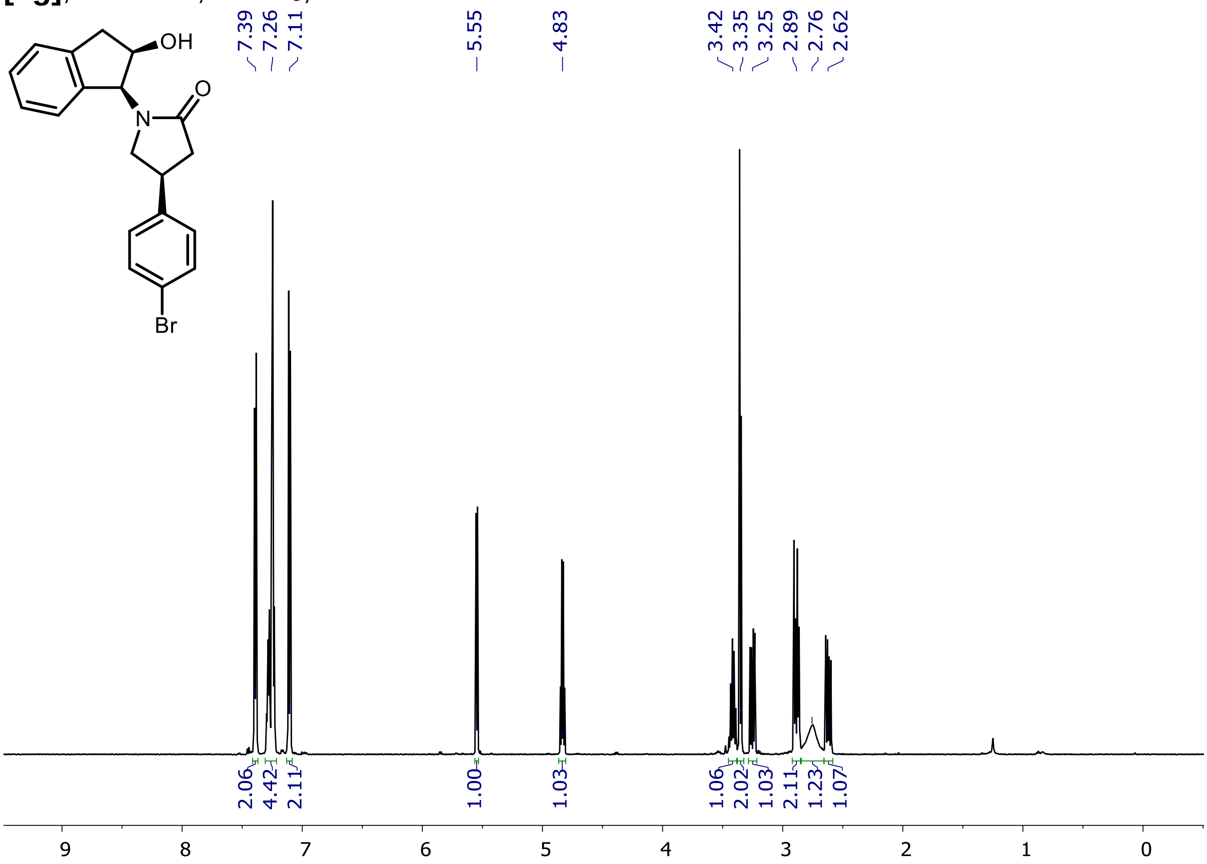


**[3g]**, $^{13}$C NMR, $CDCl_3$, 151 MHz

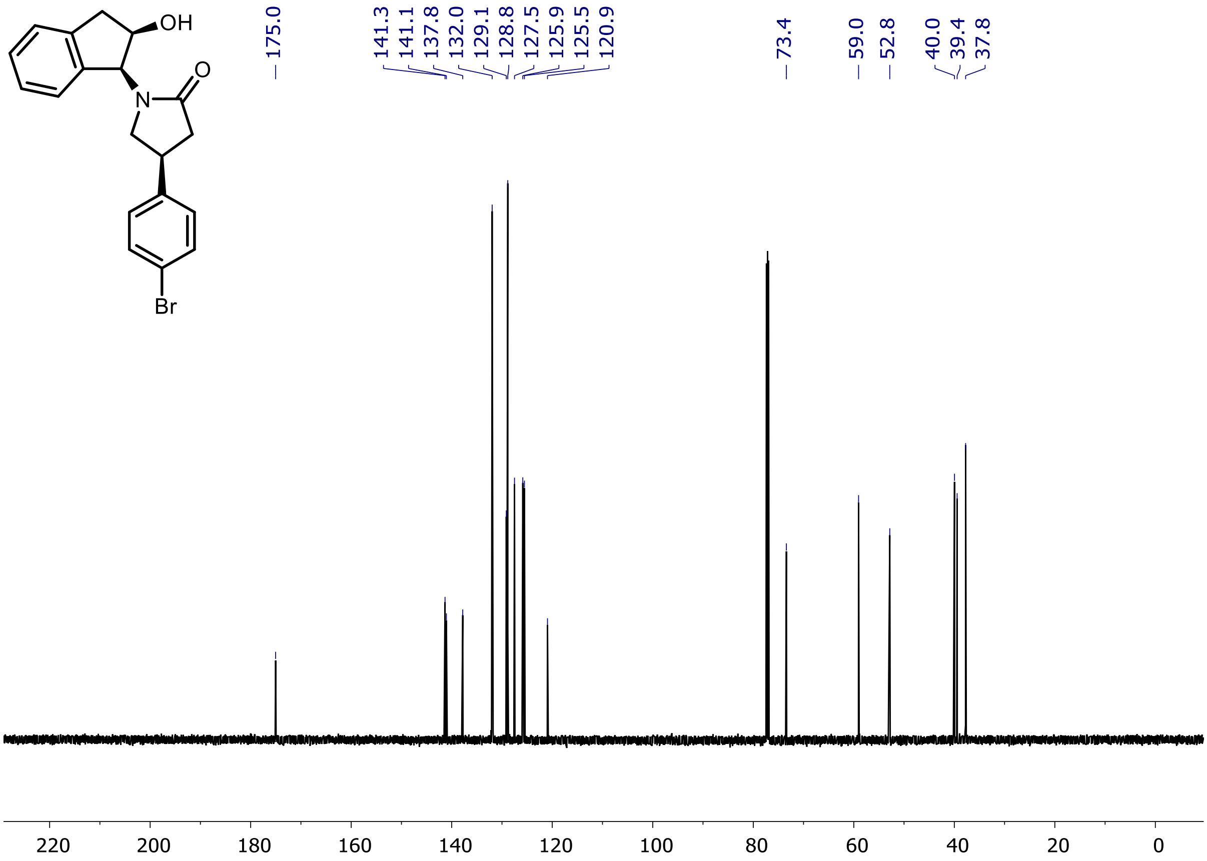

**[*epi*-3g]**, $^{1}$H NMR, CDCl$_3$, 600 MHz

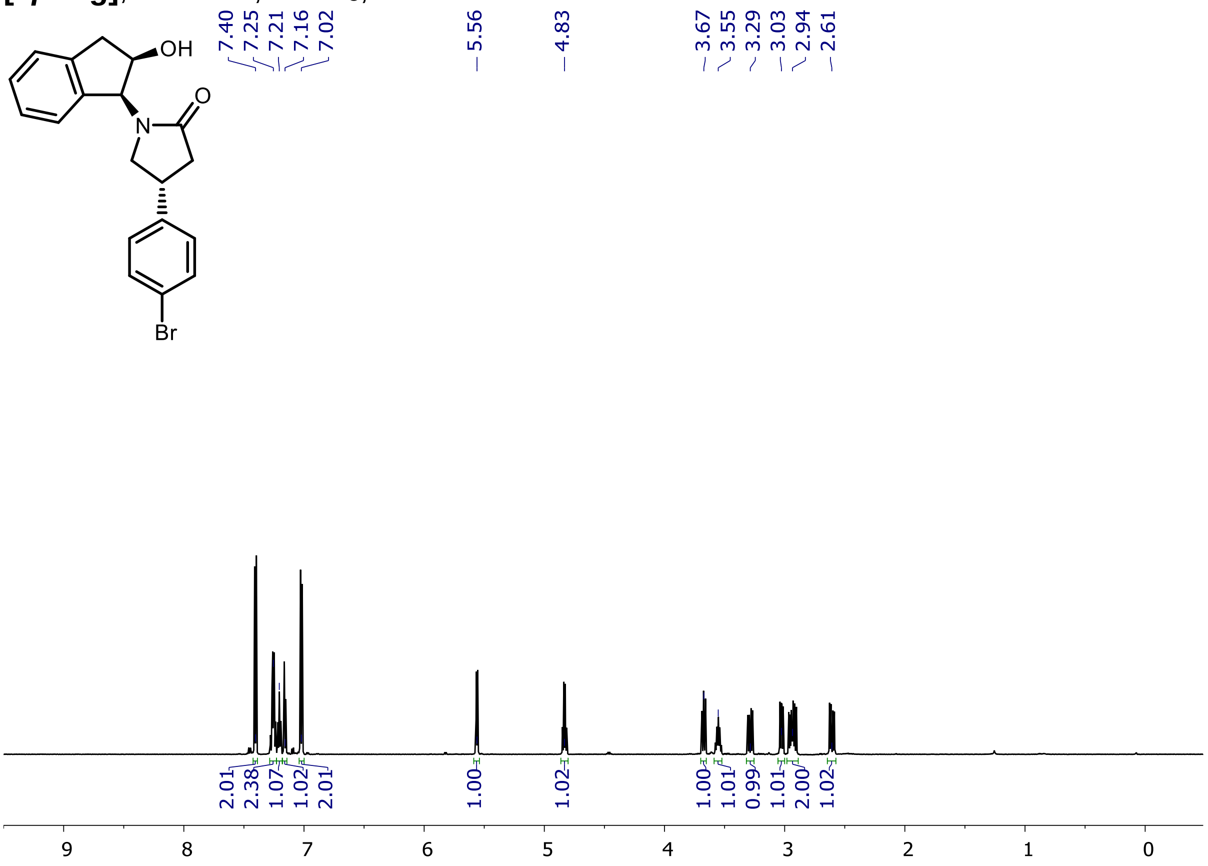


**[*epi*-3g]**, $^{13}$C NMR, CDCl$_3$, 151 MHz

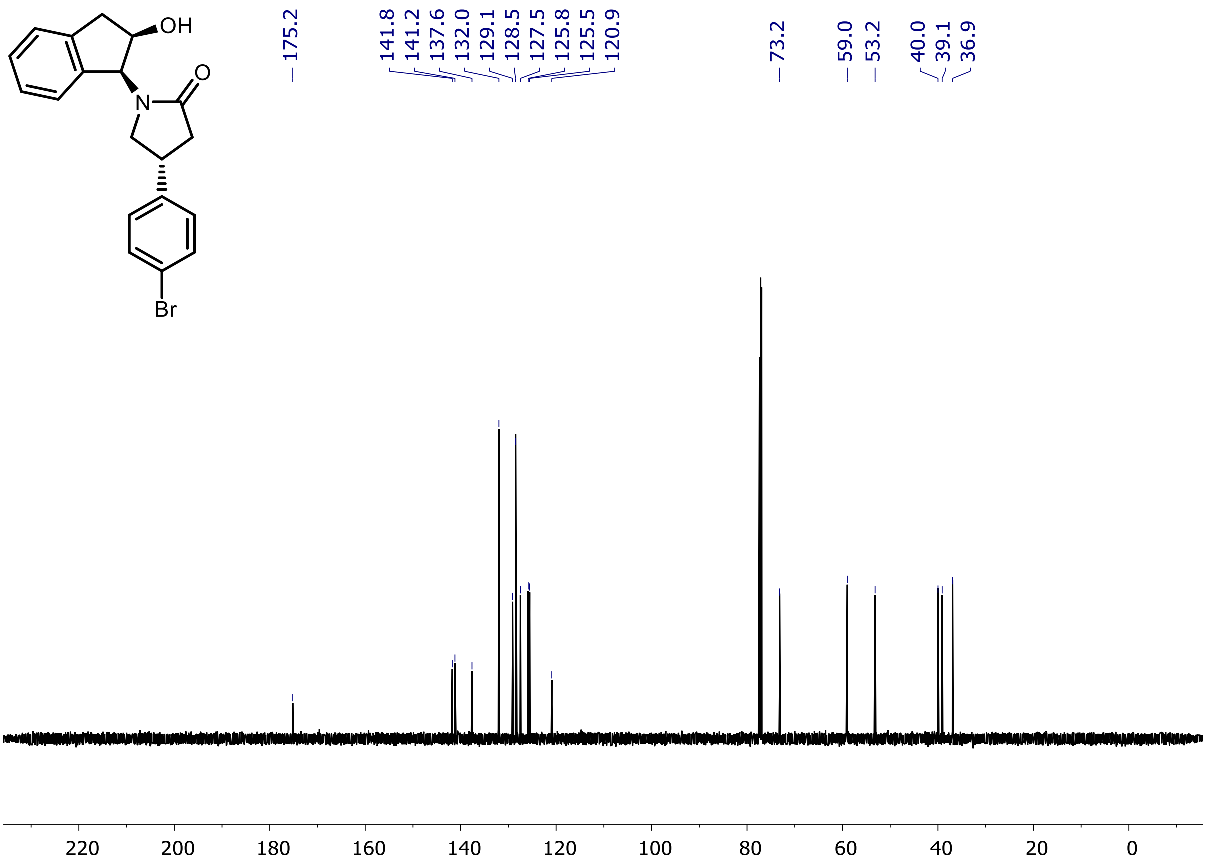

**[3h]**, $^{1}H$ NMR, $CDCl_3$, 600 MHz

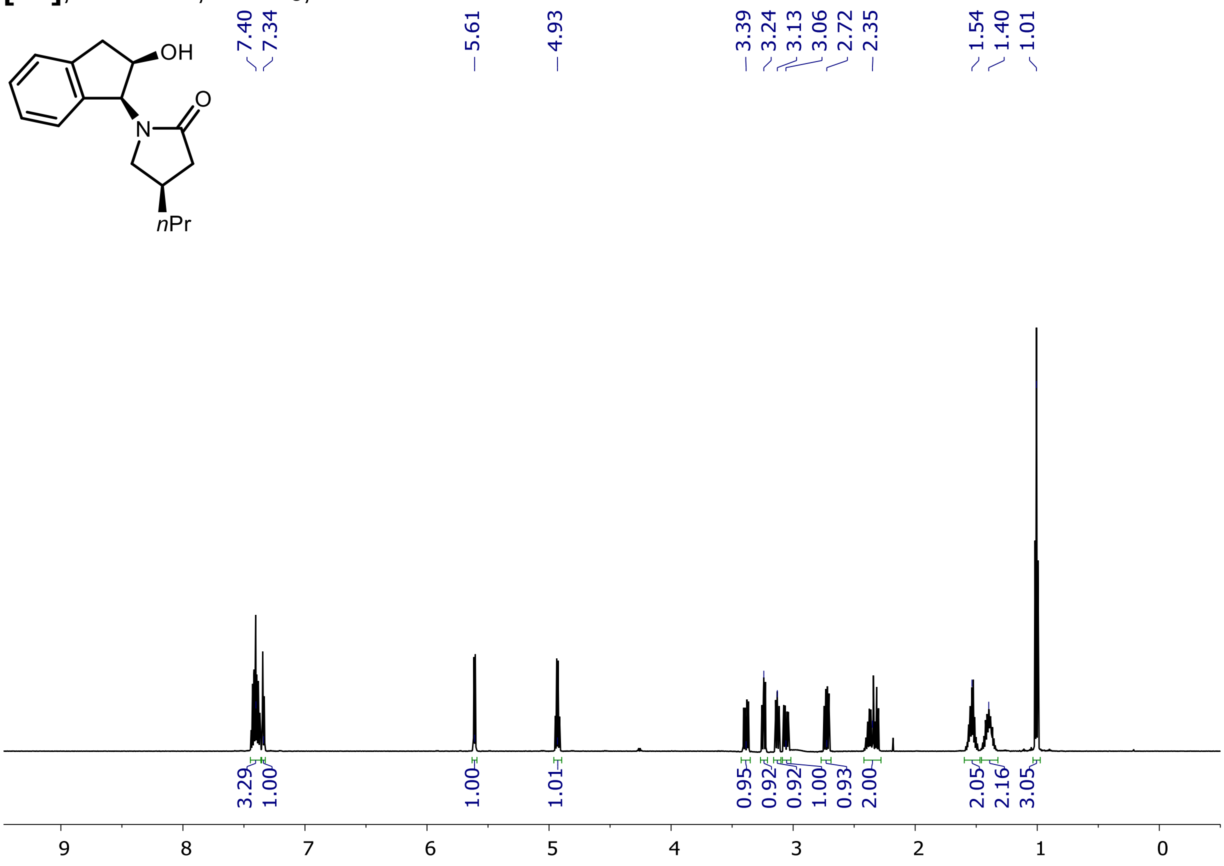


**[3h]**, $^{13}C$ NMR, $CDCl_3$, 151 MHz

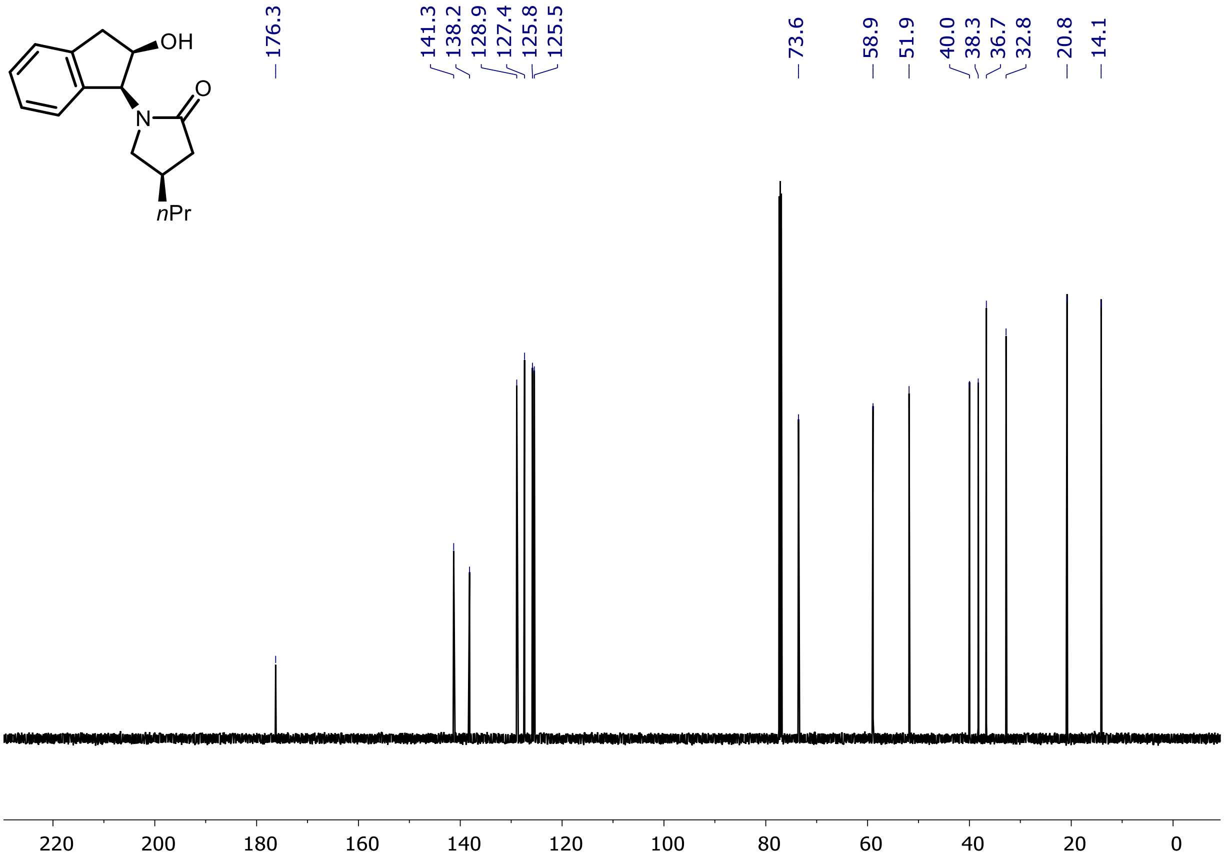

**[*epi*-3h]**, $^{1}H$ NMR, $CDCl_3$, 600 MHz

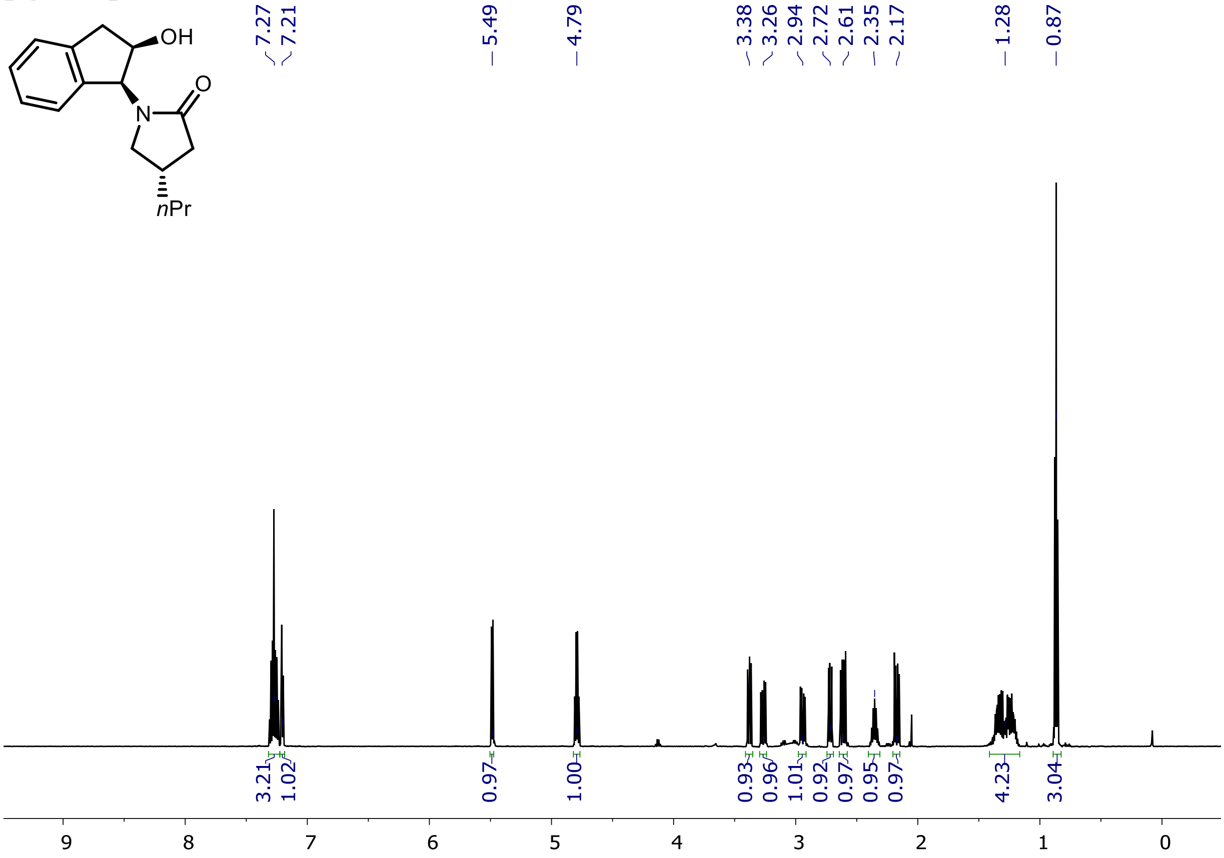


**[*epi*-3h]**, $^{13}C$ NMR, $CDCl_3$, 151 MHz

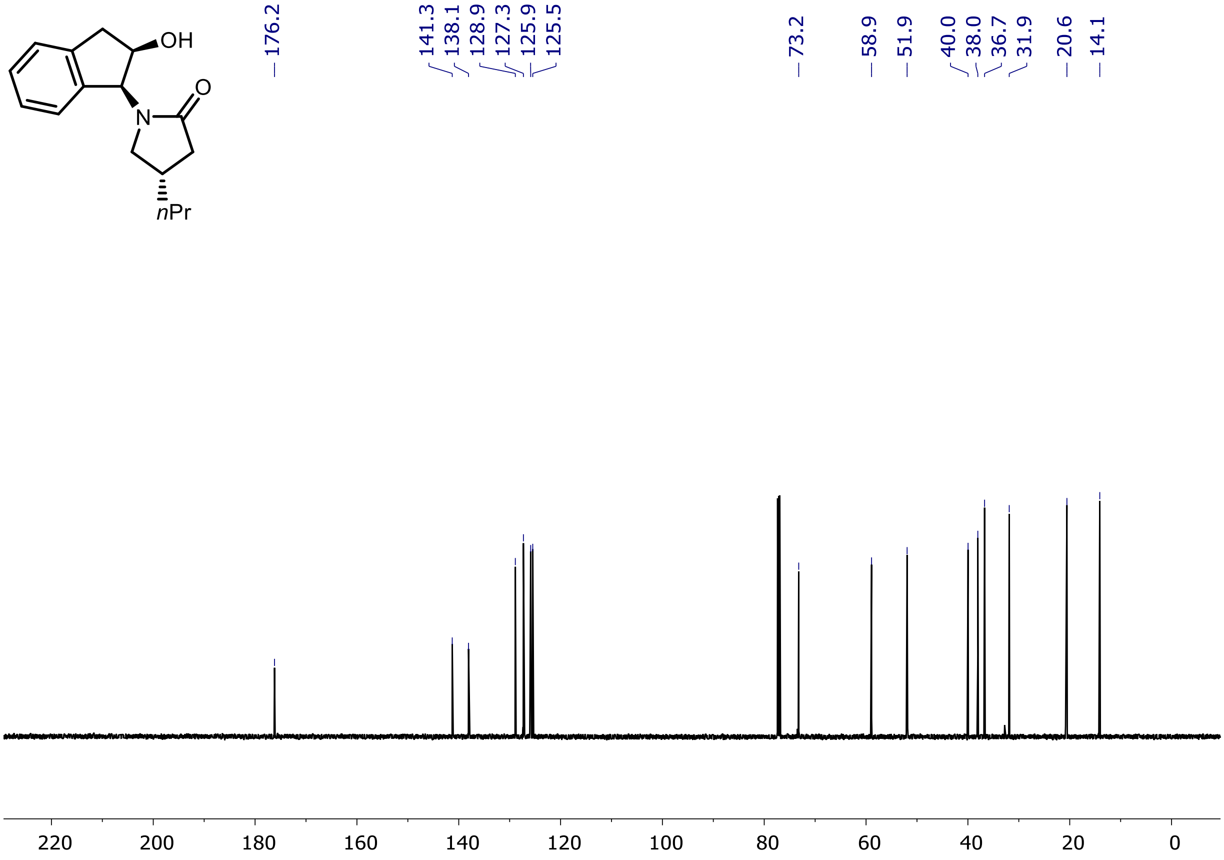

**[3i]**, $^{1}$H NMR, $CDCl_3$, 500 MHz

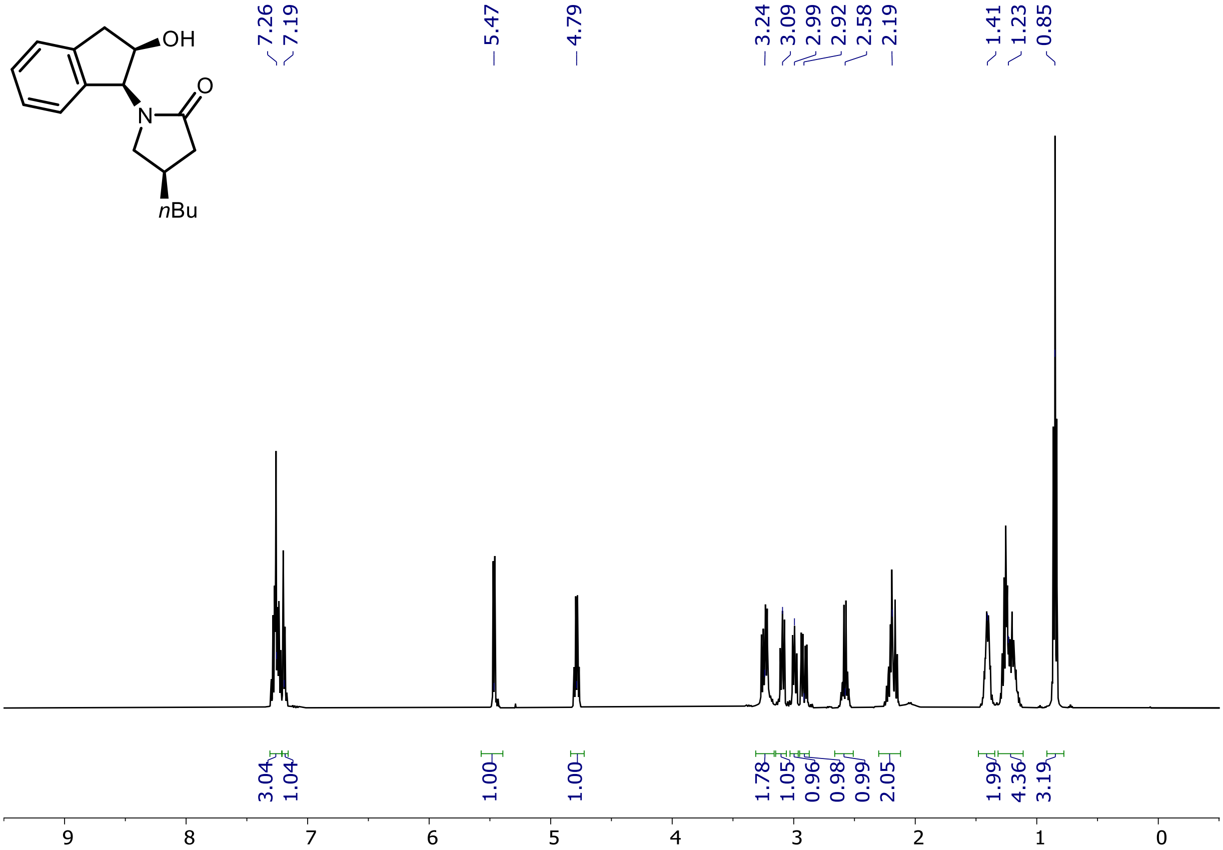


**[3i]**, $^{13}$C NMR, $CDCl_3$, 126 MHz

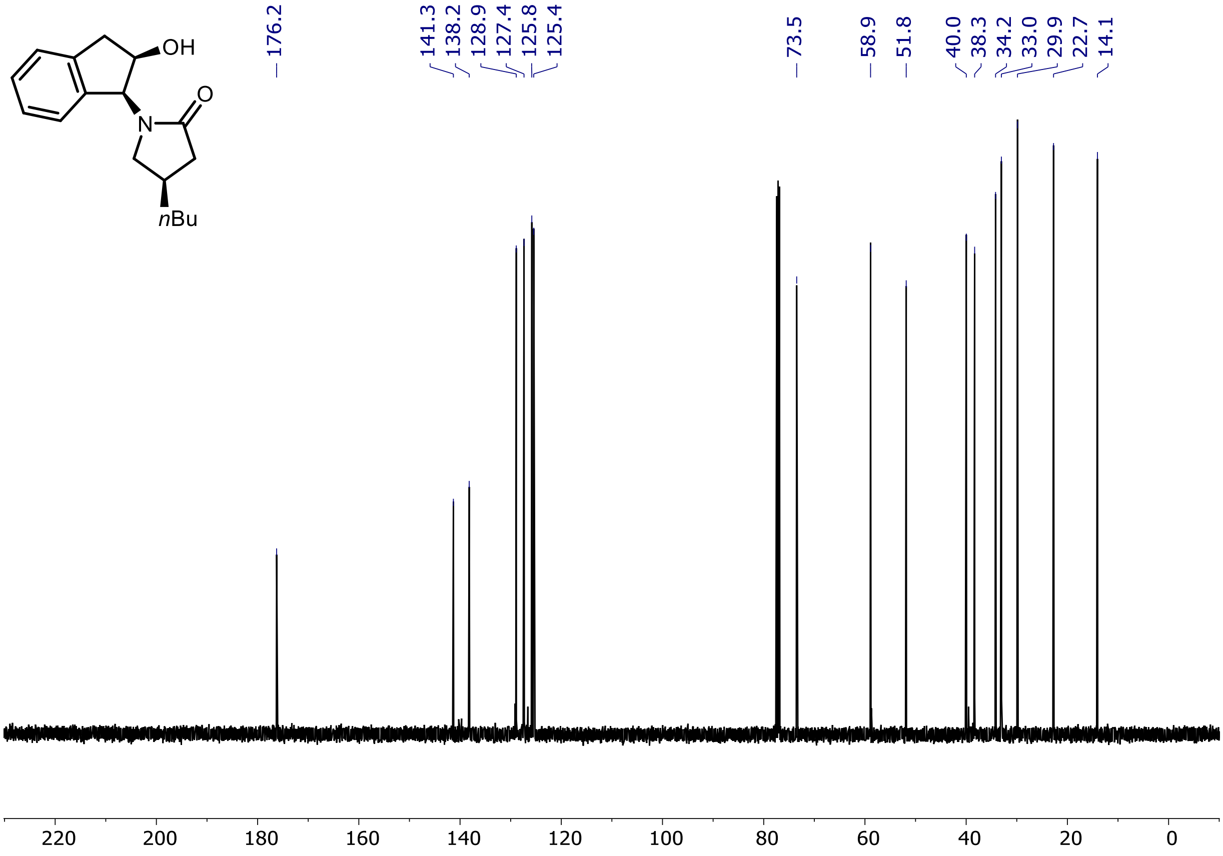

**[*epi*-3i]**, $^{1}$H NMR, CDCl$_3$, 600 MHz

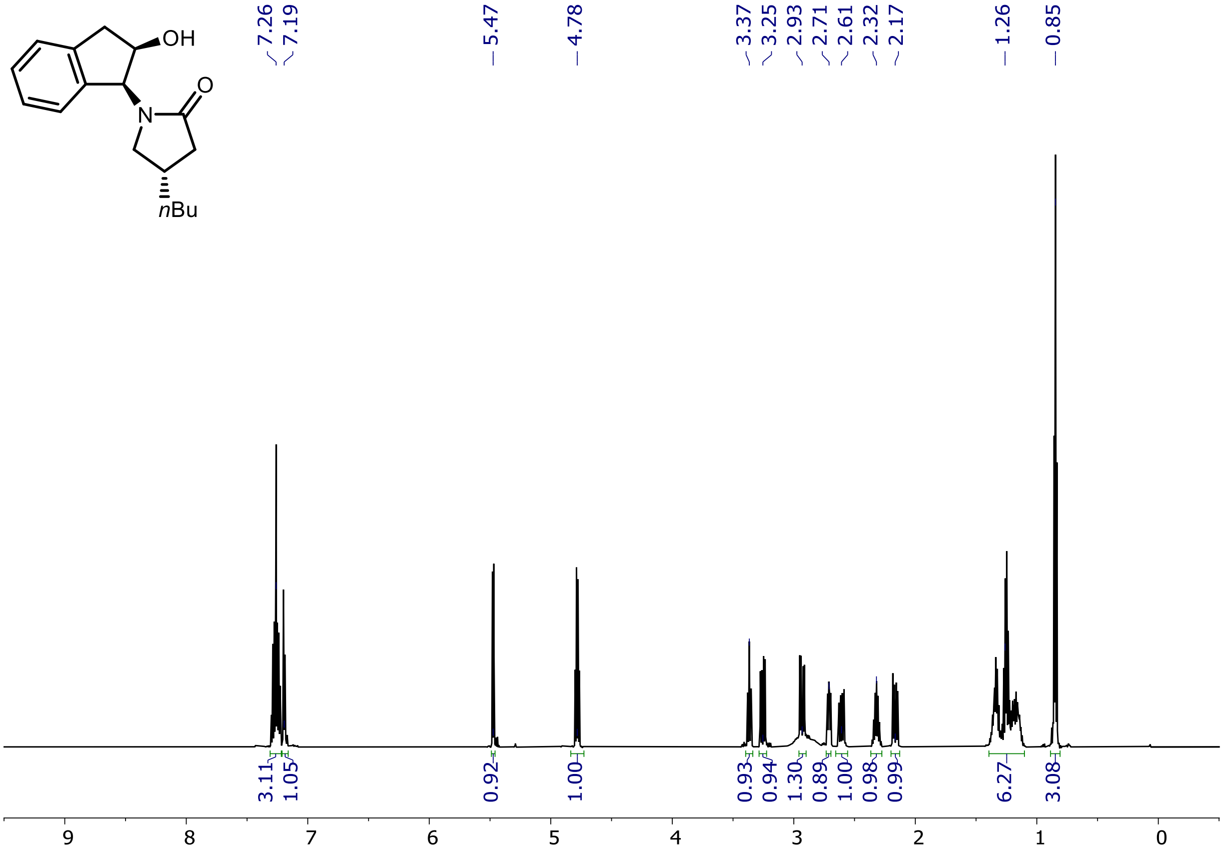


**[*epi*-3i]**, $^{13}$C NMR, CDCl$_3$, 151 MHz

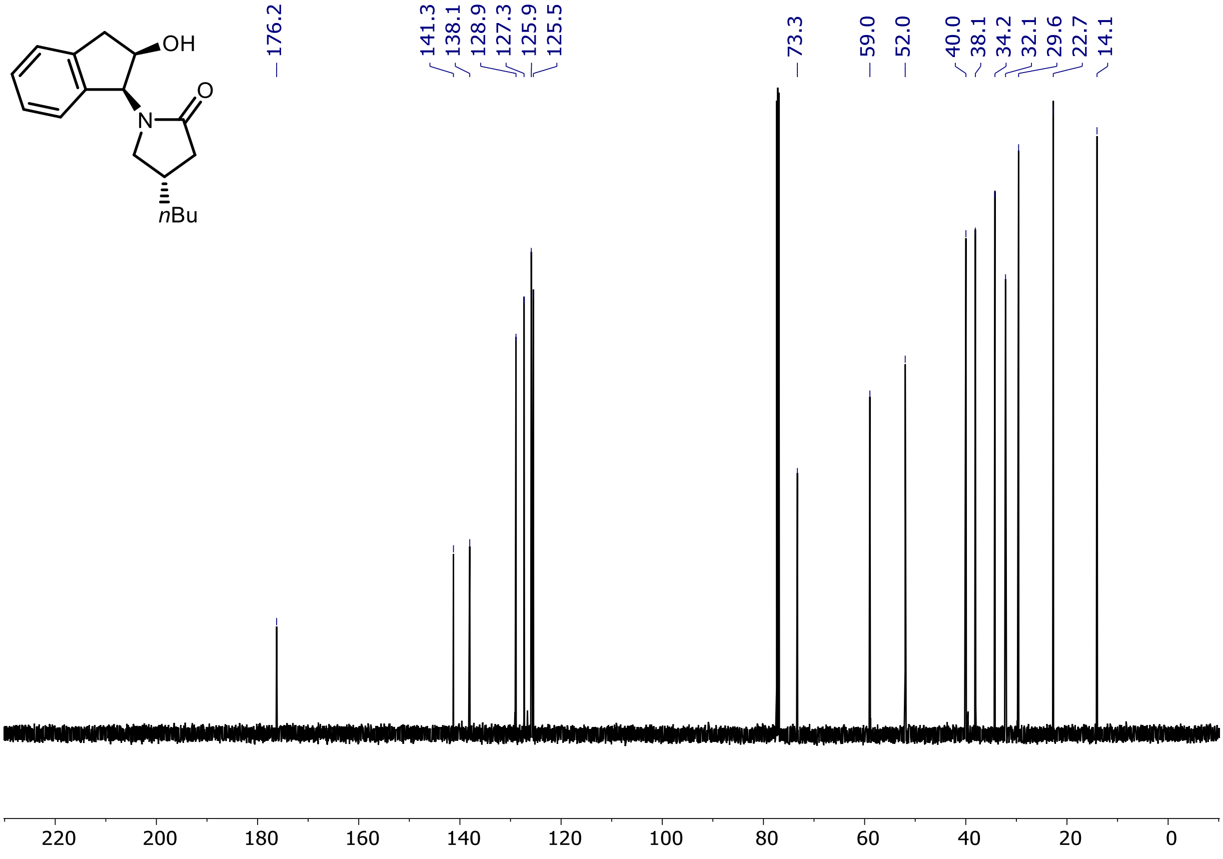

**[3j]**, $^1$H NMR, $CDCl_3$, 500 MHz

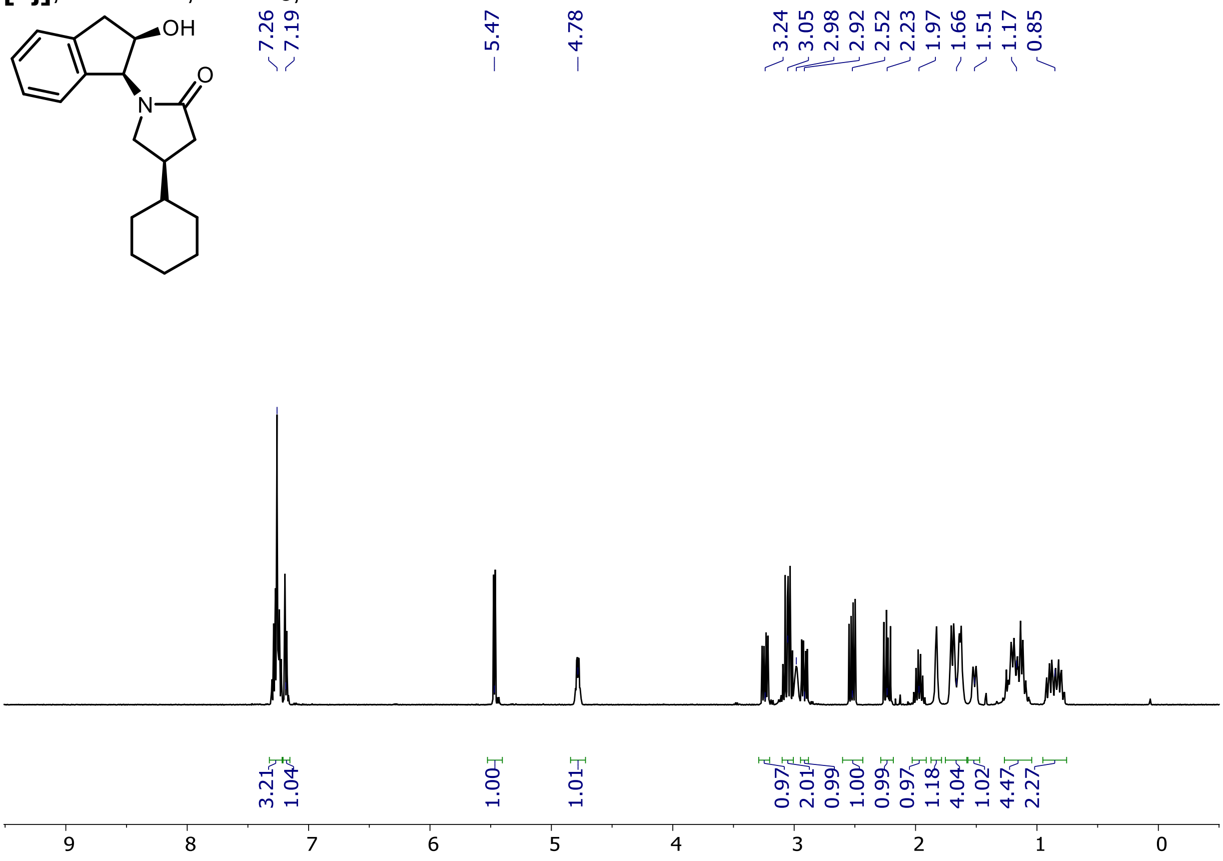


**[3j]**, $^{13}$C NMR, $CDCl_3$, 126 MHz

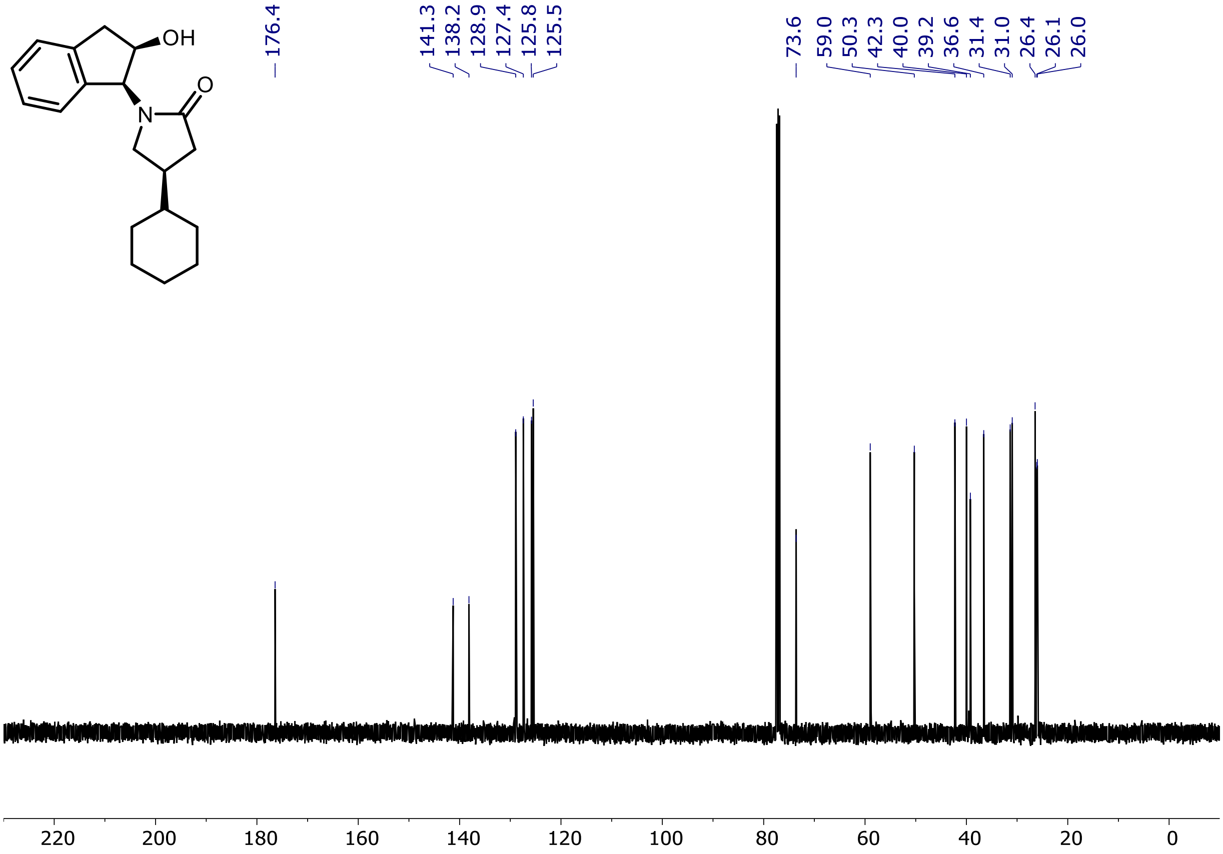

**[*epi*-3j]**, $^{1}$H NMR, $CDCl_3$, 500 MHz

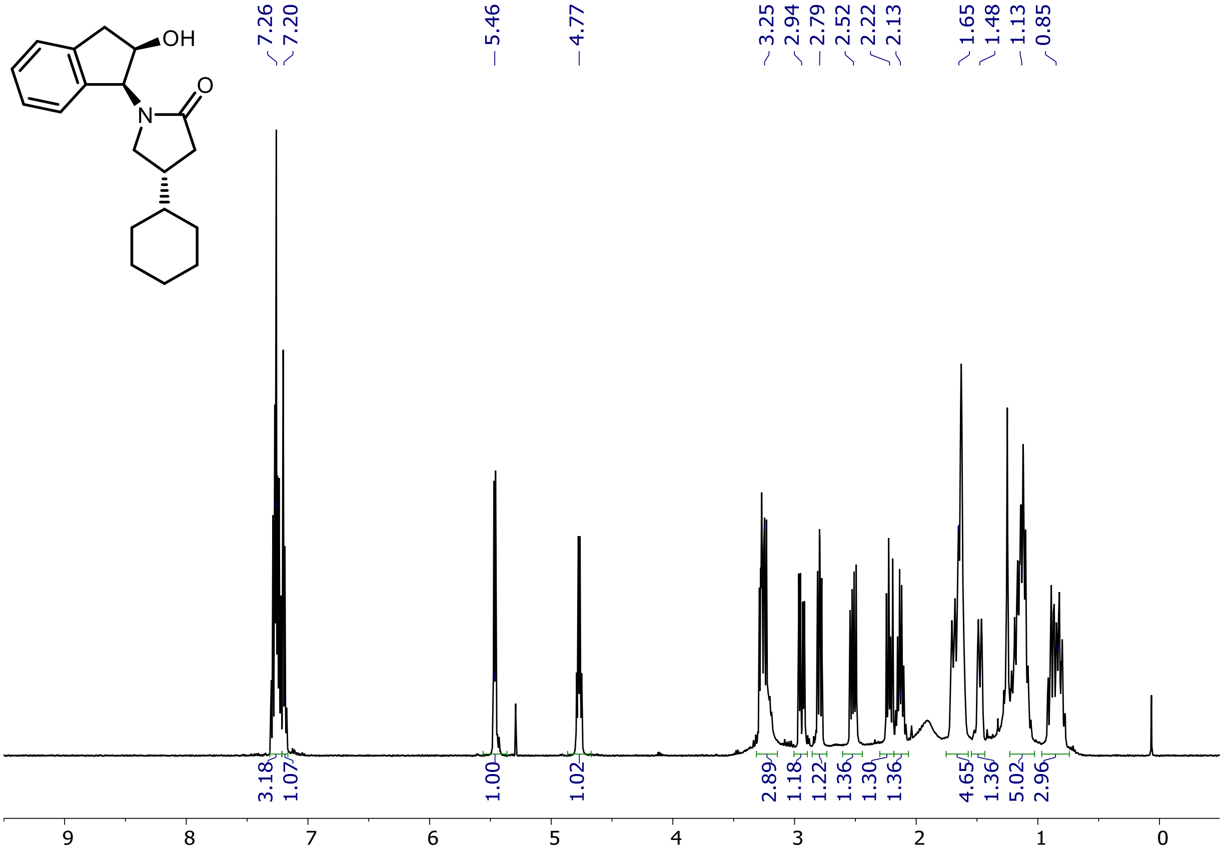


**[*epi*-3j]**, $^{13}$C NMR, $CDCl_3$, 126 MHz

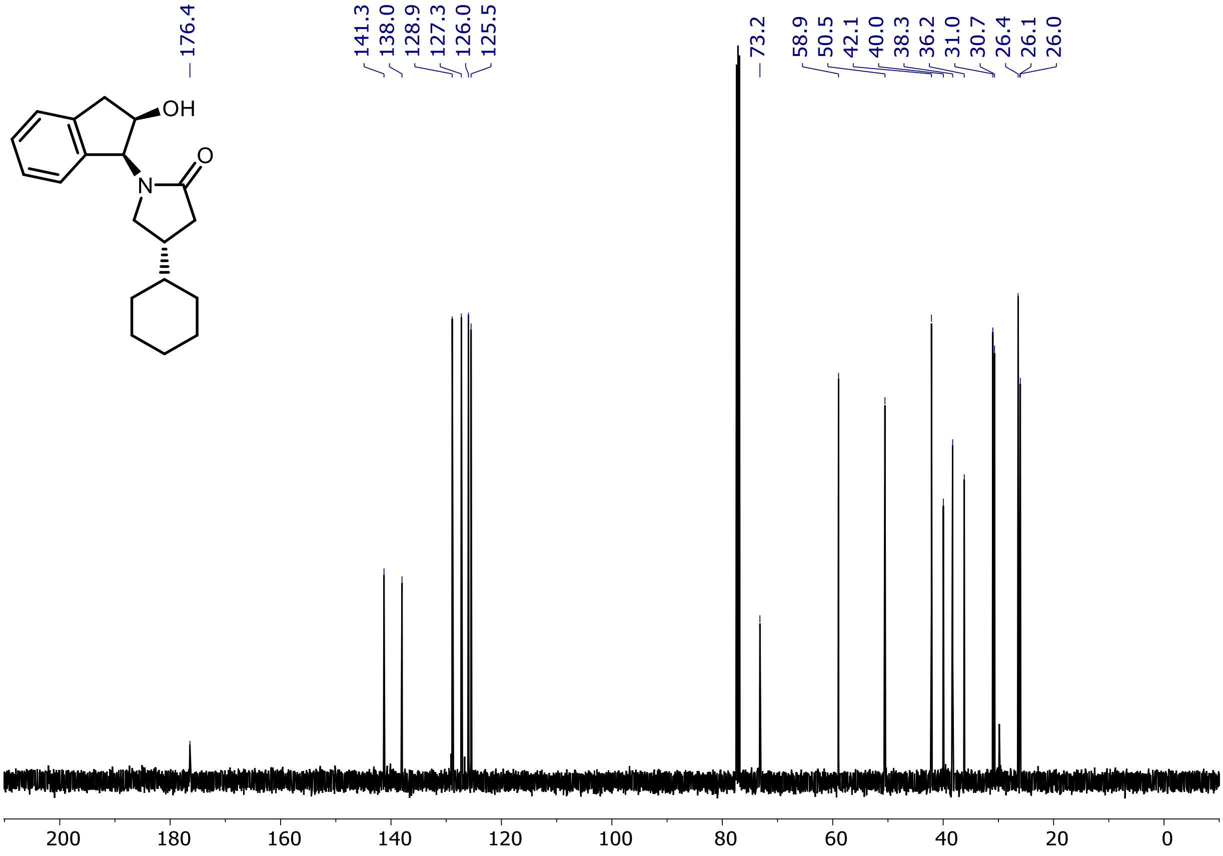

**[3k]**, $^{1}$H NMR, $CDCl_3$, 500 MHz

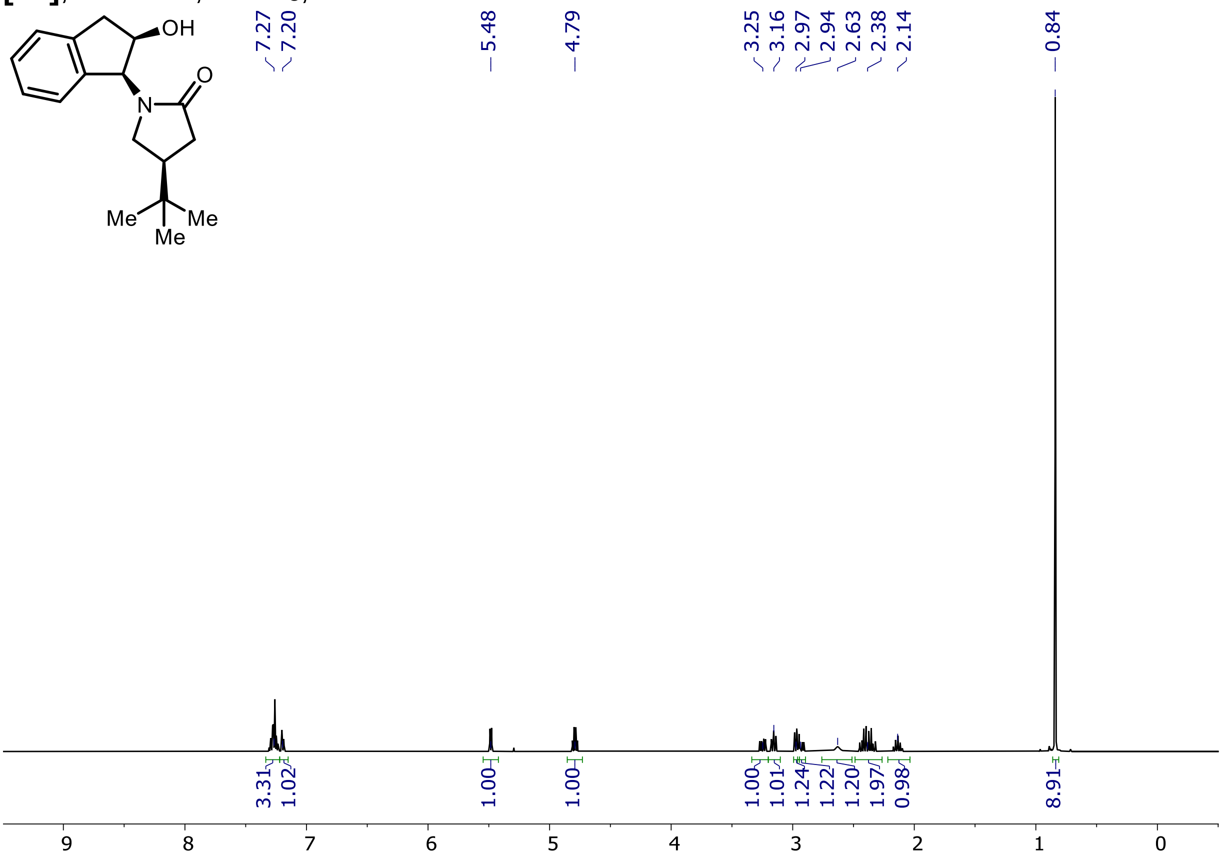


**[3k]**, $^{13}$C NMR, $CDCl_3$, 126 MHz

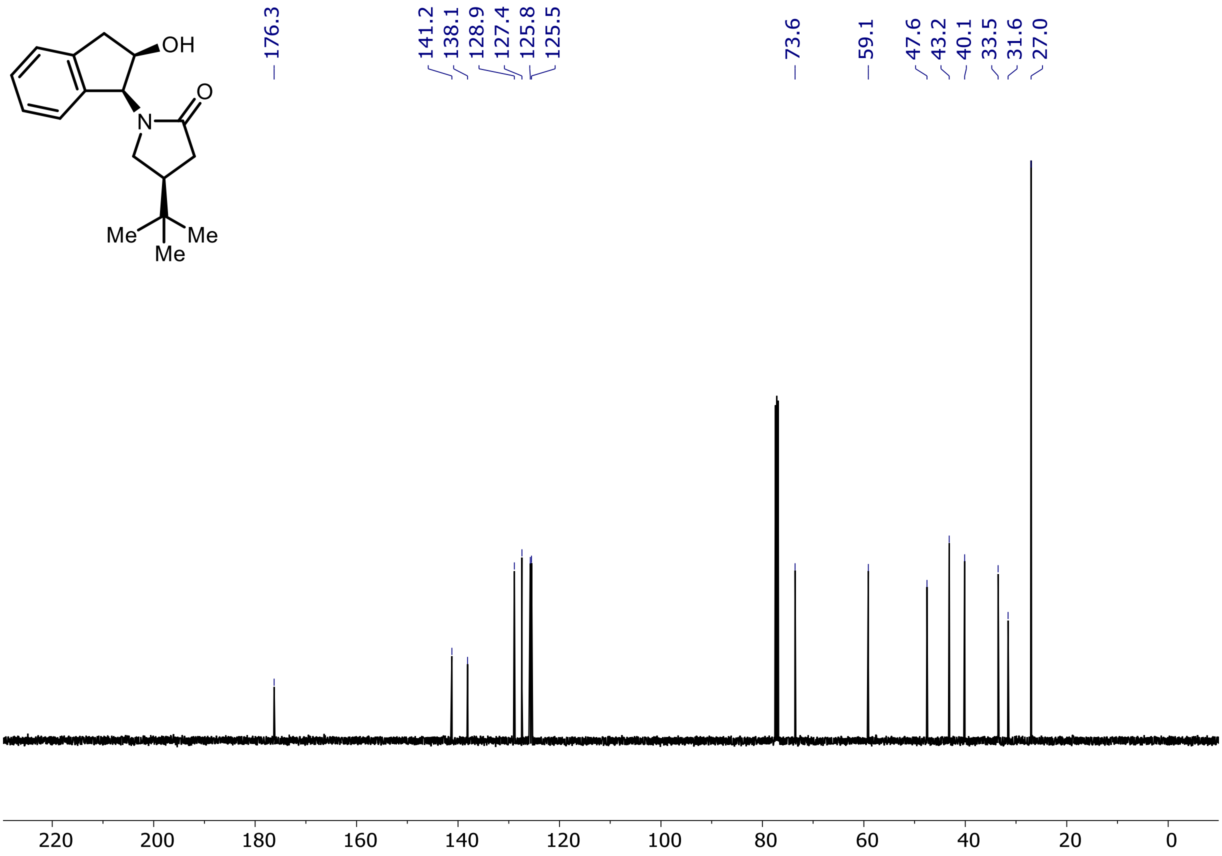

[***epi*-3k**], $^{1}$H NMR, $CDCl_3$, 500 MHz

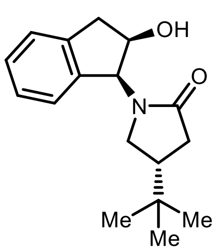

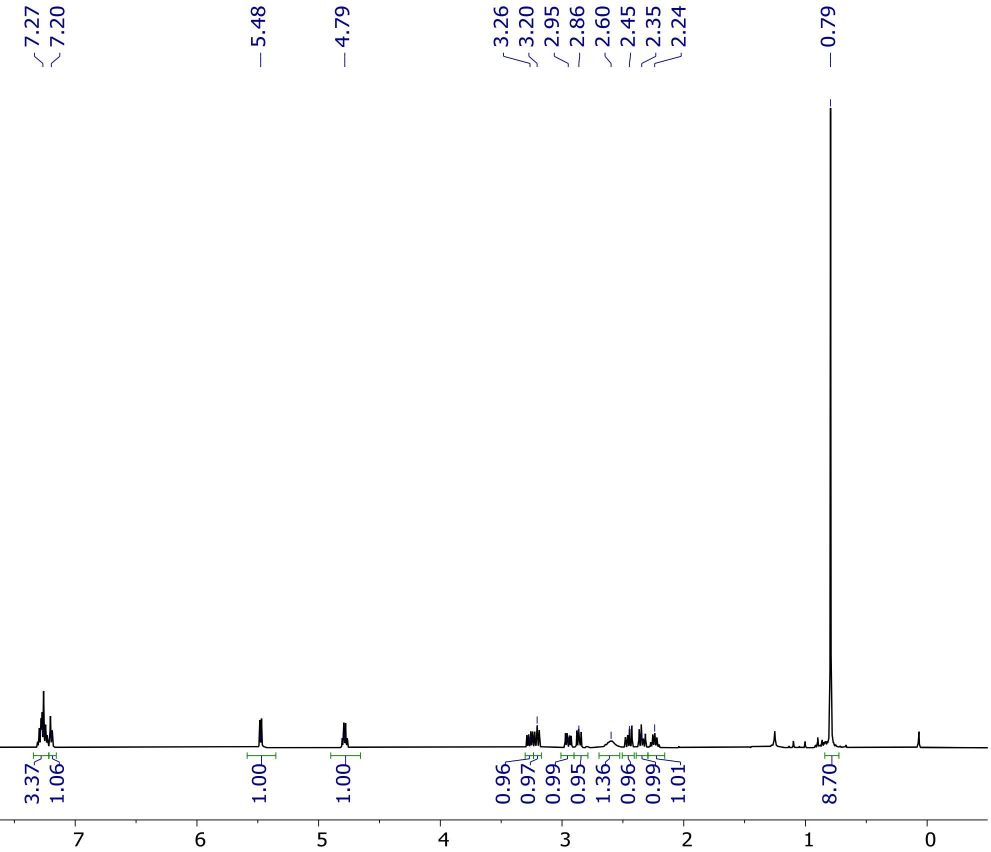


[***epi*-3k**], $^{13}$C NMR, $CDCl_3$, 126 MHz

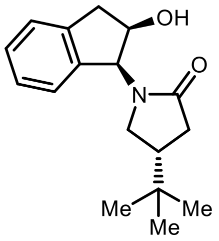

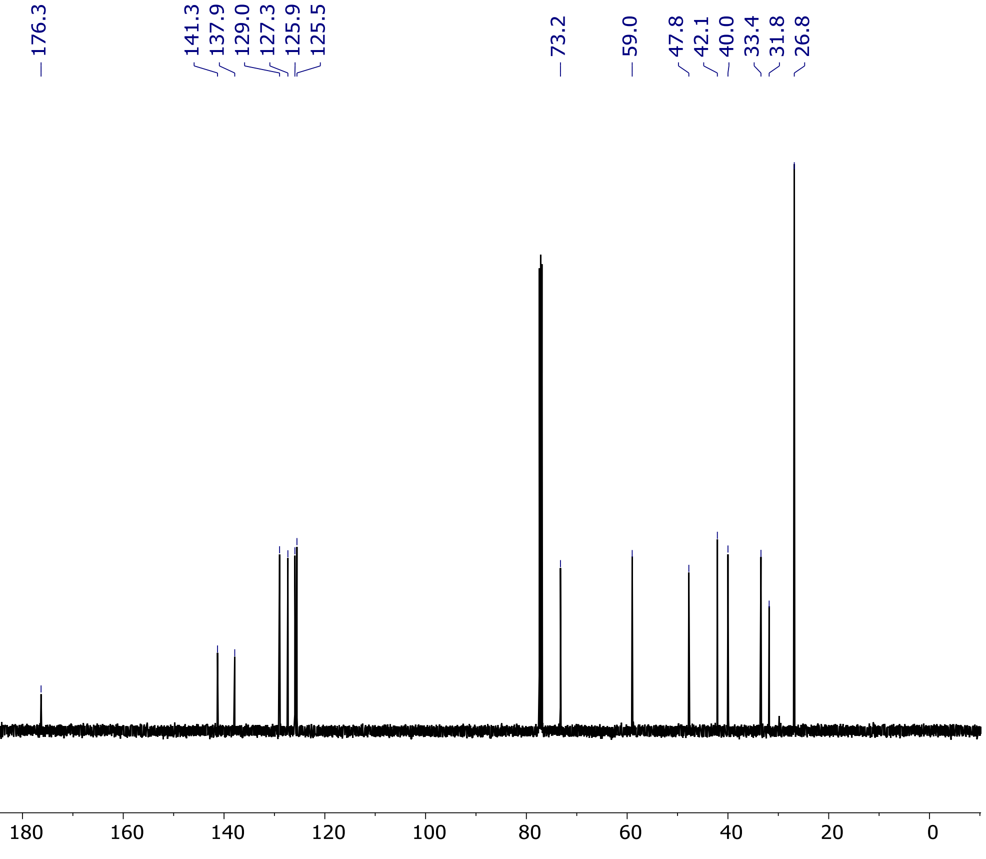

**[3l]**, $^1$H NMR, $CDCl_3$, 500 MHz

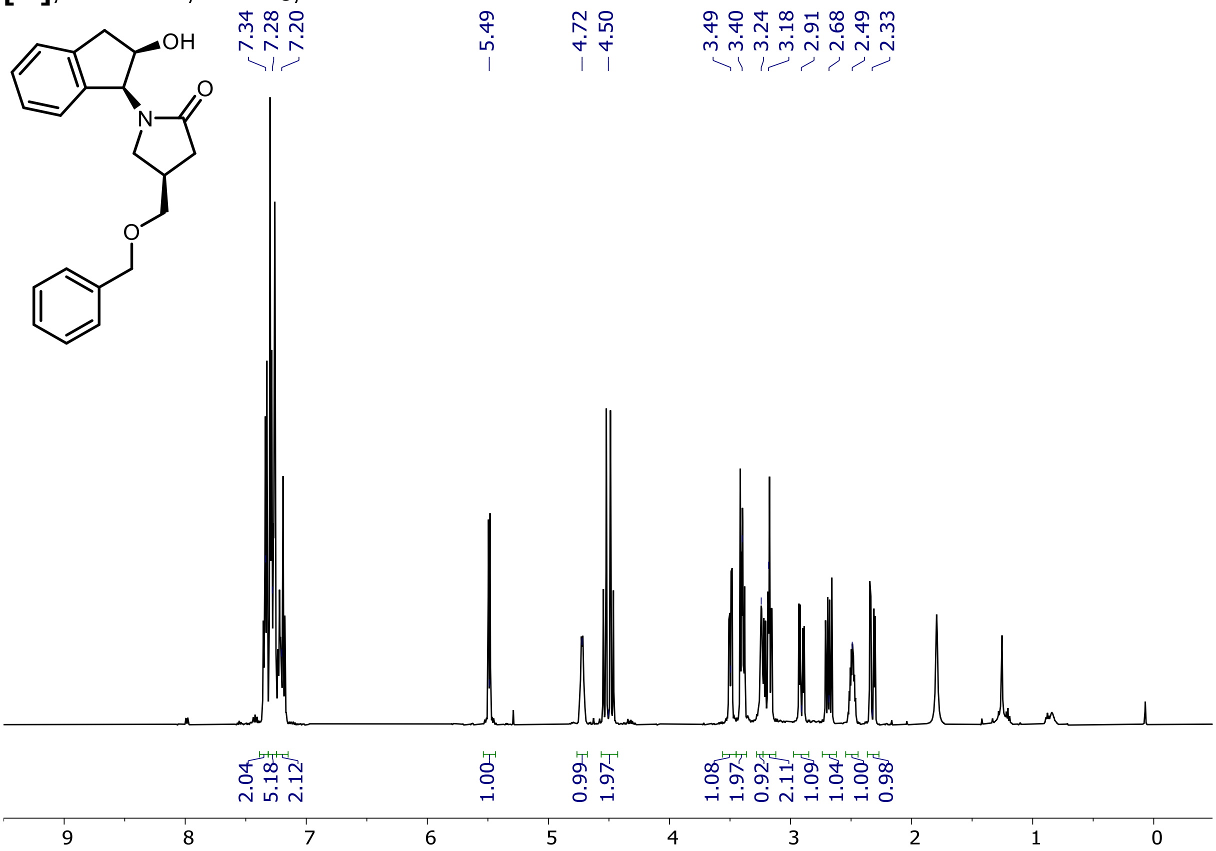


**[3l]**, $^{13}$C NMR, $CDCl_3$, 126 MHz

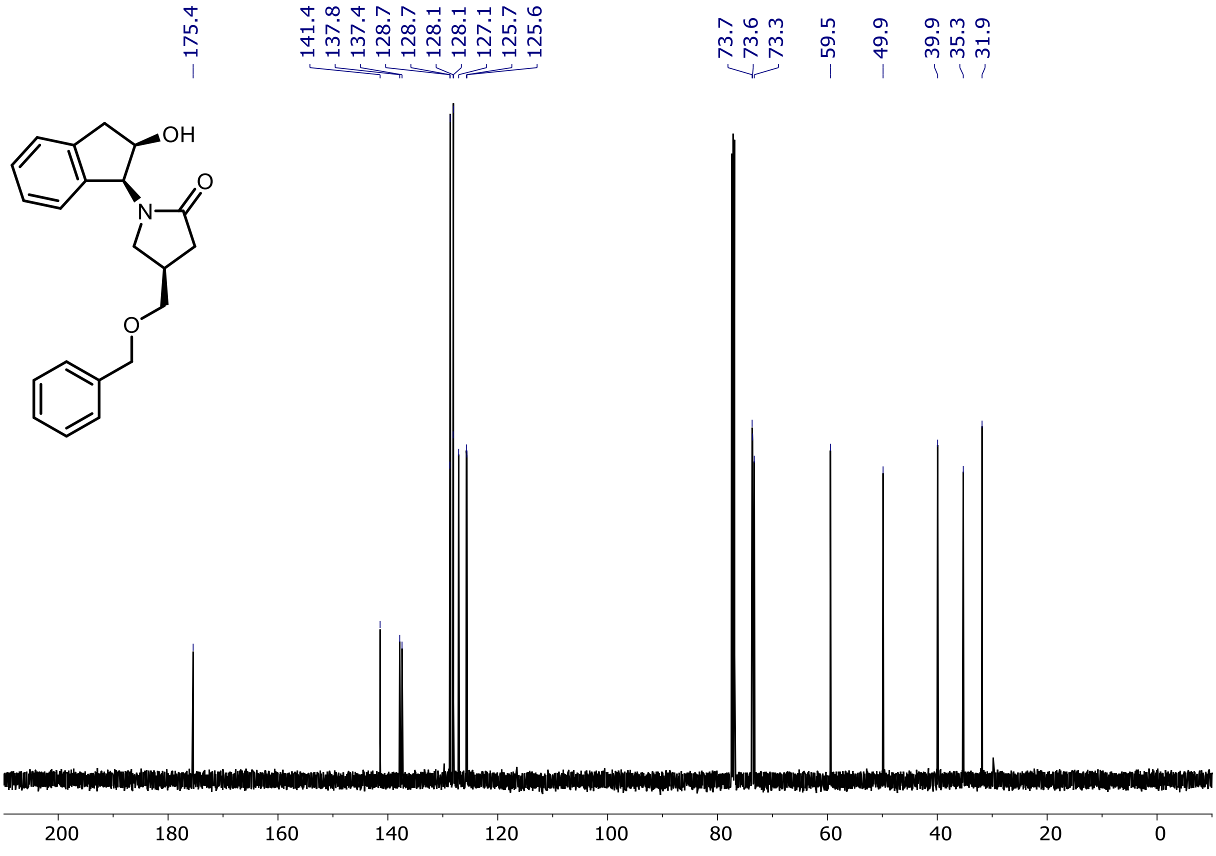

**[*epi*-3l]**, $^{1}$H NMR, $CDCl_3$, 500 MHz

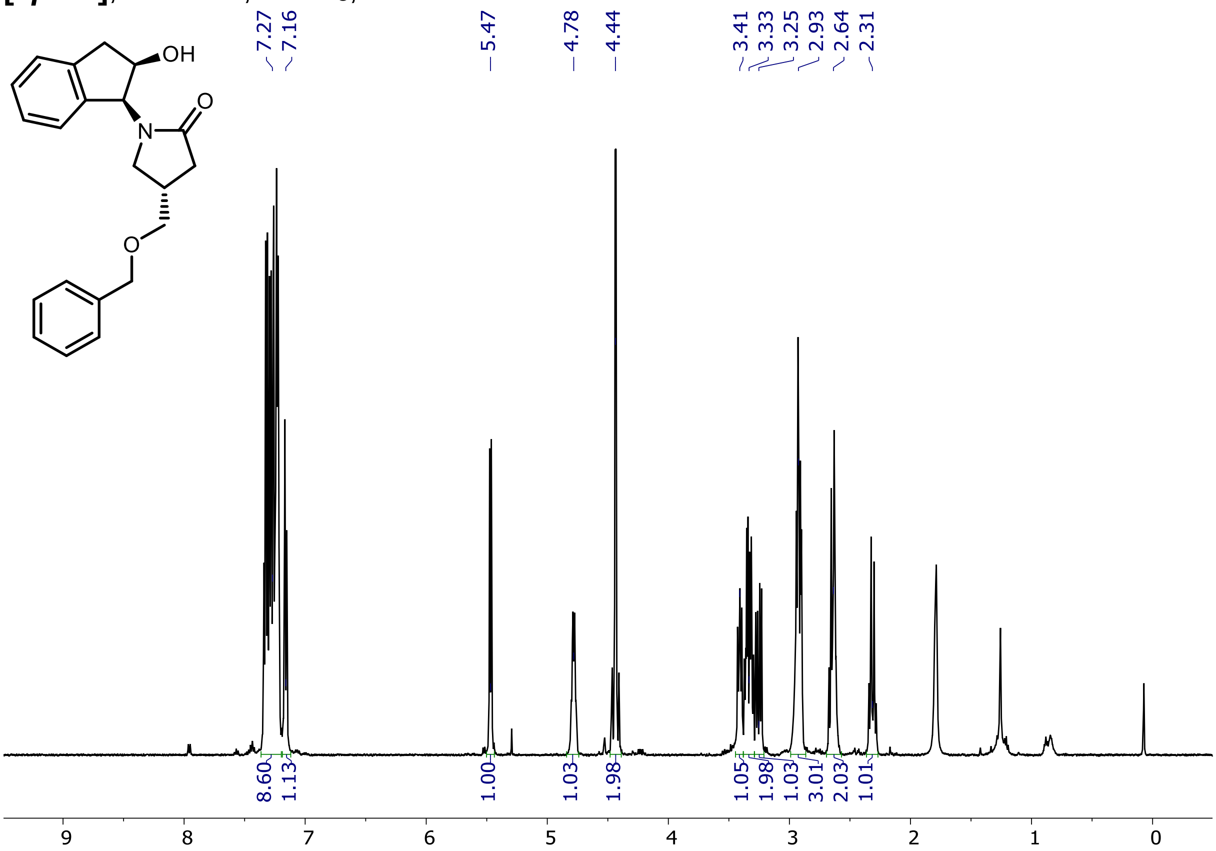


**[*epi*-3l]**, $^{13}$C NMR, $CDCl_3$, 126 MHz

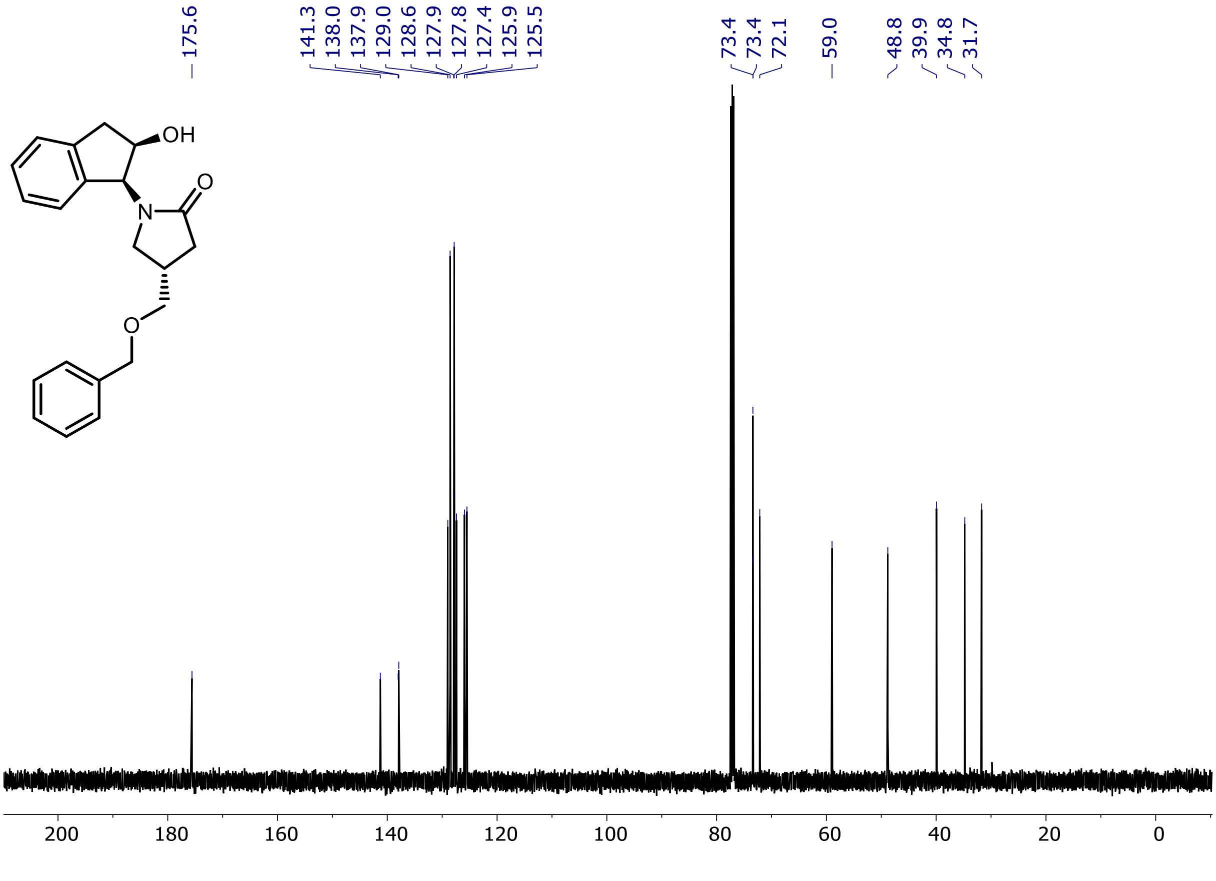

**[3m]**, $^{1}$H NMR, $CDCl_3$, 600 MHz

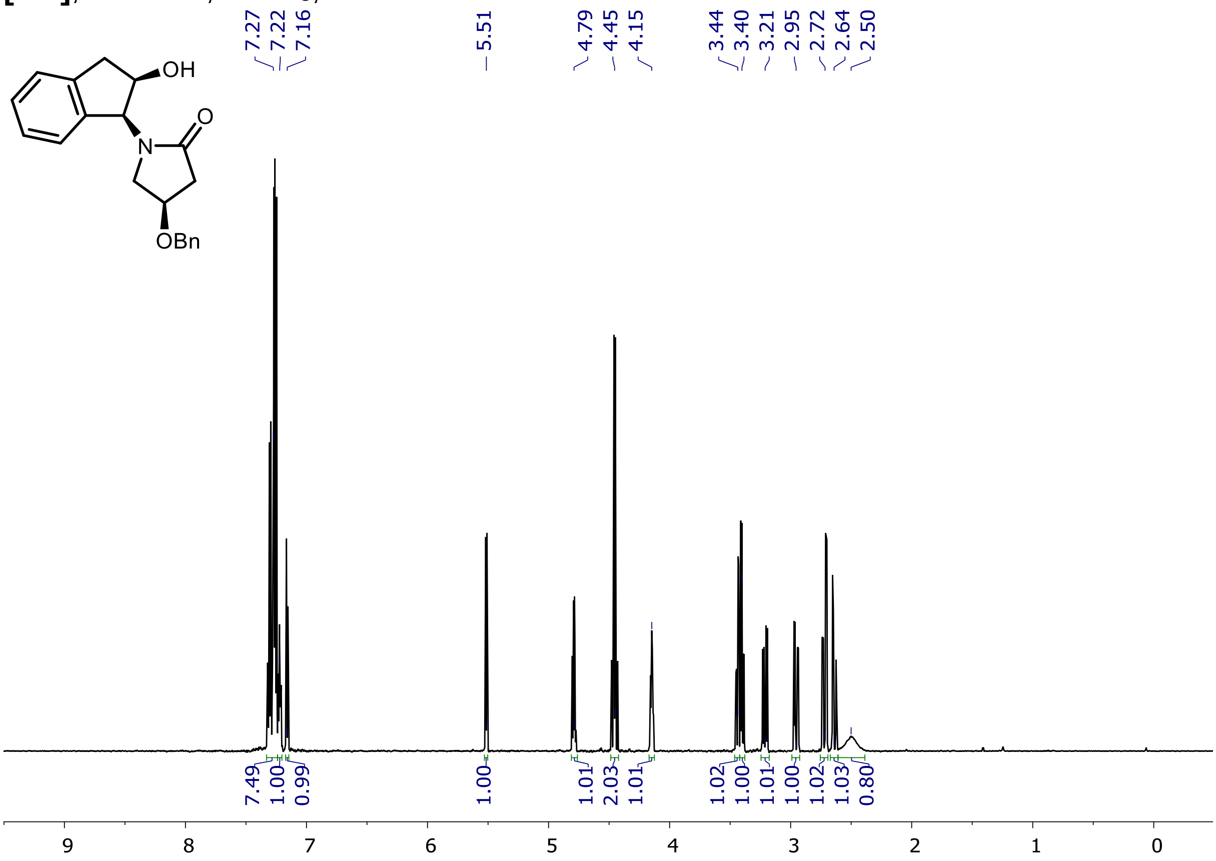


**[3m]**, $^{13}$C NMR, $CDCl_3$, 151 MHz

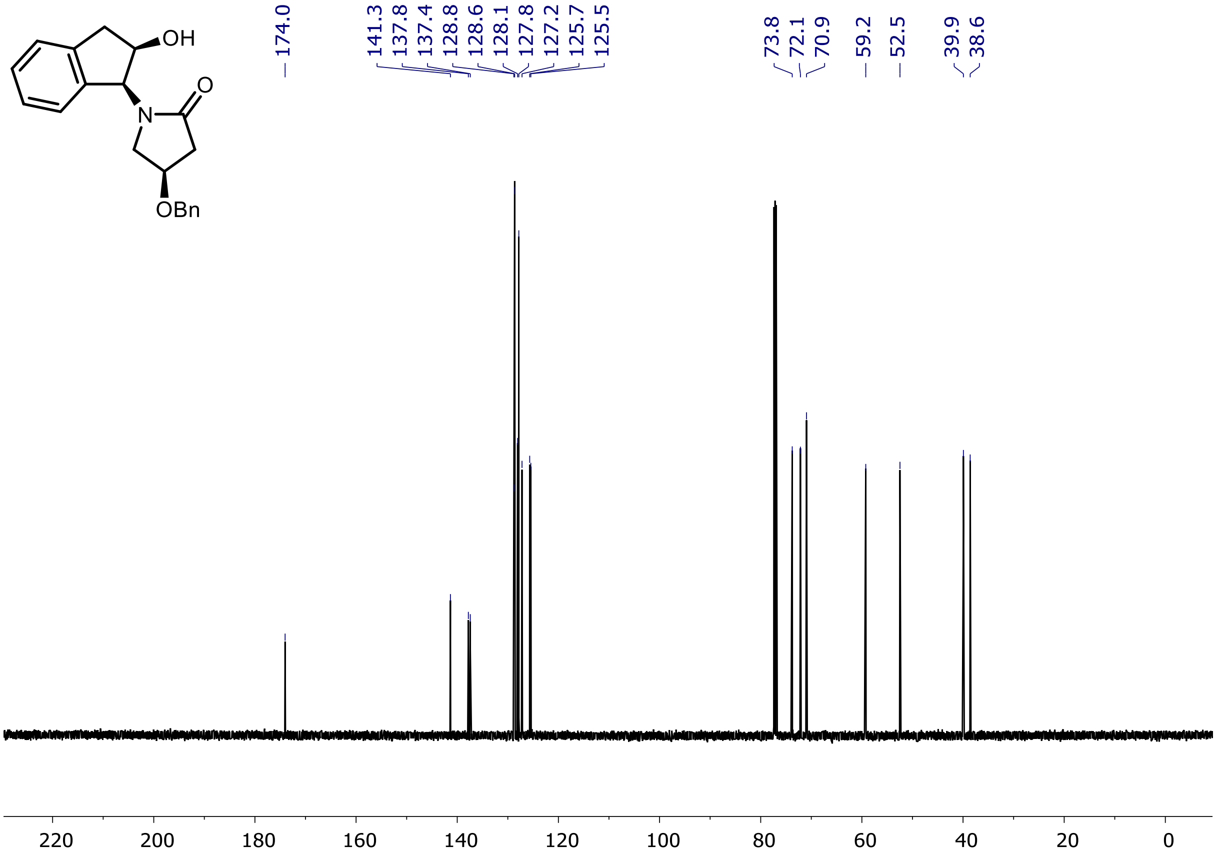

**[3n]**, $^1$H NMR, CDCl$_3$, 500 MHz

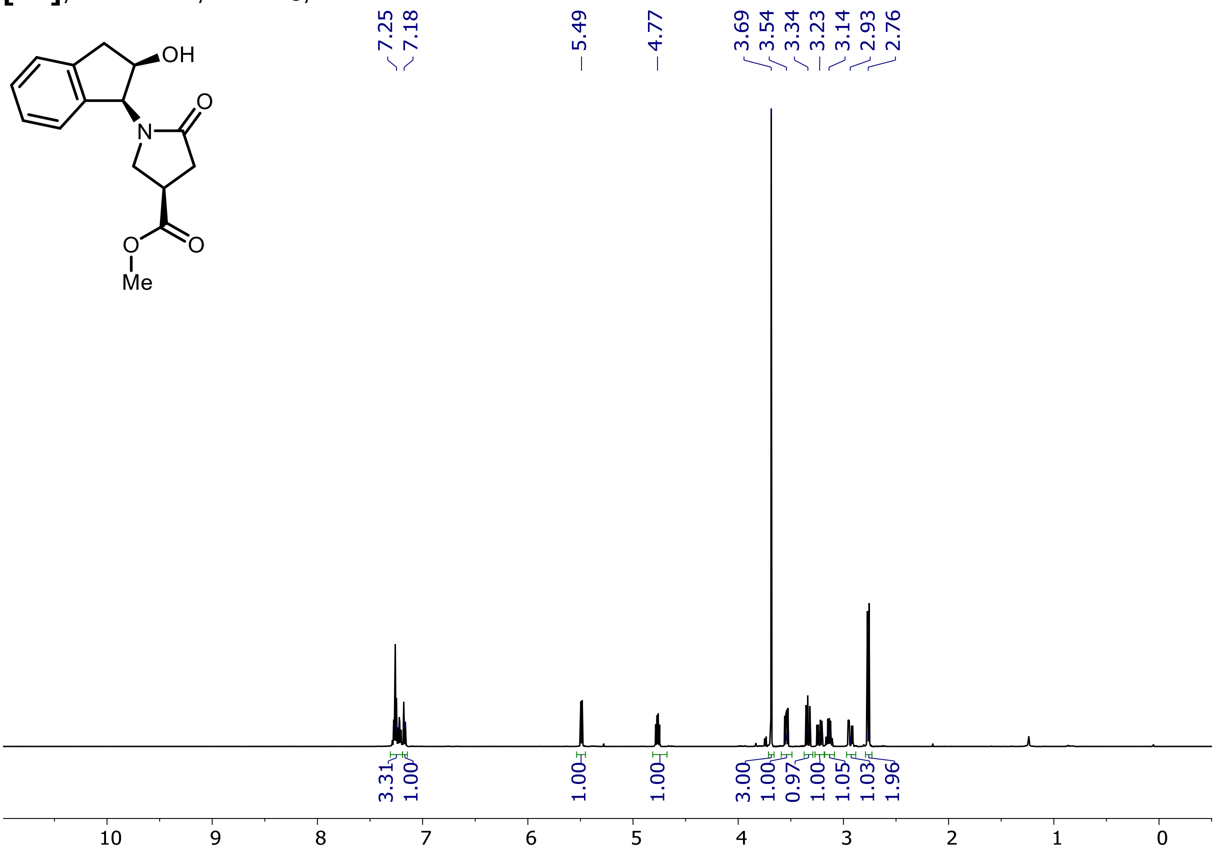


**[3n]**, $^{13}$C NMR, CDCl$_3$, 126 MHz

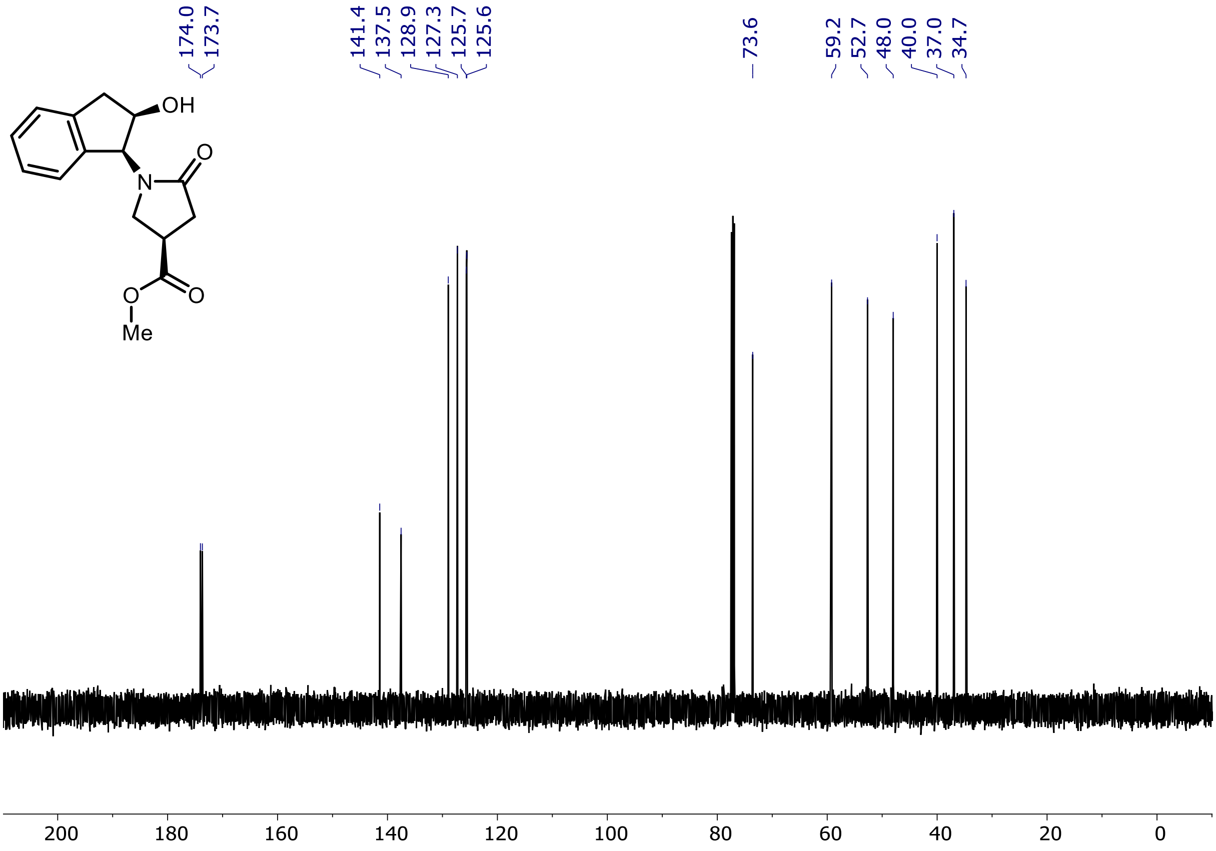

[***epi*-3n**], $^{1}$H NMR, $CDCl_3$, 600 MHz

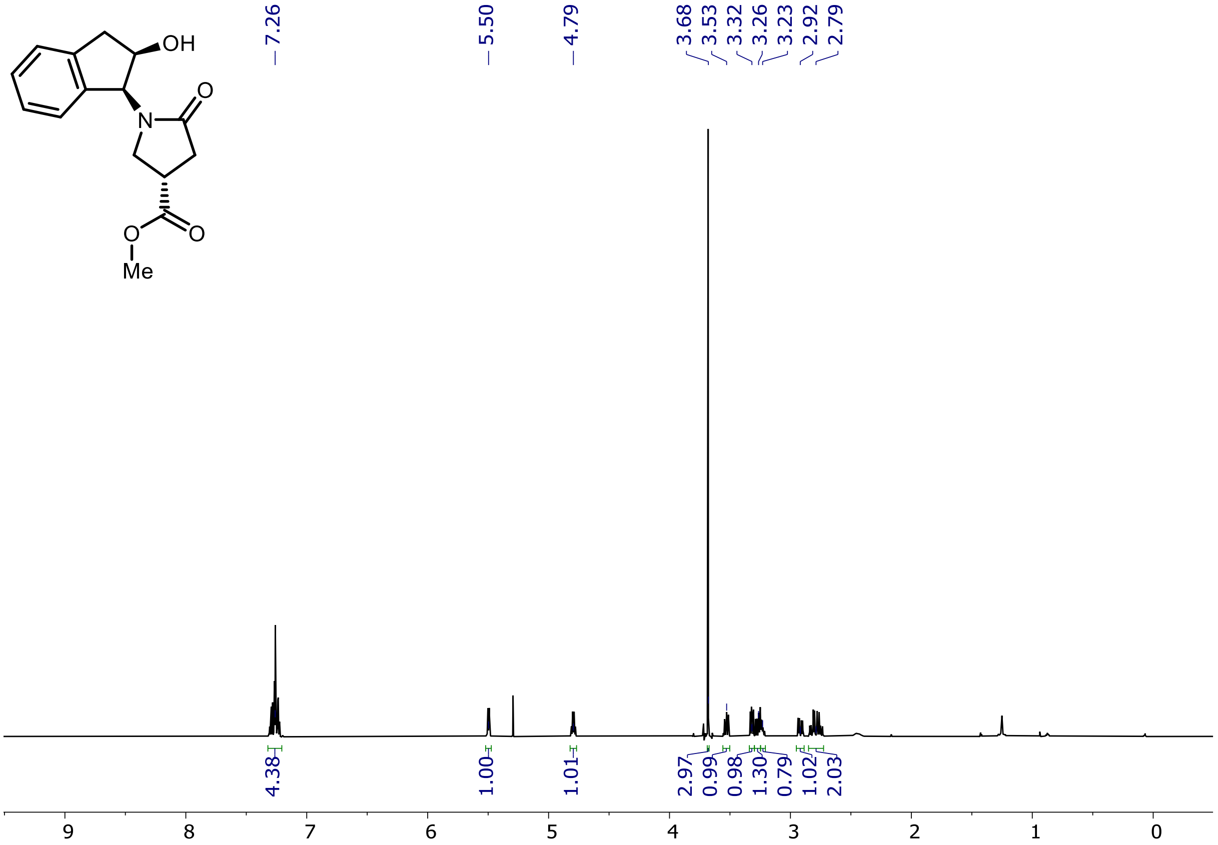


[***epi*-3n**], $^{13}$C NMR, $CDCl_3$, 126 MHz

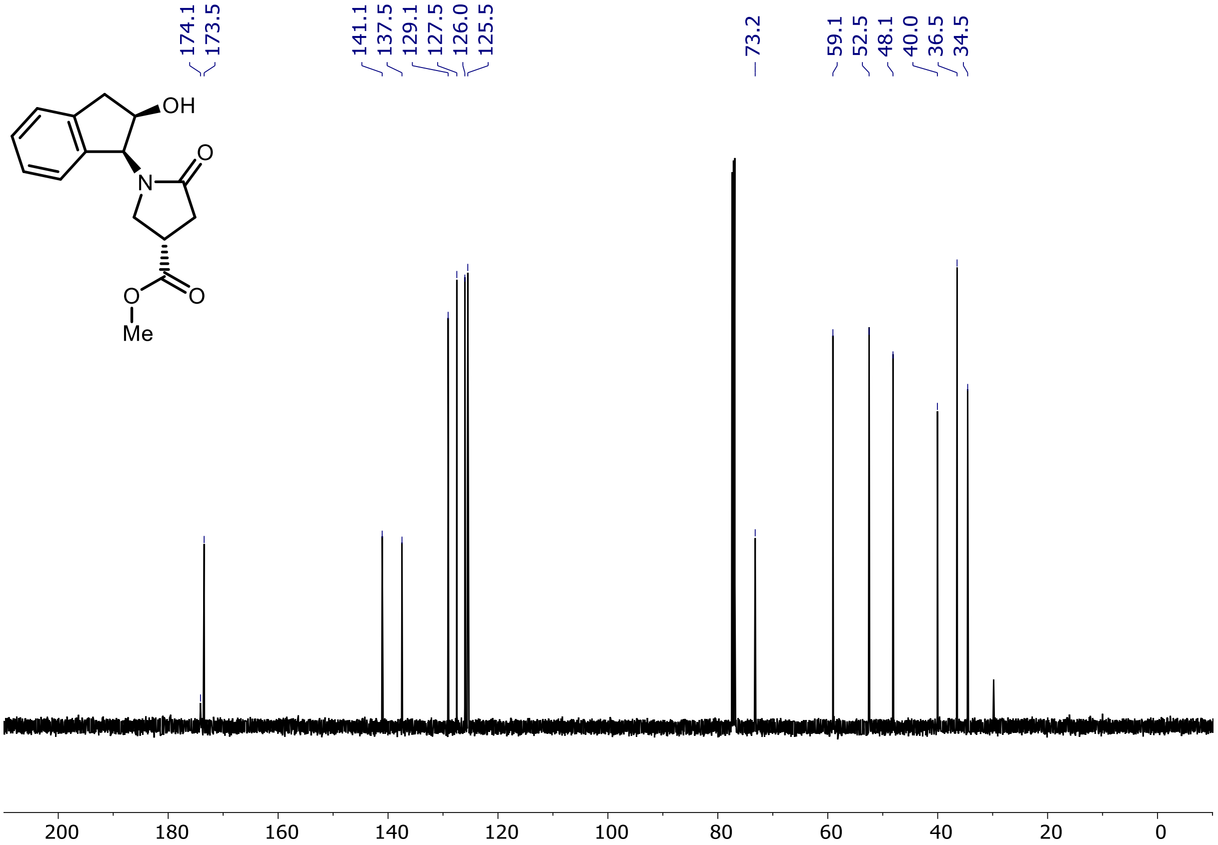

**[3o]**, $^{1}$H NMR, $CDCl_3$, 600 MHz

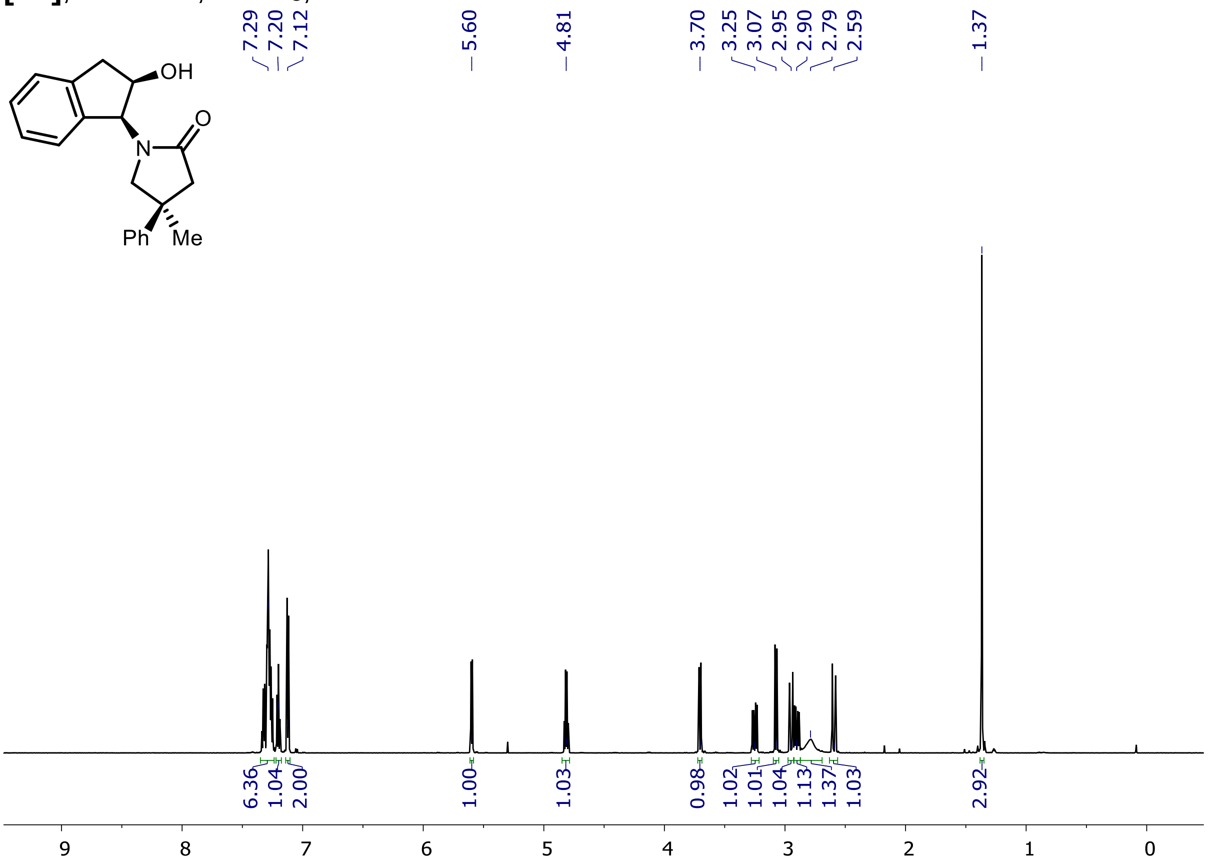


**[3o]**, $^{13}$C NMR, $CDCl_3$, 151 MHz

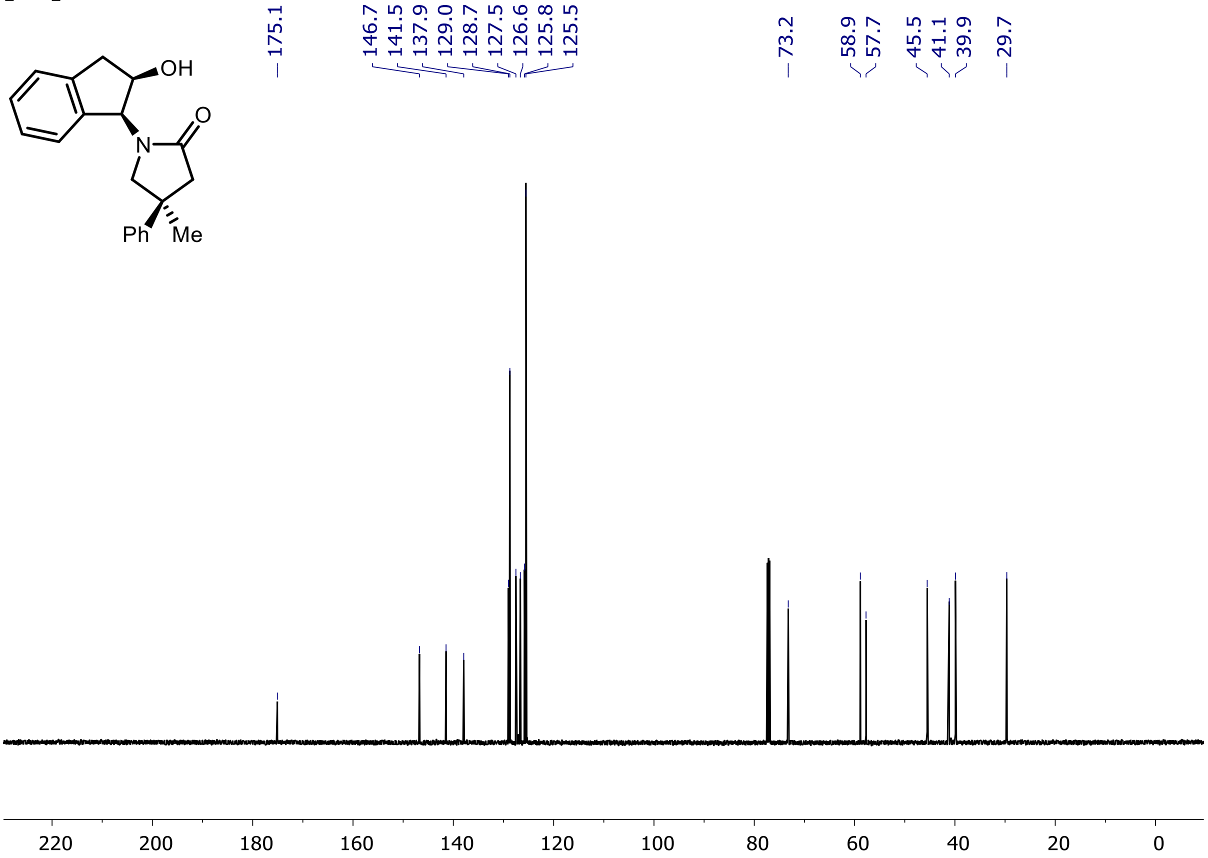

**[3p, *epi*-3p]**, $^{1}$H NMR, $CDCl_3$, 600 MHz

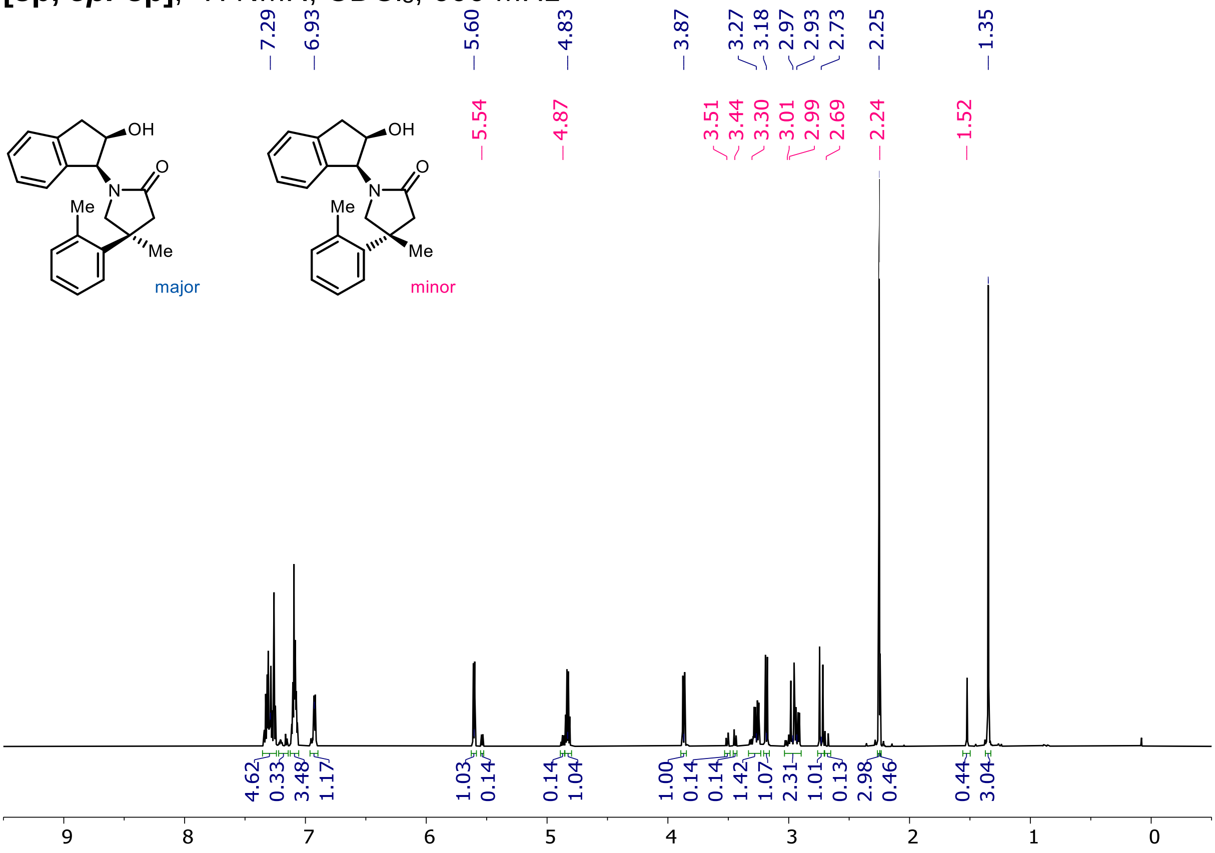


**[3p, *epi*-3p]**, $^{13}$C NMR, $CDCl_3$, 151 MHz

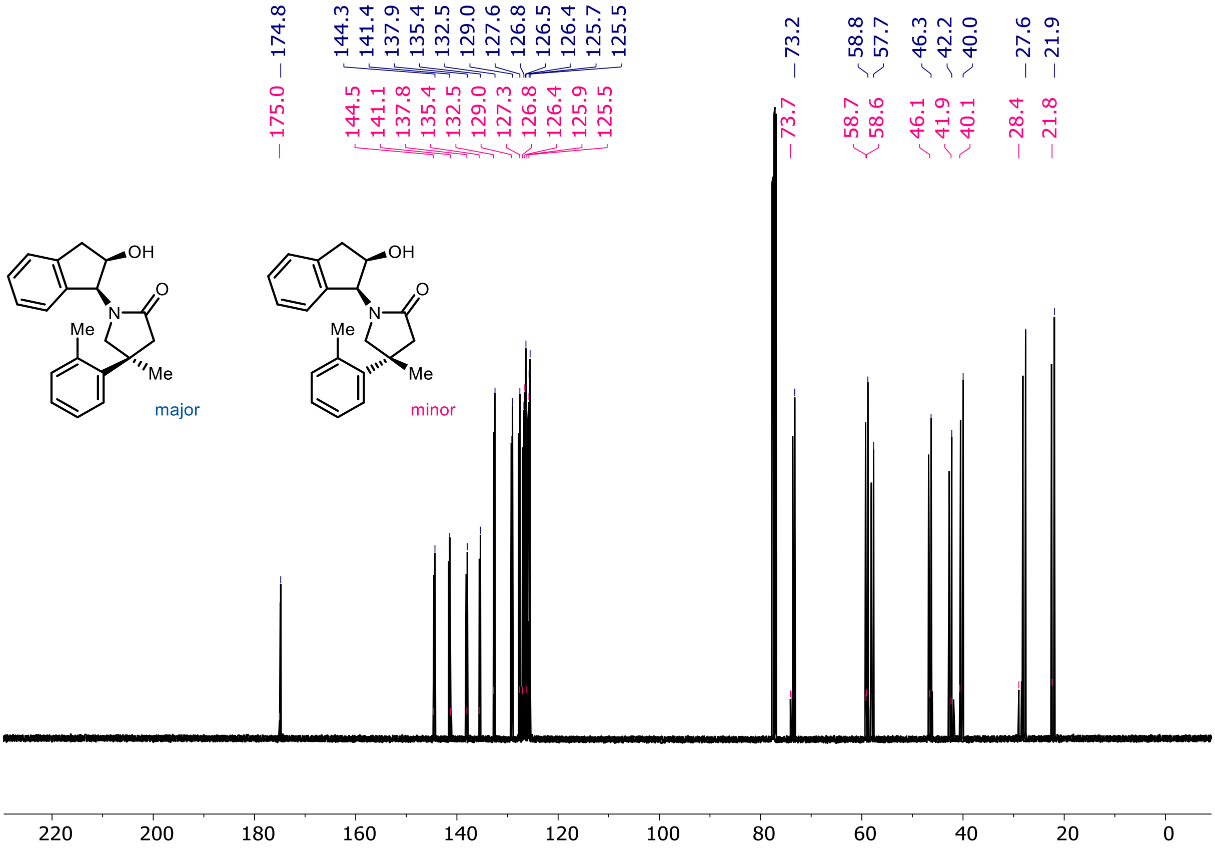

**[3q]**, $^{1}H$ NMR, $CDCl_3$, 500 MHz

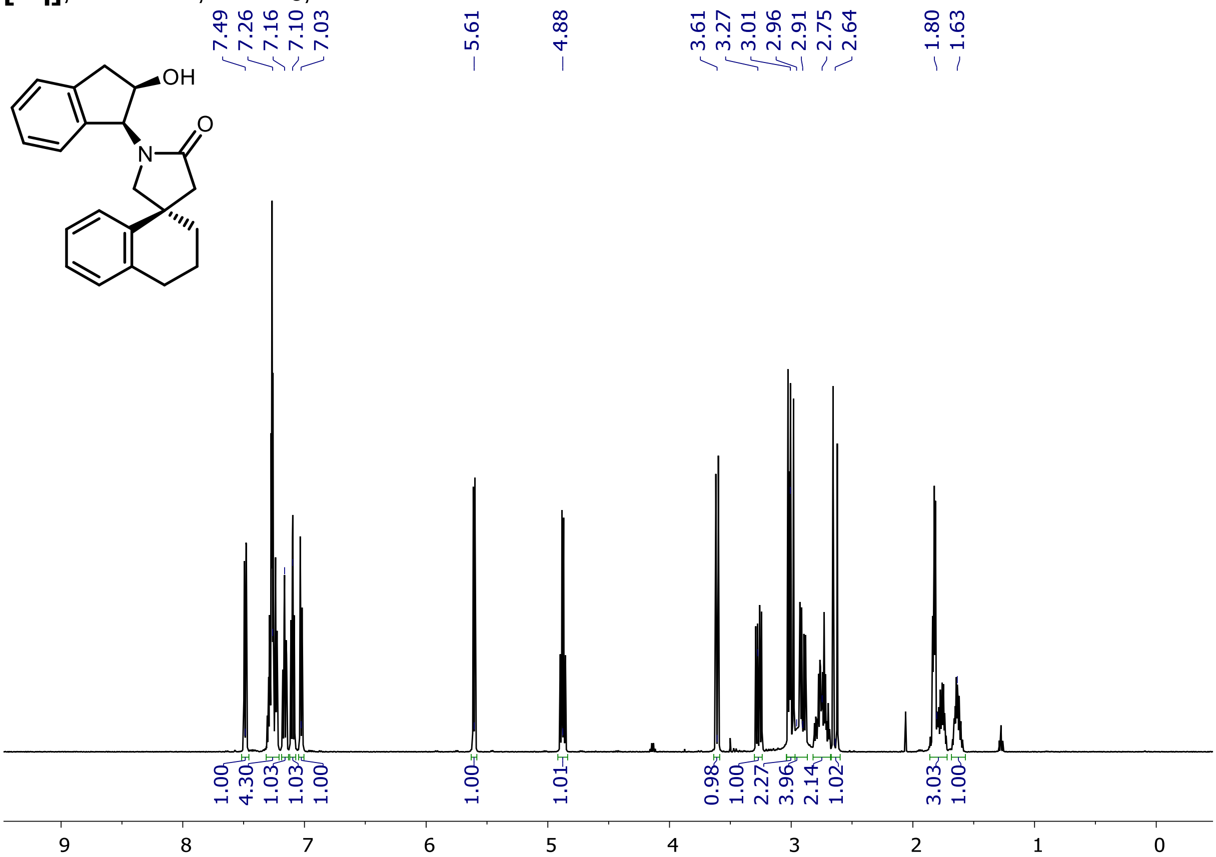


**[3q]**, $^{13}C$ NMR, $CDCl_3$, 126 MHz

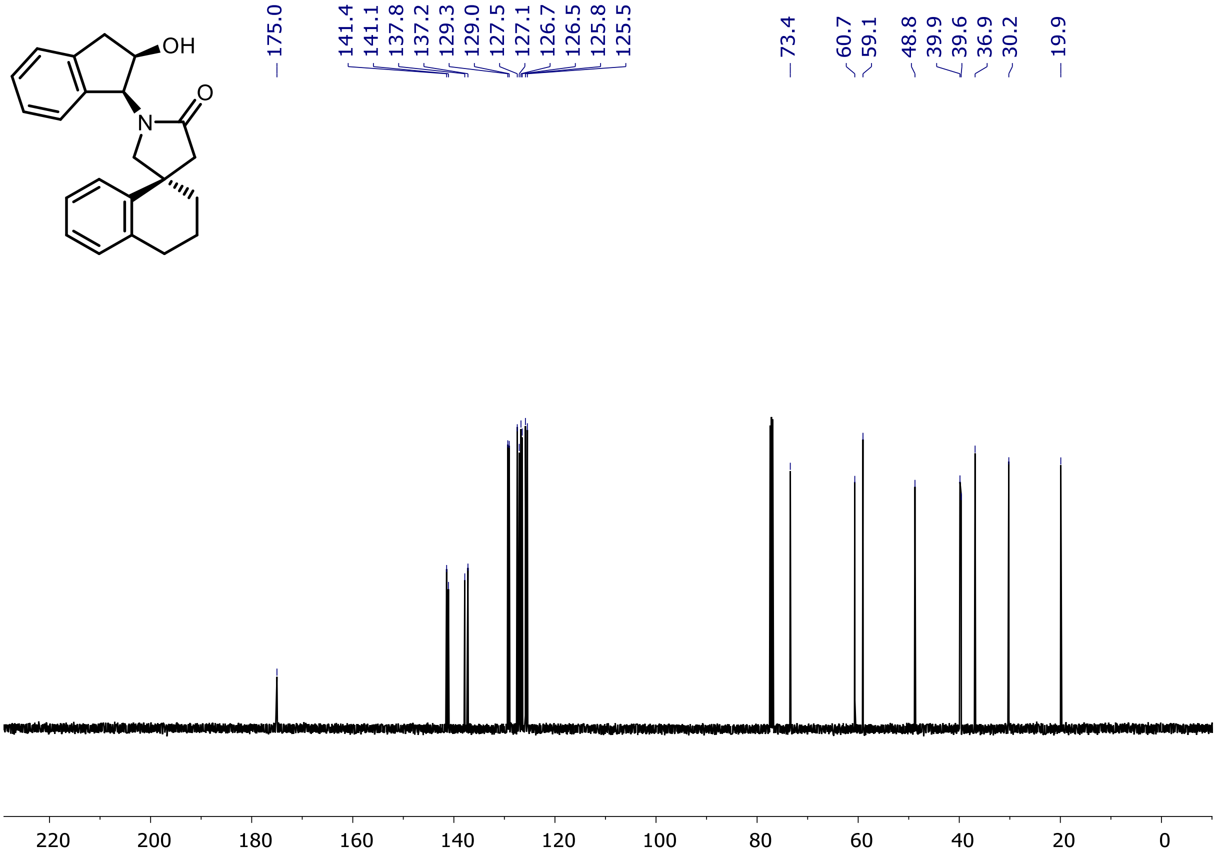

**[3r]**, $^{1}H$ NMR, $CDCl_3$, 600 MHz

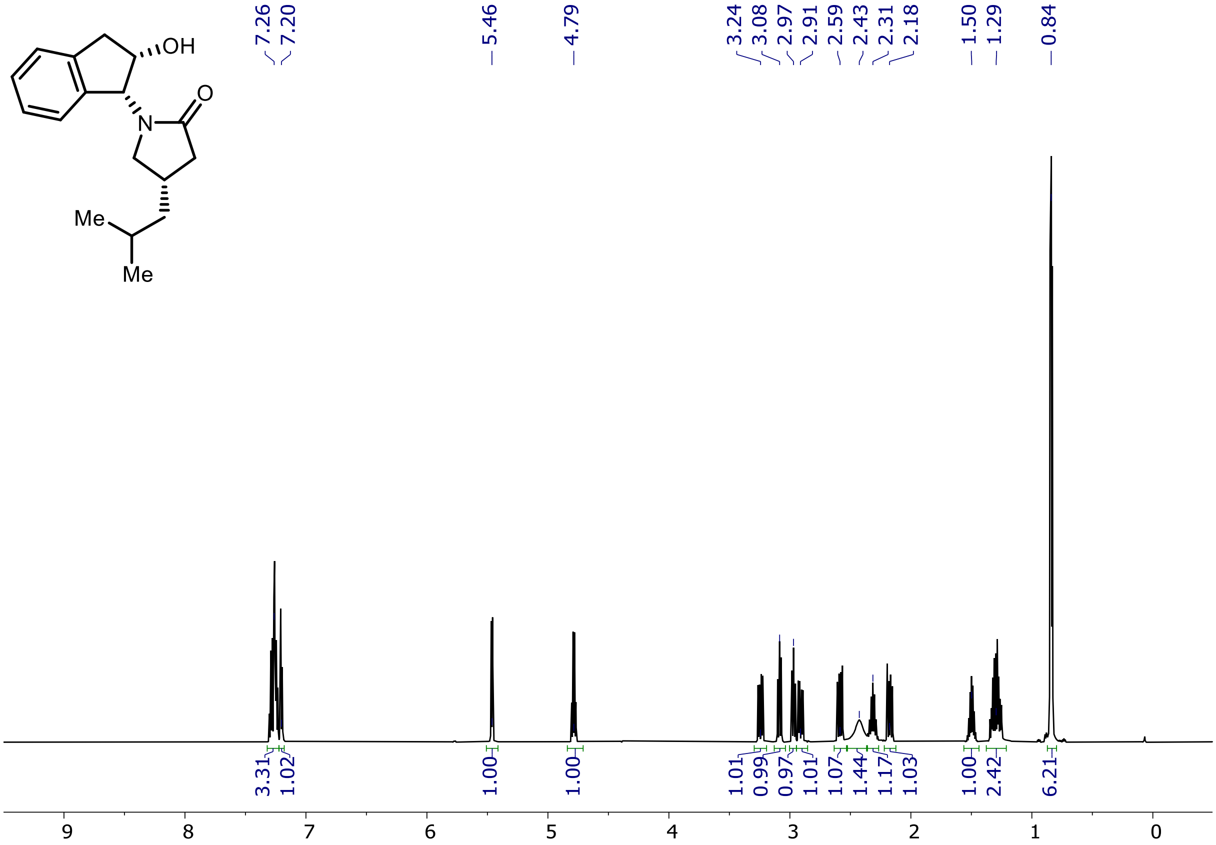


**[3r]**, $^{13}C$ NMR, $CDCl_3$, 150 MHz

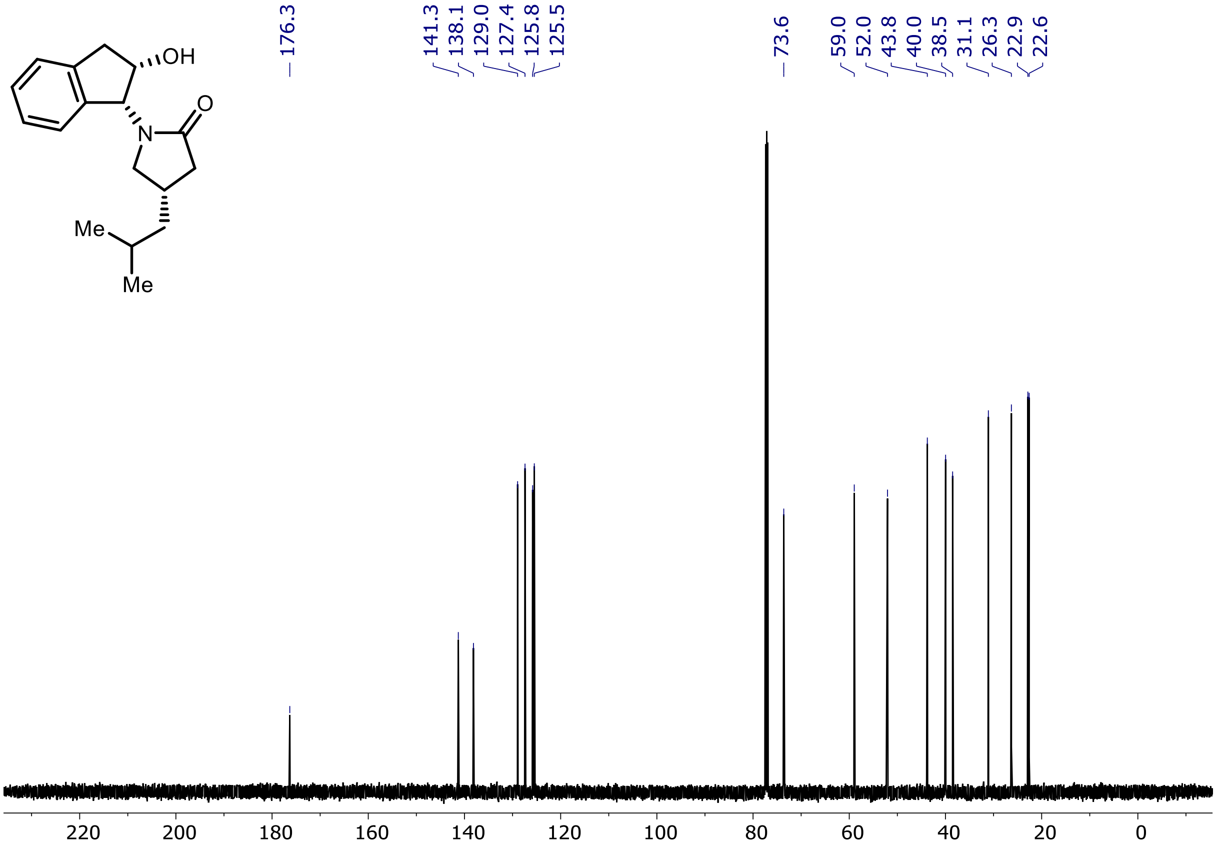

**[*epi*-3r]**, $^{1}H$ NMR, $CDCl_3$, 600 MHz

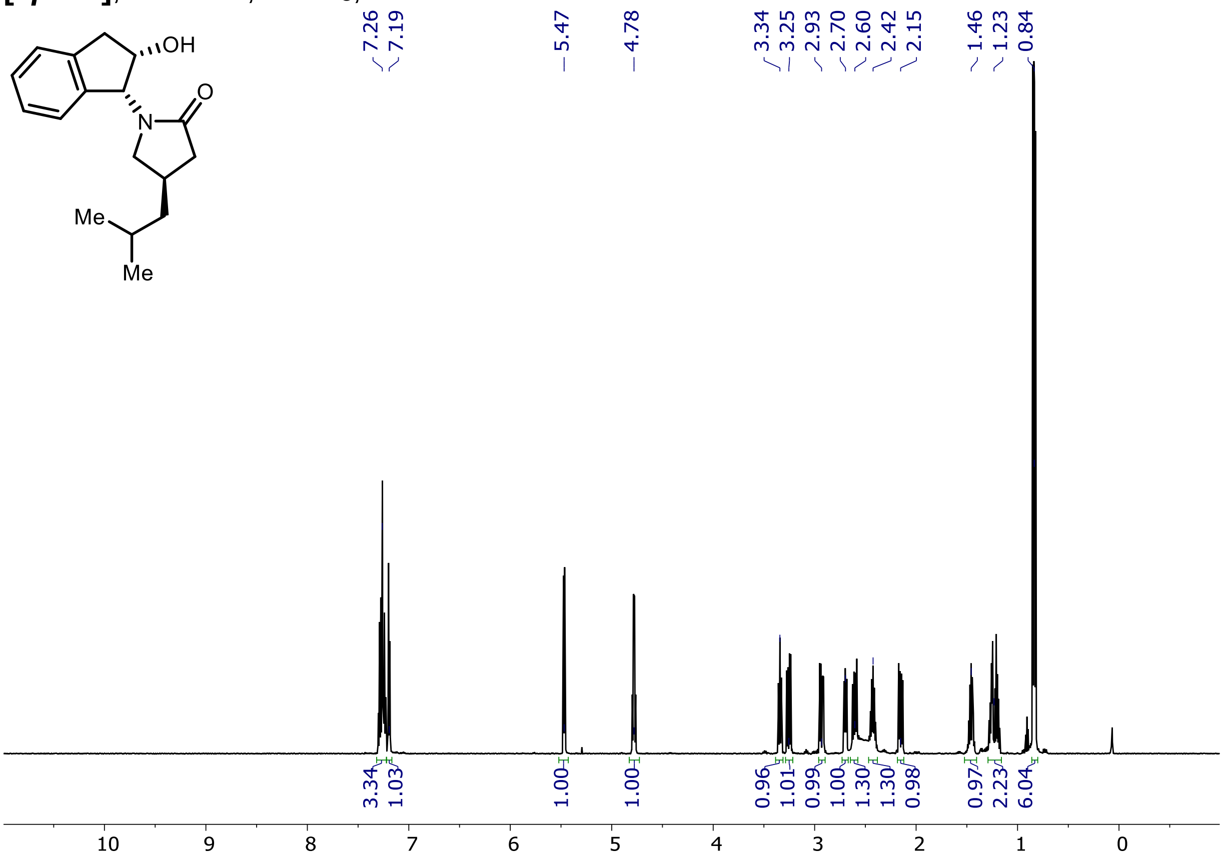


**[*epi*-3r]**, $^{13}C$ NMR, $CDCl_3$, 150 MHz

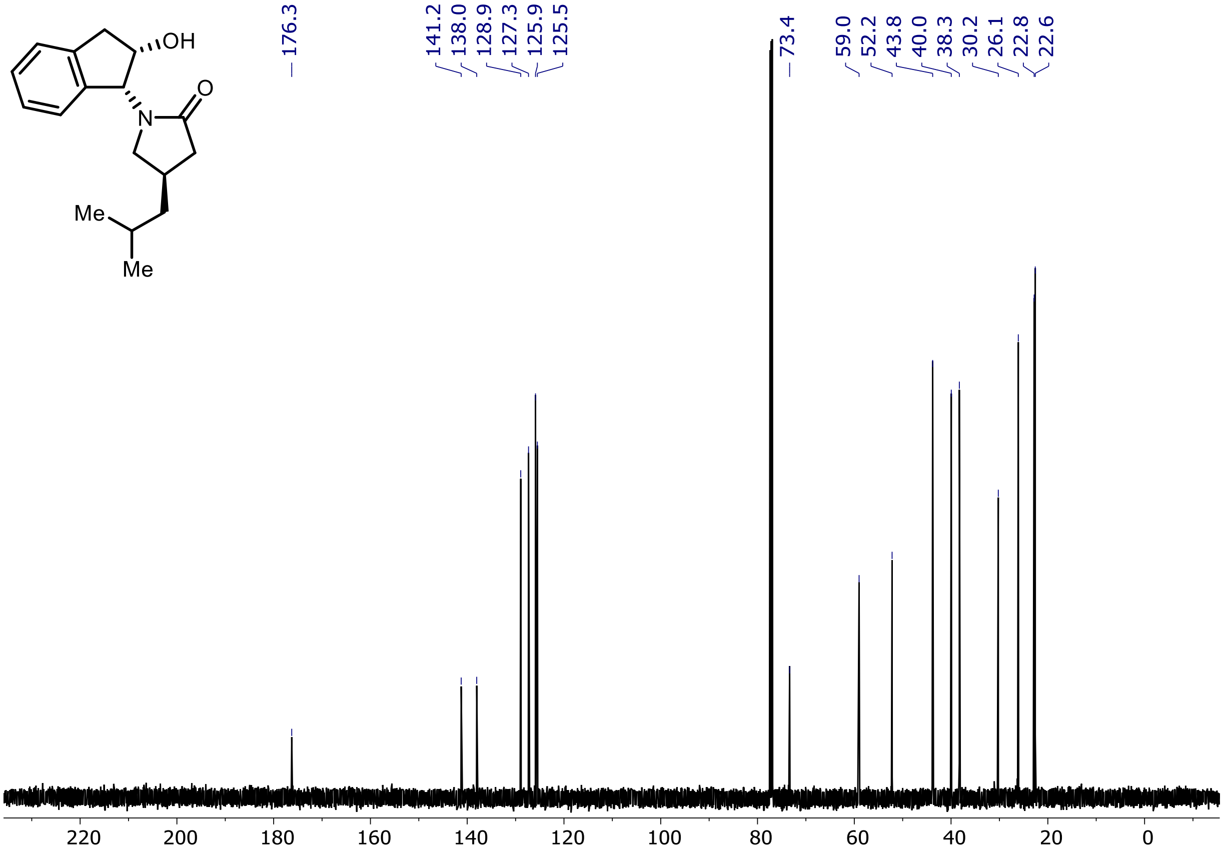

**[8a]**, $^{1}H$ NMR, $CDCl_3$, 600 MHz

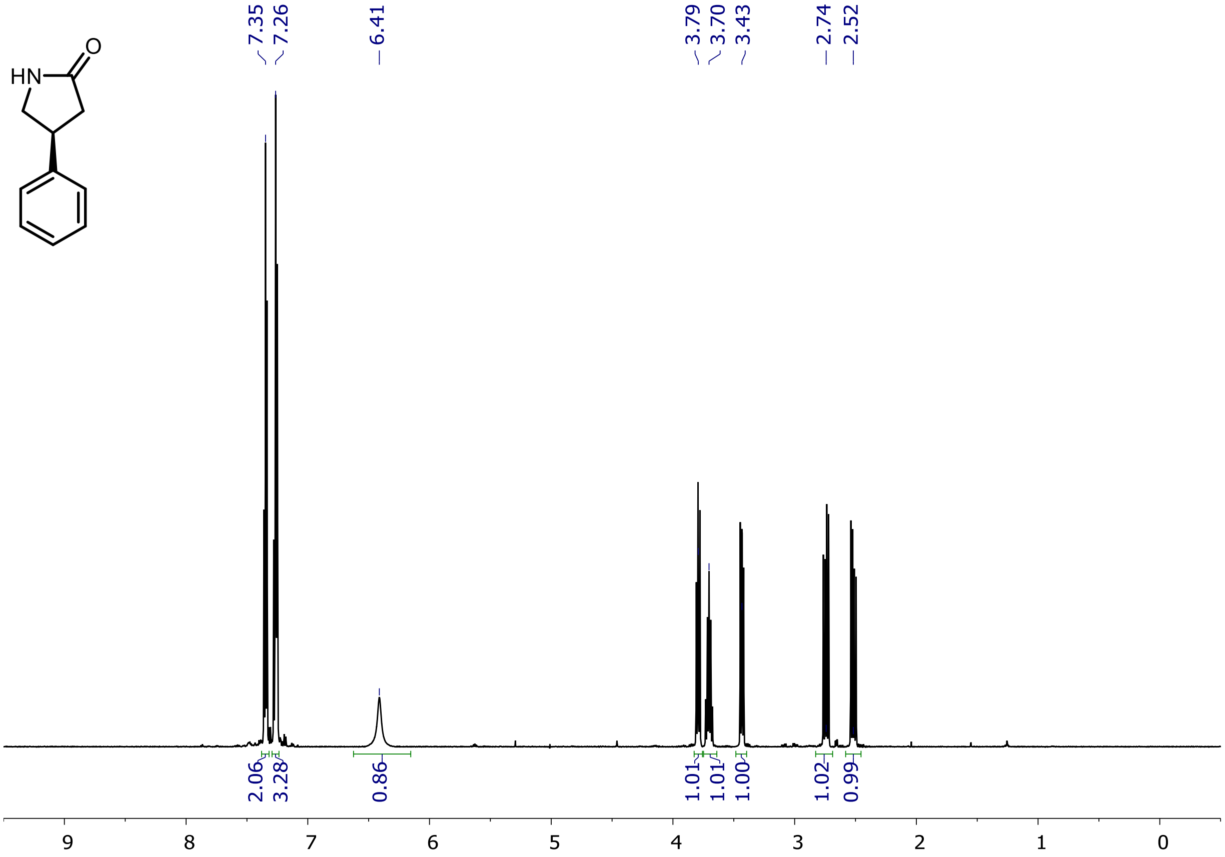


**[8a]**, $^{13}C$ NMR, $CDCl_3$, 151 MHz

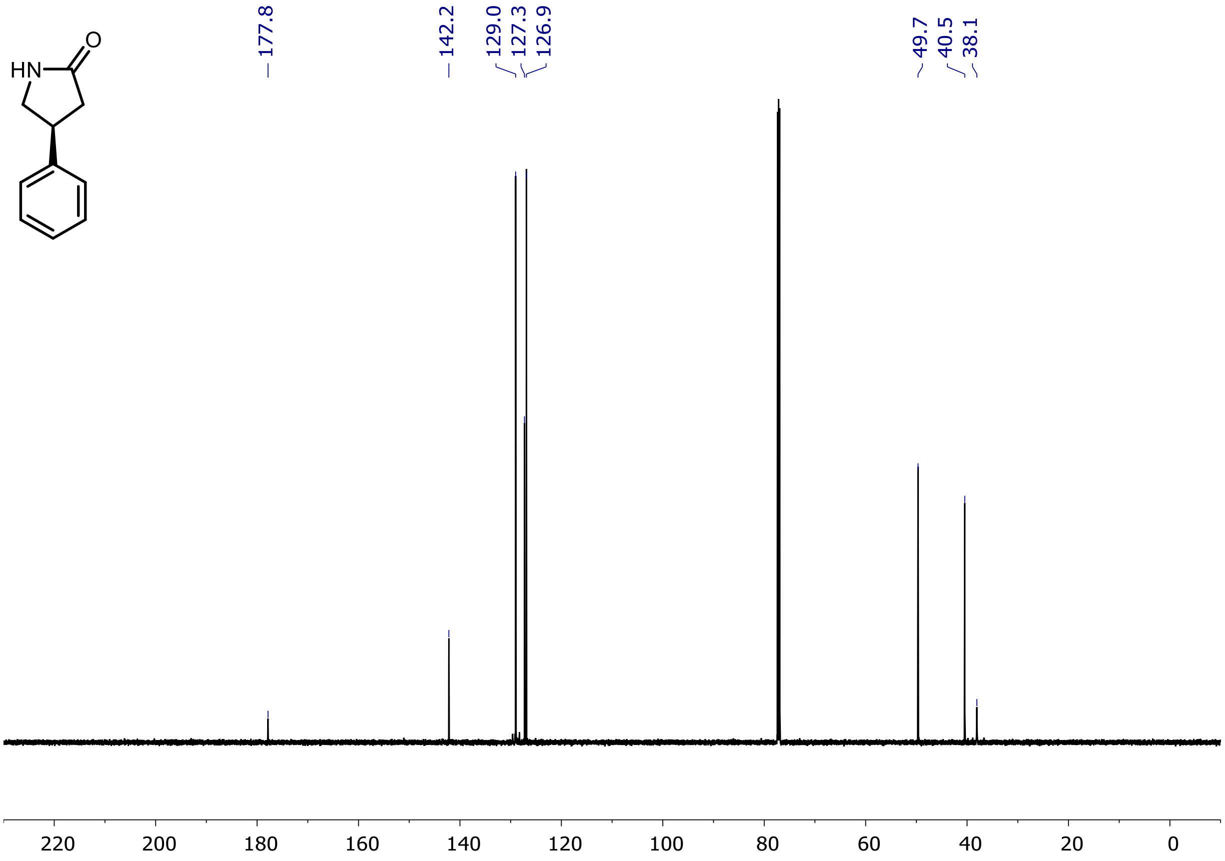

**[8a], [*epi*-8a]**

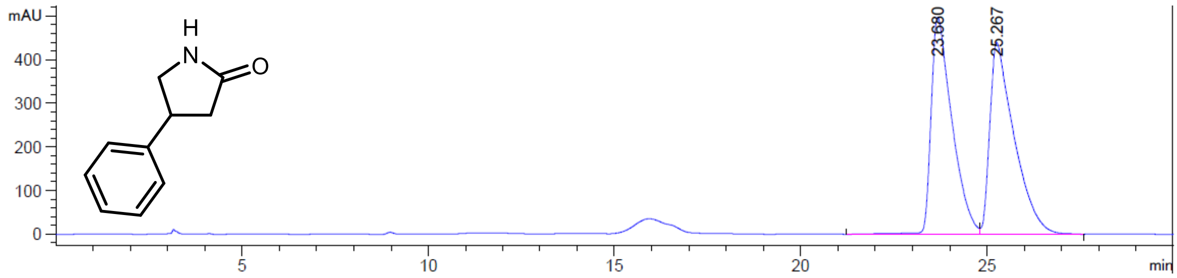


| Peak # | RetTime [min] | Type | Width [min] | Area [mAU*s] | Height [mAU] | Area % |
|---|---|---|---|---|---|---|
| 1 | 23.680 | BV | 0.5716 | 1.94712e4 | 498.59964 | 49.2504 |
| 2 | 25.267 | VB | 0.6427 | 2.00640e4 | 439.41449 | 50.7496 |

**[8a]**

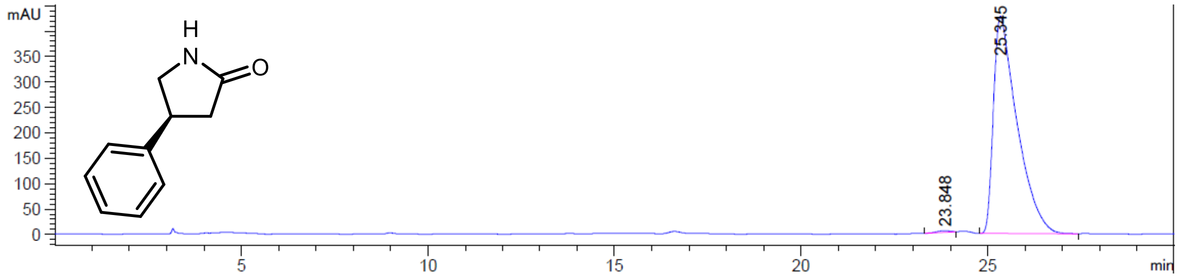


| Peak # | RetTime [min] | Type | Width [min] | Area [mAU*s] | Height [mAU] | Area % |
|---|---|---|---|---|---|---|
| 1 | 23.848 | BB | 0.3469 | 72.58104 | 3.08067 | 0.3830 |
| 2 | 25.345 | BB | 0.6386 | 1.88756e4 | 427.95706 | 99.6170 |

**[*epi*-8a]**

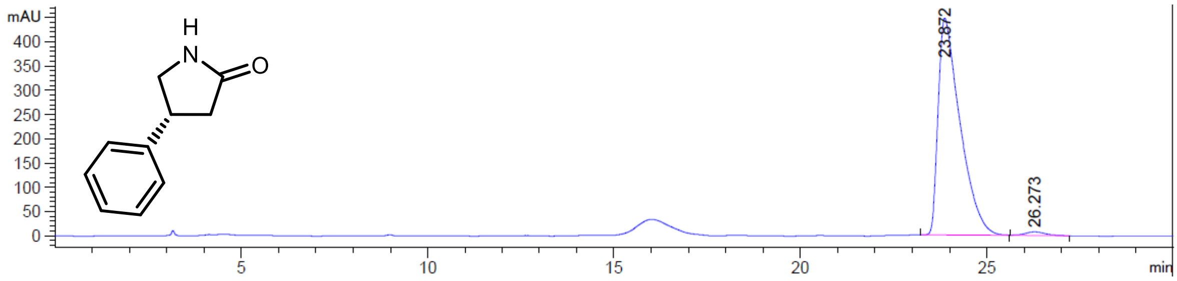


| Peak # | RetTime [min] | Type | Width [min] | Area [mAU*s] | Height [mAU] | Area % |
|---|---|---|---|---|---|---|
| 1 | 23.872 | BB | 0.5995 | 1.82374e4 | 447.44168 | 98.5950 |
| 2 | 26.273 | BB | 0.4711 | 259.88007 | 7.16652 | 1.4050 |

**[9a]**, $^1$H NMR, $CDCl_3$, 500 MHz

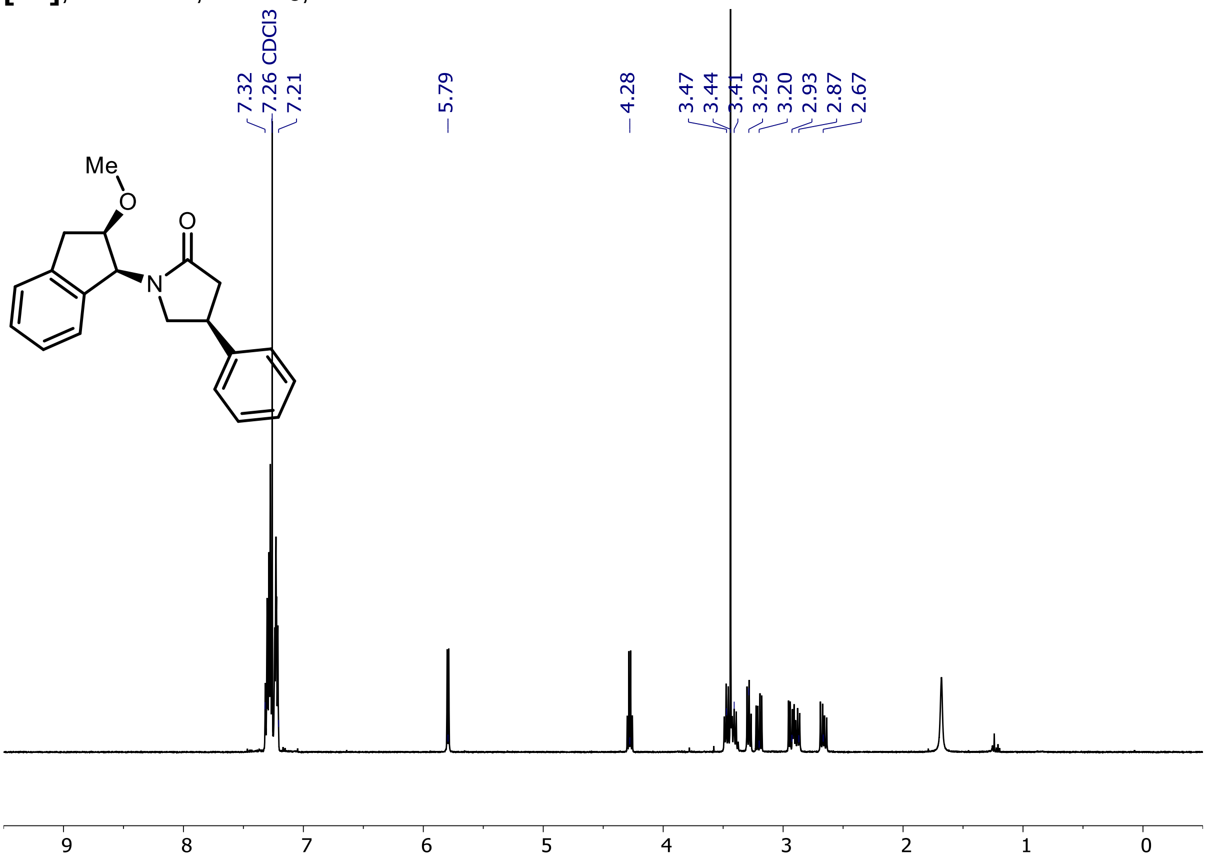


**[9a]**, $^{13}$C NMR, $CDCl_3$, 126 MHz

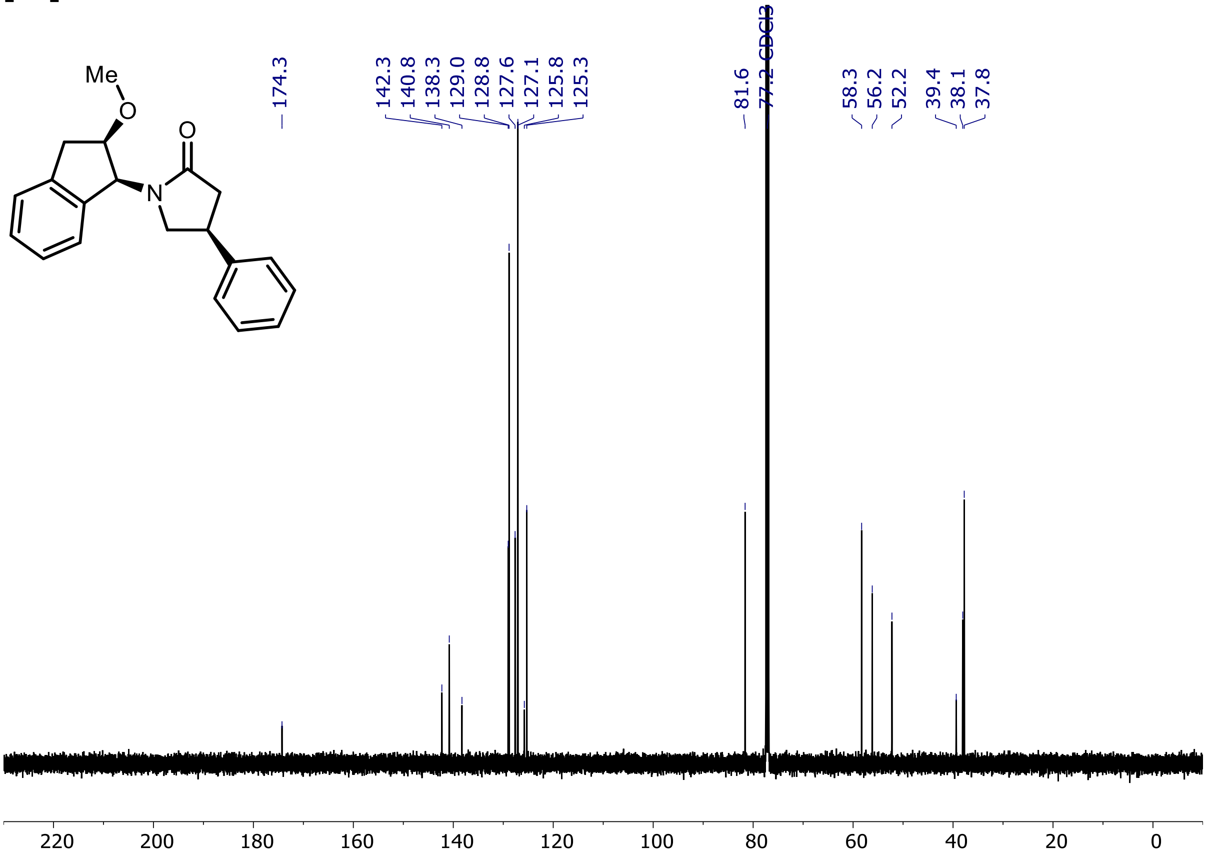

**[8f]**, $^{1}$H NMR, $CDCl_3$, 400 MHz

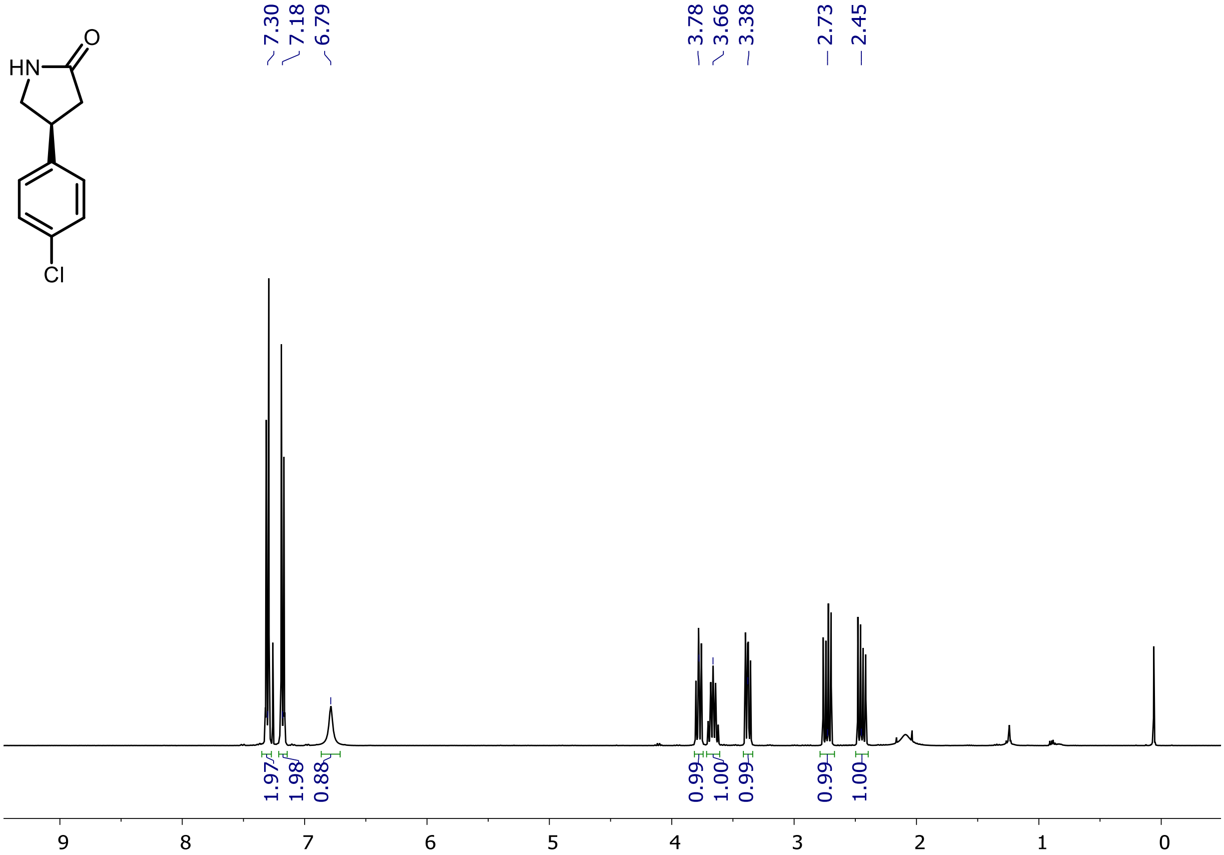


**[8f]**, $^{13}$C NMR, $CDCl_3$, 101 MHz

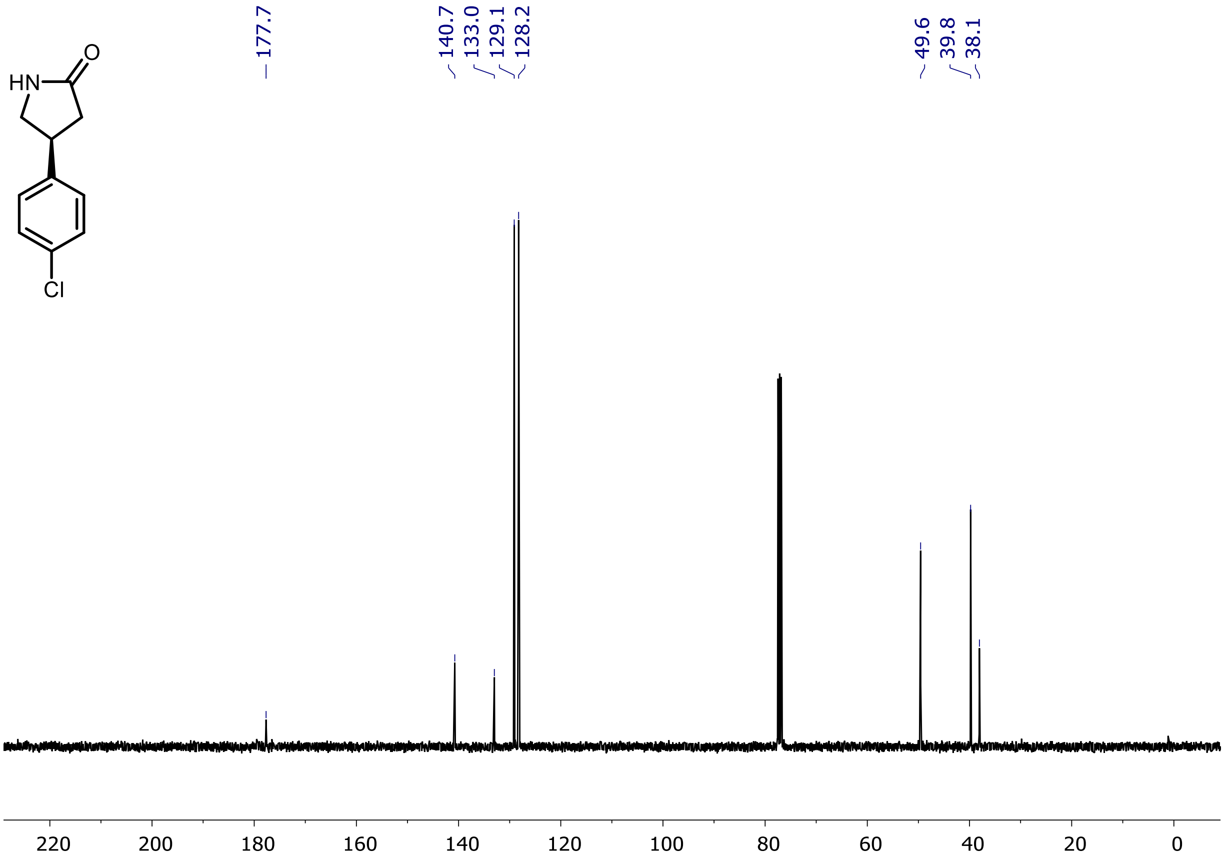

**[8h]**, $^{1}H$ NMR, $CDCl_3$, 400 MHz

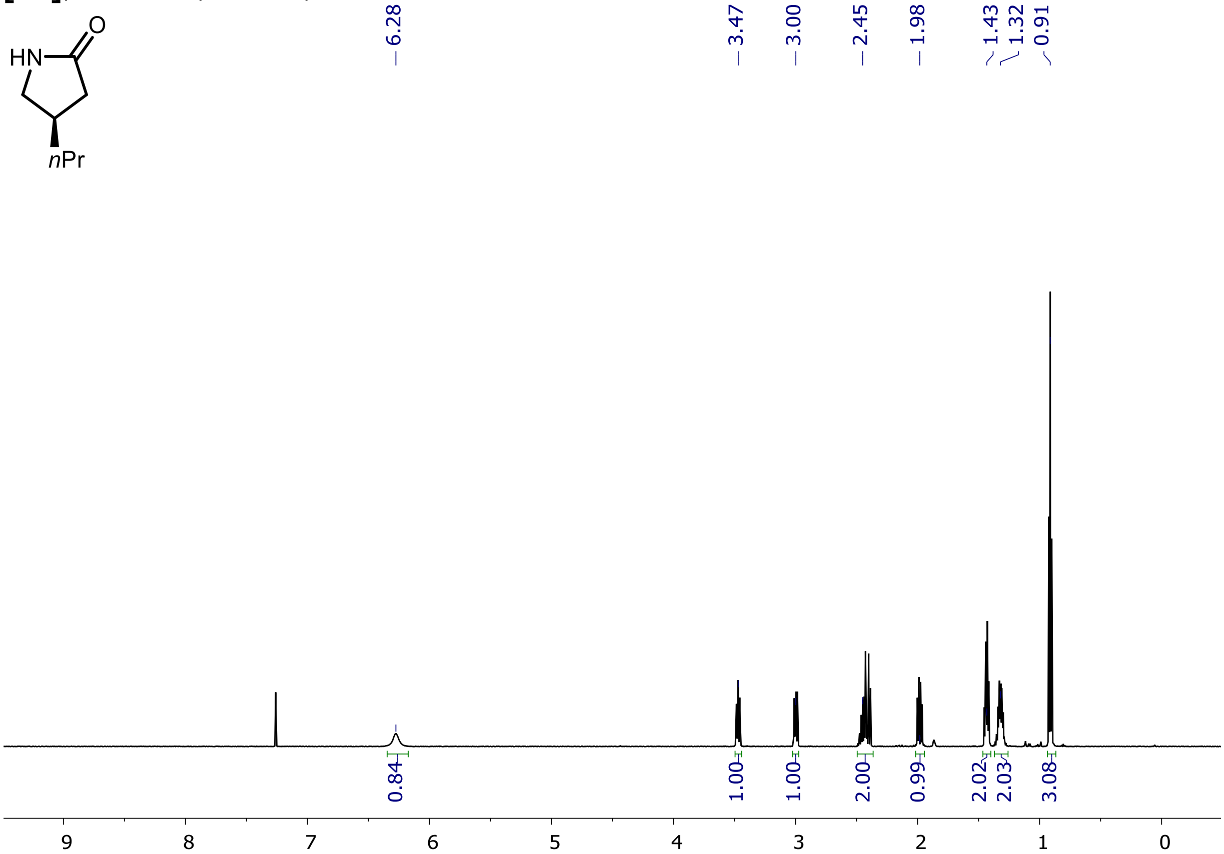


**[8h]**, $^{13}C$ NMR, $CDCl_3$, 101 MHz

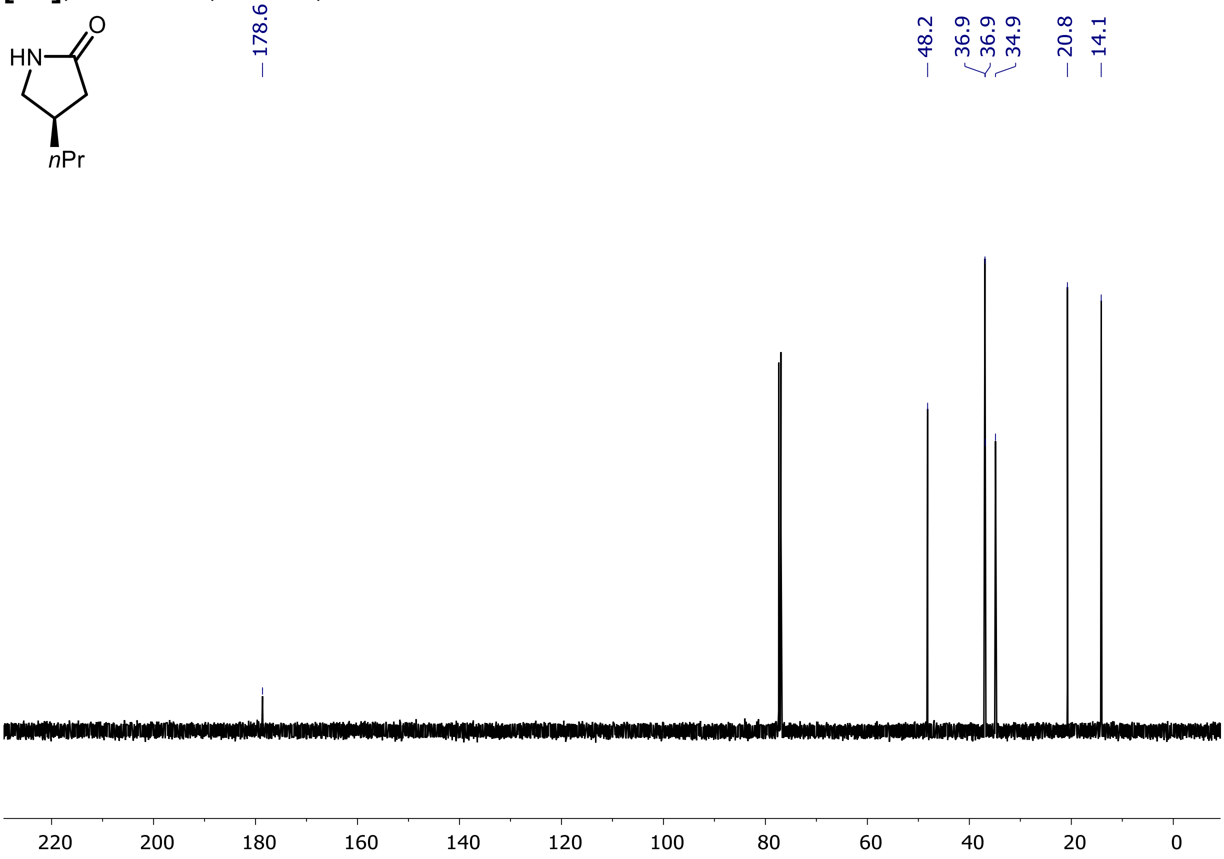

**[8r]**, $^{1}$H NMR, $CDCl_3$, 400 MHz

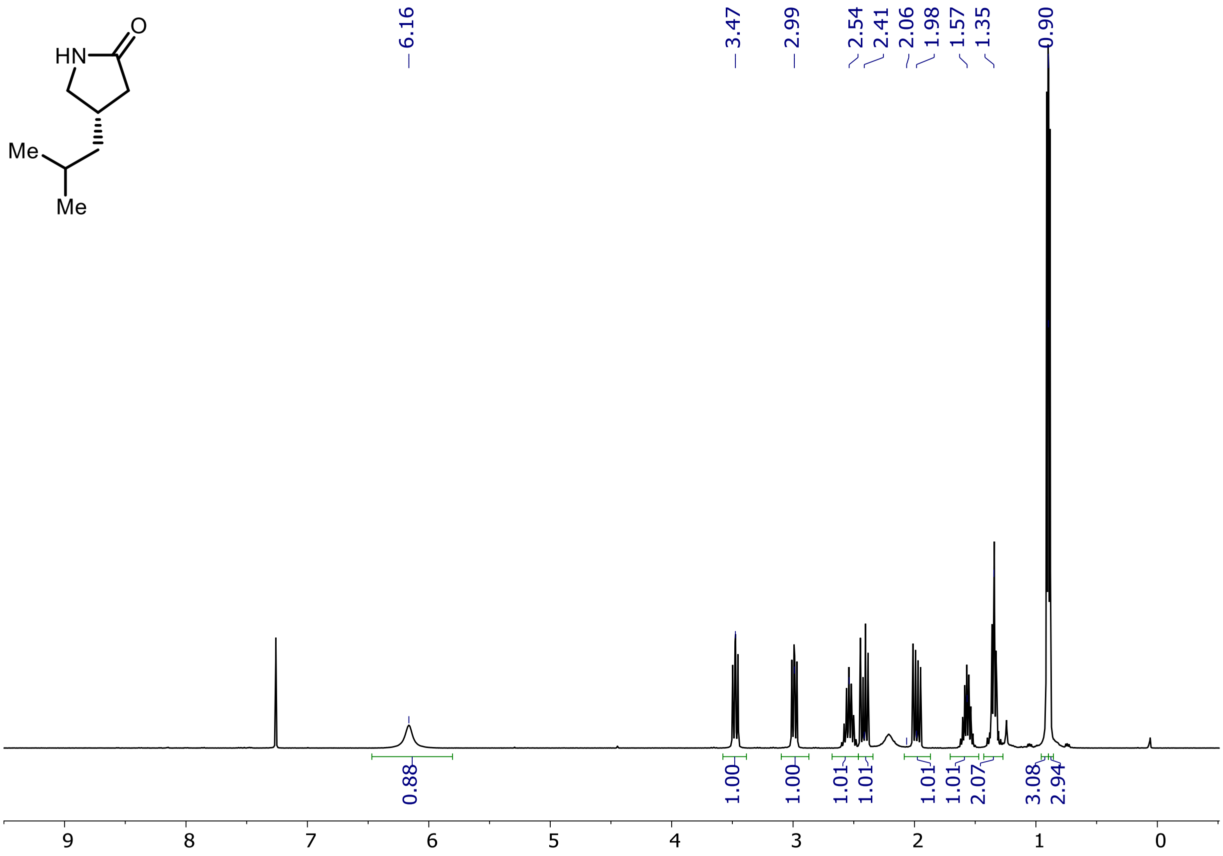


**[8r]**, $^{13}$C NMR, $CDCl_3$, 101 MHz

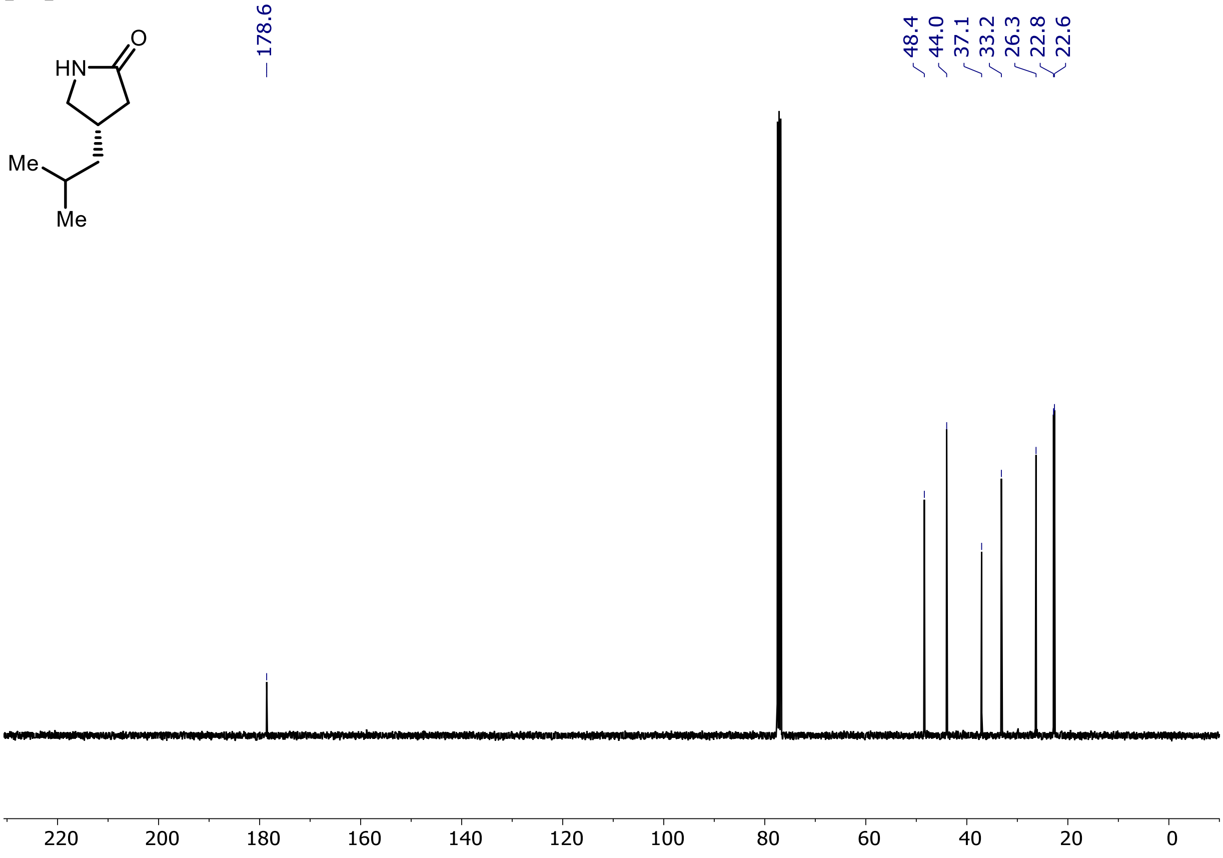

**[S5]**, $^{1}H$ NMR, $CDCl_3$, 400 MHz

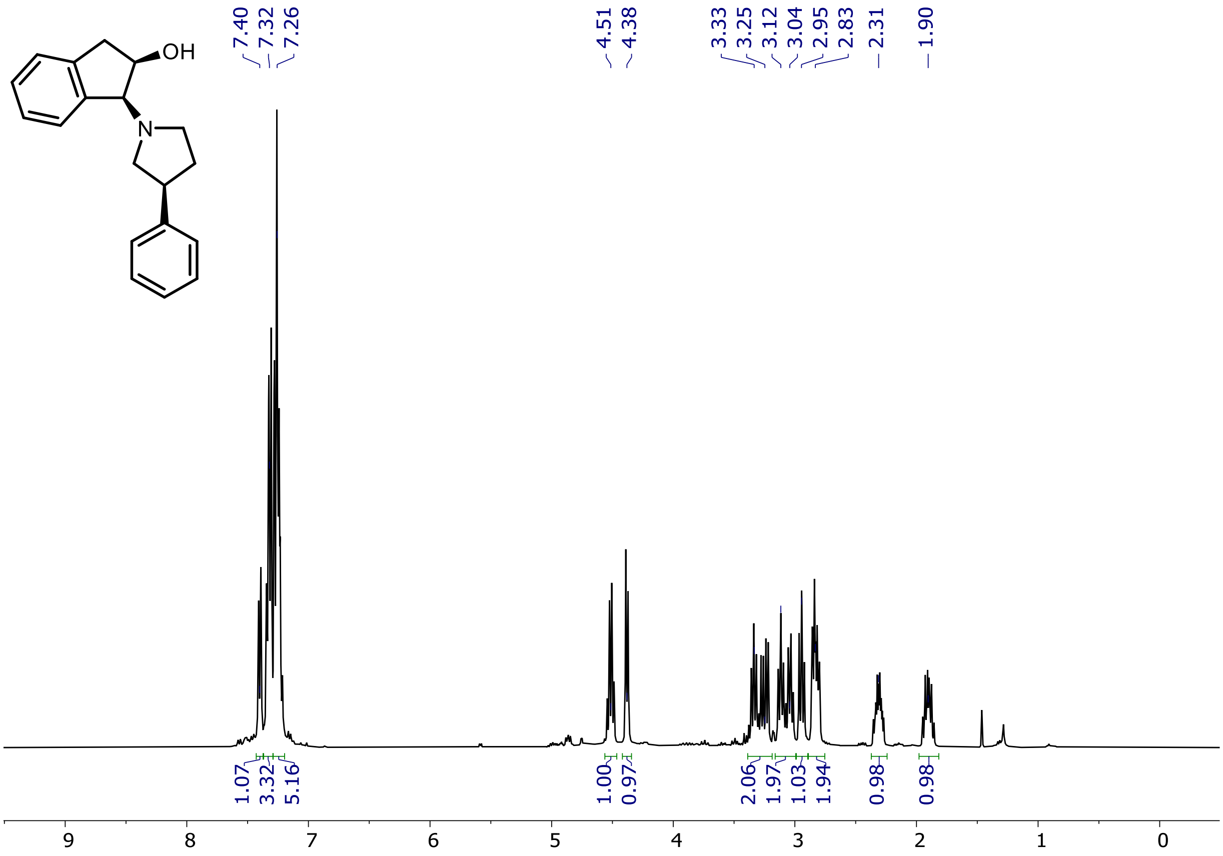


**[S5]**, $^{13}C$ NMR, $CDCl_3$, 101 MHz

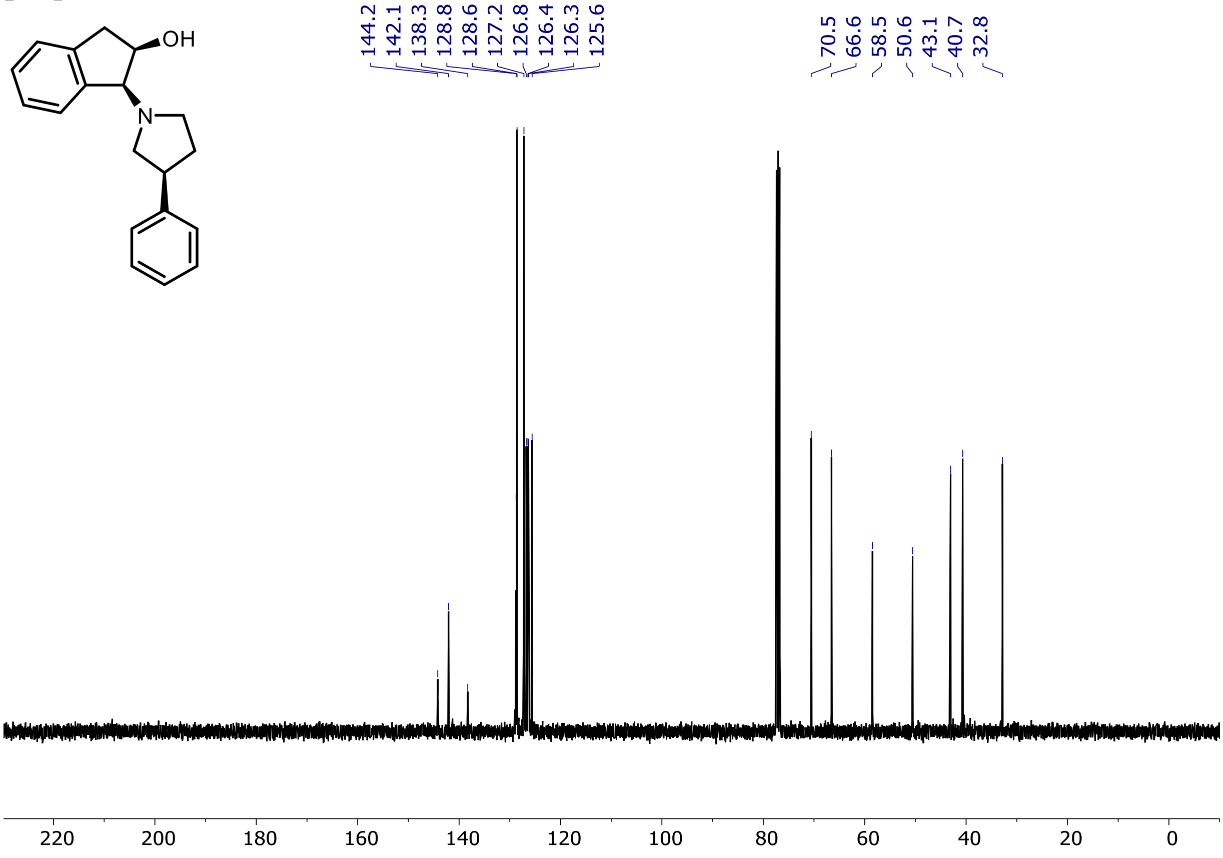

**[10a]**, $^{1}$H NMR, $CDCl_3$, 400 MHz

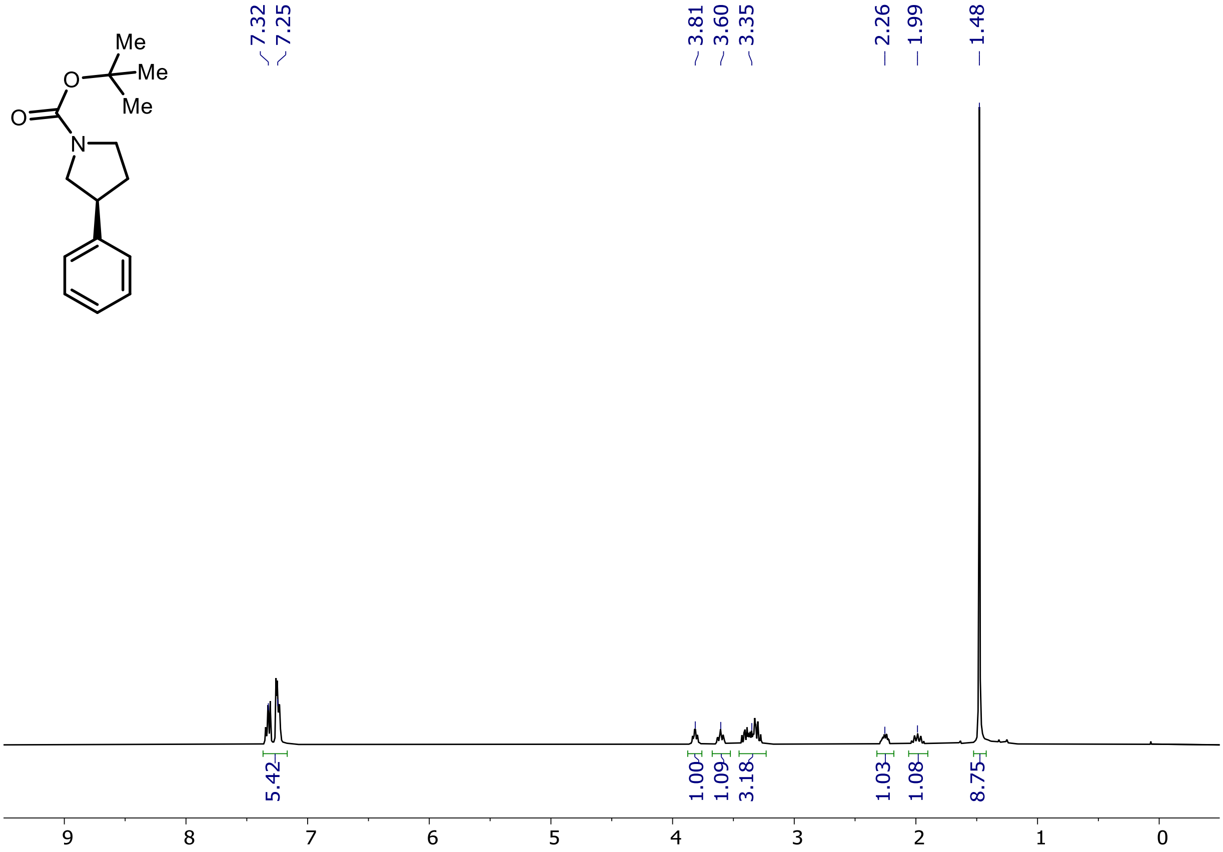


**[10a]**, $^{13}$C NMR, $CDCl_3$, 100 MHz

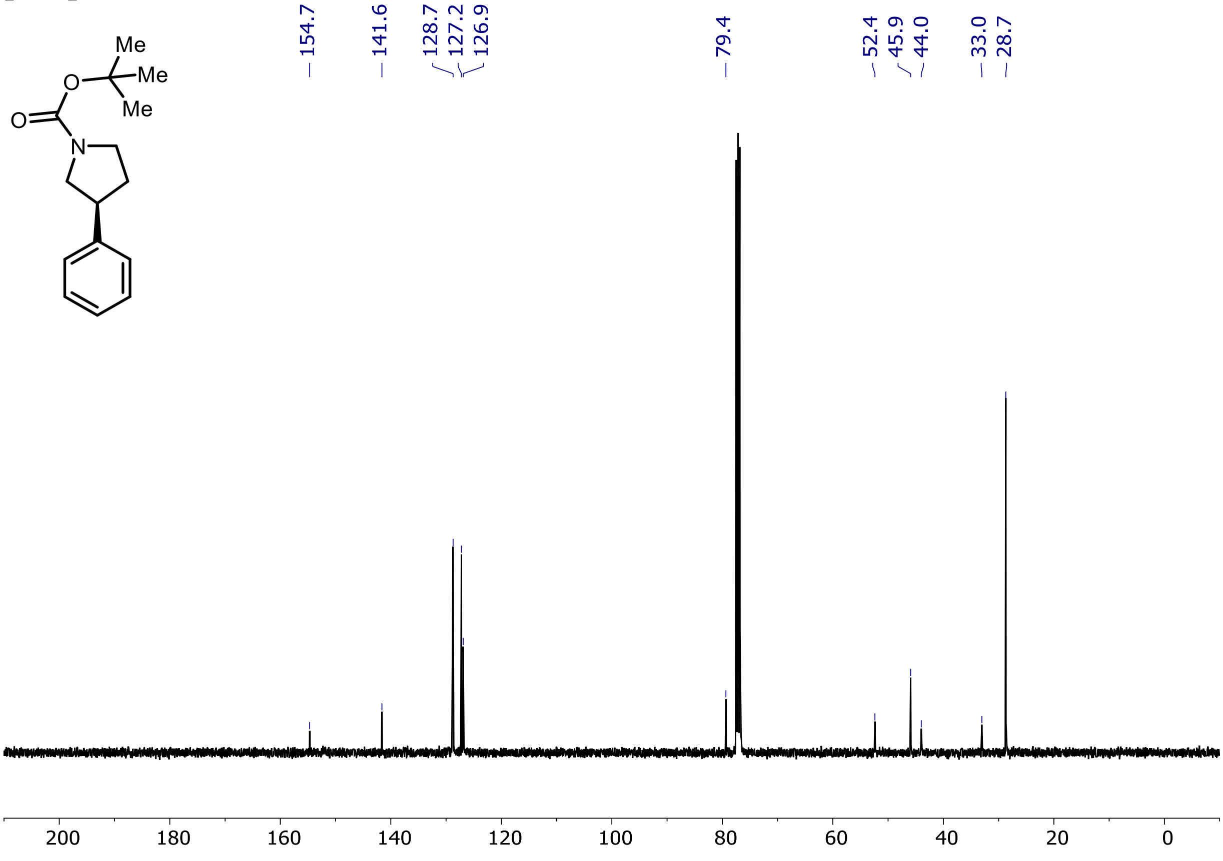

**DFT optimized (TPSS-D3/def2-TZVP) cartesian coordinates**

## [*epi*-4a]

E(TPSS-D3/def2-TZVP) = -865.9383912688 (conv)
Lowest Freq. = 23.68 cm^-1
40
epi-4a (001c1/opt)

| | | | |
|---|---|---|---|
| N | 1.8697967 | -1.3691913 | 0.7042681 |
| H | 2.1739664 | -1.1736545 | -0.2519238 |
| O | -0.2548699 | -2.1989398 | 0.2213483 |
| C | 1.9183430 | -2.8257715 | 0.9293266 |
| H | 2.7966940 | -3.2453195 | 0.4286457 |
| C | 0.5608839 | -3.3746349 | 0.3754935 |
| H | 0.6461700 | -3.8417891 | -0.6110683 |
| C | 0.0035689 | -4.3465406 | 1.4368104 |
| H | -1.0760495 | -4.2118561 | 1.5578299 |
| H | 0.1708213 | -5.3868081 | 1.1258011 |
| C | 0.7907229 | -4.0246087 | 2.6860206 |
| C | 0.5578199 | -4.4407463 | 3.9969248 |
| H | -0.2863463 | -5.0840545 | 4.2333006 |
| C | 1.4177681 | -4.0097103 | 5.0090594 |
| H | 1.2436472 | -4.3236187 | 6.0346599 |
| C | 2.4973716 | -3.1692208 | 4.7162341 |
| H | 3.1525530 | -2.8356046 | 5.5161175 |
| C | 2.7287569 | -2.7501444 | 3.4054464 |
| H | 3.5488663 | -2.0762239 | 3.1731227 |
| C | 1.8717277 | -3.1870989 | 2.3978238 |
| C | 0.4547556 | -1.0658435 | 0.7964464 |
| C | -0.0737609 | 0.2507190 | 0.2093494 |
| H | -0.5499418 | 0.1822448 | -0.7715282 |
| H | 0.7086575 | 1.0151806 | 0.1980546 |
| C | -1.0050781 | 0.4040066 | 1.4556998 |
| H | -1.9870310 | -0.0224104 | 1.2321518 |
| C | -0.1180111 | -0.6561331 | 2.1810517 |
| H | 0.6547642 | -0.1881539 | 2.7953446 |
| H | -0.6309950 | -1.4284773 | 2.7564312 |
| C | -1.1720690 | 1.7619509 | 2.0740538 |
| C | -0.0606259 | 2.5193440 | 2.4716539 |
| H | 0.9410714 | 2.1174343 | 2.3352112 |
| C | -0.2197045 | 3.7797392 | 3.0430136 |
| H | 0.6549479 | 4.3500679 | 3.3442997 |
| C | -1.4980062 | 4.3101075 | 3.2306673 |
| H | -1.6223660 | 5.2926151 | 3.6771215 |
| C | -2.6123436 | 3.5680325 | 2.8415239 |
| H | -3.6118154 | 3.9703772 | 2.9835288 |
| C | -2.4470343 | 2.3063223 | 2.2685760 |
| H | -3.3193921 | 1.7309784 | 1.9659901 |

## [4a]

E(TPSS-D3/def2-TZVP) = -865.9401717222 (conv)
Lowest Freq. = 26.62 cm^-1
40
4a (002c2/opt)

| | | | |
|---|---|---|---|
| N | -0.1320323 | -1.8442450 | 0.3784562 |
| H | -0.2738963 | -2.4335780 | 1.2009003 |
| O | 2.1924616 | -1.9523705 | 0.2407115 |
| C | 0.1875401 | -2.7131089 | -0.7691675 |
| H | -0.4033985 | -3.6321684 | -0.7021414 |
| C | 1.7308082 | -2.9569489 | -0.6819552 |
| H | 1.9982089 | -3.9338805 | -0.2659435 |

C 2.2997225 -2.7465312 -2.1006835
H 3.2400294 -2.1876405 -2.0623795
H 2.5125852 -3.7147881 -2.5738086
C 1.1997391 -2.0187289 -2.8387045
C 1.2494596 -1.3919186 -4.0840971
H 2.1719145 -1.3730387 -4.6594066
C 0.0989696 -0.7767562 -4.5812334
H 0.1269656 -0.2819058 -5.5482028
C -1.0894271 -0.7854227 -3.8422326
H -1.9747582 -0.2975191 -4.2403613
C -1.1383113 -1.4079733 -2.5944465
H -2.0488890 -1.3952213 -2.0015304
C 0.0119166 -2.0219579 -2.1030790
C 1.0916306 -1.0820367 0.5805489
C 1.0733475 0.3183961 -0.0893113
H 0.4057542 0.4717158 -0.9375658
H 2.0920320 0.6185047 -0.3492429
C 0.6488795 0.9127103 1.2892772
H -0.4424385 0.9328311 1.3561935
C 1.1862443 -0.3964820 1.9496445
H 2.2345031 -0.2922166 2.2409248
H 0.6146355 -0.8298421 2.7765553
C 1.2104986 2.2352515 1.7245073
C 2.5931512 2.4683583 1.7412161
H 3.2760153 1.6789697 1.4347963
C 3.1077024 3.6988176 2.1435198
H 4.1826599 3.8584922 2.1486685
C 2.2471230 4.7254773 2.5380719
H 2.6480984 5.6853326 2.8511171
C 0.8698739 4.5083916 2.5260561
H 0.1906051 5.3000000 2.8309596
C 0.3600444 3.2736758 2.1226473
H -0.7153193 3.1089156 2.1143377

**[*epi*-5a]**
E(TPSS-D3/def2-TZVP) = -1325.544272034 (conv)
Lowest Freq. = 23.56 cm^-1
40
epi-5a (003c1/opt)
N 1.8718566 -1.3528322 0.7038264
O -0.2331214 -2.2331163 0.1799790
C 1.9314917 -2.8205377 0.9312815
H 2.8405027 -3.2136928 0.4733583
C 0.6057161 -3.3939805 0.3456412
H 0.7187273 -3.8524422 -0.6399062
C 0.0462712 -4.3687393 1.4015580
H -1.0423689 -4.2813917 1.4722572
H 0.2746681 -5.4055362 1.1195605
C 0.7658615 -3.9884409 2.6744166
C 0.4979422 -4.3811895 3.9852676
H -0.3333810 -5.0458722 4.2064858
C 1.3064936 -3.8990355 5.0169833
H 1.1022603 -4.1927218 6.0428808
C 2.3743493 -3.0371360 4.7444675
H 2.9903126 -2.6681150 5.5594787
C 2.6462629 -2.6442119 3.4334704
H 3.4600510 -1.9590532 3.2138529
C 1.8352378 -3.1272992 2.4090651
C 0.4303740 -1.0935417 0.7474340
C -0.1252156 0.2191007 0.1883510
H -0.6293716 0.1441165 -0.7761717

H 0.6569535 0.9804540 0.1452721
C -1.0128252 0.3666596 1.4658696
H -2.0032579 -0.0578183 1.2794882
C -0.1036449 -0.6929126 2.1599129
H 0.6818930 -0.2297854 2.7601550
H -0.5927370 -1.4827262 2.7314009
epi-6aC -1.1517127 1.7215374 2.0979532
C -0.0231269 2.4830211 2.4340111
H 0.9715389 2.0899768 2.2347283
C -0.1565241 3.7392050 3.0209231
H 0.7306055 4.3136203 3.2736877
C -1.4250819 4.2605042 3.2844264
H -1.5292379 5.2406704 3.7409664
C -2.5558821 3.5138794 2.9563255
H -3.5477073 3.9096910 3.1574148
C -2.4167774 2.2559983 2.3683308
H -3.3017249 1.6770347 2.1130195
Cl 2.4414784 -1.0595486 -0.9951538

**[5a]**
E(TPSS-D3/def2-TZVP) = -1325.544721561 (conv)
Lowest Freq. = 26.37 cm^-1
40
5a (006c3/opt)
N 1.7357543 -1.4159574 0.4908382
O -0.2325749 -2.6182897 0.0943365
C 2.0127494 -2.8455454 0.8088350
H 2.9496628 -3.1323703 0.3296759
C 0.7637110 -3.6366941 0.3142596
H 0.9151886 -4.1520725 -0.6372512
C 0.3661034 -4.5916984 1.4587759
H -0.7215822 -4.6240786 1.5748589
H 0.7028145 -5.6130819 1.2361471
C 1.0862398 -4.0447095 2.6690716
C 0.9314773 -4.3892030 4.0117584
H 0.1972670 -5.1322411 4.3128357
C 1.7281256 -3.7599520 4.9704106
H 1.6110927 -4.0159747 6.0197722
C 2.6757044 -2.8010428 4.5944312
H 3.2874213 -2.3216655 5.3532747
C 2.8326491 -2.4542245 3.2523208
H 3.5498187 -1.6952515 2.9526997
C 2.0283963 -3.0797998 2.3016551
C 0.2672897 -1.3644608 0.5545648
C -0.3984054 -0.1244174 -0.0504597
H -1.4133759 -0.3830276 -0.3620074
H 0.1270396 0.3741423 -0.8658644
C -0.3876519 0.5467432 1.3563779
H 0.5272756 1.1338986 1.4752120
C -0.1816777 -0.8678152 1.9702882
H 0.5158348 -0.9672899 2.8005622
H -1.1354573 -1.3412343 2.2180243
C -1.5742173 1.3602582 1.7865206
C -2.8741462 0.8386782 1.7232172
H -3.0321227 -0.1726140 1.3545324
C -3.9706217 1.5976967 2.1260485
H -4.9700121 1.1748058 2.0689819
C -3.7882363 2.8979486 2.6015908
H -4.6431066 3.4904062 2.9149372
C -2.5010327 3.4294824 2.6700836
H -2.3476545 4.4405116 3.0376545

| | | | |
|---|---|---|---|
| C | -1.4062297 | 2.6647342 | 2.2656902 |
| H | -0.4036191 | 3.0833045 | 2.3203571 |
| Cl | 2.2655755 | -1.1799764 | -1.2326910 |

**[*epi*-6a]**
E(TPSS-D3/def2-TZVP) = -865.1518755243 (conv)
Lowest Freq. = 19.66 cm^-1
39
epi-6a (007c1/opt)

| | | | |
|---|---|---|---|
| N | 1.2874735 | -1.0386382 | 1.1430122 |
| O | -0.3378715 | -2.1184368 | 0.1001786 |
| C | 1.8646533 | -2.4073560 | 1.0975147 |
| H | 2.8234064 | -2.3717183 | 0.5723521 |
| C | 0.7350128 | -3.1610002 | 0.3157028 |
| H | 1.0097425 | -3.4742024 | -0.6899827 |
| C | 0.2108764 | -4.2858520 | 1.2165906 |
| H | -0.8773671 | -4.2201470 | 1.3175235 |
| H | 0.4312783 | -5.2604084 | 0.7674495 |
| C | 0.9497866 | -4.0962750 | 2.5229689 |
| C | 0.8084950 | -4.8295107 | 3.6998601 |
| H | 0.0752303 | -5.6273295 | 3.7735728 |
| C | 1.6325655 | -4.5287193 | 4.7855601 |
| H | 1.5313391 | -5.0933823 | 5.7071329 |
| C | 2.5978585 | -3.5198834 | 4.6977121 |
| H | 3.2398958 | -3.3121501 | 5.5476278 |
| C | 2.7463410 | -2.7873164 | 3.5203324 |
| H | 3.5095528 | -2.0170599 | 3.4471130 |
| C | 1.9070395 | -3.0798579 | 2.4453533 |
| C | 0.1015384 | -0.9990913 | 0.6047871 |
| C | -0.5234789 | 0.3457334 | 0.6427636 |
| H | -1.5630509 | 0.3124885 | 0.9763091 |
| H | -0.5131067 | 0.7775974 | -0.3655105 |
| C | 0.4229085 | 1.1124437 | 1.6294201 |
| H | -0.0368173 | 1.0698216 | 2.6207400 |
| C | 1.7316226 | 0.2573204 | 1.6857827 |
| H | 2.5280558 | 0.6624116 | 1.0548094 |
| H | 2.1040521 | 0.1309783 | 2.7035895 |
| C | 0.6630471 | 2.5591789 | 1.2664173 |
| C | 1.2400524 | 2.9044324 | 0.0371469 |
| H | 1.5229249 | 2.1313856 | -0.6759323 |
| C | 1.4584470 | 4.2405141 | -0.2902816 |
| H | 1.9053659 | 4.4968961 | -1.2458563 |
| C | 1.1024807 | 5.2472301 | 0.6096479 |
| H | 1.2724137 | 6.2884682 | 0.3543635 |
| C | 0.5285298 | 4.9120299 | 1.8348314 |
| H | 0.2489560 | 5.6906831 | 2.5376489 |
| C | 0.3103677 | 3.5729633 | 2.1611276 |
| H | -0.1387829 | 3.3148398 | 3.1173166 |

**[6a]**
E(TPSS-D3/def2-TZVP) = -865.1530381910 (conv)
Lowest Freq. = 20.25 cm^-1
39
6a (008c1/opt)

| | | | |
|---|---|---|---|
| N | 1.7055111 | -0.1950939 | 0.5722263 |
| O | 2.4342451 | 0.2760117 | 2.6071215 |
| C | 3.1450884 | 0.0400828 | 0.2915306 |
| H | 3.5709112 | -0.8419999 | -0.1940848 |
| C | 3.6753567 | 0.2915209 | 1.7449249 |
| H | 4.2966856 | -0.5054950 | 2.1496041 |
| C | 4.3100847 | 1.6858157 | 1.7757634 |

| | | | |
|---|---|---|---|
| H | 3.9290366 | 2.2631100 | 2.6239349 |
| H | 5.3944585 | 1.5990271 | 1.9088684 |
| C | 3.9679367 | 2.2827663 | 0.4287541 |
| C | 4.2171650 | 3.5769435 | -0.0243373 |
| H | 4.6800242 | 4.3140639 | 0.6252964 |
| C | 3.8696642 | 3.9123195 | -1.3343339 |
| H | 4.0566639 | 4.9183060 | -1.6969687 |
| C | 3.2994733 | 2.9653799 | -2.1916346 |
| H | 3.0516947 | 3.2404744 | -3.2117034 |
| C | 3.0542925 | 1.6671782 | -1.7445126 |
| H | 2.6258383 | 0.9291217 | -2.4170238 |
| C | 3.3802103 | 1.3452094 | -0.4269779 |
| C | 1.4241973 | 0.0043449 | 1.8291044 |
| C | 0.5143434 | -0.4120208 | -0.2680461 |
| H | 0.4825188 | -1.4573227 | -0.5886000 |
| H | 0.5561909 | 0.2391188 | -1.1427237 |
| C | -0.6527009 | -0.0223529 | 0.6948356 |
| H | -1.4575171 | -0.7546938 | 0.6127749 |
| C | -0.0225699 | -0.0927639 | 2.1350037 |
| H | -0.3572492 | 0.7174270 | 2.7867467 |
| H | -0.2241829 | -1.0437043 | 2.6428937 |
| C | -1.2065065 | 1.3592529 | 0.4084448 |
| C | -0.3599777 | 2.4428395 | 0.1352467 |
| H | 0.7182028 | 2.3075404 | 0.0793191 |
| C | -0.8883388 | 3.7134657 | -0.0844016 |
| H | -0.2218424 | 4.5431537 | -0.3000663 |
| C | -2.2681824 | 3.9170806 | -0.0325870 |
| H | -2.6793895 | 4.9063908 | -0.2070533 |
| C | -3.1167768 | 2.8439824 | 0.2367390 |
| H | -4.1914604 | 2.9935307 | 0.2713520 |
| C | -2.5876448 | 1.5719741 | 0.4561417 |
| H | -3.2542316 | 0.7373368 | 0.6604005 |

**TS(*epi*-5a-6a)**
E(TPSS-D3/def2-TZVP) = -1325.509578944 (conv)
Lowest Freq. = -400.68 cm^-1
40
TS_epi-5a-6a (009c1/opt)

| | | | |
|---|---|---|---|
| N | 0.9840033 | -0.7227130 | 1.1073615 |
| O | -0.0151855 | -2.4524585 | -0.0146914 |
| C | 1.8501766 | -1.9186106 | 1.3408098 |
| H | 2.8705517 | -1.6098400 | 1.1163565 |
| C | 1.2950178 | -3.0083095 | 0.3880147 |
| H | 1.8622512 | -3.1268303 | -0.5338152 |
| C | 1.0946410 | -4.2902118 | 1.2071411 |
| H | 0.1307871 | -4.7572564 | 0.9808423 |
| H | 1.8742338 | -5.0203559 | 0.9560548 |
| C | 1.2261066 | -3.8344442 | 2.6442215 |
| C | 0.9941522 | -4.5619851 | 3.8117113 |
| H | 0.6387734 | -5.5882787 | 3.7642782 |
| C | 1.2257584 | -3.9549860 | 5.0474163 |
| H | 1.0449916 | -4.5110663 | 5.9628927 |
| C | 1.6955144 | -2.6388143 | 5.1182227 |
| H | 1.8784497 | -2.1832601 | 6.0870849 |
| C | 1.9311720 | -1.9095344 | 3.9523142 |
| H | 2.2909298 | -0.8852532 | 4.0010750 |
| C | 1.6822899 | -2.5144177 | 2.7213254 |
| C | -0.0959650 | -1.2117535 | 0.4768290 |
| C | -1.2178553 | -0.3067352 | 0.0545857 |
| H | -2.0259517 | -0.8937371 | -0.3849441 |
| H | -0.8547767 | 0.4262347 | -0.6724819 |

| | | | |
|---|---|---|---|
| C | -1.5970057 | 0.2510714 | 1.4546060 |
| H | -2.6790163 | 0.1866215 | 1.6202387 |
| C | -0.8860928 | -0.8207128 | 2.2773947 |
| H | -0.2845573 | -0.5420693 | 3.1337671 |
| H | -1.3875572 | -1.7851310 | 2.3736639 |
| C | -1.1548150 | 1.6649626 | 1.7680235 |
| C | -0.0153717 | 2.2416915 | 1.1939546 |
| H | 0.5935287 | 1.6822717 | 0.4878425 |
| C | 0.3631989 | 3.5382252 | 1.5431687 |
| H | 1.2498049 | 3.9718482 | 1.0892622 |
| C | -0.3837142 | 4.2729453 | 2.4645065 |
| H | -0.0851056 | 5.2833631 | 2.7295473 |
| C | -1.5210564 | 3.7047993 | 3.0389365 |
| H | -2.1157235 | 4.2701532 | 3.7512020 |
| C | -1.9016530 | 2.4098440 | 2.6897788 |
| H | -2.7944482 | 1.9724180 | 3.1330063 |
| Cl | 2.1269210 | -0.0688632 | -0.9435444 |

**TS(5a-6a)**
E(TPSS-D3/def2-TZVP) = -1325.509034637 (conv)
Lowest Freq. = -394.01 cm^-1
40
TS_5a-6a (010c1/opt)

| | | | |
|---|---|---|---|
| N | 1.6282463 | -1.3522750 | 0.6779497 |
| O | 0.0390314 | -2.9407894 | 0.2276094 |
| C | 2.3235638 | -2.6734567 | 0.7852619 |
| H | 3.2320202 | -2.5970807 | 0.1892486 |
| C | 1.3033707 | -3.7094004 | 0.2478842 |
| H | 1.4794581 | -4.0160690 | -0.7817535 |
| C | 1.2062439 | -4.8488774 | 1.2707545 |
| H | 0.1639065 | -5.1303903 | 1.4515079 |
| H | 1.7197703 | -5.7399191 | 0.8883575 |
| C | 1.9099463 | -4.3061982 | 2.4957021 |
| C | 1.9974844 | -4.8697263 | 3.7688008 |
| H | 1.4902995 | -5.8035847 | 3.9979795 |
| C | 2.7484593 | -4.2191564 | 4.7495585 |
| H | 2.8203440 | -4.6471573 | 5.7453679 |
| C | 3.4168415 | -3.0246324 | 4.4594896 |
| H | 4.0056203 | -2.5357094 | 5.2302594 |
| C | 3.3329144 | -2.4600594 | 3.1862736 |
| H | 3.8477918 | -1.5311100 | 2.9560771 |
| C | 2.5660983 | -3.1029514 | 2.2156629 |
| C | 0.3340439 | -1.6720117 | 0.5179546 |
| C | -0.7173279 | -0.6092086 | 0.3931505 |
| H | -1.7095615 | -1.0639256 | 0.4335540 |
| H | -0.5976146 | -0.0544555 | -0.5419339 |
| C | -0.3861336 | 0.1705432 | 1.6934187 |
| H | 0.3483639 | 0.9464207 | 1.4664818 |
| C | 0.3142547 | -0.9593184 | 2.4262181 |
| H | 1.2012122 | -0.7714715 | 3.0196217 |
| H | -0.3172798 | -1.7637783 | 2.8060561 |
| C | -1.5483957 | 0.7776668 | 2.4461273 |
| C | -2.6588804 | 0.0057347 | 2.8138514 |
| H | -2.7005153 | -1.0478975 | 2.5465164 |
| C | -3.7179541 | 0.5728344 | 3.5192082 |
| H | -4.5723510 | -0.0390649 | 3.7944425 |
| C | -3.6821916 | 1.9234250 | 3.8710003 |
| H | -4.5081955 | 2.3661799 | 4.4198753 |
| C | -2.5812257 | 2.7005500 | 3.5122241 |
| H | -2.5465299 | 3.7530272 | 3.7790639 |
| C | -1.5221543 | 2.1293322 | 2.8066550 |

| | | | |
|---|---|---|---|
| H | -0.6658898 | 2.7378668 | 2.5256234 |
| Cl | 2.0439377 | -1.1793294 | -1.7194748 |

**TS(epi-5a-6a),Cl-syn**
E(TPSS-D3/def2-TZVP) = -1325.501968780 (conv)
Lowest Freq. = -433.74 cm^-1
40
TS_epi-5a-6a_Cl-syn (023c1/opt)

| | | | |
|---|---|---|---|
| N | 0.3972881 | -0.9790682 | 0.9765273 |
| O | -1.2304914 | -2.5410445 | 0.5446296 |
| C | 1.0985010 | -2.3060249 | 0.9715837 |
| H | 1.7532315 | -2.2919019 | 0.0926728 |
| C | -0.0445403 | -3.3503957 | 0.8776688 |
| H | 0.0723142 | -4.0591419 | 0.0558325 |
| C | -0.1421340 | -4.0372432 | 2.2507914 |
| H | -0.9615666 | -3.5876519 | 2.8229572 |
| H | -0.3216125 | -5.1129773 | 2.1610544 |
| C | 1.1801741 | -3.6998904 | 2.9026592 |
| C | 1.7268996 | -4.2289345 | 4.0680438 |
| H | 1.2129007 | -5.0150421 | 4.6149132 |
| C | 2.9434912 | -3.7259003 | 4.5349051 |
| H | 3.3787398 | -4.1302879 | 5.4444603 |
| C | 3.6063140 | -2.7061251 | 3.8459827 |
| H | 4.5498964 | -2.3242167 | 4.2247240 |
| C | 3.0638659 | -2.1784820 | 2.6734757 |
| H | 3.5706018 | -1.3794871 | 2.1395478 |
| C | 1.8507725 | -2.6818303 | 2.2173511 |
| C | -0.8421991 | -1.2586960 | 0.5403752 |
| C | -0.0556967 | -0.4457825 | -1.1044413 |
| H | -0.3956512 | -1.2283219 | -1.7855900 |
| H | 0.9975737 | -0.1978702 | -1.1862459 |
| C | -1.0677207 | 0.6445214 | -0.7514139 |
| H | -1.7037704 | 0.8535068 | -1.6194187 |
| C | -1.8489585 | -0.1640842 | 0.3241420 |
| H | -2.0417631 | 0.3571990 | 1.2656320 |
| H | -2.7774249 | -0.5815397 | -0.0686670 |
| C | -0.4381040 | 1.9450540 | -0.2999135 |
| C | -0.0033259 | 2.1503449 | 1.0151848 |
| H | -0.1202022 | 1.3712394 | 1.7650888 |
| C | 0.6026643 | 3.3557596 | 1.3700510 |
| H | 0.9342982 | 3.5009267 | 2.3943106 |
| C | 0.7824057 | 4.3662922 | 0.4249500 |
| H | 1.2518943 | 5.3039944 | 0.7089623 |
| C | 0.3487698 | 4.1688571 | -0.8862960 |
| H | 0.4749259 | 4.9520331 | -1.6287843 |
| C | -0.2578434 | 2.9652133 | -1.2427620 |
| H | -0.6028332 | 2.8171853 | -2.2645165 |
| Cl | -0.3836800 | -0.8096525 | 3.2940564 |

**TS(Cl-Inv *epi*-5a)**
E(TPSS-D3/def2-TZVP) = -1325.526105262 (conv)
Lowest Freq. = -222.03 cm^-1
40
TS_Cl-Inv_epi-5a (011c1/opt)

| | | | |
|---|---|---|---|
| N | 1.6396463 | -1.2739436 | 1.6021852 |
| O | -0.4206949 | -2.1135738 | 1.4587518 |
| C | 1.8669833 | -2.6795886 | 1.3403264 |
| H | 2.6489599 | -2.8087763 | 0.5801151 |
| C | 0.4619830 | -3.0729771 | 0.8260592 |
| H | 0.3699017 | -3.0033708 | -0.2642380 |
| C | 0.1459272 | -4.4577124 | 1.4005513 |

| | | | |
|---|---|---|---|
| H | -0.9115870 | -4.5281679 | 1.6742419 |
| H | 0.3499020 | -5.2357919 | 0.6534317 |
| C | 1.0841691 | -4.5748528 | 2.5826648 |
| C | 1.0646513 | -5.5062580 | 3.6191626 |
| H | 0.2955464 | -6.2736475 | 3.6628714 |
| C | 2.0468802 | -5.4376245 | 4.6099406 |
| H | 2.0413372 | -6.1560534 | 5.4250023 |
| C | 3.0362715 | -4.4505410 | 4.5640603 |
| H | 3.7924953 | -4.4095034 | 5.3430019 |
| C | 3.0563369 | -3.5165287 | 3.5260891 |
| H | 3.8195773 | -2.7440971 | 3.4955102 |
| C | 2.0722250 | -3.5849389 | 2.5421298 |
| C | 0.2514293 | -0.8661788 | 1.6072142 |
| C | -0.2335602 | 0.2372716 | 0.6282291 |
| H | -0.6639951 | -0.0958609 | -0.3195478 |
| H | 0.5607430 | 0.9663216 | 0.4412150 |
| C | -1.2070040 | 0.7373416 | 1.7428474 |
| H | -2.1845112 | 0.2614023 | 1.6246909 |
| C | -0.3497665 | -0.0609267 | 2.7763497 |
| H | 0.4128427 | 0.5755271 | 3.2344178 |
| H | -0.8675065 | -0.6381183 | 3.5456307 |
| C | -1.3817624 | 2.2160185 | 1.9365812 |
| C | -0.2751055 | 3.0580919 | 2.1202854 |
| H | 0.7280411 | 2.6368506 | 2.1245782 |
| C | -0.4409246 | 4.4296742 | 2.2977787 |
| H | 0.4297888 | 5.0645143 | 2.4385953 |
| C | -1.7204969 | 4.9893880 | 2.2953172 |
| H | -1.8498299 | 6.0590308 | 2.4338735 |
| C | -2.8296539 | 4.1642777 | 2.1140923 |
| H | -3.8300467 | 4.5887111 | 2.1108694 |
| C | -2.6581497 | 2.7907656 | 1.9367185 |
| H | -3.5266654 | 2.1507995 | 1.7961865 |
| Cl | 2.8965008 | -0.1665810 | 1.8807619 |

**TS(Cl-Inv 5a)**
E(TPSS-D3/def2-TZVP) = -1325.524338684 (conv)
Lowest Freq. = -229.55 cm^-1
40
TS_Cl-Inv_5a (012c1/opt)

| | | | |
|---|---|---|---|
| N | 1.5759426 | -1.3505336 | 1.2047889 |
| O | -0.4017356 | -2.3708914 | 1.3416800 |
| C | 1.9136894 | -2.7535975 | 1.1005090 |
| H | 2.6433651 | -2.9130032 | 0.2954853 |
| C | 0.5198363 | -3.3339803 | 0.7704606 |
| H | 0.3369117 | -3.4265865 | -0.3062326 |
| C | 0.3832048 | -4.6472621 | 1.5484528 |
| H | -0.6374153 | -4.7671223 | 1.9254224 |
| H | 0.5995621 | -5.5018650 | 0.8940951 |
| C | 1.4199801 | -4.5214399 | 2.6442109 |
| C | 1.5729952 | -5.2992147 | 3.7905986 |
| H | 0.8868379 | -6.1150467 | 4.0049410 |
| C | 2.6204125 | -5.0120507 | 4.6688732 |
| H | 2.7492424 | -5.6090245 | 5.5674941 |
| C | 3.5035950 | -3.9606139 | 4.4039376 |
| H | 4.3123208 | -3.7489694 | 5.0978446 |
| C | 3.3502556 | -3.1801606 | 3.2561883 |
| H | 4.0301629 | -2.3569919 | 3.0551440 |
| C | 2.3019688 | -3.4671018 | 2.3843508 |
| C | 0.1564814 | -1.0655045 | 1.3145615 |
| C | -0.4373486 | -0.0455656 | 0.2980040 |
| H | -1.4092101 | -0.3954934 | -0.0604432 |

| | | | |
|---|---|---|---|
| H | 0.1921126 | 0.2378858 | -0.5491270 |
| C | -0.5787884 | 0.9944918 | 1.4554008 |
| H | 0.2707487 | 1.6828589 | 1.4463269 |
| C | -0.3228894 | -0.1619168 | 2.4747329 |
| H | 0.3954834 | 0.0251701 | 3.2760276 |
| H | -1.2522409 | -0.5548148 | 2.8955487 |
| C | -1.8567427 | 1.7764873 | 1.5635884 |
| C | -3.1014392 | 1.1311238 | 1.6021462 |
| H | -3.1470337 | 0.0451594 | 1.5549879 |
| C | -4.2837887 | 1.8607926 | 1.7009978 |
| H | -5.2377151 | 1.3409840 | 1.7298335 |
| C | -4.2460592 | 3.2555593 | 1.7634401 |
| H | -5.1680687 | 3.8247427 | 1.8407535 |
| C | -3.0159022 | 3.9105629 | 1.7260894 |
| H | -2.9743124 | 4.9954192 | 1.7742783 |
| C | -1.8342477 | 3.1746930 | 1.6270097 |
| H | -0.8766634 | 3.6902325 | 1.5985483 |
| Cl | 2.7422202 | -0.1168517 | 1.1601909 |

**[*epi*-7a]**
E(TPSS-D3/def2-TZVP) = -1325.909778062 (conv)
Lowest Freq. = 12.99 cm^-1
41
epi-7a (015c2/opt)

| | | | |
|---|---|---|---|
| N | 1.3131298 | -1.8468796 | 0.0888759 |
| O | 2.6422985 | -1.9405257 | 2.3736524 |
| C | 1.4473967 | -3.2305879 | 0.7008522 |
| H | 0.4561726 | -3.4042173 | 1.1259597 |
| C | 2.5728250 | -3.2307544 | 1.7730406 |
| H | 2.2732386 | -3.9854645 | 2.5130319 |
| C | 3.8394054 | -3.7192640 | 1.0345593 |
| H | 4.4027843 | -2.8611781 | 0.6476385 |
| H | 4.5033466 | -4.2878094 | 1.6922850 |
| C | 3.2617564 | -4.5483621 | -0.0874839 |
| C | 3.8896985 | -5.4922398 | -0.8962332 |
| H | 4.9393874 | -5.7339468 | -0.7579323 |
| C | 3.1458623 | -6.1272597 | -1.8933586 |
| H | 3.6252843 | -6.8647731 | -2.5298425 |
| C | 1.7894237 | -5.8363058 | -2.0810343 |
| H | 1.2304817 | -6.3505571 | -2.8561309 |
| C | 1.1520055 | -4.8990816 | -1.2700529 |
| H | 0.0968391 | -4.6781222 | -1.4063267 |
| C | 1.9020057 | -4.2623549 | -0.2816528 |
| C | 0.5216935 | -0.9349242 | 0.5406323 |
| C | 0.3926329 | 0.5227253 | 0.2734408 |
| H | -0.3505481 | 0.7243949 | -0.5079741 |
| H | 1.3147459 | 1.0608542 | 0.0410848 |
| C | -0.2372980 | 0.6419563 | 1.7146206 |
| H | 0.5397313 | 0.9236195 | 2.4273683 |
| C | -0.4025682 | -0.9277597 | 1.7216265 |
| H | -0.0867143 | -1.4879340 | 2.6022977 |
| H | -1.4196851 | -1.2168676 | 1.4295452 |
| C | -1.4649835 | 1.4862616 | 1.8950705 |
| C | -2.5855669 | 1.3225200 | 1.0708532 |
| H | -2.5840141 | 0.5758809 | 0.2776166 |
| C | -3.7186284 | 2.1121890 | 1.2513431 |
| H | -4.5804480 | 1.9771072 | 0.6050691 |
| C | -3.7438761 | 3.0761926 | 2.2610464 |
| H | -4.6266720 | 3.6919416 | 2.4018962 |
| C | -2.6332882 | 3.2457994 | 3.0864970 |
| H | -2.6471818 | 3.9943213 | 3.8726998 |

| | | | |
|---|---|---|---|
| C | -1.4994863 | 2.4540450 | 2.9040957 |
| H | -0.6341738 | 2.5899126 | 3.5484010 |
| Cl | 2.4053592 | -1.4375732 | -1.1876405 |
| H | 3.2681320 | -1.9733142 | 3.1143339 |

**[epi-7a(conf2)]**
E(TPSS-D3/def2-TZVP) = -1325.907731854 (conv)
Lowest Freq. = 7.96 cm^-1
41
epi-7a_conf2 (020c1/opt)

| | | | |
|---|---|---|---|
| N | 1.4786205 | -1.5855993 | 0.3482927 |
| O | -0.9263649 | -2.5524785 | 0.5370301 |
| C | 1.4247733 | -3.0167074 | 0.7755081 |
| H | 2.1851605 | -3.5140173 | 0.1674219 |
| C | 0.0113667 | -3.6335858 | 0.4983349 |
| H | -0.0164861 | -4.1203099 | -0.4820086 |
| C | -0.1935517 | -4.6226830 | 1.6703750 |
| H | -1.2483051 | -4.6693892 | 1.9597201 |
| H | 0.1065829 | -5.6336243 | 1.3662794 |
| C | 0.7151905 | -4.1105436 | 2.7606596 |
| C | 0.7608444 | -4.4683431 | 4.1072784 |
| H | 0.0378231 | -5.1648300 | 4.5217136 |
| C | 1.7569311 | -3.9218737 | 4.9183238 |
| H | 1.7987743 | -4.1887243 | 5.9697624 |
| C | 2.7137185 | -3.0456134 | 4.3916423 |
| H | 3.4917784 | -2.6467324 | 5.0346084 |
| C | 2.6761419 | -2.6893518 | 3.0439868 |
| H | 3.4215704 | -2.0132743 | 2.6323707 |
| C | 1.6563112 | -3.2131265 | 2.2499599 |
| C | 0.9974020 | -0.5610202 | 0.9696198 |
| C | 0.3994850 | -0.3505725 | 2.3308195 |
| H | 0.9520550 | -0.7703975 | 3.1738317 |
| H | -0.6244968 | -0.7384973 | 2.3536493 |
| C | 0.4870423 | 1.1963618 | 2.0692905 |
| H | 1.3267566 | 1.6277574 | 2.6177212 |
| C | 0.9134578 | 0.8756572 | 0.5870869 |
| H | 0.1090492 | 1.0439713 | -0.1406317 |
| H | 1.8330504 | 1.3232009 | 0.1965545 |
| C | -0.7540096 | 2.0142385 | 2.2814097 |
| C | -1.9670903 | 1.6565624 | 1.6772285 |
| H | -2.0263089 | 0.7730237 | 1.0430336 |
| C | -3.1118945 | 2.4236972 | 1.8789344 |
| H | -4.0455720 | 2.1376586 | 1.4041376 |
| C | -3.0584482 | 3.5591893 | 2.6898622 |
| H | -3.9506722 | 4.1571914 | 2.8470332 |
| C | -1.8567529 | 3.9219778 | 3.2961638 |
| H | -1.8088718 | 4.8038686 | 3.9275019 |
| C | -0.7110727 | 3.1526695 | 3.0923782 |
| H | 0.2249064 | 3.4393479 | 3.5659200 |
| Cl | 2.0587743 | -1.3454656 | -1.2730107 |
| H | -1.8027671 | -2.9045453 | 0.3150177 |

**[7a]**
E(TPSS-D3/def2-TZVP) = -1325.909419501 (conv)
Lowest Freq. = 17.22 cm^-1
41
7a (016c2/opt)

| | | | |
|---|---|---|---|
| N | 2.1851916 | -1.5267632 | 0.7055860 |
| O | 2.5332135 | -1.6196313 | 3.3243504 |
| C | 1.8781145 | -2.8682309 | 1.3482640 |
| H | 0.7859363 | -2.8804280 | 1.3749544 |

| | | | |
|---|---|---|---|
| C | 2.5113753 | -2.9299413 | 2.7666670 |
| H | 1.8546629 | -3.5880981 | 3.3516422 |
| C | 3.8793853 | -3.6214378 | 2.5695002 |
| H | 4.6593442 | -2.8683870 | 2.4019377 |
| H | 4.1681999 | -4.2152205 | 3.4416034 |
| C | 3.6458523 | -4.4555760 | 1.3326052 |
| C | 4.3856825 | -5.5289630 | 0.8430882 |
| H | 5.2629503 | -5.8882917 | 1.3728522 |
| C | 3.9807500 | -6.1405167 | -0.3455567 |
| H | 4.5502067 | -6.9782469 | -0.7364339 |
| C | 2.8473806 | -5.6981169 | -1.0380435 |
| H | 2.5478384 | -6.1959281 | -1.9546128 |
| C | 2.0967723 | -4.6301989 | -0.5497504 |
| H | 1.2110966 | -4.2902985 | -1.0796925 |
| C | 2.5105288 | -4.0185726 | 0.6333455 |
| C | 1.4454913 | -0.4809684 | 0.8428156 |
| C | 1.5458467 | 0.9168155 | 0.3419761 |
| H | 1.8754699 | 1.0402171 | -0.6937378 |
| H | 2.1917155 | 1.5217432 | 0.9910421 |
| C | 0.0159491 | 1.0848351 | 0.6858072 |
| H | -0.5819244 | 0.9856207 | -0.2223260 |
| C | 0.0830580 | -0.2958584 | 1.4413519 |
| H | 0.1568622 | -0.1795738 | 2.5286300 |
| H | -0.6699390 | -1.0557385 | 1.2127925 |
| C | -0.4077312 | 2.2943477 | 1.4673908 |
| C | 0.2247115 | 2.6351112 | 2.6705780 |
| H | 1.0377266 | 2.0231089 | 3.0590419 |
| C | -0.1764060 | 3.7602176 | 3.3867656 |
| H | 0.3219521 | 4.0151708 | 4.3170596 |
| C | -1.2168797 | 4.5588992 | 2.9079635 |
| H | -1.5291603 | 5.4361219 | 3.4659075 |
| C | -1.8523398 | 4.2270884 | 1.7123678 |
| H | -2.6613951 | 4.8447700 | 1.3349231 |
| C | -1.4492276 | 3.0999108 | 0.9962594 |
| H | -1.9462223 | 2.8446611 | 0.0633509 |
| Cl | 3.7031423 | -1.3622329 | -0.1063753 |
| H | 2.7991513 | -1.6802788 | 4.2553915 |

## [7a(conf2)]

E(TPSS-D3/def2-TZVP) = -1325.909178784 (conv)
Lowest Freq. = 22.98 cm^-1
41
7a_conf2 (016c1/opt)

| | | | |
|---|---|---|---|
| N | 1.4950940 | -1.6252332 | 0.2761917 |
| O | -0.7601427 | -2.9215803 | 0.2279658 |
| C | 1.5736061 | -3.0264474 | 0.7852756 |
| H | 2.4802888 | -3.4366091 | 0.3316517 |
| C | 0.3195073 | -3.8544351 | 0.3497162 |
| H | 0.4922316 | -4.3601770 | -0.6059209 |
| C | 0.1075602 | -4.8342497 | 1.5273177 |
| H | -0.9548690 | -5.0621728 | 1.6604617 |
| H | 0.6215225 | -5.7825879 | 1.3235288 |
| C | 0.7363412 | -4.1367061 | 2.7091060 |
| C | 0.6166304 | -4.4227526 | 4.0675296 |
| H | -0.0439590 | -5.2126868 | 4.4129223 |
| C | 1.3650600 | -3.6798091 | 4.9831475 |
| H | 1.2749664 | -3.8894231 | 6.0444731 |
| C | 2.2416159 | -2.6779600 | 4.5512916 |
| H | 2.8285668 | -2.1251162 | 5.2777502 |
| C | 2.3704760 | -2.3920131 | 3.1922082 |
| H | 3.0581520 | -1.6204425 | 2.8548823 |

| | | | |
|---|---|---|---|
| C | 1.5938098 | -3.1154866 | 2.2887086 |
| C | 0.8471090 | -0.6401027 | 0.8036097 |
| C | 0.4709654 | 0.6961765 | 0.2680604 |
| H | 0.2615179 | 0.7541728 | -0.8028352 |
| H | 1.2144133 | 1.4580425 | 0.5339616 |
| C | -0.7228045 | 0.6770944 | 1.2993064 |
| H | -1.6239393 | 0.3133729 | 0.8023866 |
| C | -0.0074917 | -0.5197861 | 2.0299281 |
| H | 0.5879066 | -0.1670086 | 2.8803439 |
| H | -0.5800101 | -1.3989438 | 2.3207565 |
| C | -1.0071320 | 1.9220093 | 2.0880757 |
| C | 0.0062466 | 2.5749941 | 2.8010793 |
| H | 1.0244692 | 2.1877059 | 2.7931591 |
| C | -0.2711126 | 3.7286385 | 3.5305878 |
| H | 0.5232225 | 4.2264314 | 4.0782724 |
| C | -1.5689215 | 4.2429021 | 3.5561920 |
| H | -1.7855818 | 5.1422561 | 4.1241360 |
| C | -2.5842466 | 3.5991759 | 2.8502619 |
| H | -3.5949193 | 3.9953494 | 2.8655567 |
| C | -2.3040787 | 2.4441525 | 2.1200610 |
| H | -3.0978922 | 1.9452428 | 1.5693146 |
| Cl | 2.1880239 | -1.4012830 | -1.3012269 |
| H | -1.5456657 | -3.4040266 | -0.0744351 |

## TClCA

E(TPSS-D3/def2-TZVP) = -1885.169984064 (conv)
Lowest Freq. = 63.94 cm^-1
12
TClCA (004c1/opt)

| | | | |
|---|---|---|---|
| N | -1.1767972 | -0.0205416 | -0.0410828 |
| C | -1.8158331 | 1.2334568 | 0.0258913 |
| C | -1.7654902 | -1.2959698 | -0.1509602 |
| N | -3.1717881 | -1.2187789 | -0.1927486 |
| N | -3.2180676 | 1.1072347 | -0.0304456 |
| C | -3.9853203 | -0.0695638 | -0.1392285 |
| O | -1.1440216 | -2.3241114 | -0.2027106 |
| O | -1.2357060 | 2.2825732 | 0.1196120 |
| O | -5.1868922 | -0.0905273 | -0.1812243 |
| Cl | -3.9932103 | -2.7042306 | -0.3252608 |
| Cl | -4.0979914 | 2.5630019 | 0.0427783 |
| Cl | 0.5245153 | 0.0091517 | 0.0182833 |

## DClCA

E(TPSS-D3/def2-TZVP) = -1425.590918119 (conv)
Lowest Freq. = 71.24 cm^-1
12
DClCA (005c1/opt)

| | | | |
|---|---|---|---|
| H | -0.1595674 | -0.0027726 | -0.0057007 |
| N | -1.1734078 | -0.0204880 | -0.0409412 |
| C | -1.7962478 | 1.2186792 | 0.0255175 |
| C | -1.7465057 | -1.2805329 | -0.1491798 |
| N | -3.1543767 | -1.2224567 | -0.1924421 |
| N | -3.2008156 | 1.1115214 | -0.0295427 |
| C | -3.9671877 | -0.0692470 | -0.1385232 |
| O | -1.1061815 | -2.3034250 | -0.1998666 |
| O | -1.1970652 | 2.2632006 | 0.1195507 |
| O | -5.1689370 | -0.0902212 | -0.1804163 |
| Cl | -3.9704998 | -2.7087460 | -0.3250175 |
| Cl | -4.0754894 | 2.5683337 | 0.0437598 |

## MeOH

E(TPSS-D3/def2-TZVP) = -115.7878547413 (conv)
Lowest Freq. = 306.57 cm^-1
6
MeOH (MeOH/opt)

| | | | |
|---|---|---|---|
| C | 0.0532832 | 0.0340370 | -0.0250131 |
| H | 0.4390129 | 0.9299669 | 0.4661430 |
| H | 0.3045437 | -0.8409780 | 0.5912958 |
| H | 0.5388405 | -0.0660872 | -1.0062562 |
| O | -1.3614385 | 0.2187004 | -0.1429820 |
| H | -1.7288805 | -0.5657135 | -0.5773151 |

## MeOH2+

E(TPSS-D3/def2-TZVP) = -116.0897113572 (conv)
Lowest Freq. = 246.56 cm^-1
7
MeOH2_kat (MeOH2/opt)

| | | | |
|---|---|---|---|
| C | 0.1418249 | 0.0304901 | 0.0064878 |
| H | 0.4546596 | 0.9628343 | 0.4708013 |
| H | 0.3244021 | -0.8502047 | 0.6200444 |
| H | 0.5005738 | -0.0638536 | -1.0158394 |
| O | -1.3759765 | 0.1888198 | -0.1496579 |
| H | -1.8001225 | -0.5581603 | -0.6259638 |
| H | -1.8427429 | 0.343000 | |
